# Exceptional three- to six-photon absorption at organometallic dendrimers†

**DOI:** 10.1039/d4sc01127a

**Published:** 2024-05-14

**Authors:** Ling Zhang, Mahbod Morshedi, Torsten Schwich, Rika Kobayashi, Mark G. Humphrey

**Affiliations:** a Research School of Chemistry, Australian National University Canberra ACT 2601 Australia Mark.Humphrey@anu.edu.au; b National Computational Infrastructure, Australian National University Canberra ACT 2601 Australia

## Abstract

The light-intensity dependence of multi-photon absorption (MPA) affords outstanding spatial control. Furthermore, compared to the higher-energy photons needed for analogous linear absorption, the lower-energy photons involved in MPA often correspond to important wavelengths, such as those of the biological and telecommunications “windows”. It is therefore of crucial importance to develop molecules that exhibit outstanding MPA cross-sections. However, although progress has been made with two-photon absorption, there is currently a dearth of efficient instantaneous *n*-photon absorbers (*n* > 2), a key reason being the scarcity of structure–property studies required to understand higher-order MPA. We herein report systematically-varied metallodendrimers up to third-generation in size, together with their nonlinear absorptive responses over the spectral range 600–2520 nm. We show that the dendrimers exhibit exceptional instantaneous three- to six-photon absorption cross-sections, with maximal values increasing with dendrimer generation and installation of solubilizing group, and we report that changing the groups at the dendrimer periphery can shift the wavelengths of the *n*PA maxima. We also describe time-dependent DFT studies that have facilitated assignment of the key linear and nonlinear transitions and disclosed the crucial role of the metal in the outstanding MPA performance.

## Abstract 2

Metallodendrimers exhibit two- to six-photon absorption at key spectral regions (near-infrared I, II, and III). Structure-nonlinear absorption studies have defined the key molecular components contributing to the exceptional performance.

## Graphical abstract

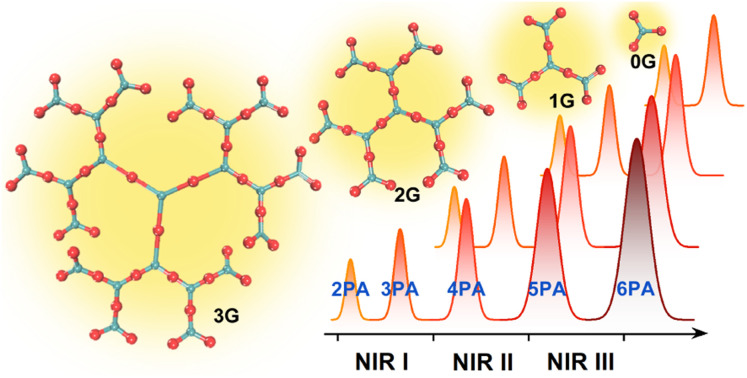

## 1 Introduction

Instantaneous multi-photon absorption (MPA) is a nonlinear optical (NLO) process involving the near simultaneous absorption of two or more photons. MPA has attracted ever-increasing interest from both the applied and fundamental perspectives.^1^ The light intensity dependence of MPA can afford exquisite three-dimensional control of interaction volume, because any subsequent “action” can be localized to the focal point of a laser, at which the light is sufficiently intense so as to manifest MPA effects. This 3D control has demonstrated or proposed uses in micromachining, data storage, photodynamic therapy, biological imaging, theranostics, and many other applications. Another practical advantage stems from the fact that the MPA excitation process involves *n*-photons of frequency (*ν*/*n*) for an overall transition energy *hν*. The use of such longer wavelength photons can result in decreased photo-damage, while simultaneously opening up technologically important spectroscopic regions (*e.g.* the windows where tissue and other biological material transparency are maximized: near-infrared I (NIR-I), 650–950 nm; NIR-II, 1000–1350 nm; NIR-III, 1550–1870 nm; and a newly proposed window, 2080–2340 nm).^2,3^ Essential to the development of MPA materials are wavelength-tunable laser systems to interrogate these spectral regions, with a particular need for ultra-short (femtosecond) low-repetition rate pulses to ensure instantaneous rather than stepwise effects are assayed.

The key to exploiting MPA is the development of materials with exceptional MPA merit. At the molecular level, MPA efficiency is usually quantified in terms of the *n*-photon absorption cross-sections *σ*_*n*_. Extensive research has uncovered structure-2PA (two-photon absorption) activity relationships that have identified key molecular design criteria to maximize *σ*_2_.^4–10^ Less is known of higher-order effects, although crucial advances have been made, largely with 3PA/*σ*_3_;^11,12^ reports of 4PA/*σ*_4_ have been much less frequent,^13,14^ while descriptions of 5PA/*σ*_5_ and even higher-order MPA are sparse (Table S1†).^15,16^ To some extent, this deficiency reflects the lack of routine access to the aforementioned low repetition rate, ultra-short pulse length long-wavelength-tunable lasers that are needed to drive the development of structure–activity relationships.

Dendrimers have attracted attention as putative NLO materials.^17–19^ Their monodisperse nature permits control of optical properties, and their hyperbranched structures facilitate maximization of both effective-chromophore density and molecular solubility. Organic dendrimers have commanded most attention, but organometallic dendrimers that exploit the flexibility in metal valence electron count and coordination geometry are also of interest. Square-planar platinum-containing dendrimers, for example, have been explored as optical limiters.^20^ Progression from 16 valence electron (VE) platinum to 18 VE metal centers is a logical approach to improve NLO responses because these properties are dependent on electron-richness as well as ease of polarization. With this in mind, we have previously carried out wavelength-dependence NLO studies of zero- and first-generation ruthenium alkynyl dendrimers,^21–26^ and reported their 2PA, 3PA and, in one case,^26^ 4PA activity. These interesting results have prompted us to examine the effect of varying dendrimer content in a more rigorous fashion, to assess the impact of dendrimer generation and composition on MPA. We herein report the syntheses and characterization of systematically-varied zeroth- to third-generation ruthenium alkynyl dendrimers with varying dendrimer framework linker length, peripheral functionalization, and solubilizing group incorporation, together with wide-wavelength femtosecond Z-scan studies that have disclosed their exceptional instantaneous 2PA–6PA performance, identified record MPA coefficients, and aided in the development of structure–MPA property relationships (the MPA studies of a few of the dendrimers that lack peripheral substituents have been reported in a preliminary fashion).^27^ We also report computational studies of linear optical properties and 2PA that permit assignment of the major linear and nonlinear optical transitions, and thereby help to rationalize the experimental observations.

## 2 Results and discussion

### 2.1 Synthesis and characterization of ruthenium alkynyl dendrimers

**Fig. 1:**
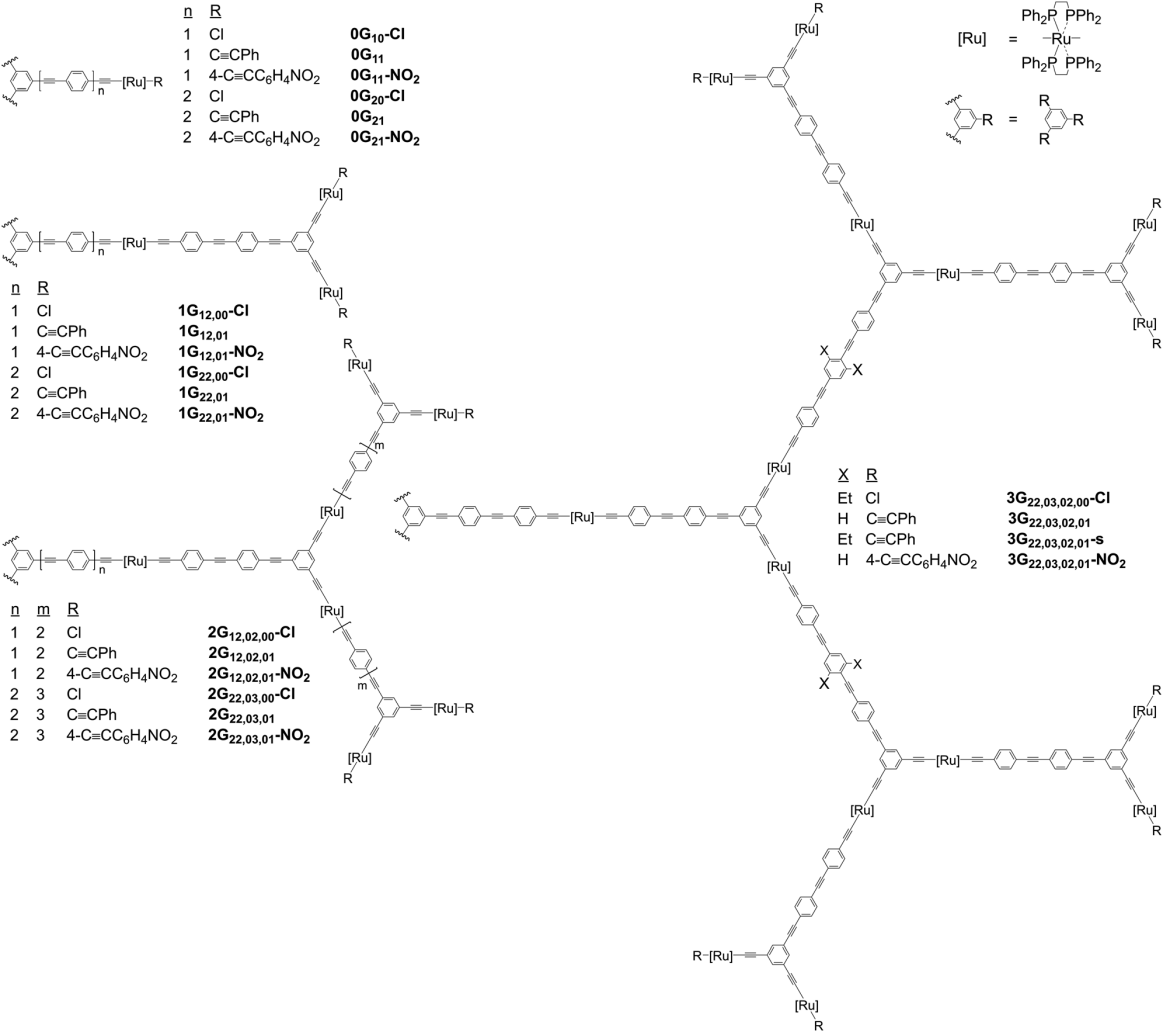
Organoruthenium dendrimers in this study. [Ru]: *trans*-[Ru(κ^2^-dppe)_2_] (dppe = 1,2-bis(diphenylphosphino)ethane). Wavy lines: equivalent dendrons at dendrimer cores. Names follow the format: (i) ***n*****G** (*n*-th generation dendrimer), (ii) ***xy*** (*x* and *y* phenyleneethynylene units between the branching points and the ligated Ru at each generation level, commencing at the core), (iii) a comma “,” separating the “*xy*” numbers of OPE units at each generation level, and in some cases (iv) **–s** (installation of solubilizing Et groups at the central phenylene of the OPE unit between the first- and second-generation branching points).

The systematically varied dendrimers in Fig. 1 permit assessment of the influence of dendrimer generation and composition on MPA. These ruthenium alkynyl dendrimers are large π-electron-delocalized macromolecules for which the constituent wedges and dendrons have rigid conjugated sub-units, and for which certain design considerations needed to be taken into account prior to synthesis. The 1,4-phenyleneethynylene (PE)-based linkages must be a minimum length to avoid insuperable steric congestion at the periphery of the second- and third-generation dendrimers. The design of these PE linkages also needs to consider the “effective conjugation length”,^28^ which is likely to be a key factor in maximizing NLO response scaled by molecular size. While the branching inherent in the dendritic construction aids solubility in comparison to that of similar-size linear analogues,^26^ it may still be insufficient to ensure the solubility of higher-generation dendrimers, so linear alkyl substituents were incorporated in the larger examples of the present study to enhance solubility (linear alkyl groups were chosen in preference to alternatives such as alkoxy groups, to avoid perturbing the OPE π-systems that contribute to the NLO effects). With these considerations in mind, the resultant suite of dendrimers depicted in Fig. 1 permits assessment of the impact on MPA merit of varying generation (zeroth- to third-generation), peripheral substituent (chlorido, phenylethynyl, 4-nitrophenylethynyl), core linker (1PE or 2PE to ruthenium), and OPE linker (0PE to 3PE between ruthenium centers and dendrimer branching points), as well as the effect of introducing solubilizing substituents (ethyl groups).

The synthetic procedures are described in the ESI† and are depicted in Schemes S1–S7.† The dendrimers were constructed by sequences of well-established procedures: vinylidene for chlorido ligand substitution, deprotonation of vinylidene ligands to form alkynyl ligands, protodesilylation of trialkylsilyl-protected alkynes to afford terminal alkynes, and Sonogashira palladium-catalyzed C–C coupling. Overall, the chlorido-terminated dendrimers were synthesized by divergent routes, while the 4-nitrophenyl-/phenyl-alkynyl-terminated dendrimers were accessed by a combination of divergent and convergent means. In all cases, the dendrimer syntheses exploited the steric control of the extent of metalation of a 1,3,5-trisubstituted arene that is possible with the sterically demanding ligated metal center *trans*-[Ru(κ^2^-dppe)_2_].^29^ This di- rather than tri-substitution, even in the presence of an excess of ruthenium reagent, affords rapid access to the dendrimer generation-defining 1,3,5-C_6_H_3_X_2_Y branching points, and thereby the key dendritic “wedge” intermediates, without the need for the protection/deprotection protocols required in analogous purely organic dendrimer synthesis.

The new complexes were initially characterized by a combination of ^1^H, ^13^C, and ^31^P NMR and IR spectroscopies, and satisfactory elemental analyses (Fig. S1–S45†). The ^31^P NMR spectra, in particular, proved useful in confirming reaction completion and product composition because resonances arising from the *trans*-[Ru(C≡CR)Cl(κ^2^-dppe)_2_] (49–50 ppm) and *trans*-[Ru(C≡CR)(C≡CR′)(κ^2^-dppe)_2_] (53–55 ppm) environments are well-separated. The dendrimers were further characterized by diffusion-ordered spectroscopy (DOSY: Fig. S46–S48†), size-exclusion chromatography (SEC), transmission electron microscopy (TEM), and MS. DOSY confirmed the product purity; the Stejskal–Tanner plot-derived diffusion coefficients revealed that the high-generation dendrimers, as expected, diffuse more slowly than the low-generation dendrimers (Fig. S49†) and, more importantly, that dendrimer diffusion is clearly distinguishable from that of possible impurities/by-products (such as branches, dendrons, wedges and other fragments). The Stokes–Einstein equation-derived hydrodynamic radii increase with increasing generation and installation of solubilizing ethyl groups, and upon proceeding from chlorido to phenylethynyl and then 4-nitrophenylethynyl peripheral ligands (Fig. S50†). The hydrodynamic radii of the dendrimers are smaller than the corresponding radii of gyration calculated from molecular mechanics-optimized geometries (Fig. S51†), with the disparity increasing with increasing generation, presumably due to the increasing proclivity of the dendrimer to rotate out of planarity. The SEC traces for all dendrimers exhibited a single peak, a narrow distribution for the number-averaged molecular weights (*M*_n_), and dispersity values consistent with the presence of uniform ruthenium dendrimers for which *M*_n_ values coincide with their formula weights (Fig. S52†). TEM micrographs of a representative example (2G_22,03,01_) revealed individual molecules with diameters of *ca.* 7.5 nm,^27^ consistent with that calculated by molecular modelling (Fig. S53†). MS proved to be of limited utility due to the low ionization efficiency of the dendrimers, the highest molecular weight example for which it proved useful being the chlorido-functionalized 1G_22,00_–Cl. This complex afforded MS signals that were simulated as corresponding to [M − 6Cl + *n*MeCN]^6+^ (*n* = 4–6),^27^ behavior that is consistent with the experimentally-observed facile ligand substitution at *trans*-[Ru(C≡CR)Cl(κ^2^-dppe)_2_].^30,31^

### 2.2 Experimental and computational studies of linear optical properties

The UV-vis-NIR spectra of the dendrimers are shown in Fig. S54 and S55,† with important data for the dendrimers and their precursors being listed in Tables 1 and S2,† respectively. Proceeding from chlorido- to phenylethynyl-terminated dendrimer results in little change in the lowest-energy bands, but an increase in absorptivity at wavelengths corresponding to higher-energy absorptions, while installation of nitro acceptor groups at the peripheral phenylethynyl ligands results in the appearance of a lower-energy band. Calculations on related monometallic model complexes suggest that the lowest energy band in the spectra of the chlorido- and phenylalkynyl-containing dendrimers corresponds to charge transfer from Ru to the OPE-containing ligand,^32^ while that in the spectra of the nitro-containing examples corresponds to ligated ruthenium to nitrophenyl charge transfer.^28,33^ Dendrimer generation increase while otherwise maintaining dendrimer composition results in a general increase in absorptivity scaling roughly with molecular size, but no change in profile.

**Table 1:** Linear optical absorption and two- to four-photon absorption cross-section maxima^a^

Complex	*λ*_max_ b [*ε*]^c^	*σ*_2_ d (*λ*_max_)^b^	*σ*_2_ e/M (*λ*_max_)^b^	*σ*_3_ f (*λ*_max_)^b^	*σ*_3_ g/M (*λ*_max_)^b^	*σ*_4_ h (*λ*_max_)^b^	*σ*_4_ i/M (*λ*_max_)^b^	Ref.
3G_22,03,02,01_–NO_2_	427 [82]	163 600 (750)	3.11 (750)	38 400 (1200)	0.73 (1200)	3800 (1600)	0.072 (1600)	This work
		123 000 (850)	2.34 (850)			3000 (1900)	0.057 (1900)	
3G_22,03,02,01_	418 [106]	113 200 (725)	2.20 (725)	22 200 (1200)	0.43 (1200)	5850 (1650)	0.11 (1650)	27
		45 900 (900)	0.89 (900)					
3G_22,03,02,01_–s	424 [81]	111 500 (700)	2.15 (700)	23 200 (1250)	0.45 (1250)	7300 (1650)	0.14 (1650)	27
		50 600 (900)	0.98 (900)					
3G_22,03,02,00_–Cl	412 [110]	113 700 (750)	2.26 (750)	23 100 (1200)	0.46 (1200)	4250 (1800)	0.085 (1800)	This work
				13 600 (1400)	0.27 (1400)			
2G_22,03,01_–NO_2_	430 [62]	42 200 (750)	1.68 (750)	15 600 (1200)	0.62 (1200)	2100 (1850)	0.083 (1850)	This work
				7600 (1450)	0.30 (1450)			
2G_22,03,01_	415 [49]	43 600 (700)	1.78 (700)	13 750 (1250)	0.56 (1250)	2950 (1650)	0.12 (1650)	27
		20 000 (900)	0.81 (900)					
2G_22,03,01_–s	421 [52]	42 450 (750)	1.71 (750)	15 000 (1250)	0.60 (1250)	3700 (1650)	0.15 (1650)	27
		16 000 (875)	0.64 (875)					
2G_22,03,00_–Cl	421 [45]	38 500 (700)	1.60 (700)	13 600 (1250)	0.56 (1250)	1000 (1750)	0.041 (1750)	This work
1G_22,01_–NO_2_	430 [33]	22 000 (680)	2.05 (680)	11 400 (1200)	1.06 (1200)	900 (1750)	0.084 (1750)	This work
						650 (1900)	0.060 (1900)	
1G_22,01_	427 [19]	13 700 (725)	1.31 (725)	4800 (1200)	0.46 (1200)	1200 (1650)	0.11 (1650)	27
		8800 (900)	0.84 (900)					
1G_22,00_–Cl	421 [20]	12 700 (680)	1.26 (680)	5800 (1200)	0.58 (1200)	300 (1850)	0.030 (1850)	This work
				2900 (1350)	0.29 (1350)			
0G_21_–NO_2_	418 [11.6]	6000 (875)	1.55 (875)	5000 (1200)	1.29 (1200)	180 (1750)	0.046 (1750)	45
0G_21_	422 [12.6]	3200 (900)	0.85 (900)	2300 (1200)	0.61 (1200)			45
0G_20_–Cl	424 [13.5]	9500 (650)	2.68 (650)	1800 (1200)	0.51 (1200)			45
		6000 (875)	1.69 (875)					
2G_12,02,01_–NO_2_	424 [63]	31 200 (850)	1.29 (850)	14 400 (1250)	0.60 (1250)	1300 (1850)	0.054 (1850)	This work
						600 (2000)	0.025 (2000)	
2G_12,02,01_	340 [96]	34 700 (750)	1.47 (750)	7950 (1250)	0.34 (1250)	2700 (1650)	0.11 (1650)	27
	409 [46]	18 000 (900)	0.76 (900)					
2G_12,02,00_–Cl	409 [46]	35 600 (700)	1.56 (700)	13 700 (1200)	0.60 (1200)	2200 (1750)	0.096 (1750)	This work
1G_12,01_–NO_2_	424 [32]	23 200 (850)	2.22 (850)	6200 (1200)	0.59 (1200)	400 (1950)	0.038 (1950)	This work
1G_12,01_	343 [38]	14 000 (725)	1.38 (725)	2500 (1250)	0.25 (1250)	900 (1650)	0.089 (1650)	27
	412 [18]	6300 (875)	0.62 (875)					
1G_12,00_–Cl	412 [18]	14 400 (750)	1.47 (750)	3200 (1200)	0.32 (1200)	650 (1600)	0.066 (1600)	This work
0G_11_–NO_2_	403 [11.2]	2500 (670)	0.70 (670)	740 (1290)	0.21 (1290)			45
		1100 (800)	0.31 (800)					
0G_11_	412 [11.6]	1490 (650)	0.43 (650)	100 (1240)	0.029 (1240)			45
		370 (810)	0.11 (810)					
0G_10_–Cl	413 [9.9]	1500 (680)	0.46 (680)	190 (1290)	0.059 (1290)			45
		1050 (845)	0.32 (845)					

a Solvent CH_2_Cl_2_.

b nm.

c 10^4^ L mol^−1^ cm^−1^.

d GM = 10^−50^ cm^4^ s^−1^ photon^−1^.

e GM mol g^−1^.

f 10^−80^ cm^6^ s^−2^ photon^−2^.

g 10^−80^ cm^6^ s^−2^ photon^−2^ mol^−1^ g^−1^.

h 10^−110^ cm^8^ s^−3^ photon^−3^.

i 10^−110^ cm^8^ s^−3^ photon^−3^ mol^−1^ g^−1^.

**Fig. 2:**
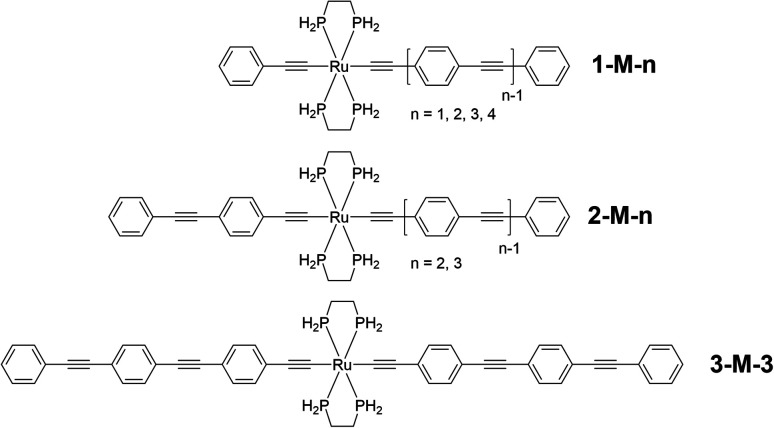
Computational models of the key dendrimer building blocks 1-M-*n* (*n* = 1–4), 2-M-*n* (*n* = 2, 3), and 3-M-3.

To shed more light on the UV-vis-NIR spectra, and in particular understand charge transfer within the dendritic interior, further computational studies were undertaken in the present work. Once again, due to the size of the dendrimers, it was necessary to approach assignment of the underlying transitions responsible for the linear absorption bands by undertaking calculations on monometallic models of the key components of the dendrimers, specifically, the *trans*-[Ru(C≡CR)(C≡CR′)(κ^2^-dppe)_2_] linear units (R = Ph, R′ = (1,4-C_6_H_4_C≡C)_0–3_Ph; R = 1,4-C_6_H_4_C≡CPh, R′ = (1,4-C_6_H_4_C≡C)_1-2_Ph; R = R′ = (1,4-C_6_H_4_C≡C)_2_Ph) that terminate at the 1,3,5-C_6_H_3_X_2_Y branching points in the dendritic structures. The phenyl groups of the experimental κ^2^-dppe ligands were replaced by H in the model complexes (Fig. 2: dHpe ligands), an approximation that previous studies on monometallic complexes had confirmed was an acceptable compromise to mitigate computational expense.^34^ The geometry-optimized coordinates are given in Tables S3–S9† (PBE0 (ref. 35)/6-31G(d),^36^ no symmetry constraints, D3BJ dispersion correction,^37^ PCM CH_2_Cl_2_ (ref. 38 and 39)). The low-energy absorption bands were calculated using both the hybrid functional PBE0 and the long-range corrected functional CAM-B3LYP (6G(d,p)/SDD non-metal/Ru basis sets)^40–43^ (Table S10†). The latter was found to significantly overestimate the transition energies and, as the former was found to better approximate the experimental data, PBE0 was employed for the subsequent linear absorption computations.

The transitions with calculated oscillator strengths greater than 0.3 are listed in Table S11,† isodensity plots of the key orbitals involved in the low-energy transitions are depicted in Fig. S56–S62,† and natural transition orbitals for the low-energy transitions are shown in Fig. S63.† A number of observations can be made. Contributions to these frontier orbitals are dominated by the OPE ligands and the metal, the exceptions being the smallest complexes for which there is charge-transfer involving the Ru(κ^2^-dHpe)_2_ moiety (1-M-2: LUMO+1; 1-M-3: LUMO+2). Increasing the number of phenylethynyl units in unsymmetrical complexes while otherwise maintaining complex composition (proceeding from 1-M-2 to 1-M-3 and finally 1-M-4) results in a diminishing LUMO ← HOMO contribution to, and a diminishing red-shift in, the lowest-energy band, which is predominantly C_2_RuC_2_Ph to OPE charge-transfer in character; a similar trend is seen proceeding from 1-M-2 to 2-M-3. Increasing the number of phenylethynyl units in symmetrical complexes (proceeding from 1-M-1 to 2-M-2 and then 3-M-3) results in diminishing LUMO ← HOMO contributions to, and diminishing red-shifts for, the lowest-energy bands, which correspond to symmetric charge transfer from the core C_2_RuC_2_ unit to the OPE ligands. Comparison of the positional isomers 1-M-3 and 2-M-2, and 1-M-4 and 2-M-3, reveals that the isomers with the phenylethynyl ligand (1-M-*n* (*n* = 3, 4)) exhibit the lower energy bands. The LUMOs of the 1-M-*n* complexes have significant contributions from all phenyl groups of the OPE ligand with the exception of 1-M-4, for which the proximal and distal rings of the OPE make comparatively small contributions (Fig. S59†), and consistent with prior reports suggesting limits in π-delocalization in such complexes.^28^

### 2.3 Experimental studies of nonlinear optical properties

**Fig. 3:**
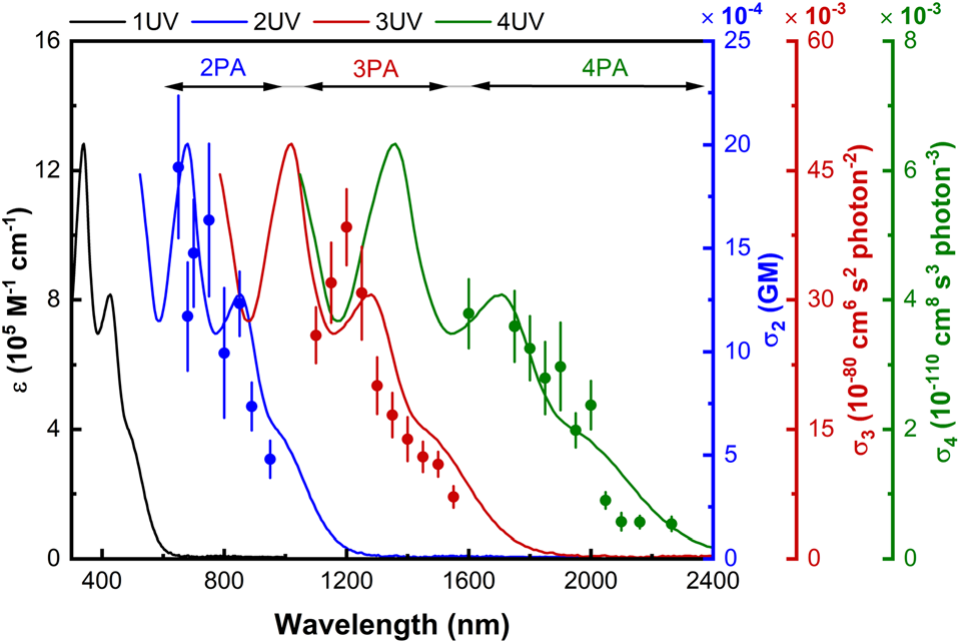
Wavelength dependence of the nonlinear absorption of 3G_22,03,02,01_–NO_2_. Plots of *σ*_2_ (blue scatter), *σ*_3_ (red scatter), and *σ*_4_ (green scatter), overlaid on the UV/Vis spectrum (black line), and including plots of the UV/Vis spectrum as a function of twice (blue line), three times (red line), and four times (green line) the wavelength.

**Fig. 4:**
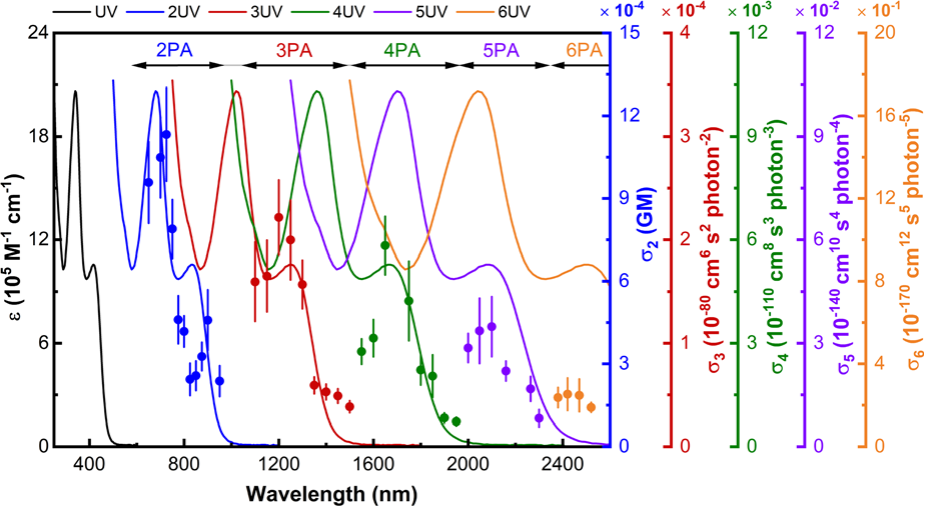
Wavelength dependence of the nonlinear absorption of 3G_22,03,02,01_. Plots of *σ*_2_ (blue scatter), *σ*_3_ (red scatter), *σ*_4_ (green scatter), *σ*_5_ (purple scatter), and *σ*_6_ (orange scatter) overlaid on the UV/Vis spectrum (black line), and including plots of the UV/Vis spectrum as a function of twice (blue line), three times (red line), four times (green line), five times (purple line), and six times (orange line) the wavelength.

The complexes in the present study are insufficiently emissive to employ multiphoton-excited fluorescence to measure their nonlinear absorption, so nonlinear absorptive and nonlinear refractive data for the dendrimers and their key precursors were obtained using a combination of open-aperture and closed-aperture Z-scan experiments over the spectral range 600–2520 nm, and employing low-repetition rate *ca.* 130 femtosecond light pulses. The open-aperture experiments revealed very large absorptive nonlinearities that have been replotted as the corresponding multi-photon absorption cross-sections in Fig. 3, 4 and S64–S84.† The multi-photon absorption plots also contain the linear optical absorption spectra plotted at twice to six-times the wavelength, to highlight the coincidence or near-coincidence of multiples of certain bands to MPA bands. In particular, the wavelengths of the maximal values of the intensity dependence-assigned MPA correspond closely to the appropriate multiples of the wavelengths of the low-energy MLCT bands (assigned by analogy with the low-energy bands of the model complexes which are predominantly LUMO ← HOMO and MLCT in nature), rather than multiples of the higher-energy, more intense, MLCT/ILCT/LLCT admixtures. This correlation with the low-energy MLCT bands emphasizes the importance of ligated metal modules and of certain types of charge transfer in the MPA performance. The closed-aperture experiments show negative or zero *γ*_real_ values for all compounds, with the negative maximal values occurring in regions of significant MPA; this is consistent with the anticipated dependence of *γ*_real_ on all nonlinear absorption processes through a nonlinear Kramers–Krönig relationship.^44^

The nonlinear absorption maximal values of the dendrimers are collected in Table 1, organized by core OPE linker (**G**_***x***_, *x* = 1, 2), dendrimer generation (***n*****G**, *n* = 0–3), and peripheral substituent (**–NO**_**2**_ = 4-nitrophenylethynyl, “blank” = phenylethynyl, **–Cl** = chlorido). The phenylethynyl-terminated dendrimers exhibit nonlinear absorption maxima at 700–750 nm, 875–900 nm, 1200–1250 nm, and 1650 nm, confirmed to be 2PA, 2PA, 3PA, and 4PA in nature, respectively, from the intensity dependencies of the corresponding open-aperture fs Z-scan traces (3G_22,03,02,01_–s: Fig. S85–S89†), and supported by the location of the *n*PA maxima at wavelengths close to the corresponding multiples of that of the linear absorption maxima. The chlorido-terminated dendrimers exhibit nonlinear absorption maxima at similar wavelength ranges of *ca.* 650–750 nm (2PA), 1200–1300 nm (3PA), and 1600–1850 nm (4PA), while the 4-nitrophenyl-terminated dendrimers show nonlinear absorption maxima at the red-shifted wavelengths of *ca.* 650–875 nm (2PA), 1200–1450 nm (3PA), and 1600–2000 nm (4PA); the red-shift following installation of peripheral nitro substituent mimics the corresponding multiple of the 1PA profile with its longer wavelength MLCT to the nitro group, and is of a sufficient extent that 3G_22,03,02,01_–NO_2_ exhibits appreciable 4PA at wavelengths beyond 2000 nm (Fig. 3). In general, the *σ*_*n*_ maximal values increase with generation increase and installation of nitro group; the increased activity accompanying generation increase results in measurable 4PA being observed for all *n*G (*n* > 0) examples. Focusing on the *σ*_4_ data, for which few precedent structure–activity guidelines exist, the 4PA cross-sections increase with dendrimer generation, installation of alkyl solubilizing groups, and progression from peripheral chlorido and nitrophenylalkynyl to phenylalkynyl ligand.

The maximal nonlinear absorption values of the precursors 16–18, 20, 24, and 35 were also determined and are collected in Table S2,† together with those of the key intermediates 22, 26, and 36. The data reveal significant 2PA and 3PA activity for all precursors and intermediates, and with the largest examples 35 and 36 also displaying measurable 4PA, outcomes consistent with the behavior of the dendrimers.

Various procedures to compare 2PA data for different molecules have been examined, including scaling the experimental data by molecular volume, cost of production,^46^ number of constituent chromophore units,^47^ and number of “effective” electrons,^48,49^ but the most commonly accepted approach to compare data is to scale by molecular weight, a procedure we have implemented for *σ*_*n*_ (*n* = 2–4) in Table 1. For the chlorido-terminated dendrimers, the *σ*_3_/M data reveal a super-linear increase on generation increase for examples with a 1,4-C_2_C_6_H_4_C_2_ core-[Ru] linkage, but the *σ*_3_/M data are relatively invariant for examples with a longer 1,4-C_2_C_6_H_4_C_2_–1,4-C_6_H_4_C_2_ core-[Ru] linkage (for example 0G_10_–Cl 0.059 (1290), 1G_12,00_–Cl 0.32 (1200), 2G_12,02,00_–Cl 0.60 (1200), *c.f.*0G_20_–Cl 0.51 (1200), 1G_22,00_–Cl 0.58 (1200), 2G_22,03,00_–Cl 0.56 (1250)). In general, the *σ*_3_/M data increase on introduction of nitro substituent while the *σ*_4_/M data are greater in the absence of the nitro group on the peripheral phenylalkynyl ligand.

The molecular weight-scaled performances of certain dendrimer synthesis intermediates also deserve comment (Table S2†). The hexaruthenium wedge precursor 26, with a strongly dipolar composition, exhibits the largest *σ*_2_/M value (4.44 GM mol^−1^ g^−1^ at 900 nm). The Sonogashira coupling precursors 35 and 36 are, effectively, organometallic dendrimers that are peripherally hexaiodo- and dodecaiodo-functionalized, respectively, and thereby benefit from a strong heavy-atom effect, with striking *σ*_2_/M parameters, the largest *σ*_3_/M parameter from the present study (36), significant *σ*_4_ activity, and the second largest *σ*_4_/M parameter (35).

As mentioned above, the maximal values increase with dendrimer generation and, indeed, the third-generation dendrimers and some of the second-generation examples are sufficiently active as to also show measurable higher-order MPA at longer wavelengths, the maximal data being collected in Table 2 and the wavelength dependencies being shown in Fig. 4, S65, S67 and S69,† with the assignments again being supported by intensity dependencies of the corresponding open-aperture fs Z-scan traces (3G_22,03,02,01_–s: Fig. S85–S89†). Note that the small red-shift in *σ*_*n*_ maxima seen on replacing phenylalkynyl by chlorido peripheral ligand and the larger red-shift that results from introduction of nitro substituent results in possible 6PA activity (chlorido) and 5PA and 6PA activity (nitrophenylalkynyl) being outside our measurement window. Reports of molecular 5PA are rare; excluding our dendrimer study, only two *σ*_5_ data are extant [(*E*)-3-(4-(2-(1-hexyl-4-methyl-1*H*-imidazole-5-yl)vinyl)pyridinium-1-yl)propyl sulfate] (0.00192 × 10^−140^ cm^10^ s^−4^ photon^−4^ at 2100 nm, fs nonlinear transmission)^16^ and a spiro-fused ladder-type oligo(*p*-phenylene) (9320 × 10^−140^ cm^10^ s^−4^ photon^−4^ at 1540 nm, 120 fs Z-scan and five-photon excited fluorescence).^15^ The present data reveal an increase in *σ*_5_ with generation increase, replacement of phenylalkynyl by chlorido ligand, and installation of solubilizing alkyl groups, and with the *σ*_5_/M data revealing that the dendrimer generation increases are super-linear in nature. Dendrimers 3G_22,03,02,01_–s and 3G_22,03,02,01_ display the first quantifiable molecular 6PA data for organometallics and only the second and third overall, the precedent being the aforementioned spiro-fused ladder-type oligo(*p*-phenylene) which is active at a much shorter incident wavelength (86.7 × 10^−170^ cm^12^ s^−5^ photon^−5^ at 1820 nm, 120 fs Z-scan and six-photon excited fluorescence).^15^ As with *σ*_4_ and *σ*_5_, the *σ*_6_ and *σ*_6_/M data increase with introduction of the ethyl solubilizing groups.

**Table 2:** Linear optical absorption and five- and six-photon absorption cross-section maxima^a^

Complex	*λ*_max_ b [*ε*]^c^	*σ*_5_ d (*λ*_max_)^b^	*σ*_5_ e/M (*λ*_max_)^b^	*σ*_6_ f (*λ*_max_)^b^	*σ*_6_ g/M (*λ*_max_)^b^
3G_22,03,02,01_ (ref. 27)	418 [106]	350 (2100)	0.0068 (2100)	25 (2420)	0.00049 (2420)
3G_22,03,02,01_–s^27^	424 [81]	500 (2050)	0.0096 (2050)	53 (2470)	0.0010 (2470)
3G_22,03,02,00_–Cl	412 [110]	500 (2300)	0.0100 (2300)		
2G_22,03,01_ (ref. 27)	415 [49]	100 (2160)	0.0041 (2160)		
2G_22,03,01_–s^27^	421 [52]	170 (2100)	0.0068 (2100)		
2G_22,03,00_–Cl	421 [45]	110 (2160)	0.0046 (2160)		

a Solvent CH_2_Cl_2_.

b nm.

c 10^4^ L mol^−1^ cm^−1^.

d 10^−140^ cm^10^ s^−4^ photon^−4^.

e 10^−140^ cm^10^ s^−4^ photon^−4^ mol^−1^ g^−1^.

f 10^−170^ cm^12^ s^−5^ photon^−5^.

g 10^−170^ cm^12^ s^−5^ photon^−5^ mol^−1^ g^−1^.

### 2.4 Computational studies of two-photon absorption

**Fig. 5:**
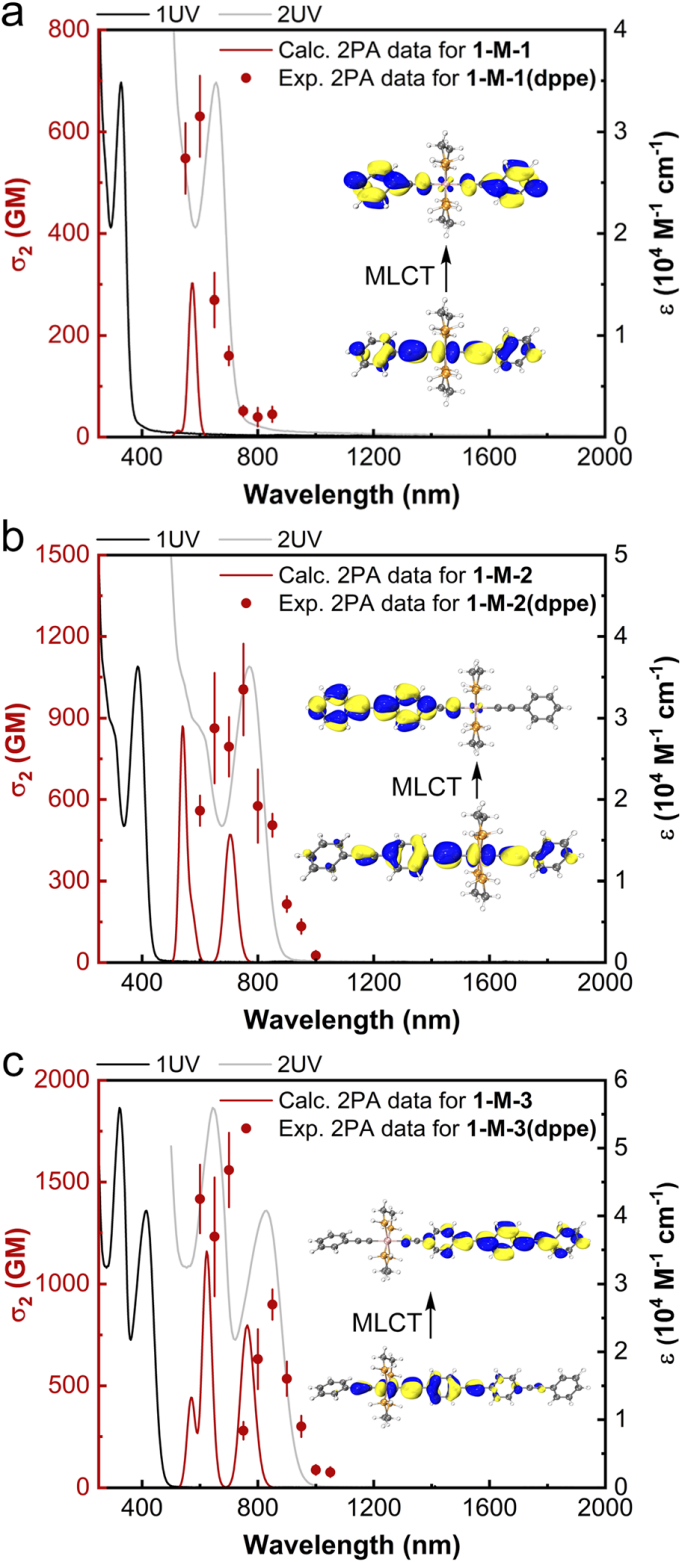
Calculated 2PA cross-sections of 1-M-*n* (*n* = 1–3) (red traces), experimental 2PA cross-sections of 1-M-*n*(dppe) (*n* = 1–3) (red circles), linear absorption spectra of 1-M-*n*(dppe) (*n* = 1–3) (black traces), and plots of the UV/Vis spectrum as a function of twice the wavelength (light gray traces). Insets: the metal-to-ligand charge transfer (MLCT) corresponding to the calculated low-energy 2PA maxima.

To shed further light on the outstanding nonlinear absorption behavior of the dendrimers, the 2PA cross-sections of the aforementioned dendritic fragment model complexes 1-M-*n* (*n* = 1–3) were calculated through a residue of the quadratic response function^50^ implemented in Dalton 2020.1 (ref. 51 and 52) (CAM-B3LYP, dHpe co-ligands, PCM^53^ CH_2_Cl_2_, stuttgart_rsc_1997_ecp^54^ (Ru) and 6+G(d)^55^ (non-metal atoms) basis sets; further details are given in the ESI†). Calculation of 2PA spectra is computationally demanding, so the two-photon transition strengths were computed using the ten lowest excited states only. Despite this necessary simplification, the calculations are generally in good agreement with the experimental results; the 2PA profiles are similar, with one (1-M-1/1-M-1(dppe)), two (1-M-2/1-M-2(dppe)), and three (1-M-3/1-M-3(dppe)) maxima in the 450 nm range, and the significant red shift in the lowest-energy 2PA maximum that is seen experimentally upon lengthening the OPE alkynyl ligand being reproduced computationally (Table 3 and Fig. 5). We note that the shortest wavelength 2PA maxima coincide with the onset of one-photon absorption and deconvoluting experimental data in this wavelength range is known to be difficult,^56^ so comments are necessarily cautious.

**Table 3:** Experimental^a^ and calculated^b^ 2PA maximal values

Complex	*σ*_2,exp_ (GM) (*λ*_max_ (nm))	Model	*σ*_2,calc_ (GM) (*λ*_max_ (nm))
1-M-1(dppe)	630 ± 80 (600)	1-M-1	290 (574)
1-M-2(dppe)	860 ± 200 (650)	1-M-2	850 (540)
	1000 ± 170 (750)		470 (704)
1-M-3(dppe)	1400 ± 170 (600)	1-M-3	370 (572)
	1560 ± 180 (700)		1100 (623)
	900 ± 70 (850)		800 (763)

a Ph_2_PCH_2_CH_2_PPh_2_ (dppe) ligands.

b H_2_PCH_2_CH_2_PH_2_ (dHpe) ligands.

The 2PA peak of 1-M-1 at 574 nm is attributed to the largest transition moment (4.32 eV (287 nm): Table S12†), corresponding to the linear MLCT transition at 285 nm (1-M-1 calc.) and 328 nm (1-M-1(dppe) exp.). Similarly, the lower-lying 2PA maximum of 1-M-2 and the lowest-lying 2PA maximum of 1-M-3 correspond to metal-to-alkynyl-ligand charge transfer, confirming the key role the metal plays in the MPA merit of these complexes.

The calculated 2PA cross-sections for 1-M-*n* (*n* = 1–3) are collected in Table 3, together with those of the experimental complexes 1-M-*n*(dppe) (*n* = 1–3). The increase in maximal values seen on proceeding from 1-M-1 to 1-M-3 reproduces the experimental trend seen with 1-M-*n*(dppe) (*n* = 1–3). The clear increase in *σ*_2_ (both maximal value and integrated intensity) upon OPE alkynyl ligand lengthening highlights the desirability of incorporating the longer OPE units in MPA materials, and confirms that the OPE-rich dendrimer composition in the present study is ideal for MPA efficiency.

## 3 Conclusions

The present study has identified optimal compositions for MPA-efficient molecules. The *trans*-[Ru(κ^2^-dppe)_2_] modules incorporate polarizable low oxidation state pseudo-octahedral electron-rich metal centers, for which stepwise chemistry at the two axial sites permits the designed construction of π-electron-delocalizable architectures in which the metal engages in strong low-energy MLCT interactions. The *trans*-[Ru(κ^2^-dppe)_2_] units impart greatly superior solubility and optical nonlinearity to those of analogous purely organic aryleneethynylene-based structures. The resultant metallodendrimers have been definitively identified by a combination of small molecule and macromolecule characterization techniques.

Multi-photon absorption studies have identified exceptional performance across a spectral range extending deep into the NIR region. The dendrimers exhibit outstanding 2PA, 3PA, and 4PA cross-sections. Installation of peripheral nitro substituents red-shifts the MPA maxima, such that exceptional 4PA is seen at wavelengths beyond 2000 nm. Structural modifications that enhance MPA merit have been identified: increasing the length of the OPE linkage to the core, installation of alkyl solubilizing groups, and peripheral phenylalkynyl ligand incorporation are all beneficial compositional changes. Examination of the synthesis intermediates has identified peripheral iodination as a further beneficial compositional change. Computational studies of linear and two-photon absorption suggest the use of the 3PE linker as optimum for π-delocalization and maximizing MPA merit.

In addition to record values of (2–4)PA, the present study has also afforded molecules exhibiting higher-order MPA. Rare examples of molecular 5PA have been found with the second- and third-generation metallodendrimers. Generation increase, installation of peripheral chlorido ligands, and incorporation of alkyl solubilizing groups have been identified as molecular modifications that improve 5PA. The second and third examples of molecular 6PA have been found with the third-generation metallodendrimers; these exhibit activity at much longer wavelengths than the only extant literature example. The *σ*_6_ and *σ*_6_/M parameters of the dendrimers increase on incorporation of alkyl substituents at the OPE group between the first- and second-generation branching points.

## Data availability

The data that support the findings of this study are available in the ESI† of this article.

## Author contributions

Conceptualization: M. G. H. Data curation: L. Z., M. M. Formal analysis: L. Z., M. M., T. S. Funding acquisition: M. G. H. Investigation: L. Z., M. M., T. S. Methodology: L. Z., M. M., R. K., M. G. H. Project administration: M. G. H. Resources: M. G. H. Software: L. Z., M. M., R. K. Supervision: M. G. H. Validation: L. Z., M. M., R. K. Visualization: L. Z., M. M. Writing – original draft: L. Z., M. M., M. G. H. Writing – review and editing: L. Z., M. M., R. K., M. G. H.

## Conflicts of interest

There are no conflicts to declare.

## Supplementary Material

SC-015-D4SC01127A-s001

## References

[cit1] He G. S., Tan L.-S., Zheng Q., Prasad P. N. (2008). Chem. Rev..

[cit2] Feng Z., Tang T., Wu T., Yu X., Zhang Y., Wang M., Zheng J., Ying Y., Chen S., Zhou J., Fan X., Zhang D., Li S., Zhang M., Qian J. (2021). Light: Sci. Appl..

[cit3] Liu C. J., Roy A., Simons A. A., Farinella D. M., Kara P. (2020). Sci. Rep..

[cit4] Albota M., Beljonne D., Bredas J. L., Ehrlich J., Fu J. Y., Heikal A. A., Hess S. E., Kogej T., Levin M. D., Marder S. R., McCord-Maughon D., Perry J. W., Rockel H., Rumi M., Subramaniam G., Webb W. W., Wu X. L., Xu C. (1998). Science.

[cit5] Joshi M. P., Swiatkiewicz J., Xu F., Prasad P. N., Reinhardt B. A., Kannan R. (1998). Opt. Lett..

[cit6] Reinhardt B. A., Brott L. L., Clarson S. J., Dillard A. G., Bhatt J. C., Kannan R., Yuan L., He G. S., Prasad P. N. (1998). Chem. Mater..

[cit7] Cho B. R., Son K. H., Lee S. H., Song Y. S., Lee Y. K., Jeon S. J., Choi J. H., Lee H., Cho M. H. (2001). J. Am. Chem. Soc..

[cit8] Xu L., Zhang J., Yin L., Long X., Zhang W., Zhang Q. (2020). J. Mater. Chem. C.

[cit9] Xu L., Lin W., Huang B., Zhang J., Long X., Zhang W., Zhang Q. (2021). J. Mater. Chem. C.

[cit10] Weishäupl S. J., Mayer D. C., Cui Y., Kumar P., Oberhofer H., Fischer R. A., Hauer J., Pöthig A. (2022). J. Mater. Chem. C.

[cit11] Chołuj M., Behera R., Petrusevich E. F., Bartkowiak W., Alam Md. M., Zaleśny R. (2022). J. Phys. Chem. A.

[cit12] Zhou F., Ji W. (2017). Laser Photonics Rev..

[cit13] Hernández F. E., Belfield K. D., Cohanoschi I., Balu M., Schafer K. J. (2004). Appl. Opt..

[cit14] Szeremeta J., Nyk M., Wawrzynczyk D., Samoc M. (2013). Nanoscale.

[cit15] Jiang Y., Li K. F., Gao K., Lin H., Tam H. L., Liu Y. Y., Shu Y., Wong K. L., Lai W. Y., Cheah K. W., Huang W. (2021). Angew. Chem., Int. Ed..

[cit16] Zheng Q., Zhu H., Chen S.-C., Tang C., Ma E., Chen X. (2013). Nat. Photonics.

[cit17] Wu W., Wang C., Li Q., Ye C., Qin J., Li Z. (2014). Sci. Rep..

[cit18] Tang R., Zhou S., Cheng Z., Yu G., Peng Q., Zeng H., Guo G., Li Q., Li Z. (2017). Chem. Sci..

[cit19] Wu W., Ye C., Qin J., Li Z. (2013). ACS Appl. Mater. Interfaces.

[cit20] Vestberg R., Westlund R., Eriksson A., Lopes C., Carlsson M., Eliasson B., Glimsdal E., Lindgren M., Malmström E. (2006). Macromolecules.

[cit21] Powell C. E., Morrall J. P., Ward S. A., Cifuentes M. P., Notaras E. G. A., Samoc M., Humphrey M. G. (2004). J. Am. Chem. Soc..

[cit22] Cifuentes M. P., Powell C. E., Morrall J. P., McDonagh A. M., Lucas N. T., Humphrey M. G., Samoc M., Houbrechts S., Asselberghs I., Clays K., Persoons A., Isoshima T. (2006). J. Am. Chem. Soc..

[cit23] Samoc M., Morrall J. P., Dalton G. T., Cifuentes M. P., Humphrey M. G. (2007). Angew. Chem., Int. Ed..

[cit24] Roberts R. L., Schwich T., Corkery T. C., Cifuentes M. P., Green K. A., Farmer J. D., Low P. J., Marder T. B., Samoc M., Humphrey M. G. (2009). Adv. Mater..

[cit25] Green K. A., Cifuentes M. P., Samoc M., Humphrey M. G. (2011). Coord. Chem. Rev..

[cit26] Simpson P. V., Watson L. A., Barlow A., Wang G., Cifuentes M. P., Humphrey M. G. (2016). Angew. Chem., Int. Ed..

[cit27] Zhang L., Morshedi M., Humphrey M. G. (2022). Angew. Chem., Int. Ed..

[cit28] Babgi B., Rigamonti L., Cifuentes M. P., Corkery T. C., Randles M. D., Schwich T., Petrie S., Stranger R., Teshome A., Asselberghs I., Clays K., Samoc M., Humphrey M. G. (2009). J. Am. Chem. Soc..

[cit29] Powell C. E., Hurst S. K., Morrall J. P., Roberts R. L., Cifuentes M. P., Samoc M., Humphrey M. G. (2007). Organometallics.

[cit30] Touchard D., Morice C., Cadierno V., Haquette P., Toupet L., Dixneuf P. H. (1994). J. Chem. Soc., Chem. Commun..

[cit31] West P. J., Cifuentes M. P., Schwich T., Randles M. D., Morrall J. P., Kulasekera E., Petrie S., Stranger R., Humphrey M. G. (2012). Inorg. Chem..

[cit32] Powell C. E., Cifuentes M. P., Morrall J. P. L., Stranger R., Humphrey M. G., Samoc M., Luther-Davies B., Heath G. A. (2003). J. Am. Chem. Soc..

[cit33] Babgi B. A., Kodikara M. S., Morshedi M., Wang H., Quintana C., Schwich T., Moxey G. J., Van Steerteghem N., Clays K., Stranger R., Cifuentes M. P., Humphrey M. G. (2018). ChemPlusChem.

[cit34] Zhang L., Morshedi M., Kodikara M., Humphrey M. G. (2022). Angew. Chem., Int. Ed..

[cit35] Adamo C., Barone V. (1999). J. Chem. Phys..

[cit36] Hehre W. J., Ditchfield R., Pople J. A. (1969). J. Chem. Phys..

[cit37] Grimme S., Ehrlich S., Goerigk L. (2011). J. Comput. Chem..

[cit38] Miertus S., Scrocco E., Tomasi J. (1981). Chem. Phys..

[cit39] Tomasi J., Mennucci B., Cammi R. (2005). Chem. Rev..

[cit40] Yanai T., Tew D., Handy N. (2004). Chem. Phys. Lett..

[cit41] Peach M. J. G., Helgaker T., Sazek P., Keal T. W., Lutnæs O. B., Tozer D. J., Handy N. C. (2006). Phys. Chem. Chem. Phys..

[cit42] Krishnan R., Binkley J. S., Seeger R., Pople J. A. (1980). J. Chem. Phys..

[cit43] Andrae D., Haeussermann U., Dolg M., Stoll H., Preuss H. (1990). Theor. Chem. Acc..

[cit44] Sheik-Bahae M., Hagan D. J., van Stryland E. W. (1990). Phys. Rev. Lett..

[cit45] Schwich T., Barlow A., Cifuentes M. P., Szeremeta J., Samoc M., Humphrey M. G. (2017). Chem.–Eur. J..

[cit46] Schwich T., Cifuentes M. P., Gugger P. A., Samoc M., Humphrey M. G. (2011). Adv. Mater..

[cit47] Drobizhev M., Karotki A., Kruk M., Rebane A. (2002). Chem. Phys. Lett..

[cit48] Moreno J. P., Kuzyk M. G. (2005). J. Chem. Phys..

[cit49] Kuzyk M. G., Pérez-Moreno J., Shafei S. (2013). Phys. Rep..

[cit50] Olsen J., Jørgensen P. (1985). J. Chem. Phys..

[cit51] Aidas K., Angeli C., Bak K. L., Bakken V., Bast R., Boman L., Christiansen O., Cimiraglia R., Coriani S., Dahle P., Dalskov E. K., Ekström U., Enevoldsen T., Eriksen J. J., Ettenhuber P., Fernández B., Ferrighi L., Fliegl H., Frediani L., Hald K., Halkier A., Hättig C., Heiberg H., Helgaker T., Hennum A. C., Hettema H., Hjertenæs E., Høst S., Høyvik I.-M., Iozzi M. F., Jansik B., Jensen H. J. Aa., Jonsson D., Jørgensen P., Kauczor J., Kirpekar S., Kjærgaard T., Klopper W., Knecht S., Kobayashi R., Koch H., Kongsted J., Krapp A., Kristensen A., Ligabue A., Lutnæs O. B., Melo J. I., Mikkelsen K. V., Myhre R. H., Neiss C., Nielsen C. B., Norman P., Olsen J., Olsen J. M. H., Osted A., Packer M. J., Pawlowski F., Pedersen T. B., Provasi P. F., Reine S., Rinkevicius Z., Ruden T. A., Ruud K., Rybkin V., Salek P., Samson C. C. M., Sánchez de Merás A., Saue T., Sauer S. P. A., Schimmelpfennig B., Sneskov K., Steindal A. H., Sylvester-Hvid K. O., Taylor P. R., Teale A. M., Tellgren E. I., Tew D. P., Thorvaldsen A. J., Thøgersen L., Vahtras O., Watson M. A., Wilson D. J. D., Ziolkowski M., Ågren H. (2014). WIREs Comput. Mol. Sci..

[cit52] Ågren H., Vahtras O., Koch H., Jørgensen P., Helgaker T. (1993). J. Chem. Phys..

[cit53] Frediani L., Ågren H., Ferrighi L., Ruud K. (2005). J. Chem. Phys..

[cit54] Andrae D., Haeussermann U., Dolg M., Stoll H., Preuss H. (1990). Theor. Chem. Acc..

[cit55] Krishnan R., Binkley J. S., Seeger R., Pople J. A. (1980). J. Chem. Phys..

[cit56] Kamada K., Ohta K., Iwase Y., Kondo K. (2003). Chem. Phys. Lett..

