# Supplementary material for: Exceptional three- to six-photon absorption at organometallic dendrimers
Source: Chem Sci. 2024 May 14;15(23):8731–9. doi: 10.1039/d4sc01127a (PMC11168170; doi:10.1039/d4sc01127a)
Supplement: SC-015-D4SC01127A-s001 [file SC-015-D4SC01127A-s001.pdf]

Electronic Supplementary Material (ESI) for Chemical Science.
This journal is © The Royal Society of Chemistry 2024

Supporting Information for

# Exceptional Three- to Six-Photon Absorption at Organometallic Dendrimers

Ling Zhang,[a] Mahbod Morshedi,[a] Torsten Schwich,[a] Rika Kobayashi,[b] and Mark G. Humphrey*[a]

[a] Research School of Chemistry, Australian National University, Canberra, ACT 2601, Australia

[b] National Computational Infrastructure, Australian National University, Canberra, ACT 2601, Australia

* To whom correspondence should be addressed. E: Mark.Humphrey@anu.edu.au.

# Contents

Extant multi-photon absorption data……………………………………………………………..S3

Synthesis schemes…...……………………….……………………………………………...S13

General conditions and reagents………..………………………………………………..…..S20

Instrumentation……………………………………………………………..……......................S22

Syntheses and characterization…………………………………………………………………S25

NMR spectra……………….…………………………………………..…………………………S40

DOSY, SEC, TEM, and MS studies……………………………………………………….....S84

UV-vis-NIR studies………………………………………………………………......................S92

Computational studies – general comments……………………………………….....................S95

Computational studies – 1PA calculations……………………………………………………S113

Z-scan studies……………………………………………………………….............................S125

Computational studies – 2PA calculations……………………………………………….........S146

References………………………………………………………..……………………...S149

# Extant multi-photon absorption data

**Table S1.** Three-photon, four-photon, five-photon, and six-photon absorption data for coordination complexes, organic molecules, MOFs, perovskites, and other inorganic materials reported 2022-present (for data pre-2016, see ref [1], and for 2016-2021, see ref [2]).

| **Three-photon absorption materials** | **3PA quantities ($\lambda_{ex}$/nm)** | **Conditions** | **Ref.** |
|---|---|---|---|
| **Coordination complexes** | | | |
| 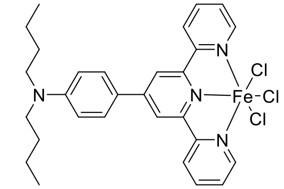 | $2780 \times 10^{-80}$ cm$^6$ s$^2$ photon$^{-2}$ (1400 nm) | 140 fs, 1 kHz, Z-scan | 3 |
| 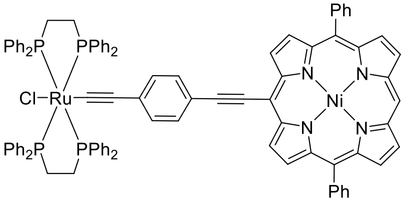 | $14000 \times 10^{-80}$ cm$^6$ s$^2$ photon$^{-2}$ (1200 nm) | 130 fs, 1 kHz, Z-scan | 4 |
| 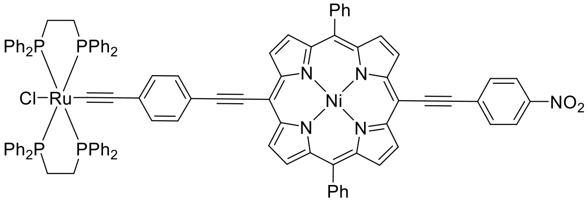 | $37600 \times 10^{-80}$ cm$^6$ s$^2$ photon$^{-2}$ (1300 nm) | 130 fs, 1 kHz, Z-scan | 4 |
| 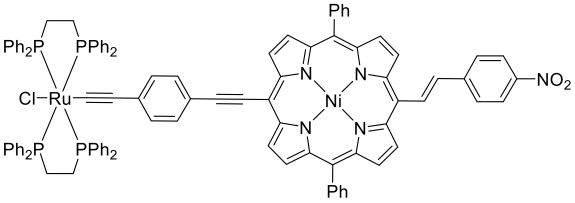 | $18200 \times 10^{-80}$ cm$^6$ s$^2$ photon$^{-2}$ (1200 nm) | 130 fs, 1 kHz, Z-scan | 4 |
| 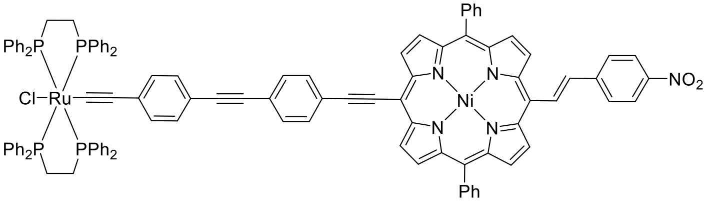 | $4000 \times 10^{-80}$ cm$^6$ s$^2$ photon$^{-2}$ (1250 nm) | 130 fs, 1 kHz, Z-scan | 4 |

| | | | |
|---|---|---|---|
| 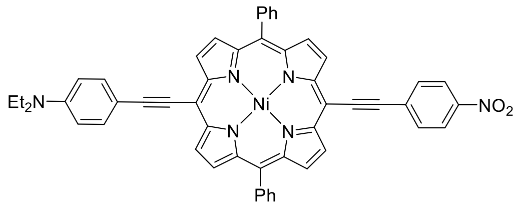 | 1600 × $10^{-80}$ $cm^6$ $s^2$ $photon^{-2}$ (1250 nm) | 130 fs, 1 kHz, Z-scan | 4 |
| 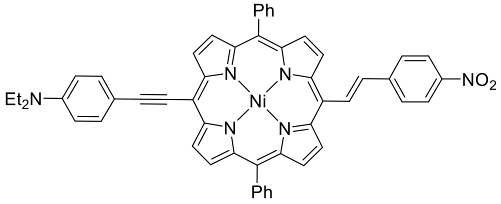 | 560 × $10^{-80}$ $cm^6$ $s^2$ $photon^{-2}$ (1250 nm) | 130 fs, 1 kHz, Z-scan | 4 |
| 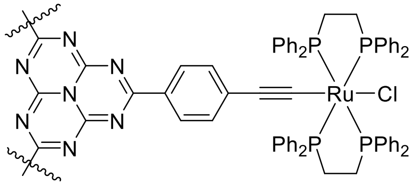 | 1640 × $10^{-80}$ $cm^6$ $s^2$ $photon^{-2}$ (1600 nm) | 130 fs, 1 kHz, Z-scan | 5 |
| 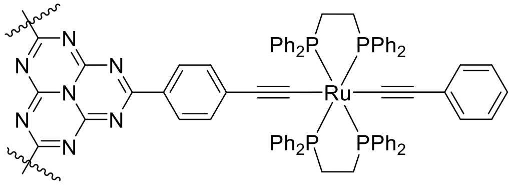 | 1150 × $10^{-80}$ $cm^6$ $s^2$ $photon^{-2}$ (1600 nm) | 130 fs, 1 kHz, Z-scan | 5 |
| 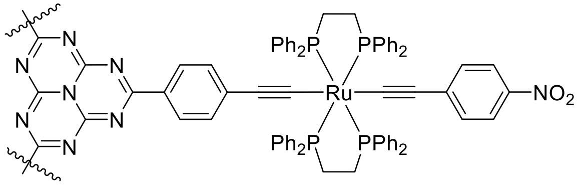 | 1250 × $10^{-80}$ $cm^6$ $s^2$ $photon^{-2}$ (1750 nm) | 130 fs, 1 kHz, Z-scan | 5 |
| 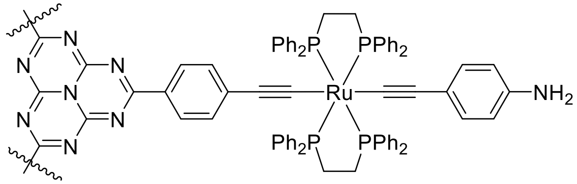 | 2030 × $10^{-80}$ $cm^6$ $s^2$ $photon^{-2}$ (1730 nm) | 130 fs, 1 kHz, Z-scan | 5 |

**Organic molecules**

| | | | |
|---|---|---|---|
| 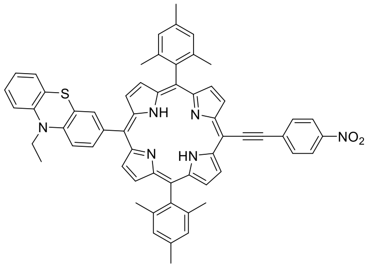 | (1000 to 34000) × $10^{-6}$ $cm^3$ $GW^{-2}$ (1100-1450 nm) | 120 fs,1 kHz, Z-scan | 6 |
| (*E*)-2-(Benzo[*d*]thiazol-2-yl)-3-(7-(diphenylamino)-9-ethyl-9H-carbazol-2-yl)acrylonitrile | 157 × $10^{-80}$ $cm^6$ $s^2$ $photon^{-2}$ (1550 nm) | (n/a) fs, MPEF | 7 |
| 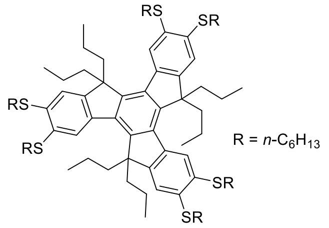 | 26.4 × $10^{-90}$ $cm^6$ $s^2$ $photon^{-2}$ (800 nm) | 100 fs, 10 Hz, Z-scan | 8 |
| 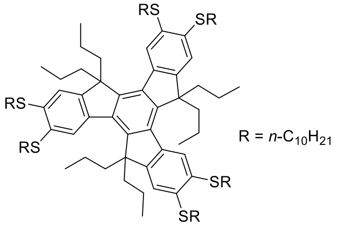 | 77.0 × $10^{-90}$ $cm^6$ $s^2$ $photon^{-2}$ (800 nm) | 100 fs, 10 Hz, Z-scan | 8 |
| 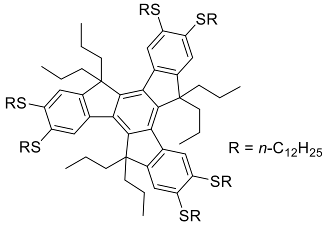 | 136 × $10^{-90}$ $cm^6$ $s^2$ $photon^{-2}$ (800 nm) | 100 fs, 10 Hz, Z-scan | 8 |
| 2-(4-Chlorophenyl)-3-methyl-4-(4-methylphenyl)-1,3-thiazolium-5-thiolate | (52 ± 5) × $10^{-80}$ $cm^6$ $s^2$ $J^{-2}$ (800 nm) | 100 fs, 1 kHz, Z-scan | 9 |

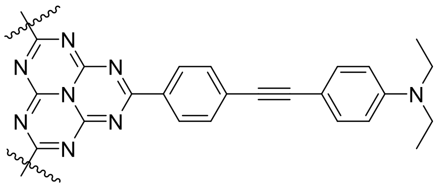

| | | | |
|---|---|---|---|
| | $120 \times 10^{-80}$ cm$^6$ s$^2$ photon$^{-2}$ (1650 nm) | 130 fs, 1 kHz, Z-scan | 5 |
| (*E*)-2-(Benzo[*d*]thiazol-2-yl)-3-(7-(diphenylamino)-9-ethyl-9H-carbazol-2-yl)acrylonitrile (DCBT) mixed with Fluronic F127, DCBT dots | $561 \times 10^{-80}$ cm$^6$ s$^2$ photon$^{-2}$ (1550 nm) | (n/a) fs, 3PEF | 7 |

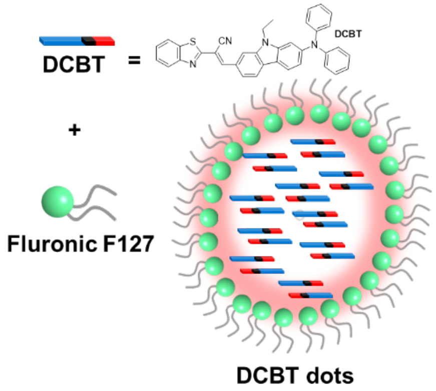


**MOFs**

| | | | |
|---|---|---|---|
| Zr-based 4,4’-(thiazolo[5,4-d]thiazole-2,5-diyl)dibenzoic acid ligated UiO-type MOF | $1.11 \times 10^{-80}$ cm$^6$ s$^2$ photon$^{-2}$ (1250 nm) | 120 fs, 1 kHz, MPEF | 10 |
| Zr-based 4,4’-(thiazolo[5,4-d]thiazole-2,5-diyl)dibenzoic acid ligated UiO-type MOF with methylation | $1.16 \times 10^{-80}$ cm$^6$ s$^2$ photon$^{-2}$ (1250 nm) | 120 fs, 1 kHz, MPEF | 10 |
| $Zn_2$(TCPE), a zinc-AIEgen MOF | $(1.2\text{-}6.5) \times 10^{-63}$ cm$^6$ s$^2$ photon$^{-2}$ (1200-1300 nm) | ca. 200 fs, 1 MHz, MPEF | 11 |

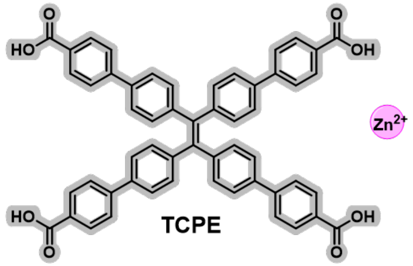

| | | | |
|---|---|---|---|
| $Zn_2(TCPE)(L_1)$, a zinc-AIEgen MOF<br>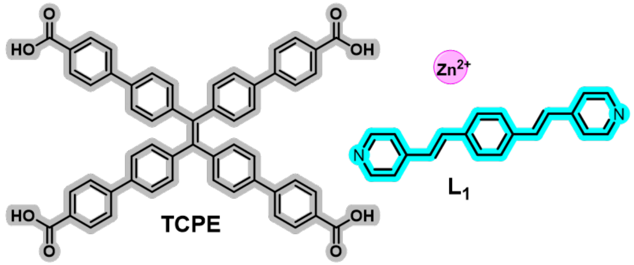<br> | (300000-30000000) × $10^{-80}$ cm$^6$ s$^2$ photon$^{-2}$ (1200-1300 nm) | ca. 200 fs, 1 MHz, MPEF | 11 |
| $Zn_2(TCPE)(L_2)$, a zinc-AIEgen MOF<br>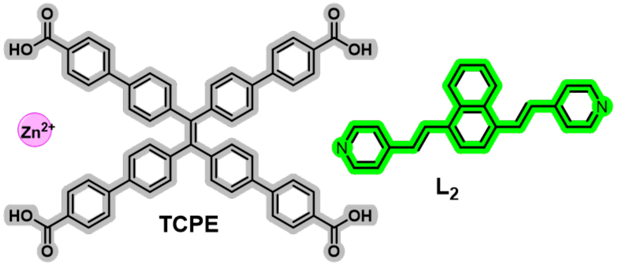<br> | (0.154-2.9) × $10^{-70}$ cm$^6$ s$^2$ photon$^{-2}$ (1200-1300 nm) | ca. 200 fs, 1 MHz, MPEF | 11 |
| $Zn_2(TCPE)(L_3)$, a zinc-AIEgen MOF<br>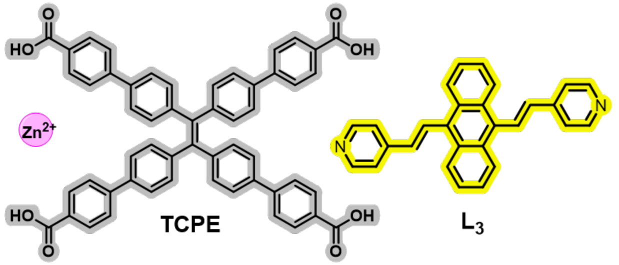<br> | (0.00495-0.0785) × $10^{-70}$ cm$^6$ s$^2$ photon$^{-2}$ (1200-1300 nm) | ca. 200 fs, 1 MHz, MPEF | 11 |
| $Zn_2(TCPE)(L_4)$, a zinc-AIEgen MOF | (3000-1000000) × $10^{-80}$ cm$^6$ s$^2$ photon$^{-2}$ (1200-1300 nm) | ca. 200 fs, 1 MHz, MPEF | 11 |

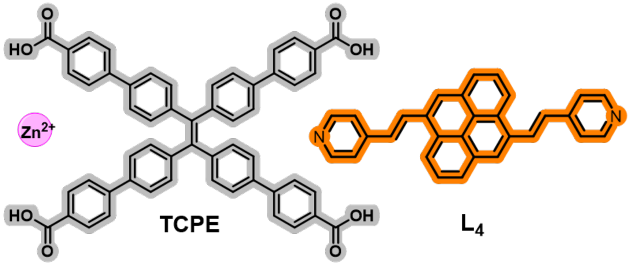


| | | | |
|---|---|---|---|
| Zn(SCN)$_2$L$_2$@PCN-56, a zinc MOF composite | 9.43 × $10^{-80}$ cm$^6$ s$^2$ photon$^{-2}$ (1200 nm) | 120 fs, 1 kHz, MPEF | 12 |
| [Ir(PBTF)$_2$Cl]$_2$-modified UiO(bpdc) with orotic acid coating | 2.6 × $10^{-80}$ cm$^6$ s$^2$ photon$^{-2}$ (1250 nm) | 120 fs, 1 kHz, MPEF | 13 |
| **Perovskites** | | | |
| FA$_{0.8}$MA$_{0.2}$PbI$_3$ | 261 ± 50 cm$^3$ GW$^{-2}$ (1300 nm) | 30 ps, 50 Hz, MPEF | 14 |
| FAPbBr$_3$/CsPbBr$_3$ core-shell nanocrystals | 5500 × $10^{-80}$ cm$^6$ s$^2$ photon$^{-2}$ nm$^{-3}$ (1300 nm) | 100 fs, 1 kHz, MPEF and Z-scan | 15 |
| FAPbBr$_3$ nanocrystals | 630 × $10^{-80}$ cm$^6$ s$^2$ photon$^{-2}$ nm$^{-3}$ (1300 nm) | 100 fs, 1 kHz, MPEF and Z-scan | 15 |
| MAPbBr$_3$ film on ITO glass | 7.35 × $10^4$ cm$^3$ GW$^{-2}$ (n/a) | OA Z-scan | 16 |
| MAPbBr$_3$ single crystals | (1.9-8.2) × $10^{-7}$ cm$^3$ GW$^{-2}$ (1200-1500 nm) | 35 fs, 1 kHz, Z-scan | 17 |
| MAPbCl$_3$ single crystals | 1.3 × $10^{-7}$ cm$^3$ GW$^{-2}$ (1200 nm) | 35 fs, 1 kHz, Z-scan | 17 |
| CsPbBr$_3$ microparticles | 41000 ± 2000 × $10^{-80}$ cm$^6$ s$^2$ photon$^{-2}$ (1300 nm) | 216 fs, 60 kHz, MPEF | 18 |
| CsPbBr$_3$ quantum dots in ZIF-8 | 11000000 ± 2000000 × $10^{-80}$ cm$^6$ s$^2$ photon$^{-2}$ (1300 nm) | 216 fs, 60 kHz, MPEF | 18 |
| Cs$_2$TeCl$_6$ | (5.42-143.58) × $10^{-80}$ cm$^6$ s$^2$ (800-1150 nm) | 35 fs, 1 kHz, MPAPS | 19 |
| **Other inorganic molecules** | | | |
| NiO NPs calcined at 400 °C | 328 × $10^{-80}$ cm$^6$ s$^2$ photon$^{-2}$ (1064 nm) | 9 ns, 10 Hz, Z-scan | 20 |
| Arginine-stabilized 6-aza-2-thiothymine Au nanoclusters, Arg/ATT-Au NCs | 150 × $10^{-80}$ cm$^6$ s$^2$ photon$^{-2}$ (1400 nm) | (n/a) fs, MPAPS | 21 |
| InP/ZnSeS/ZnS quantum dots | 128 × $10^{-80}$ cm$^6$ s$^2$ photon$^{-2}$ (1240 nm) | 100 fs, 80 MHz, MEPL | 22 |
| InP/ZnS quantum dots | 42 × $10^{-80}$ cm$^6$ s$^2$ photon$^{-2}$ (1260 nm) | 100 fs, 80 MHz, MEPL | 22 |
| Indium-tin oxide quantum dots thin film | 0.142 cm$^3$ GW$^{-2}$ (950 nm) | 150 fs, 500 kHz, Z-scan | 23 |
| 2D CdSe nanoplatelets | 4.0 × $10^{-2}$ cm$^3$ GW$^{-2}$ (800 nm) | 120 fs, 1 kHz, Z-scan | 24 |
| 2D CdSe/CdS core-shell nanoplatelets | 0.9 × $10^{-2}$ cm$^3$ GW$^{-2}$ (800 nm) | 120 fs, 1 kHz, Z-scan and 185 fs, 76 MHz, OKG | 24 |

| Four-photon absorption materials | 4PA quantities ($\lambda_{ex}$/nm) | Conditions | |
|---|---|---|---|
| **Coordination complexes** | | | |
| 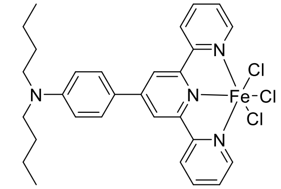 | $6.87 \times 10^{-74}$ cm$^8$ s$^3$ photon$^{-3}$ (2100 nm) | 140 fs, 1 kHz, Z-scan | 3 |
| 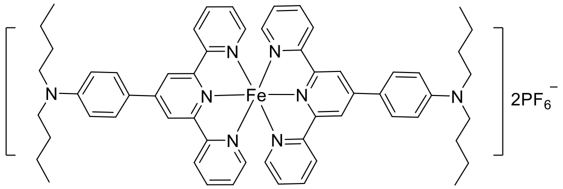 | $3.26 \times 10^{-73}$ cm$^8$ s$^3$ photon$^{-3}$ (2200 nm) | 120 fs, 1 kHz, Z-scan | 3 |
| 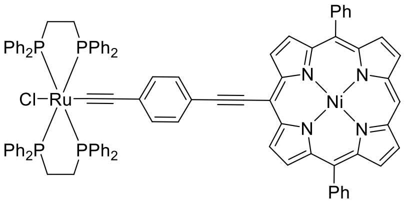 | $860 \times 10^{-110}$ cm$^8$ s$^3$ photon$^{-3}$ (1600 nm) | 130 fs, 1 kHz, Z-scan | 4 |
| 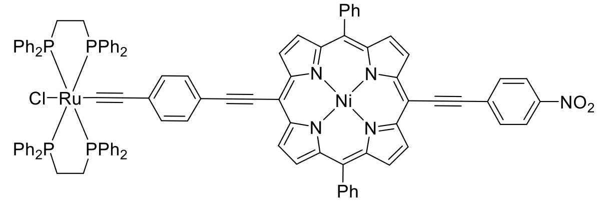 | $2030 \times 10^{-110}$ cm$^8$ s$^3$ photon$^{-3}$ (1700 nm) | 130 fs, 1 kHz, Z-scan | 4 |
| 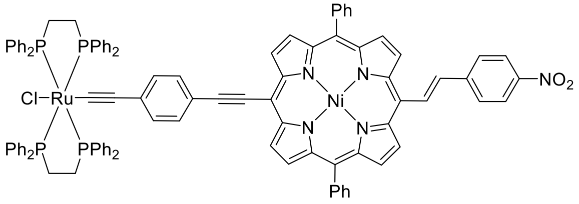 | $2130 \times 10^{-110}$ cm$^8$ s$^3$ photon$^{-3}$ (1700 nm) | 130 fs, 1 kHz, Z-scan | 4 |
| 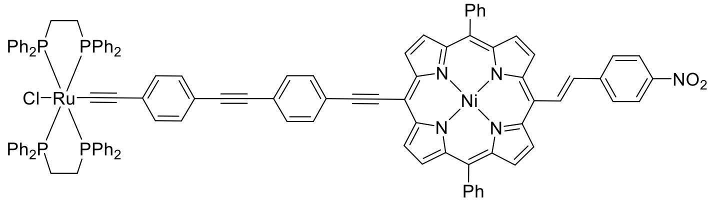 | $110 \times 10^{-110}$ cm$^8$ s$^3$ photon$^{-3}$ (1600 nm) | 130 fs, 1 kHz, Z-scan | 4 |

| | | | |
|---|---|---|---|
| 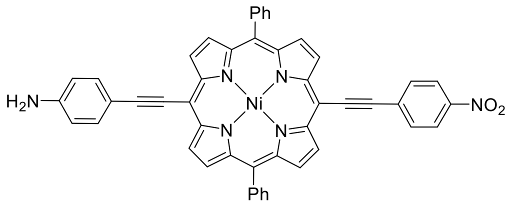 | $270 \times 10^{-110}$ $cm^8$ $s^3$ $photon^{-3}$ (1650 nm) | 130 fs, 1 kHz, Z-scan | 4 |
| 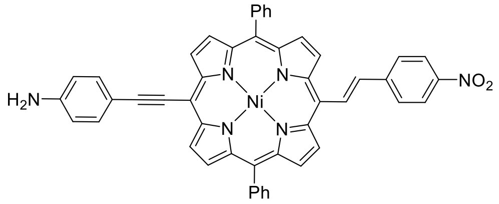 | $350 \times 10^{-110}$ $cm^8$ $s^3$ $photon^{-3}$ (1600 nm) | 130 fs, 1 kHz, Z-scan | 4 |
| 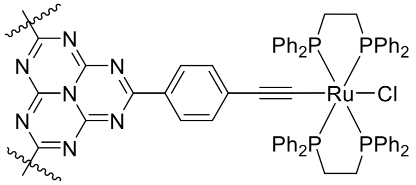 | $110 \times 10^{-110}$ $cm^8$ $s^3$ $photon^{-3}$ (2300 nm) | 130 fs, 1 kHz, Z-scan | 5 |
| 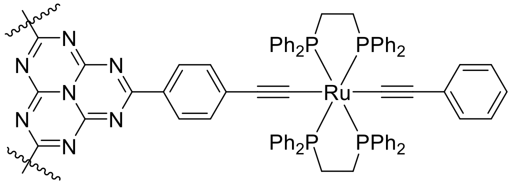 | $100 \times 10^{-110}$ $cm^8$ $s^3$ $photon^{-3}$ (2260 nm) | 130 fs, 1 kHz, Z-scan | 5 |
| 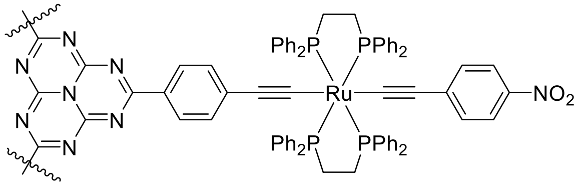 | $160 \times 10^{-110}$ $cm^8$ $s^3$ $photon^{-3}$ (2260 nm) | 130 fs, 1 kHz, Z-scan | 5 |
| 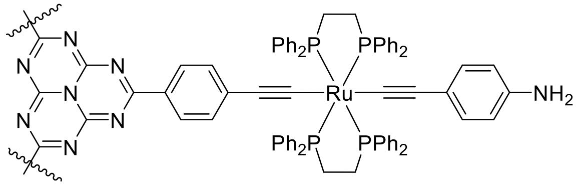 | $210 \times 10^{-110}$ $cm^8$ $s^3$ $photon^{-3}$ (2100 nm) | 130 fs, 1 kHz, Z-scan | 5 |

| | | | |
|---|---|---|---|
| **Organic molecules** | | | |
| 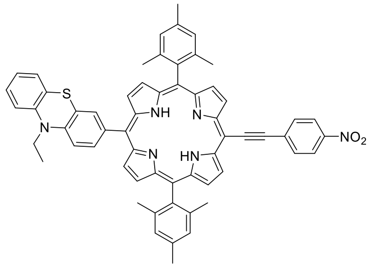 | $(1\text{-}2.7) \times 10^{-3}$ $cm^5$ $GW^{-3}$ (1500-1600 nm) | 120 fs, 1 kHz, Z-scan | 6 |
| **MOFs** | | | |
| Zr-based 4,4'-(thiazolo[5,4-d]thiazole-2,5-diyl)dibenzoic acid ligated UiO-type MOF | $4.41 \times 10^{-80}$ $cm^8$ $s^3$ $photon^{-3}$ (1450 nm) | 120 fs, 1 kHz, MPEF | 9 |
| Zr-based 4,4'-(thiazolo[5,4-d]thiazole-2,5-diyl)dibenzoic acid ligated UiO-type MOF with methylation | $5.27 \times 10^{-80}$ $cm^8$ $s^3$ $photon^{-3}$ (1550 nm) | 120 fs, 1 kHz, MPEF | 9 |
| $Zn(SCN)_2L_2$@PCN-56, a zinc MOF composite | $7.48 \times 10^{-80}$ $cm^8$ $s^3$ $photon^{-3}$ (1550 nm) | 120 fs, 1 kHz, MPEF | 12 |
| $[Ir(PBTF)_2Cl]_2$ modified UiO(bpdc) with orotic acid coating, MIrA | $26.7 \times 10^{-80}$ $cm^8$ $s^3$ $photon^{-3}$ (1450 nm) | 120 fs, 1 kHz, MPEF | 13 |
| **Perovskites** | | | |
| $MAPbCl_3$ single crystals | $5.1 \times 10^{-10}$ $cm^5$ $GW^{-3}$ (1500 nm) | 35 fs, 1 kHz, Z-scan | 17 |
| $MAPbBr_3$ single crystals | $1.8 \times 10^{-8}$ $cm^5$ $GW^{-3}$ (2100 nm) | 35 fs, 1 kHz, Z-scan | 17 |
| $CsPbBr_3$ microparticles | $15000 \pm 1000 \times 10^{-110}$ $cm^8$ $s^3$ $photon^{-3}$ (1800 nm) | 216 fs, 60 kHz, MPEF | 18 |
| $CsPbBr_3$ quantum dots in ZIF-8 | $7500000 \pm 1600000 \times 10^{-110}$ $cm^8$ $s^3$ $photon^{-3}$ (1800 nm) | 216 fs, 60 kHz, MPEF | 18 |
| $Cs_2TeCl_6$ | $6.51 \times 10^{-110}$ $cm^8$ $s^3$ $photon^{-3}$ (1560 nm) | 35 fs, 1 kHz, MPAPS | 19 |
| **Other inorganic molecules** | | | |
| Arginine-stabilized 6-aza-2-thiothymine Au nanoclusters, Arg/ATT-Au NCs | $550 \times 10^{-110}$ $cm^8$ $s^3$ $photon^{-3}$ (1700 nm) | (n/a) fs, MPAPS | 21 |
| **Five-photon absorption materials** | **5PA quantities ($\lambda_{ex}$/nm)** | **Conditions** | |
| **Perovskites** | | | |
| $FAPbBr_3/CsPbBr_3$ core-shell nanocrystals | $86.4 \times 10^{-140}$ $cm^{10}$ $s^4$ $photon^{-4}$ $nm^{-3}$ (2300 nm) | 100 fs, 1 kHz, MPEF and Z-scan | 15 |
| $FAPbBr_3$ nanocrystals | $1280 \times 10^{-110}$ $cm^8$ $s^3$ $photon^{-3}$ $nm^{-3}$ (2300 nm) | 100 fs, 1 kHz, MPEF and Z-scan | 15 |

| | | | |
|---|---|---|---|
| $MAPbBr_3$ single crystals | $8.4 \times 10^{-13}$ $cm^7$ $GW^{-4}$ (2400 nm) | 35 fs, 1 kHz, Z-scan | 17 |
| $MAPbCl_3$ single crystals | $8.7 \times 10^{-11}$ $cm^7$ $GW^{-4}$ (2100 nm) | 35 fs, 1 kHz, Z-scan | 17 |
| $CsPbBr_3$ microparticles | $80 \pm 6 \times 10^{-140}$ $cm^{10}$ $s^4$ $photon^{-4}$ (2200 nm) | 216 fs, 60 kHz, MPEF | 18 |
| $CsPbBr_3$ quantum dots in ZIF-8 | $6.2 \pm 1.3 \times 10^{-104}$ $cm^8$ $s^3$ $photon^{-3}$ (2200 nm) | 216 fs, 60 kHz, MPEF | 18 |
| $Cs_2TeCl_6$ | $0.02425 \times 10^{-140}$ $cm^{10}$ $s^4$ $photon^{-4}$ (1640 nm) | 35 fs, 1 kHz, MPAPS | 19 |
| **Six-photon absorption materials** | **6PA quantities ($\lambda_{ex}$/nm)** | **Conditions** | |
| **Perovskites** | | | |
| $Cs_2TeCl_6$ | $0.000187 \times 10^{-170}$ $cm^{12}$ $s^5$ $photon^{-5}$ (1980 nm) | 35 fs, 1 kHz, MPAPS | 20 |

AIEgen: aggregation-induced emission luminogen. bpdc: 2,2`-bipyridine-5,5`-dicarboxylic acid. D-A: donor-acceptor. DCBT: (*E*)-2-(benzo[*d*]thiazol-2-yl)-3-(7-(diphenylamino)-9-ethyl-9*H*-carbazol-2-yl)acrylonitrile. FA: formamidinium. L: (*E*)-*N,N*-diethyl-4-(2-(pyridin-4-yl)vinyl)aniline. MA: methylammonium. MIC: mesoionic compound. MOF: metal-organic framework. MPAPS: multiphoton absorption photoluminescence saturation method. MPEF: multi-photon excited fluorescence. NPs: nanoparticles. OA: open-aperture. OKG: optical Kerr gate. PBTF: 2-(3,4-difluorophenyl)benzo[*d*]thiazole. PCN-56: $Zr_3O_2(OH)_2(TPDC\text{-}2Me)_3$. TPDC-2Me: 2`,5`-dimethyl-[1,1`:4`,1``-terphenyl]-4,4``-dicarboxylic acid. UiO: $Zr_6O_4(OH)_4$. ZIF: zeolitic imidazolylate framework.

## Synthesis schemes

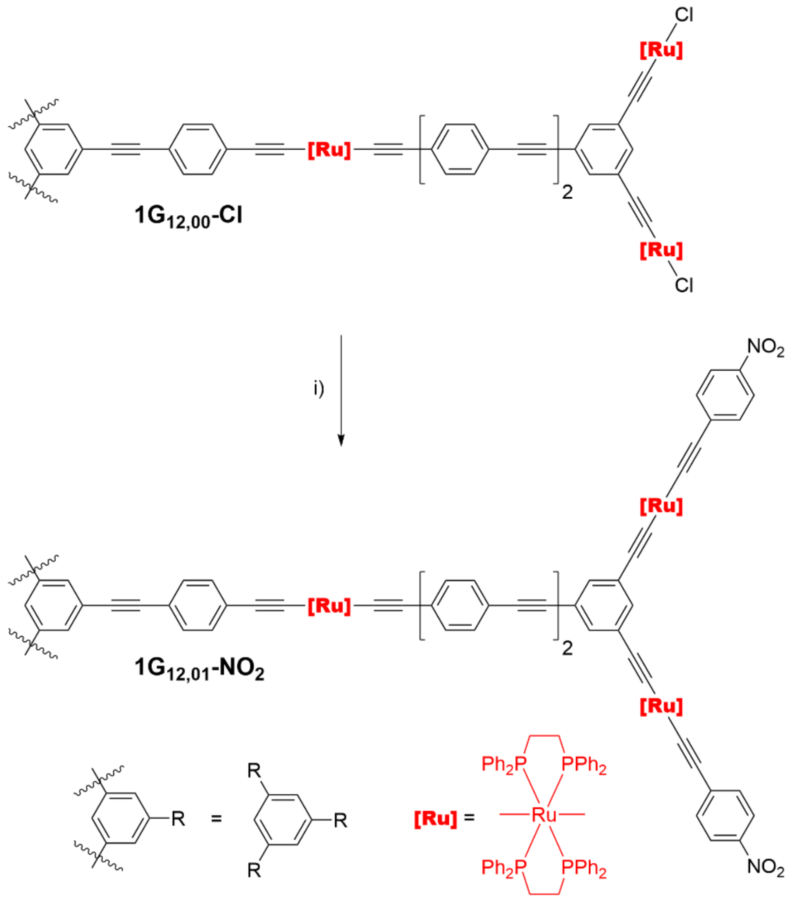


**Scheme S1.** Synthesis of **$1G_{12,01}$-$NO_2$**. i) 1,4-HC≡$CC_6H_4NO_2$, $NaPF_6$, deoxygenated $CH_2Cl_2/NEt_3$, room temperature, 16 h, 82%.

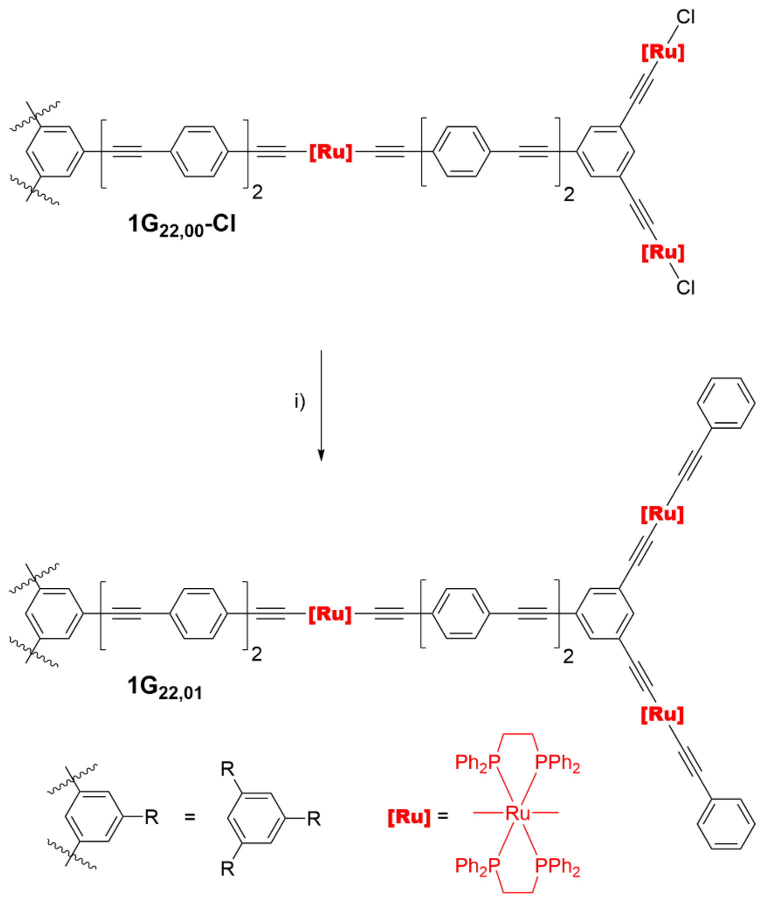


**Scheme S2.** Synthesis of **$1G_{22,01}$**. i) Ethynylbenzene, $NaPF_6$, deoxygenated $CH_2Cl_2/NEt_3$, room temperature, 3 days, 82%.

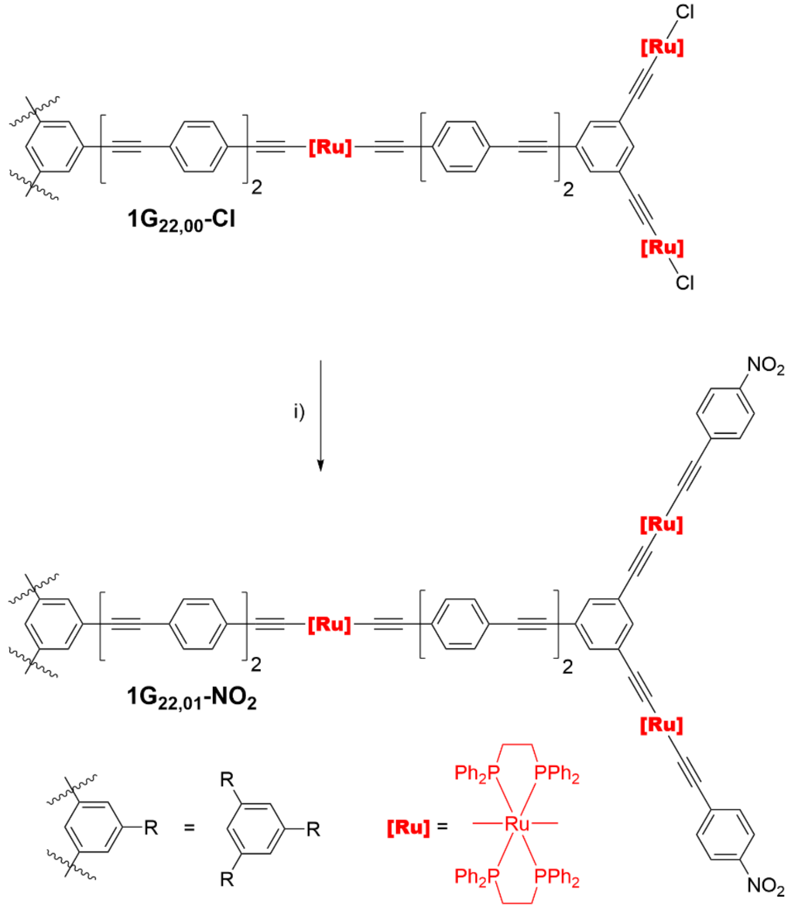


**Scheme S3.** Synthesis of **$1G_{22,01}$-$NO_2$**. i) 1,4-HC≡$CC_6H_4NO_2$, $NaPF_6$, deoxygenated $CH_2Cl_2/NEt_3$, room temperature, 16 h, 73%.

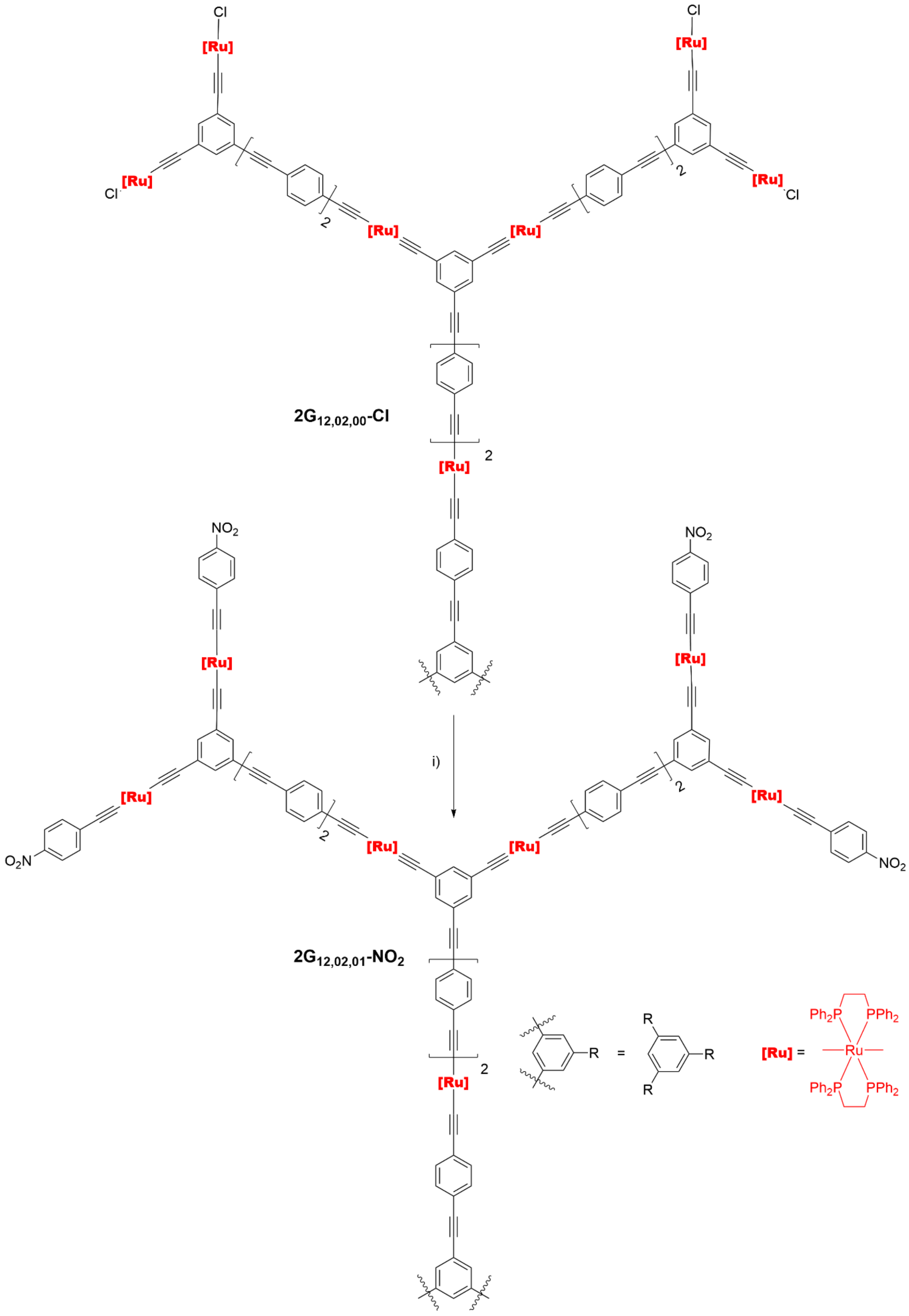


**Scheme S4.** Synthesis of **$2G_{12,02,01}$-$NO_2$**. i) 1,4-HC≡$CC_6H_4NO_2$, $NaPF_6$, deoxygenated $CH_2Cl_2/NEt_3$, room temperature, 16 h, 89%.

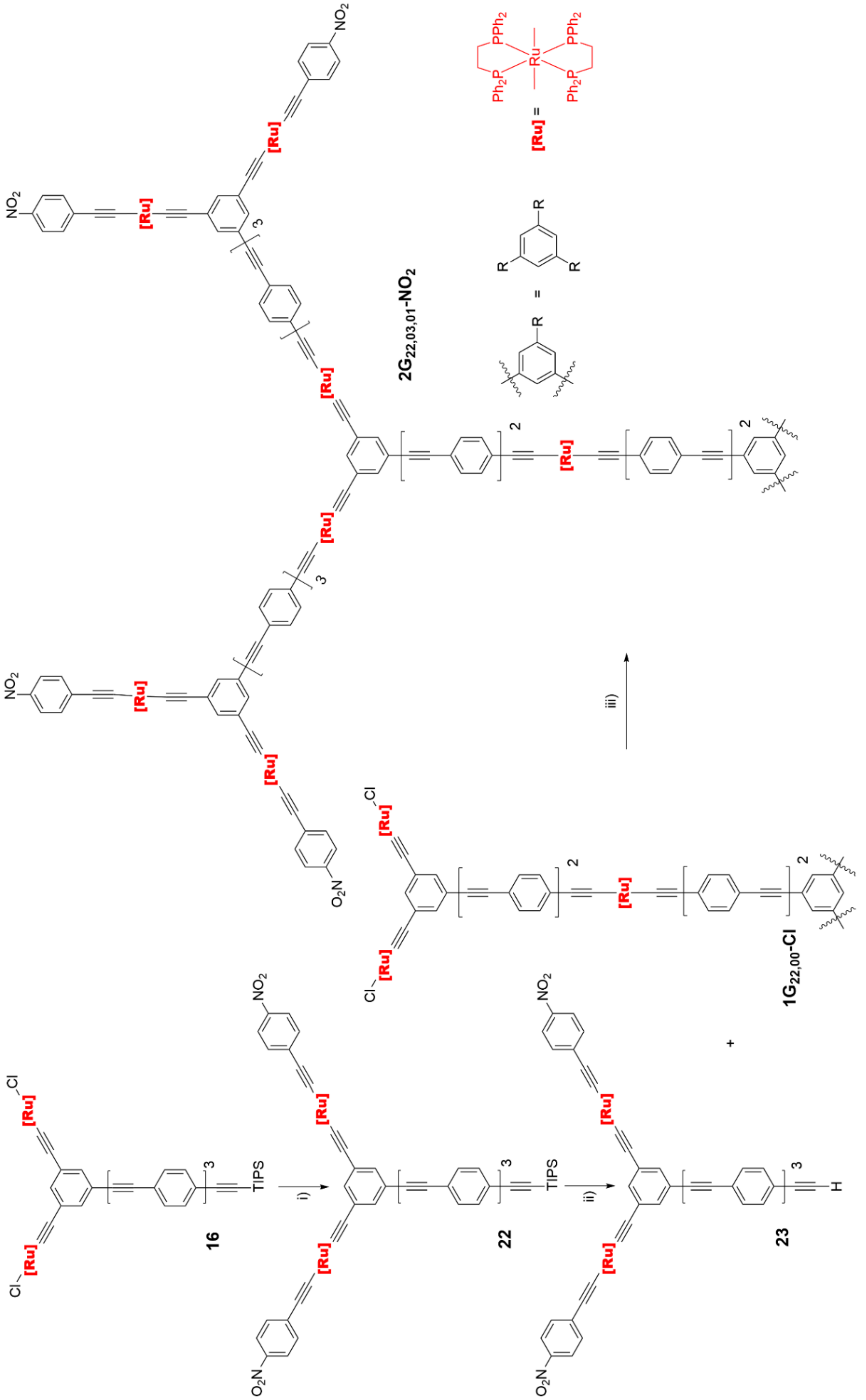


**Scheme S5.** Synthesis of **$2G_{22,03,01}$-$NO_2$**. i) 1,4-HC≡$CC_6H_4NO_2$, $NaPF_6$, deoxygenated $CH_2Cl_2$, $NEt_3$, room temperature, 16 h, 82%; ii) deoxygenated $CH_2Cl_2$, $NEt_3$, TBAF, room temperature, 2 h, 95%; iii) $NaPF_6$, deoxygenated $CH_2Cl_2$, $NEt_3$, room temperature, 3 days, 87%.

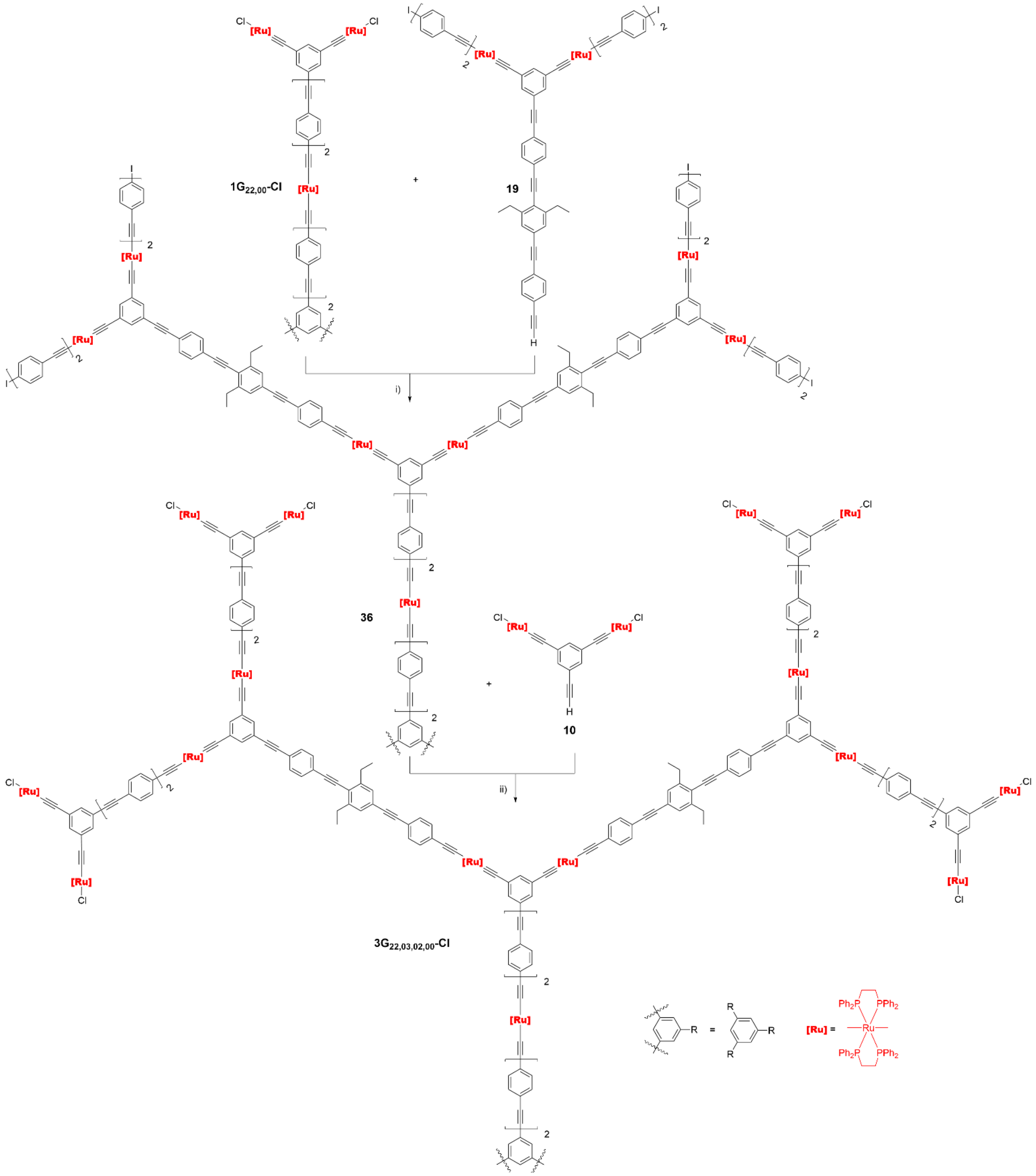


**Scheme S6.** Synthesis of **$3G_{22,03,02,00}$-Cl**. i) $NaPF_6$, deoxygenated $CH_2Cl_2$, $NEt_3$, reflux, 24 h, 91%; ii) $Pd(PPh_3)_4$, $[Cu(NCMe)_4]PF_6$, deoxygenated $CH_2Cl_2/NEt_3$, room temperature, 3 days, 50%.

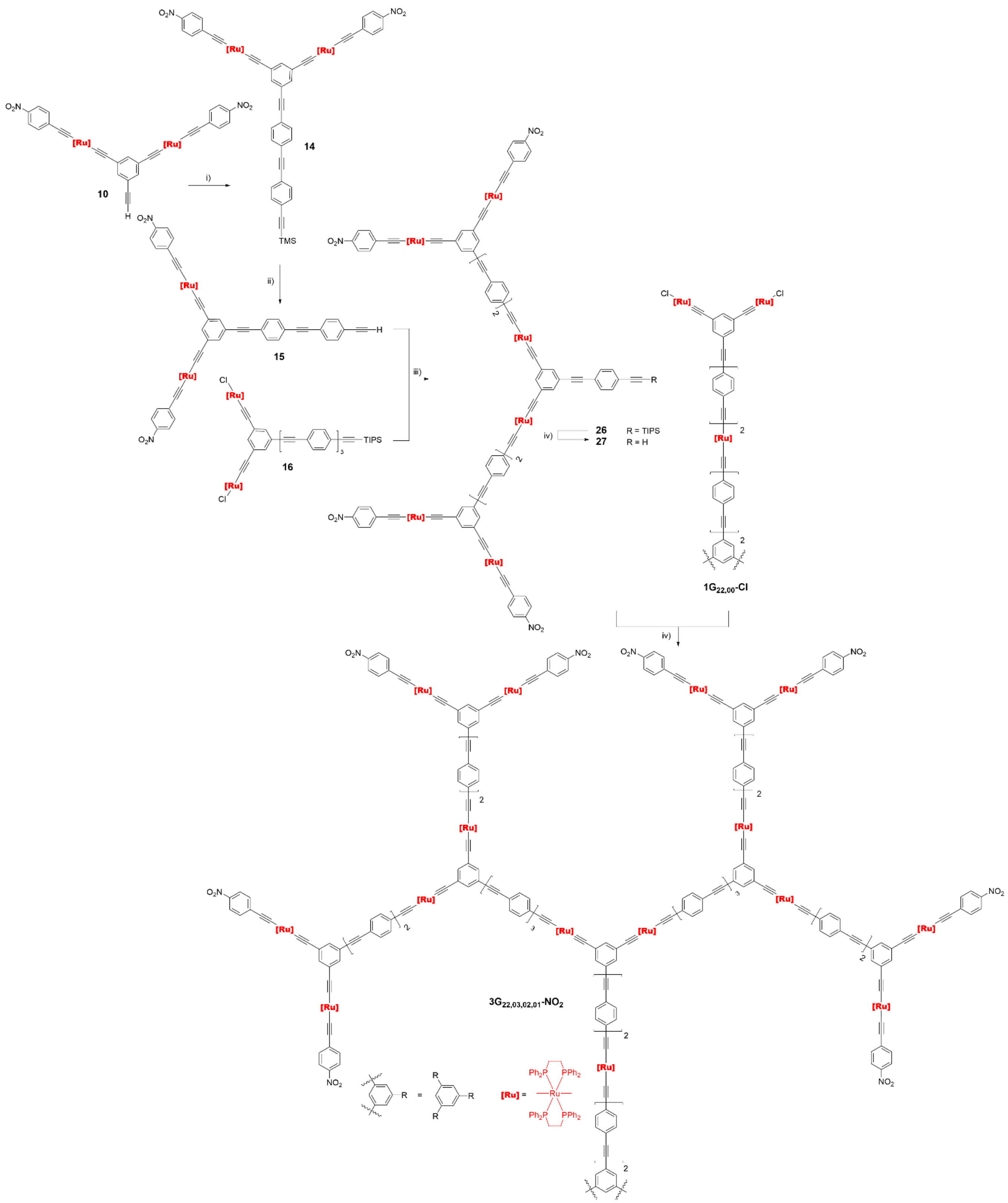


**Scheme S7.** Synthesis of **$3G_{22,03,02,01}$-$NO_2$**. i) 1,4-$Me_3SiC{\equiv}CC_6H_4C{\equiv}C$-1,4-$C_6H_4I$, $Pd(PPh_3)_4$, CuI, deoxygenated $CH_2Cl_2$/$NEt_3$, room temperature, 3 days, 80%; ii) $NaPF_6$, deoxygenated $CH_2Cl_2$, $NEt_3$, room temperature, 2 h, 88%; iii) deoxygenated $CH_2Cl_2$, $NEt_3$, TBAF, room temperature, 16 h, 91%; iv) $NaPF_6$, deoxygenated $CH_2Cl_2$, $NEt_3$, room temperature, 16 h, 84%.

## General conditions and reagents

Reactions were performed under a nitrogen atmosphere using standard Schlenk techniques with flame-dried glassware and Teflon-coated magnetic stirring bars. All work-up and purification procedures were carried out with reagent grade solvent in air. The following reagents, catalysts and solvents were commercially available, purchased from common chemical suppliers and used as received unless mentioned otherwise: tetra-*n*-butylammonium fluoride (TBAF, as a 1.0 M solution in THF), ethynylbenzene, $NaPF_6$, CuI, $NEt_3$, *n*-pentane, methanol, diethyl ether, Celite. The term "petrol" refers to a fraction of petroleum with a boiling range of 60-80 °C. Reagent grade solvent dichloromethane ($CH_2Cl_2$) (Merck) was dried by distilling over calcium hydride and stored under a nitrogen atmosphere. Column chromatography was performed using activated basic alumina (Sigma-Aldrich).

Commercially available $Pd(PPh_3)_4$ was recrystallized from deoxygenated ethanol and stored under a nitrogen atmosphere before use. Commercial $[Cu(NCMe)_4]PF_6$ was recrystallized from hot acetonitrile prior to use and kept under a nitrogen atmosphere avoiding light. The purification followed the standard procedure.[25]

The following materials were synthesized based on reported procedures or slight modifications thereof:
1,4-$HC{\equiv}CC_6H_4NO_2$[26]
1,4-$Me_3SiC{\equiv}CC_6H_4C{\equiv}C$-1,4-$C_6H_4I$[27]
1,4-$HC{\equiv}CC_6H_4$-1,4-$C_6H_4C{\equiv}C$-4-$C{\equiv}CPh$[28]
*trans*-$[Ru(C{\equiv}CPh)Cl(dppe)_2]$[29,30]
1,3-{*trans*-$[(dppe)_2(Cl)Ru(C{\equiv}C)]\}_2$-5-$HC{\equiv}CC_6H_3$ (**10**)[31]
1,3-{*trans*-$[(dppe)_2$(1,4-$O_2NC_6H_4C{\equiv}C)Ru(C{\equiv}C)]\}_2$-5-$HC{\equiv}CC_6H_3$ (**11**)[32]
1,3-{*trans*-$[(dppe)_2(Cl)Ru(C{\equiv}C)]\}_2$-5-{1,4-(*i*-Pr)$_3SiC{\equiv}CC_6H_4C{\equiv}C$-1,4-$C_6H_4C{\equiv}C$-1,4-$C_6H_4C{\equiv}C\}C_6H_3$ (**16**)[2]
1,3-{*trans*-$[(dppe)_2(Cl)Ru(C{\equiv}C)\}_2$-5-{1,4-(*i*-Pr)$_3SiC{\equiv}CC_6H_4C{\equiv}C$-3,5-$Et_2$-1,4-$C_6H_2C{\equiv}C$-1,4-$C_6H_4C{\equiv}C\}C_6H_3$ (**17**)[2]
1,3-{*trans*-$[(dppe)_2$(1,4-$IC_6H_4C{\equiv}C$-1,4-$C_6H_4C{\equiv}C)Ru(C{\equiv}C)]\}_2$-5-{(*i*-Pr)$_3SiC{\equiv}C$-1,4-$C_6H_4C{\equiv}C$-3,5-$Et_2$-1,4-$C_6H_2C{\equiv}C$-1,4-$C_6H_4C{\equiv}C\}C_6H_3$ (**18**)[2]
1,3-{*trans*-$[(dppe)_2$(1,4-$IC_6H_4C{\equiv}C$-1,4-$C_6H_4C{\equiv}C)Ru(C{\equiv}C)]\}_2$-5-(1,4-$HC{\equiv}CC_6H_4C{\equiv}C$-3,5-$Et_2$-1,4-$C_6H_2C{\equiv}C$-1,4-$C_6H_4C{\equiv}C)C_6H_3$ (**19**)[2]
1,3-{*trans*-$[(dppe)_2(PhC{\equiv}C)Ru(C{\equiv}C)]\}_2$-5-{(*i*-Pr)$_3SiC{\equiv}C$-1,4-$C_6H_4C{\equiv}C$-1,4-$C_6H_4C{\equiv}C$-1,4-$C_6H_4C{\equiv}C\}C_6H_3$ (**20**)[2]
1,3-{*trans*-[(1,3-*trans*-$[(dppe)_2(PhC{\equiv}C)Ru(C{\equiv}C)]_2C_6H_3$-5-$C{\equiv}C$-1,4-$C_6H_4C{\equiv}C$-1,4-$C_6H_4C{\equiv}C)Ru(dppe)_2(C{\equiv}C)]\}_2$-5-{(*i*-Pr)$_3SiC{\equiv}C$-1,4-$C_6H_4C{\equiv}C$-1,4-$C_6H_4C{\equiv}C$-1,4-$C_6H_4C{\equiv}C\}C_6H_3$ (**24**)[2]
1,3,5-{*trans*-[{3,5-{*trans*-$[(dppe)_2$(1,4-$IC_6H_4C{\equiv}C$-2,6-$Et_2$-1,4-$C_6H_2C{\equiv}C$-1,4-$C_6H_4C{\equiv}C)Ru(C{\equiv}C)]\}_2C_6H_3$-1-($C{\equiv}C$-1,4-$C_6H_4C{\equiv}C$-1,4-$C_6H_4C{\equiv}C)\}Ru(dppe)_2\{C{\equiv}C$-1,4-$C_6H_4C{\equiv}C$-1,4-$C_6H_4C{\equiv}C\}]\}_3C_6H_3$ (**35**)[2]
1,3,5-{*trans*-$[(dppe)_2(Cl)Ru(C{\equiv}C$-1,4-$C_6H_4C{\equiv}C$-1,4-$C_6H_4C{\equiv}C)]\}_3C_6H_3$ (**$0G_{20}$-Cl**)[33]
1,3,5-{*trans*-$[(dppe)_2(PhC{\equiv}C)Ru(C{\equiv}C$-1,4-$C_6H_4C{\equiv}C$-1,4-$C_6H_4C{\equiv}C)]\}_3C_6H_3$ (**$0G_{21}$**)[33]
1,3,5-{*trans*-$[(dppe)_2$(1,4-$O_2NC_6H_4C{\equiv}C)Ru(C{\equiv}C$-1,4-$C_6H_4C{\equiv}C$-1,4-$C_6H_4C{\equiv}C)]\}_3C_6H_3$ (**$0G_{21}$-$NO_2$**)[33]
1,3,5-{*trans*-[{1,3-{*trans*-$[(dppe)_2(Cl)Ru(C{\equiv}C)]\}_2C_6H_3$-5-($C{\equiv}C$-1,4-$C_6H_4C{\equiv}C$-1,4-$C_6H_4C{\equiv}C)\}Ru(dppe)_2(C{\equiv}C$-1,4-$C_6H_4C{\equiv}C$-1,4-$C_6H_4C{\equiv}C)]\}_3C_6H_3$ (**$1G_{22,00}$-Cl**)[2]
1,3,5-{*trans*-[{1,3-{*trans*-$[(dppe)_2(Cl)Ru(C{\equiv}C)]\}_2C_6H_3$-5-($C{\equiv}C$-1,4-$C_6H_4C{\equiv}C$-1,4-$C_6H_4C{\equiv}C)\}Ru(dppe)_2(C{\equiv}C$-1,4-$C_6H_4C{\equiv}C)]\}_3C_6H_3$ (**$1G_{12,00}$-Cl**)[34]
1,3,5-{*trans*-[{1,3-{*trans*-$[(dppe)_2(PhC{\equiv}C)Ru(C{\equiv}C)]\}_2C_6H_3$-5-($C{\equiv}C$-1,4-$C_6H_4C{\equiv}C$-1,4-$C_6H_4C{\equiv}C)\}Ru(dppe)_2(C{\equiv}C$-1,4-$C_6H_4C{\equiv}C)]\}_3C_6H_3$ (**$1G_{12,01}$**)[34]

1,3,5-{*trans*-[{1,3-{*trans*-[(1,3-{*trans*-[(dppe)$_2$(Cl)RuC≡C]}$_2$C$_6$H$_3$-5-C≡C-1,4-C$_6$H$_4$C≡C-1,4-C$_6$H$_4$C≡C)Ru(dppe)$_2$(C≡C)]}$_2$C$_6$H$_3$-5-(C≡C-1,4-C$_6$H$_4$C≡C-1,4-C$_6$H$_4$C≡C)}Ru(dppe)$_2$(C≡C-1,4-C$_6$H$_4$C≡C)]}$_3$C$_6$H$_3$ (**2G$_{12,02,00}$-Cl**)[2]

1,3,5-{*trans*-[{1,3-{*trans*-[(1,3-{*trans*-[(dppe)$_2$(PhC≡C)RuC≡C]}$_2$-5-C≡CC$_6$H$_3$-1,4-C≡CC$_6$H$_4$-1,4-C$_6$H$_4$C≡C)Ru(dppe)$_2$(C≡C)]}$_2$C$_6$H$_3$-1-(C≡C-1,4-C$_6$H$_4$C≡C-1,4-C$_6$H$_4$C≡C)}Ru(dppe)$_2$(C≡C-1,4-C$_6$H$_4$C≡C)]}$_3$C$_6$H$_3$ (**2G$_{12,02,01}$**)[34]

1,3,5-{*trans*-[1,3-{*trans*-[1,3-{*trans*-[(dppe)$_2$(Cl)Ru(C≡C)]}$_2$C$_6$H$_3$-5-(C≡C-1,4-C$_6$H$_4$C≡C-2,6-Et$_2$-1,4-C$_6$H$_2$C≡C-1,4-C$_6$H$_4$C≡C)Ru(dppe)$_2$(C≡C)]}$_2$C$_6$H$_3$-5-(C≡C-1,4-C$_6$H$_4$C≡C-1,4-C$_6$H$_4$C≡C)Ru(dppe)$_2$(C≡C-1,4-C$_6$H$_4$C≡C-1,4-C$_6$H$_4$C≡C)]}$_3$C$_6$H$_3$ (**2G$_{22,03,00}$-Cl**)[2]

1,3,5-{*trans*-[{1,3-{*trans*-[(1,3-{*trans*-[(dppe)$_2$(PhC≡C)Ru(C≡C)]}$_2$-5-C$_6$H$_3$(C≡C-1,4-C$_6$H$_4$C≡C-1,4-C$_6$H$_4$C≡C-1,4-C$_6$H$_4$C≡C)Ru(dppe)$_2$(C≡C)]}$_2$C$_6$H$_3$-5-(C≡C-1,4-C$_6$H$_4$C≡C-1,4-C$_6$H$_4$C≡C)}Ru(dppe)$_2$(C≡C-1,4-C$_6$H$_4$C≡C-1,4-C$_6$H$_4$C≡C)]}$_3$C$_6$H$_3$ (**2G$_{22,03,01}$**)[2]

1,3,5-{*trans*-[1,3-{*trans*-[1,3-{*trans*-[1,3-{*trans*-[(dppe)$_2$(PhC≡C)Ru(C≡C)]}$_2$C$_6$H$_3$-5-(C≡C-1,4-C$_6$H$_4$C≡C-1,4-C$_6$H$_4$C≡C)Ru(dppe)$_2$(C≡C)]}$_2$C$_6$H$_3$-5-(C≡C-1,4-C$_6$H$_4$C≡C-1,4-C$_6$H$_4$C≡C-1,4-C$_6$H$_4$C≡C)Ru(dppe)$_2$(C≡C)]}$_2$C$_6$H$_3$-5-(C≡C-1,4-C$_6$H$_4$C≡C-1,4-C$_6$H$_4$C≡C)Ru(dppe)$_2$(C≡C-1,4-C$_6$H$_4$C≡C-1,4-C$_6$H$_4$C≡C)]}$_3$C$_6$H$_3$ (**3G$_{22,03,02,01}$**)[2]

1,3,5-{*trans*-[1,3-{*trans*-[1,3-{*trans*-[(dppe)$_2$(PhC≡C)Ru(C≡C)]}$_2$C$_6$H$_3$-5-(C≡C-1,4-C$_6$H$_4$C≡C-2,6-Et$_2$-1,4-C$_6$H$_2$C≡C-1,4-C$_6$H$_4$C≡C)Ru(dppe)$_2$(C≡C)]}$_2$C$_6$H$_3$-5-(C≡C-1,4-C$_6$H$_4$C≡C-1,4-C$_6$H$_4$C≡C)Ru(dppe)$_2$(C≡C-1,4-C$_6$H$_4$C≡C-1,4-C$_6$H$_4$C≡C)]}$_3$C$_6$H$_3$ (**2G$_{22,03,01}$-s**)[2]

1,3,5-{*trans*-[1,3-{*trans*-[1,3-{*trans*-[1,3-{*trans*-[(dppe)$_2$(PhC≡C)Ru(C≡C)]}$_2$C$_6$H$_3$-5-(C≡C-1,4-C$_6$H$_4$C≡C-1,4-C$_6$H$_4$C≡C)Ru(dppe)$_2$(C≡C)]}$_2$C$_6$H$_3$-5-(C≡C-1,4-C$_6$H$_4$C≡C-2,6-Et$_2$-1,4-C$_6$H$_2$C≡C-1,4-C$_6$H$_4$C≡C)Ru(dppe)$_2$(C≡C)]}$_2$C$_6$H$_3$-5-(C≡C-1,4-C$_6$H$_4$C≡C-1,4-C$_6$H$_4$C≡C)Ru(dppe)$_2$(C≡C-1,4-C$_6$H$_4$C≡C-1,4-C$_6$H$_4$C≡C)]}$_3$C$_6$H$_3$ (**3G$_{22,03,02,01}$-s**)[2]

*trans*-[Ru(C≡CPh)$_2$(dppe)$_2$] (**1-M-1(dppe)**)[30]

*trans*-[Ru(C≡CPh)(C≡C-1,4-C$_6$H$_4$C≡CPh)(dppe)$_2$] (**1-M-2(dppe)**)[35]

# Instrumentation

Infrared spectra were recorded on a PerkinElmer Spectrum One FT-IR spectrometer using attenuated total reflectance (ATR). UV-vis spectra were recorded as $CH_2Cl_2$ solutions in 1 cm quartz cells using a PerkinElmer Lambda 950 spectrophotometer and are reported as $\nu_{max}$ ($cm^{-1}$) [ε ($10^3$ $M^{-1}$ $cm^{-1}$)]. Electrospray ionization (ESI) time-of-flight (TOF) mass spectrometry (MS) data were recorded using solutions in methanol or acetonitrile and a Waters LCT Premier XE TOF mass spectrometer; peaks are reported as *m/z* (assignment, relative intensity). Microanalyses were carried out at the London Metropolitan University. The TEM image was recorded on a TEM JEOL 2100F in STEM mode.

**Nuclear magnetic resonance (NMR) spectroscopy.** NMR spectra were recorded using Bruker Avance 400 MHz, 500 MHz, 600 MHz, 700 MHz, and 800 MHz and Varian 400 MHz NMR spectrometers and are referenced to residual $CHCl_3$ ($^1H$, 7.26 ppm) or $C_6D_5H$ ($^1H$, 7.16 ppm), $CDCl_3$ ($^{13}C$, 77.16 ppm) or $C_6D_6$ ($^{13}C$, 128.06 ppm), and external 85% $H_3PO_4$ ($^{31}P$, 0.0 ppm). Proton and carbon labelling of the spectral data for the new compounds start from the arene core, as shown in Fig. S1. Within each generation (e.g., from the core to the first-generation branching point as generation zero, from the first-generation branching point to the next branching point as generation one, etc.), atoms are labelled followed the dendrimer's extension direction and the label contains generation number followed by the number of the atom. The atoms in the ethyl groups are labelled as "s1" and "s2" to distinguish them from the dendrimer skeleton atoms. The atoms in the silyl protecting groups are labelled as "p1" and "p2". The Ru($\kappa^2$-dppe)$_2$ units are abbreviated as "[Ru]" with Greek letter subscript for generation number (α, β, γ, δ for generations 0, 1, 2, 3, respectively). For each ruthenium centre, the labelling starts from carbons on the ethylene group through all the carbons on the aromatic rings. All the protons attached to the same carbon atom that are chemically equivalent share the same number. Peaks in the $^{13}C\{^1H\}$ spectra were assigned with the assistance of 2D spectra (H-H COSY, H-C HSQC and H-C HMBC), and are labelled as "$C_{xx}$" (xx represents the atom's number). Proton peaks are labelled as "$H_{xx}$" in the same manner.

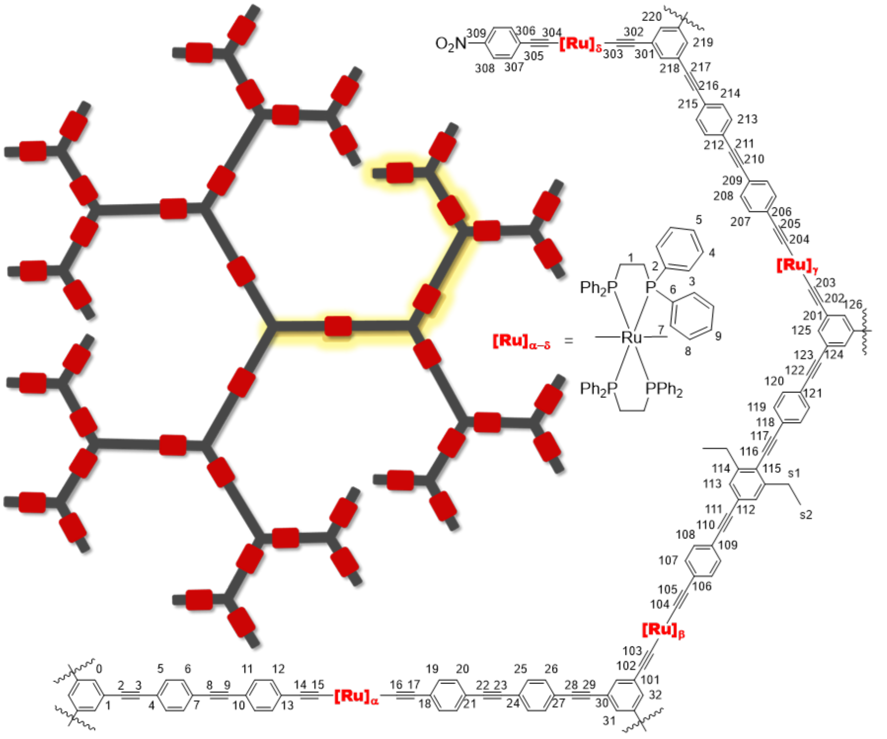


**Fig. S1** $^1H$ and $^{13}C$ NMR labelling for new compounds.

**Diffusion-ordered spectroscopy (DOSY).** All DOSY experiments were performed on a Bruker Avance 600 MHz NMR instrument with a cryoprobe. The standard Bruker pulse sequence **dstebpgp3s**, employing a double stimulated echo sequence, bipolar gradient pulsed for diffusion, and three spoil gradients, was chosen to minimize the convection effect. The gradient amplitudes were set from 2% to 95 % in 32 linear steps. The gradient duration and the diffusion time were optimized for each sample and were in the ranges 0.5-2.5 ms and 100-200 ms, respectively. Four dummy scans were used for temperature equilibration in the sample. The recycle delay was adjusted for each sample by measuring $T_1$ values (using the standard inversion recovery method) for all the resonances of interest and setting the recycle delay to 5 times the $T_1$ value of the most slowly relaxing resonance of interest. Signal intensities *I* were measured by integration. Plots of $ln(I/I_0)$ *vs.* $mG^2$ were fitted using a standard linear regression algorithm implemented in the Bruker TopSpin 3.5 pl 7 with $T_1/T_2$ module. The diffusion coefficients of three different molecules (methanol, phenol, and anthracene) were measured in $CDCl_3$ solution for comparison to literature values.[36] At 298.0 ± 0.1 K, the diffusion coefficients of the three compounds were found to be: methanol, $D = 3.310 \times 10^{-9}$ m$^2$ s$^{-1}$; phenol, $D = 2.016 \times 10^{-9}$ m$^2$ s$^{-1}$; anthracene, $D = 1.742 \times 10^{-9}$ m$^2$ s$^{-1}$, which agree with the reported values.[36]

Due to the temperature dependence of the diffusion coefficients, as well as the solvent viscosity, the actual sample temperature inside the probe must be calibrated before experiments, following the standard procedure in the Bruker manual. Neat methanol samples were used to calibrate the actual temperatures. Results showed that the temperature was maintained at a constant 298.0 ± 0.1 K after 20 min in the NMR bore.

The gradient strength was calibrated using literature values for water diffusion in $HDO/D_2O$ obtained from Aldrich ($D_2O$ % = 99.96%).[37]

For NMR diffusion measurements, the samples were placed into 5 mm NMR tubes to a height of 40 mm. The magnetic susceptibility of the sample tube was closely matched to solution samples, thereby obtaining good magnetic homogeneity over the whole sample volume while keeping good signal-to-noise ratio in the $^1H$ NMR spectrum. The concentration of samples was ca. $10^{-4}$ M in $CDCl_3$ solution at room temperature to ensure that the solution viscosity could be approximated by that of pure $CDCl_3$.

The diffusion coefficient (*D*) can be determined from the slope of the Stejskal-Tanner equation:

$$In\left(\frac{I}{I_0}\right) = -\gamma^2\delta^2G^2\left(\Delta - \frac{\delta}{3}\right)D = -mG^2D \tag{1}$$

$$m = \gamma^2\delta^2(\Delta - \frac{\delta}{3}) \tag{2}$$

where *G* is the gradient field strength, *I* is the integral of the peak area at a given value *G*, $\gamma$ is the magnetogyric constant of the nucleus which equals $2.675 \times 10^8$ T$^{-1}$ s$^{-1}$ for $^1H$, $\delta$ is the diffusion length parameter, and $\Delta$ is the diffusion delay. The diffusion coefficient can then afford the molecular size from the Stokes-Einstein equation:

$$D = \frac{k_BT}{f} = \frac{k_BT}{6\pi\eta r_o} \tag{3}$$

where $r_o$ is the hydrodynamic radius of a spherical particle, $\eta$ is the viscosity of the solution, $k_B$ is the Boltzmann constant, *T* is the absolute temperature, and *f* is the so-called hydrodynamic frictional coefficient. The equation is valid for solute molecules at infinite dilution diffusing through a continuum solvent where the solvent molecules are much smaller than the solute.

**Size Exclusion Chromatography (SEC).** The molecular weight distributions of the samples were determined using high-performance gel liquid chromatography on a Viscotek GPC Max VE2001 fitted with a Viscotek TDA 305 triple detector array consisting of a differential viscometer, right-angle laser-light scattering, low-angle laser-light scattering, and refractive index detectors. The column set consisted

of a Viscotek TGuard Organic Guard Column (10 × 4.6 mm) and two Viscotek LT5000L Mixed Medium Organic Columns (300 × 7.8 mm, 300 × 8.0 mm). The system was fitted with an online solvent degasser system and the eluent (THF) flow rate was set to 1 mL min$^{-1}$ with columns held at 30 °C. Calibration was carried out using PolyCAL™ poly(methyl methacrylate) standards with Mp = 800, 2380, 5050, 9680, 18700, 41400, 88500, 202000, 340000, 608000, and 988000 using OmniSEC software version 4.6.1.354. Injection volumes of 100 µL were employed.

**Cubic NLO and MPA studies.** The real and imaginary parts of the second hyperpolarizability γ ($\gamma_{real}$ and $\gamma_{imag}$, respectively) were determined using the Z-scan technique. An amplified femtosecond laser system consisting of an Integra-C regenerative amplifier (Quantronix) operating as an 800 nm pump and a Palitra-FS BIBO crystal-based optical parametric amplifier (Quantronix) was used. The system was tunable over a wavelength range from 650-2520 nm, delivering 130 fs pulses at a 1 kHz repetition rate. The output wavelength was confirmed using an Ocean Optics USB2000+ spectrometer (650-1000 nm) or an Ocean Optics NIRQuest+ spectrometer (1000-2500 nm). Coloured glass filters and a Thorlabs polarizing filter were used to remove unwanted wavelengths, and the power was adjusted by use of neutral density filters to obtain nonlinear phase shifts between 0.2 and 1.3 rad. The focal length of the lens used in the experiment was 75 mm, which gave 25-50 µm beam waists resulting in Rayleigh lengths longer than that of the sample thickness. Measurements were made in 1 mm optical cells, such that the total thickness was ⩽ 3 mm including the glass walls, so results could safely be treated using the thin-sample approximation. Samples travelled down the Z-axis on a Thorlabs motorized stage between -20 and +20 mm (where 0 was the laser focus). Data were collected by three Thorlabs photodiodes, 650-1100 nm with Si-based detectors, 1100-1700 nm with InGaAs detectors, and 1700-2520 nm with amplified InGaAs detectors. Data from the detectors were collected by a Tektronix oscilloscope with a custom LabVIEW program and curve-fitted with theoretical traces computed using equations derived by Sheik-Bahae et al.[38]

All measurements were calibrated against closed-aperture Z-scans of the solvent, as well as those of a 3 mm thick silica plate. The real and imaginary components of the second hyperpolarizability (γ) of the materials were calculated assuming additivity of the contributions of the solvent and the solute. The negative $\gamma_{real}$ maximal values approximately coincide with the positive maximal values of $\gamma_{imag}$, and are therefore consistent with the expected dependence of $\gamma_{real}$ on all nonlinear absorption processes through a nonlinear Kramers-Krönig relationship.[39,40]

Samples were prepared with the concentration around 0.3 w/w% in deoxygenated and distilled $CH_2Cl_2$. Overall, the fitting of data is consistent with n-photon absorption (nPA) in the indicated spectral regions, although fits at the extrema of the indicated ranges did not necessarily fit the corresponding ideal multi-photon curves, which may suggest that multiple absorptive processes occur at these wavelengths. Data at wavelengths for which assignment is uncertain are colored gray in the MPA spectral dependence plots. The analysis focuses on the maximal values for which the nPA assignments are unambiguous.

Multi-photon absorption cross-sections were calculated using[41]:

$$\sigma_{nPA} = \frac{\alpha_n(\hbar\omega)^{n-1}}{N_0} \quad (4)$$

where $n$ = 2, 3, 4, 5, and 6, and $\alpha_n$ is the appropriate nonlinear absorption coefficient, while $N_0$ is the molecular number density.

**Structural modelling.** The molecular modelling program SPARTAN 18 (Wavefunction, Inc.) was employed to model the hydrodynamic behaviour of the ruthenium dendrimers in solution. The molecular equilibrium geometries of the ground states were optimized using molecular mechanics and the SYBYL force field within the program package.

## Syntheses and characterization

***Synthesis of 1,3-{trans-[(dppe)$_2$(1,4-O$_2$NC$_6$H$_4$C≡C)Ru(C≡C)]}$_2$-5-{1,4-(i-Pr)$_3$SiC≡CC$_6$H$_4$C≡C-1,4-C$_6$H$_4$C≡C-1,4-C$_6$H$_4$C≡C}C$_6$H$_3$ (22).***

Compound **16** (0.240 g, 0.10 mmol) and 1,4-HC≡CC$_6$H$_4$NO$_2$ (0.036 g, 0.24 mmol) were added to distilled deoxygenated $CH_2Cl_2$ (50 mL) and 1 mL $NEt_3$ was added to the solution. $NaPF_6$ (0.104 g, 0.62 mmol) was added to the flask and the reaction was stirred for 16 h at room temperature. The crude product was obtained by passing the mixture through a Celite pad and removing the solvent *in vacuo*. Further purification was conducted by successive precipitations from MeOH (20 mL) and *n*-pentane (10 mL) to afford **22** as a red powder (0.253 g, 0.08 mmol, 82%). $^1$H NMR (700 MHz, CDCl$_3$, δ): 7.97 (d, $J$ = 8.9, 4H; $H_{208}$), 7.71-7.70 (m, 16H; $H_{[Ru]\gamma\text{-}3}$ or $H_{[Ru]\gamma\text{-}7}$), 7.60 (s, 4H; $H_{119}$, $H_{120}$), 7.54-7.53 (m, 4H; $H_{113}$, $H_{114}$), 7.47 (s, 4H; $H_{107}$, $H_{108}$), 7.24-7.23 (m, 16H; $H_{[Ru]\gamma\text{-}7}$ or $H_{[Ru]\gamma\text{-}3}$), 7.18-7.10 (m, 16H; $H_{[Ru]\gamma\text{-}5}$, $H_{[Ru]\gamma\text{-}9}$), 6.99-6.91 (m, 32H; $H_{[Ru]\gamma\text{-}4}$, $H_{[Ru]\gamma\text{-}8}$), 6.82 (s, 1H; $H_{126}$), 6.69 (s, 2H; $H_{125}$), 6.52 (d, $J$ = 8.8, 4H; $H_{207}$), 2.69 (m, 16H; $H_{[Ru]\gamma\text{-}1}$), 1.14 (s, 21H; $H_{p1}$, $H_{p2}$) ppm; $^{13}$C{$^1$H} NMR (176 MHz, CDCl$_3$, δ): 142.7 ($C_{209}$), 137.5 ($C_{206}$), 137.1, 136.4 ($C_{[Ru]\gamma\text{-}2}$, $C_{[Ru]\gamma\text{-}6}$), 134.5, 133.9 ($C_{[Ru]\gamma\text{-}3}$, $C_{[Ru]\gamma\text{-}7}$), 132.2 ($C_{108}$), 131.8 ($C_{107}$), 131.7, 131.6 ($C_{113}$, $C_{114}$), 130.1 ($C_{207}$), 129.9 ($C_{125}$, $C_{126}$), 129.2, 128.9 ($C_{[Ru]\gamma\text{-}5}$, $C_{[Ru]\gamma\text{-}9}$), 127.4, 127.3 ($C_{[Ru]\gamma\text{-}4}$, $C_{[Ru]\gamma\text{-}8}$), 124.5 ($C_{118}$ or $C_{121}$), 123.8 ($C_{109}$ or $C_{106}$), 123.5 ($C_{208}$), 123.3 ($C_{109}$ or $C_{106}$), 123.0, 122.6 ($C_{112}$, $C_{115}$), 121.6 ($C_{124}$), 118.7, 118.5 ($C_{202}$ or $C_{205}$), 106.8 ($C_{123}$), 93.2 ($C_{110}$), 91.4, 91.3, 91.1, 91.0 ($C_{116}$, $C_{111}$, $C_{117}$, $C_{104}$), 87.8 ($C_{122}$), 31.6 ($C_{[Ru]\gamma\text{-}1}$), 18.8 ($C_{p2}$), 11.5 ($C_{p1}$) ppm; $^{31}$P{$^1$H} NMR (162 MHz, CDCl$_3$, δ): 53.3 ppm; IR: $\nu$ = 2044 cm$^{-1}$ (C≡C); UV-vis (CH$_2$Cl$_2$, $\nu_{max}$ in cm$^{-1}$, [ε] in 10$^3$ M$^{-1}$ cm$^{-1}$): 20 650 [46.4], 28 900 [137.9]; HR ESI MS *m/z*: calcd. for $C_{165}H_{141}N_2O_4P_8Ru_2Si$: 2693.6706; found: 2693.6592 ([M + H]$^+$); Anal. calcd. for $C_{165}H_{140}N_2O_4P_8Ru_2Si$: C, 73.59; H, 5.24; N, 1.04%; found: C, 73.67; H, 5.13; N, 1.14%.

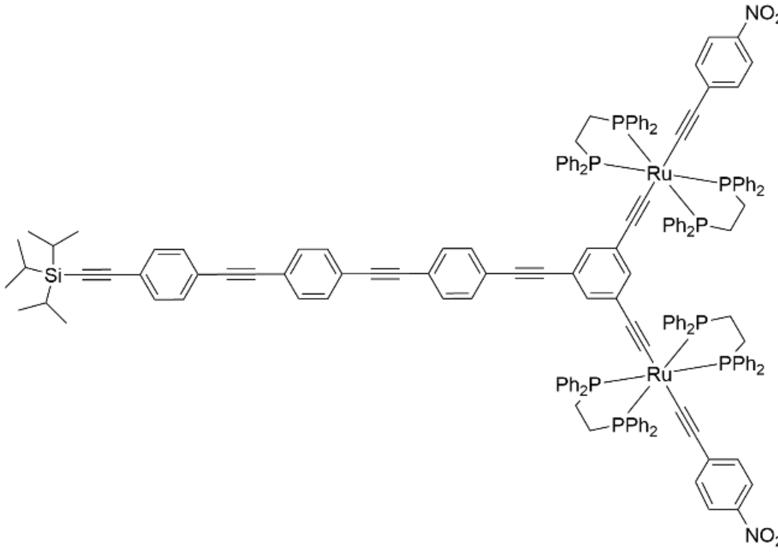

***Synthesis of 1,3-{trans-[(dppe)$_2$((1,4-$O_2NC_6H_4C{\equiv}C$)Ru(C≡C)]}$_2$-5-(1,4-$HC{\equiv}CC_6H_4C{\equiv}C$-1,4-$C_6H_4C{\equiv}C$-1,4-$C_6H_4C{\equiv}C$)$C_6H_3$ (23).***

Compound **22** (0.150 g, 0.056 mmol) was added to distilled, deoxygenated $CH_2Cl_2$ (60 mL), and several drops of $NEt_3$ and TBAF (1.0 M in THF, 0.1 mL) were added dropwise by syringe. The reaction was stirred at room temperature for 2 h. The crude product was obtained by removing the solvent *in vacuo*. Further purification was conducted by successive precipitations from MeOH (2 × 10 mL) and *n*-pentane (10 mL) to afford compound **23** as a red powder (0.134 g, 0.053 mmol, 95%). $^1$H NMR (700 MHz, $CDCl_3$, δ): 7.97 (d, $J$ = 8.8, 4H; $H_{208}$), 7.71-7.70 (m, 16H; $H_{[Ru]\gamma\text{-}3}$ or $H_{[Ru]\gamma\text{-}7}$), 7.60 (s, 4H; $H_{119}$, $H_{120}$),7.54-7.53 (m, 4H; $H_{113}$, $H_{114}$), 7.49 (s, 4H; $H_{107}$, $H_{108}$), 7.24-7.23 (m, 16H; $H_{[Ru]\gamma\text{-}7}$ or $H_{[Ru]\gamma\text{-}3}$), 7.18-7.10 (m, 16H; $H_{[Ru]\gamma\text{-}5}$, $H_{[Ru]\gamma\text{-}9}$), 6.99-6.91 (m, 32H; $H_{[Ru]\gamma\text{-}4}$, $H_{[Ru]\gamma\text{-}8}$), 6.82 (s, 1H; $H_{126}$), 6.69 (s, 2H; $H_{125}$), 6.53 (d, $J$ = 8.8, 4H; $H_{207}$), 3.19 (s, 1H; $H_{104}$), 2.69 (m, 16H; $H_{[Ru]\gamma\text{-}1}$) ppm; $^{13}$C{$^1$H} NMR (151 MHz, $CDCl_3$, δ): 142.7 ($C_{209}$), 137.5 ($C_{206}$), 137.1, 136.4 ($C_{[Ru]\gamma\text{-}2}$, $C_{[Ru]\gamma\text{-}6}$), 134.5, 133.9 ($C_{[Ru]\gamma\text{-}3}$, $C_{[Ru]\gamma\text{-}7}$), 132.3 ($C_{108}$), 131.8, 131.6 (m, $C_{107}$, $C_{108}$, $C_{113}$, $C_{114}$, $C_{119}$, $C_{120}$), 130.1 ($C_{207}$), 130.0, 129.9 ($C_{125}$, $C_{126}$), 129.2, 128.9 ($C_{[Ru]\gamma\text{-}5}$, $C_{[Ru]\gamma\text{-}9}$), 127.4, 127.3 ($C_{[Ru]\gamma\text{-}4}$, $C_{[Ru]\gamma\text{-}8}$), 124.5 ($C_{118}$ or $C_{121}$), 123.6 ($C_{109}$ or $C_{206}$), 123.5 ($C_{208}$), 123.4, 122.3 ($C_{112}$, $C_{115}$, $C_{118}$ or $C_{121}$), 121.6 ($C_{124}$), 118.7, 118.5 ($C_{202}$ or $C_{205}$), 93.2 ($C_{123}$), 91.4, 91.0 ($C_{110}$, $C_{111}$, $C_{116}$, $C_{117}$) , 87.8 ($C_{122}$), 83.4 ($C_{105}$), 79.2 ($C_{104}$), 31.6 ($C_{[Ru]\gamma\text{-}1}$) ppm; $^{31}$P{$^1$H} NMR (162 MHz, $CDCl_3$, δ): 53.3 ppm; IR: $v$ = 2042 $cm^{-1}$ (C≡C); HR ESI MS *m/z*: calcd. for $C_{156}H_{121}N_2O_4P_8Ru_2$: 2537.5370; found: 2537.5245 ($[M + H]^+$); Anal. calcd. for $C_{156}H_{120}N_2O_4P_8Ru_2$: C, 73.87; H, 4.77; N, 1.10%; found: C, 73.68; H, 4.73; N, 1.13%.

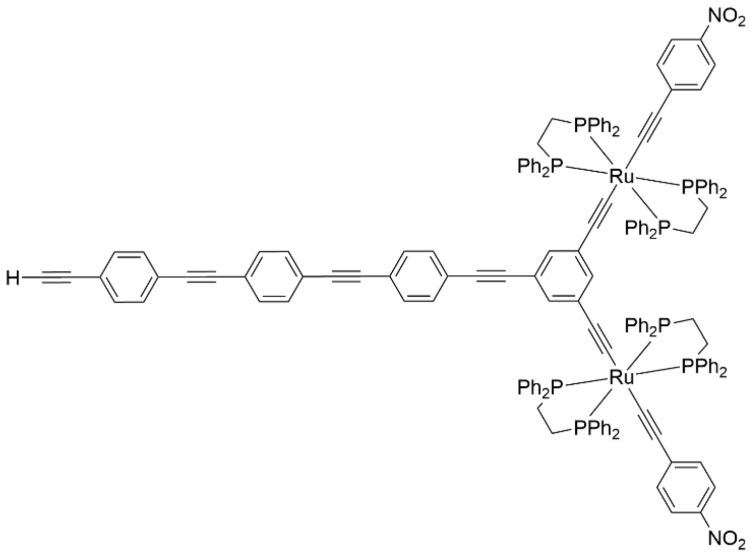

***Synthesis of 1,3-{trans-[(dppe)$_2$(1,4-O$_2$NC$_6$H$_4$C≡C)Ru(C≡C)]}$_2$-5-(1,4-Me$_3$SiC≡CC$_6$H$_4$C≡C-1,4-C$_6$H$_4$C≡C)C$_6$H$_3$ (14).***

Compound **11** (0.300 g, 0.134 mmol) and 1,4-Me$_3$SiC≡CC$_6$H$_4$C≡C-1,4-C$_6$H$_4$I (0.081 g, 0.20 mmol) were added to a 100 mL flask with solvent mixture CH$_2$Cl$_2$/NEt$_3$ (1:1, 60 mL). After degassing and backfilling with nitrogen three times, Pd(PPh$_3$)$_4$ (0.005 g, 0.004 mmol) and CuI (0.001 g, 0.004 mmol) were added to the solution. The reaction mixture was stirred at room temperature for three days. The solvent was removed *in vacuo* and the residue was purified by precipitation from stirring MeOH (2 × 20 mL). Further purification was conducted via alumina column chromatography (eluting with petrol/CH$_2$Cl$_2$ = 1:3) to afford compound **14** as a red powder (0.270 g, 0.11 mmol, 80%). $^1$H NMR (500 MHz, CDCl$_3$, δ): 7.99 (d, *J* = 9.0 Hz, 4H; H$_{305}$), 7.78-7.69 (m, 16H; H$_{261}$), 7.61 (s, 4H; H$_{210}$, H$_{211}$), 7.56-7.46 (m, 4H; H$_{204}$, H$_{205}$), 7.32-7.23 (m, 18H; H$_{251}$), 7.23-7.10 (m, 18H; H$_{253}$, H$_{263}$), 7.06-6.90 (m, H$_{252}$, H$_{262}$), 6.85 (s, 1H; H$_{23}$), 6.72 (s, 2H; H$_{21}$), 6.55 (d, *J* = 9.0 Hz, 4H; H$_{304}$), 2.78-2.65 (m, 18H; H$_{270}$), 0.29 (s, 9H; H$_{10}$) ppm; $^{13}$C{$^1$H} NMR (126 MHz, CDCl$_3$, δ): 142.5 (C$_{306}$), 137.4 (C$_{303}$), 136.9 (C$_{250}$), 136.2 (C$_{260}$), 134.3 (C$_{261}$), 133.7 (C$_{251}$), 131.9, 131.6 (C$_{204}$, C$_{205}$), 131.42, 131.38 (C$_{210}$, C$_{211}$), 129.9 (C$_{304}$), 129.8 (C$_{22}$), 129.0, 128.8 (C$_{263}$, C$_{253}$), 127.3, 127.1 (C$_{252}$, C$_{263}$), 124.3, 122.4 (C$_{209}$ or C$_{212}$), 121.3 (C$_{305}$), 123.1 (C$_{203}$, C$_{206}$), 121.4 (C$_{20}$), 118.5, 118.3 (C$_{302}$, C$_{215}$), 104.6 (C$_{203}$), 96.5 (C$_{201}$), 93.0 (C$_{214}$), 91.1 (C$_{214}$), 90.8 (C$_{207}$, C$_{208}$), 87.6 (C$_{213}$), 31.4 (C$_{270}$), -0.1 (C$_{10}$) ppm; $^{31}$P{$^1$H} NMR (202 MHz, CDCl$_3$, δ): 54.1 ppm; IR: $\nu$ = 2049 cm$^{-1}$ (C≡C); UV-vis (CH$_2$Cl$_2$, $\nu_{max}$ in cm$^{-1}$, [ε] in 10$^3$ M$^{-1}$ cm$^{-1}$): 20 600 [36.3], 28 500 [7.12, sh], 29 500 [8.61]; ESI MS *m/z*: 2509 ([M]$^+$, 65), 1944 (100), 1661 (10), 1190 (35), 1085 (82); HR ESI MS *m/z*: calcd. for C$_{151}$H$_{124}$N$_2$O$_4$P$_8$Ru$_2$Si: 2509.5397; found: 2509.5479; Anal. calcd. for C$_{151}$H$_{124}$N$_2$O$_4$P$_8$Ru$_2$Si: C 72.29, H 4.98, N 1.12%; found: C 72.36, H 5.14, N 1.01%.

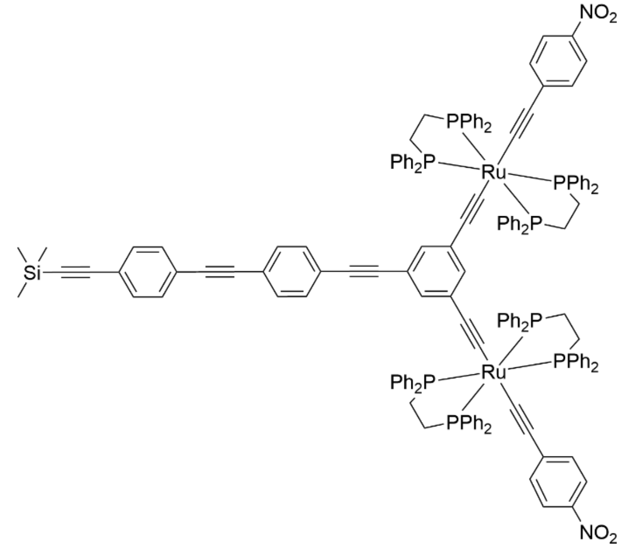

***Synthesis of 1,3-{trans-[(dppe)$_2$(1,4-O$_2$NC$_6$H$_4$C≡C)Ru(C≡C)]}$_2$-5-(1,4-HC≡CC$_6$H$_4$C≡C-1,4-C$_6$H$_4$C≡C)C$_6$H$_3$ (15)***.

Compound **14** (0.213 g, 0.085 mmol) was dissolved in distilled deoxygenated $CH_2Cl_2$ (80 mL) containing several drops of $NEt_3$, and TBAF (1.0 M solution in THF; 0.30 mL, 0.30 mmol) was added. The resultant solution was stirred at room temperature for 2 h. The volume of the solution was reduced to 2 mL *in vacuo*, and the residual mixture was dropped into stirring methanol. A dark red precipitate was formed, which was separated from the clear, orange-red solution by filtration through a sintered glass filter funnel. The solid thus obtained was redissolved in a minimal amount of $CH_2Cl_2$. The resultant, dark red solution was filtered through tightly packed cotton wool. The clear filtrate was dropped into stirring *n*-pentane, giving a flocculent, brick-red precipitate, which was separated from the clear, orange solution by filtration through a sintered glass filter funnel. A red solid was obtained that was dried under vacuum, affording 0.180 g (0.075 mmol, 88%) of the product. $^1$H NMR (500 MHz, $CDCl_3$, δ): 7.98 (d, *J* = 9.0 Hz, 4H; $H_{305}$), 7.75-7.68 (m, 16H; $H_{251}$), 7.60 (s, 4H; $H_{210}$, $H_{211}$), 7.54-7.47 (m, 4H; $H_{204}$, $H_{205}$), 7.26–7.20 (m, 16H; $H_{261}$), 7.20-7.15, 7.15–7.08 (m, 16H; $H_{261}$, $H_{251}$), 7.02-6.95, 6.95-6.88 (m, 32H; $H_{252}$, $H_{262}$), 6.83 (s, 1H; $H_{23}$), 6.69 (s, 2H; $H_{21}$), 6.52 (d, *J* = 9.0 Hz, 4H; $H_{304}$), 3.20 (s, 1H; $H_{201}$), 2.75-2.63 (m, 16H; $H_{270}$) ppm; $^{13}$C{$^1$H} NMR (126 MHz, $CDCl_3$, δ): 142.4 ($C_{306}$), 137.4 ($C_{303}$), 136.8 ($C_{250}$), 136.2 ($C_{260}$), 134.3, 133.7 ($C_{251}$, $C_{261}$), 132.1 ($C_{204}$), 131.7 ($C_{205}$), 131.5, 131.4 ($C_{210}$, $C_{211}$), 129.9 ($C_{304}$), 129.8 ($C_{23}$), 129.6 ($C_{21}$), 129.0, 128.7 ($C_{253}$, $C_{263}$), 127.2, 127.1 ($C_{252}$, $C_{262}$), 124.3, 122.3 ($C_{209}$, $C_{212}$), 123.5 ($C_{206}$), 123.3 ($C_{305}$), 122.0 ($C_{203}$), 121.3 ($C_{20}$), 118.5 ($C_{302}$), 118.3 ($C_{215}$), 93.0 ($C_{214}$), 91.2 ($C_{208}$), 90.6 ($C_{207}$), 87.6 ($C_{213}$), 83.2 ($C_{202}$), 79.1 ($C_{201}$), 31.4 (24 Hz, $C_{270}$) ppm; $^{31}$P{$^1$H} NMR (202 MHz, $CDCl_3$, δ): 54.0 ppm; IR: $v$ = 2046 cm$^{-1}$ (C≡C); UV-vis ($CH_2Cl_2$, $v_{max}$ in cm$^{-1}$, [ε] in $10^3$ M$^{-1}$ cm$^{-1}$): 20 600 [49.0], 28 500 [93.7], 29 700 [114.7]; ESI MS *m/z*: 2438 ([M + H]$^+$, 4), 1791 (2), 1689 (2), 1072 (58), 1051 (100); HR ESI MS *m/z*: calcd. for $C_{148}H_{117}N_2O_4P_8Ru_2$: 2437.5001; found: 2437.5039; Anal. calcd. for $C_{148}H_{116}N_2O_4P_8Ru_2$: C 72.96, H 4.80, N 1.15%; found: C 73.09, H 4.61, N 0.90%.

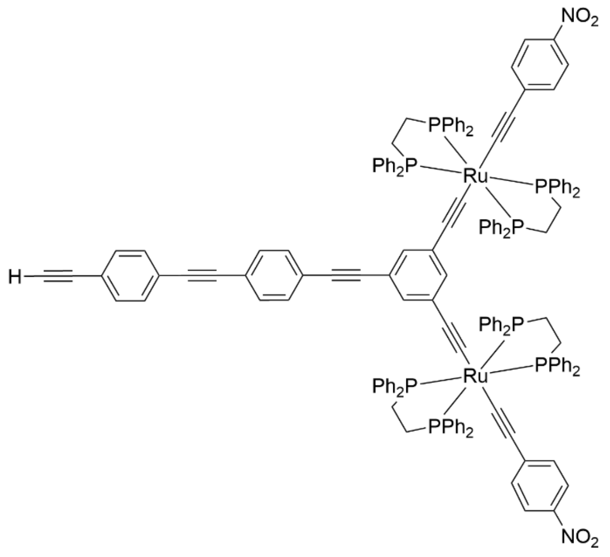

***Synthesis of 1,3-{trans-[(1,3-trans-[(dppe)$_2$(1,4-$O_2NC_6H_4C{\equiv}C$)Ru(C≡C)]$_2C_6H_3$-5-C≡C-1,4-$C_6H_4C{\equiv}C$-1,4-$C_6H_4C{\equiv}C$)Ru(dppe)$_2$(C≡C)]}$_2$-5-{1,4-(i-Pr)$_3SiC{\equiv}CC_6H_4C{\equiv}C$-1,4-$C_6H_4C{\equiv}C$-1,4-$C_6H_4C{\equiv}C$}$C_6H_3$ (26).***

Compound **16** (0.160 g, 0.065 mmol) and compound **15** (0.363 g, 0.15 mmol) were added to distilled deoxygenated $CH_2Cl_2$ (50 mL), and $NEt_3$ (1 mL) was added to the solution. $NaPF_6$ (0.218 g, 1.30 mmol) was added to the flask and the reaction was stirred for 16 h at room temperature. The crude product was obtained by passing the residue through a Celite pad and removing the solvent *in vacuo*. Further purification was conducted by several successive precipitations from MeOH (2 × 15 mL) and *n*-pentane (15 mL), to afford **26** as a red powder (0.430 g, 0.059 mmol, 91%). $^1$H NMR (400 MHz, $CDCl_3$, δ): 7.97 (d, $J$ = 8.7 Hz, 8H; $H_{308}$), 7.72-7.70 (m, 32H; $H_{[Ru]\delta\text{-}3}$ or $H_{[Ru]\delta\text{-}7}$), 7.66-7.64 (m, 16H; $H_{[Ru]\gamma\text{-}3}$ or $H_{[Ru]\gamma\text{-}7}$), 7.60-7.59 (m, 4H; $H_{119}$, $H_{120}$), 7.54 (m, 4H; $H_{113}$, $H_{114}$), 7.49, 7.47 (s, 4H; $H_{107}$, $H_{108}$), 7.36-7.34 (m, 16H; $H_{[Ru]\gamma\text{-}7}$ or $H_{[Ru]\gamma\text{-}3}$), 7.32 (m, 4H; $H_{208}$), 7.25-7.23 (m, 32H; $H_{[Ru]\delta\text{-}7}$ or $H_{[Ru]\delta\text{-}3}$), 7.20-7.09 (m, 48H; $H_{[Ru]\gamma\text{-}5}$, $H_{[Ru]\gamma\text{-}9}$, $H_{[Ru]\delta\text{-}5}$, $H_{[Ru]\delta\text{-}9}$), 7.00-6.91 (m, 96H; $H_{[Ru]\gamma\text{-}4}$, $H_{[Ru]\gamma\text{-}8}$, $H_{[Ru]\delta\text{-}4}$, $H_{[Ru]\delta\text{-}8}$), 6.82-6.60 (m, 21H; $H_{125}$, $H_{126}$, $H_{207}$, $H_{219}$, $H_{220}$, $H_{307}$, $H_{308}$), 6.52 (d, $J$ = 8.7 Hz, 8H; $H_{307}$), 2.62 (m, 48H; $H_{[Ru]\gamma\text{-}1}$, $H_{[Ru]\delta\text{-}1}$), 1.14 (s, 21H; $H_{p1}$, $H_{p2}$) ppm; $^{13}$C{$^1$H} NMR (176 MHz, $CDCl_3$ δ): 142.7 ($C_{309}$), 137.5 ($C_{306}$), 137.1, 136.4 (m, $C_{[Ru]\gamma\text{-}2}$, $C_{[Ru]\gamma\text{-}6}$, $C_{[Ru]\delta\text{-}2}$, $C_{[Ru]\delta\text{-}6}$), 134.5, 133.9 (m, $C_{[Ru]\gamma\text{-}3}$, $C_{[Ru]\gamma\text{-}7}$, $C_{[Ru]\delta\text{-}3}$, $C_{[Ru]\delta\text{-}7}$), 132.2 ($C_{108}$), 131.82, 131.75 ($C_{113}$, $C_{114}$), 131.6 ($C_{119}$, $C_{120}$), 131.0, 130.2, 130.1 ($C_{307}$), 130.0, 129.9, 129.2, 128.7 (m, $C_{[Ru]\gamma\text{-}4}$, $C_{[Ru]\gamma\text{-}8}$, $C_{[Ru]\delta\text{-}4}$, $C_{[Ru]\delta\text{-}8}$), 127.4, 127.2 (m, $C_{[Ru]\gamma\text{-}5}$, $C_{[Ru]\gamma\text{-}9}$, $C_{[Ru]\delta\text{-}5}$, $C_{[Ru]\delta\text{-}9}$), 124.6, 123.7, 123.6, 123.5 ($C_{308}$), 123.3, 123.2, 123.0, 122.4, 121.7, 121.3, 118.7, 118.5, 116.5, 106.8, 100.1, 93.5, 93.2, 92.9, 92.8, 91.5, 91.3, 91.0, 89.6, 88.0, 31.6 (m, $C_{[Ru]\gamma\text{-}1}$, $C_{[Ru]\delta\text{-}1}$), 18.8 ($C_{p2}$), 11.5 ($C_{p1}$) ppm; $^{31}$P{$^1$H} NMR (162 MHz, $CDCl_3$, δ): 53.6 (8P; $P_{[Ru]\gamma}$), 53.3 (16P; $P_{[Ru]\delta}$) ppm; IR: $\nu$ = 2043 cm$^{-1}$ (C≡C); UV-vis ($CH_2Cl_2$, $\nu_{max}$ in cm$^{-1}$, [ε] in $10^3$ M$^{-1}$ cm$^{-1}$): 20 650 [100.7, sh], 23 600 [130.3], 29 400 [292.9]; Anal. calcd. for $C_{445}H_{362}N_4O_8P_{24}Ru_6Si$: C, 73.50; H, 5.02; N, 0.77%; found: C, 73.59; H, 4.88; N, 0.79%.

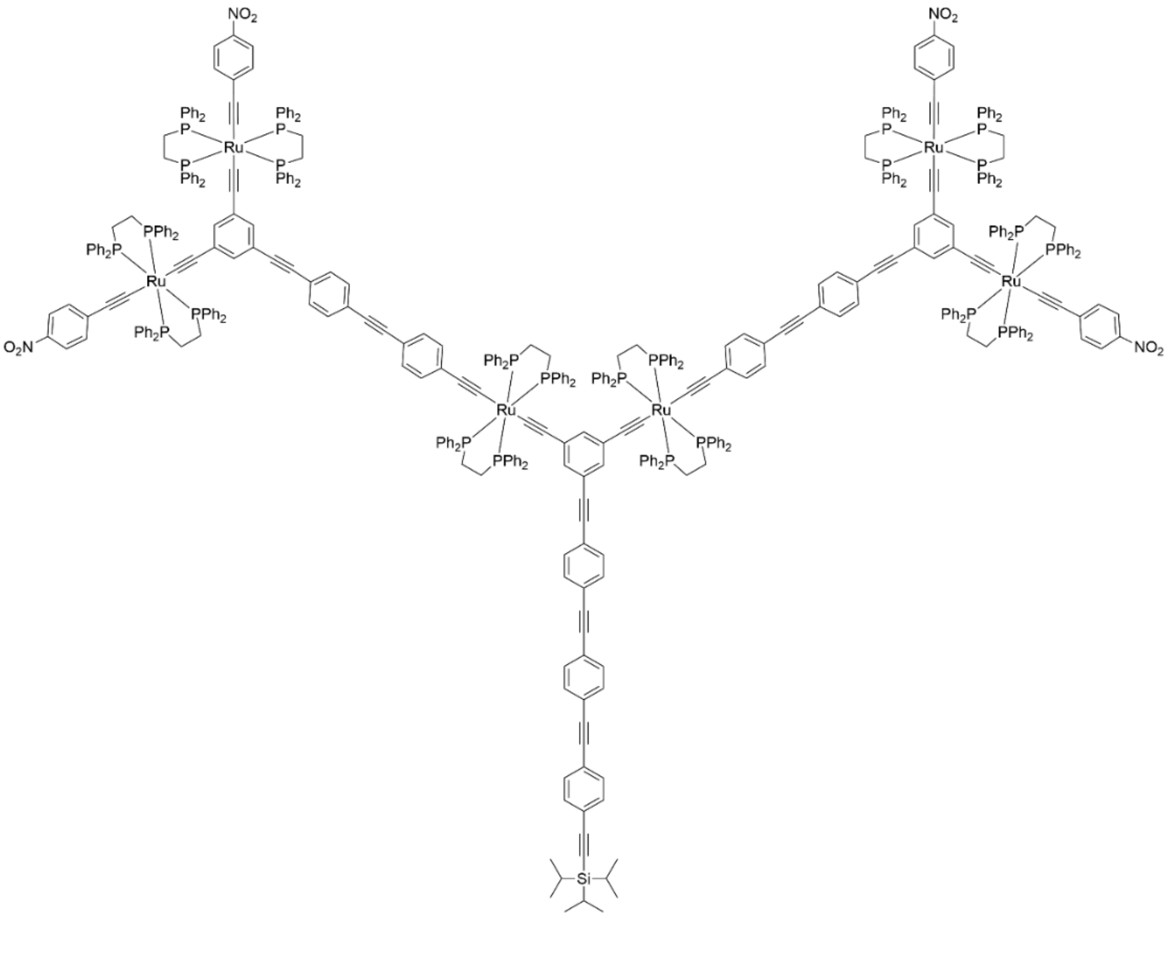

***Synthesis of 1,3-{trans-[(1,3-{trans-[(dppe)$_2$(1,4-$O_2NC_6H_4C$≡C)Ru(C≡C)]}$_2C_6H_3$-5-C≡C-1,4-$C_6H_4C$≡C-1,4-$C_6H_4C$≡C)(dppe)$_2$Ru(C≡C)]}$_2$-5-(1,4-HC≡C$C_6H_4C$≡C-1,4-$C_6H_4C$≡C-1,4-$C_6H_4C$≡C)$C_6H_3$ (27).***

Compound **26** (0.100 g, 0.014 mmol) was added to distilled deoxygenated $CH_2Cl_2$ (50 mL) and TBAF (0.1 mL, 1.0 M in THF) was added by syringe. The reaction was stirred for 16 h at room temperature. The crude product was obtained by removing the solvent *in vacuo*. Further purification was conducted by several successive precipitations from MeOH (2 × 15 mL) and *n*-pentane (20 mL), to afford a dark-red powder identified as compound **27** (0.075 g, 0.011 mmol, 75%). $^1$H NMR (700 MHz, $CDCl_3$, δ): 7.98-7.98 (d, $J$ = 8.6 Hz, 4H, $H_{308}$), 7.72 (m, 32H, $H_{[Ru]\delta\text{-}3}$ or $H_{[Ru]\delta\text{-}7}$), 7.67-7.66 (m, 16H, $H_{[Ru]\gamma\text{-}3}$ or $H_{[Ru]\gamma\text{-}7}$), 7.61-7.60 (m, 4H, $H_{119}$, $H_{120}$), 7.55-7.54 (m, 4H; $H_{113}$, $H_{114}$), 7.49 (m, 8H; $H_{213}$, $H_{214}$), 7.38-7.37 (m, 16H; $H_{[Ru]\gamma\text{-}7}$ or $H_{[Ru]\gamma\text{-}3}$), 7.35-7.33 (m, 4H; $H_{208}$), 7.26-7.25 (m, 32H; $H_{[Ru]\delta\text{-}7}$ or $H_{[Ru]\delta\text{-}3}$) 7.20-7.11 (m, 48H; $H_{[Ru]\gamma\text{-}5}$, $H_{[Ru]\gamma\text{-}9}$, $H_{[Ru]\delta\text{-}5}$, $H_{[Ru]\delta\text{-}9}$), 7.01-6.92 (m, 96H; $H_{[Ru]\gamma\text{-}4}$, $H_{[Ru]\gamma\text{-}8}$, $H_{[Ru]\delta\text{-}4}$, $H_{[Ru]\delta\text{-}8}$), 6.83-6.62 (m, 21H; $H_{207}$, $H_{219}$, $H_{220}$, $H_{126}$, $H_{125}$), 6.54-6.53 (d, $J$ = 8.5 Hz, 4H; $H_{307}$), 3.19 (s, 1H; $H_{104}$), 2.69 (m, 48H; $H_{[Ru]\gamma\text{-}1}$, $H_{[Ru]\delta\text{-}1}$) ppm; $^{13}$C{$^1$H} NMR (201 MHz, $CDCl_3$, δ): 142.7 ($C_{309}$), 137.5 ($C_{306}$), 137.1, 136.4 ($C_{[Ru]\gamma\text{-}2}$, $C_{[Ru]\gamma\text{-}6}$, $C_{[Ru]\delta\text{-}2}$, $C_{[Ru]\delta\text{-}6}$), 134.5, 133.9 ($C_{[Ru]\gamma\text{-}3}$, $C_{[Ru]\gamma\text{-}7}$, $C_{[Ru]\delta\text{-}3}$, $C_{[Ru]\delta\text{-}7}$), 132.3 ($C_{108}$), 131.8 ($C_{113}$, $C_{114}$), 131.6 ($C_{119}$, $C_{120}$), 131.0, 130.14, 130.07 ($C_{307}$), 129.2, 128.7 ($C_{[Ru]\gamma\text{-}5}$, $C_{[Ru]\gamma\text{-}9}$, $C_{[Ru]\delta\text{-}5}$, $C_{[Ru]\delta\text{-}9}$), 127.4, 127.3 ($C_{[Ru]\gamma\text{-}4}$, $C_{[Ru]\gamma\text{-}8}$, $C_{[Ru]\delta\text{-}4}$, $C_{[Ru]\delta\text{-}8}$), 127.2, 123.5 ($C_{308}$), 123.1, 122.3, 121.7, 118.7, 118.5 ($C_{302}$, $C_{305}$), 117.5, 116.6, 92.9, 91.5, 91.0, 89.5, 88.0, 87.6, 83.4, 31.6 ($C_{[Ru]\gamma\text{-}1}$, $C_{[Ru]\delta\text{-}1}$) ppm; $^{31}$P{$^1$H} NMR (162 MHz, $CDCl_3$, δ): 53.6 (16P; $P_{[Ru]\delta}$), 53.3 (8P; $P_{[Ru]\gamma}$) ppm; IR: $\nu$ = 2043 cm$^{-1}$ (C≡C); Anal. calcd. for $C_{436}H_{342}N_4O_8P_{24}Ru_6$: C, 73.60; H, 4.84, N, 0.79%; found: C, 73.49; H, 4.80; N, 0.80%.

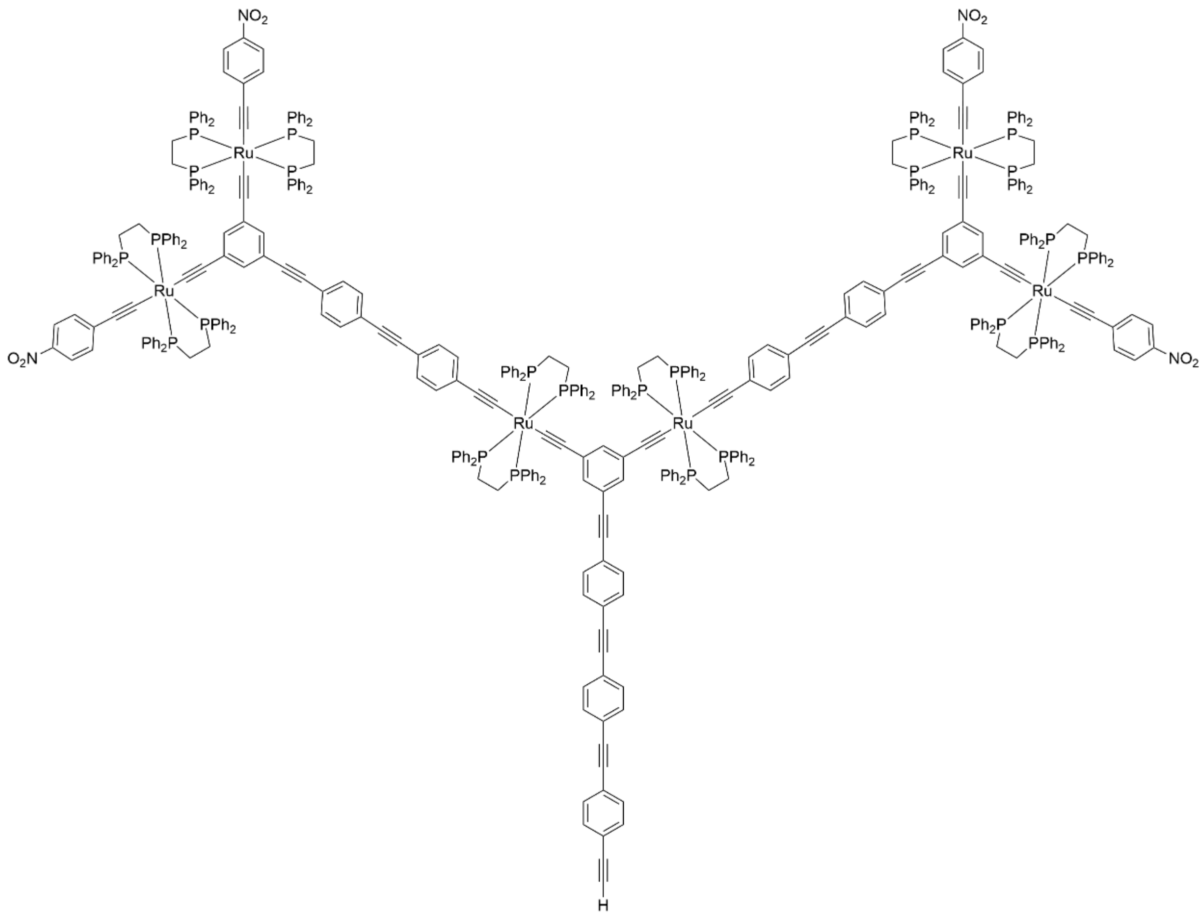

***Synthesis of 1,3,5-{trans-[{1,3-{trans-[(dppe)$_2$(1,4-O$_2$NC$_6$H$_4$C≡C)Ru(C≡C)]}$_2$C$_6$H$_3$-5-(C≡CC$_6$H$_4$-4-C≡CC$_6$H$_4$-4-C≡C)}Ru(dppe)$_2${C≡CC$_6$H$_4$-4-C≡C}]}$_3$C$_6$H$_3$ (1G$_{12,01}$-NO$_2$).***

Compound **1G$_{12,00}$-Cl** (0.150 g, 0.015 mmol) and 1,4-HC≡CC$_6$H$_4$NO$_2$ (0.016 mg, 0.11 mmol) were added to distilled deoxygenated $CH_2Cl_2$ (50 mL), and $NEt_3$ (1 mL) was added to the solution. $NaPF_6$ (0.022 g, 0.12 mmol) was added to the flask and the reaction was stirred for 16 h at room temperature. The crude product was obtained by passing the solution through a Celite pad and removing the solvent *in vacuo*. Further purification was conducted by precipitation from MeOH (2 × 15 mL) and *n*-pentane (20 mL)), to afford a red powder identified as **1G$_{12,01}$-NO$_2$** (0.132 g, 0.013 mmol, 82%). $^1$H NMR (800 MHz, CDCl$_3$, δ): 7.90 (d, *J* = 8.3 Hz, 12H; H$_{108}$), 7.63-7.44 (m, 144H; H$_{[Ru]\alpha\text{-}3}$, H$_{[Ru]\alpha\text{-}7}$, H$_{[Ru]\beta\text{-}3}$, H$_{[Ru]\beta\text{-}7}$), 7.28, 7.27, 7.17, 7.10-7.05 (m, 72H; H$_{[Ru]\alpha\text{-}5}$, H$_{[Ru]\alpha\text{-}9}$, H$_{[Ru]\beta\text{-}5}$, H$_{[Ru]\beta\text{-}9}$), 6.91-6.85 (m, 144H; H$_{[Ru]\alpha\text{-}4}$, H$_{[Ru]\alpha\text{-}8}$, H$_{[Ru]\beta\text{-}4}$, H$_{[Ru]\beta\text{-}8}$), 6.75-6.63 (m, 18H; H$_6$, H$_{25}$, H$_{26}$), 6.45 (d, *J* = 8.3 Hz, 12H; H$_{107}$), 2.62-2.59 (m, 72H; H$_{[Ru]\alpha\text{-}1}$, H$_{[Ru]\beta\text{-}1}$) ppm; $^{13}$C{$^1$H} NMR (176 MHz, CDCl$_3$, δ): 142.7 (C$_{109}$), 137.5 (C$_{106}$), 137.0, 136.4 (C$_{[Ru]\alpha\text{-}2}$, C$_{[Ru]\alpha\text{-}6}$, C$_{[Ru]\beta\text{-}2}$, C$_{[Ru]\beta\text{-}6}$), 134.5, 134.3, 133.9 (C$_{[Ru]\alpha\text{-}3}$, C$_{[Ru]\alpha\text{-}7}$, C$_{[Ru]\beta\text{-}3}$, C$_{[Ru]\beta\text{-}7}$), 131.6, 131.2, 131.1, 130.1 (C$_{107}$), 130.0, 129.9, 129.2, 128.9 (C$_{[Ru]\alpha\text{-}5}$, C$_{[Ru]\alpha\text{-}9}$, C$_{[Ru]\beta\text{-}5}$, C$_{[Ru]\beta\text{-}9}$), 127.4, 127.3 (C$_{[Ru]\alpha\text{-}4}$, C$_{[Ru]\beta\text{-}4}$, C$_{[Ru]\alpha\text{-}8}$, C$_{[Ru]\beta\text{-}7}$), 123.5 (C$_{108}$), 121.7, 118.7, 118.5, 116.8, 92.8, 88.3, 31.6 (C$_{[Ru]\alpha\text{-}1}$, C$_{[Ru]\beta\text{-}1}$) ppm; $^{31}$P{$^1$H} NMR (162 MHz, CDCl$_3$, δ): δ 53.4 (12P; P$_{[Ru]\alpha}$), 53.3 (24P; P$_{[Ru]\beta}$) ppm; IR: $\nu$ = 2043 cm$^{-1}$ (C≡C); UV-vis (CH$_2$Cl$_2$, $\nu_{max}$ in cm$^{-1}$, [ε] in 10$^3$ M$^{-1}$ cm$^{-1}$): 20 300 [137.8, sh], 23 550 [317.4], 30 200 [307.6]. Anal. calcd. for $C_{636}H_{504}N_6O_{12}P_{36}Ru_9$: C, 73.12; H, 4.86; N, 0.80%; found: C, 73.49; H, 4.80; N, 0.80%.

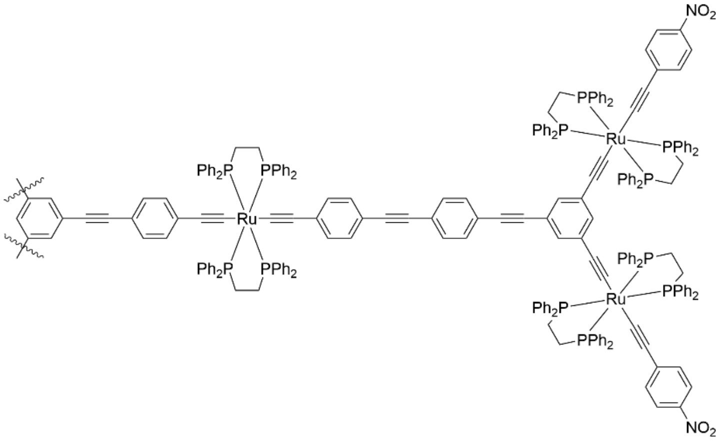

***Synthesis of 1,3,5-{trans-[{1,3-{trans-[(dppe)$_2$(PhC≡C)Ru(C≡C)]}$_2$C$_6$H$_3$-5-(C≡C-1,4-C$_6$H$_4$C≡C-1,4-C$_6$H$_4$C≡C)}Ru(dppe)$_2$(C≡C-1,4-C$_6$H$_4$C≡C-1,4-C$_6$H$_4$C≡C)]}$_3$C$_6$H$_3$ (1G$_{22,01}$).***

Compound **1G$_{22,00}$-Cl** (0.120 g, 0.012 mmol) and ethynylbenzene (0.016 mL, 0.14 mmol) were added to distilled $CH_2Cl_2$ (60 mL), and $NEt_3$ (1 mL) was added to the solution. $NaPF_6$ (0.022 g, 0.12 mmol) was added to the flask and the reaction was stirred at room temperature for three days. The crude product was obtained by passing the solution through a Celite pad and removing the solvent *in vacuo*. Further purification was conducted by precipitation from MeOH (2 × 15 mL) and *n*-pentane (10 mL) to afford a yellow powder identified as **1G$_{22,01}$** (0.102 g, 0.010 mmol, 82%). $^1$H NMR (700 MHz, $CDCl_3$, δ): 7.69 (s, 3H; $H_0$), 7.59-7.45 (m, 144H; $H_{[Ru]\alpha\text{-}3}$, $H_{[Ru]\alpha\text{-}7}$, $H_{[Ru]\beta\text{-}3}$, $H_{[Ru]\beta\text{-}7}$), 7.35-7.33 (m, 12H; $H_{11}$, $H_{20}$), 7.19-7.11 (m, 72H; $H_{[Ru]\alpha\text{-}5}$, $H_{[Ru]\alpha\text{-}9}$, $H_{[Ru]\beta\text{-}5}$, $H_{[Ru]\beta\text{-}9}$), 7.00 (s, 1H; $H_{109}$), ), 6.98-6.94 (m, 144H; $H_{[Ru]\alpha\text{-}4}$, $H_{[Ru]\alpha\text{-}8}$, $H_{[Ru]\beta\text{-}4}$, $H_{[Ru]\beta\text{-}8}$), 6.76-6.70 (m, 21H; $H_{32}$, $H_{107}$, $H_{19}$), 6.53 (m, 6H; $H_{31}$), 2.70-2.65 (m, 72H; $H_{[Ru]\alpha\text{-}1}$, $H_{[Ru]\beta\text{-}1}$) ppm; $^{13}$C{$^1$H} NMR (151 MHz, $CDCl_3$, δ): 137.3, 136.9 ($C_{[Ru]\alpha\text{-}2}$, $C_{[Ru]\alpha\text{-}6}$, $C_{[Ru]\beta\text{-}2}$, $C_{[Ru]\beta\text{-}6}$), 134.5, 134.3 ($C_{[Ru]\alpha\text{-}3}$, $C_{[Ru]\alpha\text{-}7}$, $C_{[Ru]\beta\text{-}3}$, $C_{[Ru]\beta\text{-}7}$), 131.8, 131.5, 131.1, 130.8 ($C_{106}$), 130.2, 130.1 ($C_{12}$, $C_{19}$, $C_{107}$), 129.8 ($C_{101}$), 128.9, 128.6 ($C_{[Ru]\alpha\text{-}4}$, $C_{[Ru]\alpha\text{-}8}$, $C_{[Ru]\beta\text{-}4}$, $C_{[Ru]\beta\text{-}8}$), 124.4, 124.2, 123.9, 122.9 ($C_{109}$), 121.2, 117.0, 116.3 ($C_{14}$, $C_{17}$, $C_{102}$, $C_{105}$), 93.2, 92.6, 90.7, 89.6, 89.4, 87.6, 31.8 ($C_{[Ru]\alpha\text{-}1}$, $C_{[Ru]\beta\text{-}1}$) ppm; $^{31}$P{$^1$H} NMR (283 MHz, $CDCl_3$, δ): 53.9 (24P; $P_{[Ru]\beta}$), 53.4 (12P; $P_{[Ru]\alpha}$) ppm; IR: $\nu$ = 2054 cm$^{-1}$ (C≡C); UV-vis ($CH_2Cl_2$, $\nu_{max}$ in cm$^{-1}$, [ε] in 10$^3$ M$^{-1}$ cm$^{-1}$): 29 900 [451.1], 23 400 [282.6]; Anal. calcd. for $C_{660}H_{522}P_{36}Ru_9$: C 75.65, H 5.02%; found: C 75.95, H 5.12%.

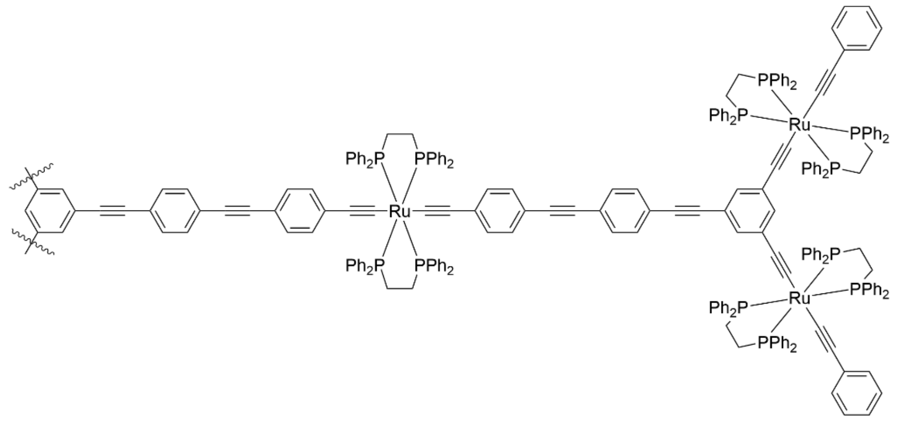

***Synthesis of 1,3,5-{trans-[{1,3-{trans-[(dppe)$_2$(1,4-O$_2$NC$_6$H$_4$C≡C)Ru(C≡C)]}$_2$C$_6$H$_3$-5-(C≡C-1,4-C$_6$H$_4$C≡C-1,4-C$_6$H$_4$C≡C)}Ru(dppe)$_2$(C≡C-1,4-C$_6$H$_4$C≡C-1,4-C$_6$H$_4$C≡C)]}$_3$C$_6$H$_3$ (1G$_{22,01}$-NO$_2$).***

Compound **1G$_{22,00}$-Cl** (0.150 g, 0.015 mmol) and 1,4-HC≡CC$_6$H$_4$NO$_2$ (0.022 g, 0.15 mmol) were added to distilled CH$_2$Cl$_2$ (60 mL), and NEt$_3$ (1 mL) was added to the solution. NaPF$_6$ (0.050 g, 0.30 mmol) was added to the flask and the reaction was stirred for 16 h at room temperature. The crude product was obtained by passing the solution through a Celite pad and removing the solvent *in vacuo*. Further purification was conducted by precipitation from MeOH (2 × 15 mL) and *n*-pentane (10 mL) to afford a dark red powder identified as **1G$_{22,01}$-NO$_2$**. (0.117 g, 0.011 mmol, 73%). $^1$H NMR (700 MHz, CDCl$_3$, δ): 7.99 (d, *J* = 8.4 Hz, 12H; H$_{108}$), 7.73–7.52 (m, 144H; H$_{[Ru]\alpha\text{-}3}$, H$_{[Ru]\alpha\text{-}7}$, H$_{[Ru]\beta\text{-}3}$, H$_{[Ru]\beta\text{-}7}$), 7.36, 7.20-7.14 (m, 72H; H$_{[Ru]\alpha\text{-}5}$, H$_{[Ru]\alpha\text{-}9}$, H$_{[Ru]\beta\text{-}5}$, H$_{[Ru]\beta\text{-}9}$), 6.84, 6.72 (m, 30H; H$_6$, H$_{11}$, H$_{12}$, H$_{25}$, H$_{26}$), 6.54 (d, *J* = 8.4 Hz, 12H; H$_{107}$), 2.71-2.66 (m, 72H; H$_{[Ru]\alpha\text{-}1}$, H$_{[Ru]\beta\text{-}1}$) ppm; $^{13}$C{$^1$H} NMR (151 MHz, CDCl$_3$, δ): 142.7 (C$_{109}$), 137.0, 136.4 (C$_{[Ru]\alpha\text{-}2}$, C$_{[Ru]\alpha\text{-}6}$, C$_{[Ru]\beta\text{-}2}$, C$_{[Ru]\beta\text{-}6}$), 134.5, 134.3, 134.0 (C$_{[Ru]\alpha\text{-}3}$, C$_{[Ru]\alpha\text{-}7}$, C$_{[Ru]\beta\text{-}3}$, C$_{[Ru]\beta\text{-}7}$), 131.8, 131.6, 131.1, 130.1 (C$_{107}$), 129.2, 128.9 (C$_{[Ru]\alpha\text{-}5}$, C$_{[Ru]\alpha\text{-}9}$, C$_{[Ru]\beta\text{-}5}$, C$_{[Ru]\beta\text{-}9}$), 127.4, 127.3 (C$_{[Ru]\alpha\text{-}4}$, C$_{[Ru]\alpha\text{-}8}$, C$_{[Ru]\beta\text{-}4}$, C$_{[Ru]\beta\text{-}8}$), 123.5 (C$_{108}$), 118.7, 118.5, 93.0, 89.4, 31.6 (C$_{[Ru]\alpha\text{-}1}$, C$_{[Ru]\beta\text{-}1}$) ppm; $^{31}$P{$^1$H} NMR (162 MHz, CDCl$_3$, δ): 53.4 (12P; P$_{[Ru]\alpha}$), 53.3 (24P; P$_{[Ru]\beta}$) ppm; IR: $\nu$ = 2047 cm$^{-1}$ (C≡C); UV-vis (CH$_2$Cl$_2$, $\nu_{max}$ in cm$^{-1}$, [ε] in 10$^3$ M$^{-1}$ cm$^{-1}$): 20 040 [112.0, sh], 23 260 [330.5], 29 950 [407.6]; Anal. calcd. for C$_{660}$H$_{516}$N$_6$O$_{12}$P$_{36}$Ru$_9$: C 73.76, H 4.84, N 0.78%; found: C 73.93, H 5.07, N 0.94%.

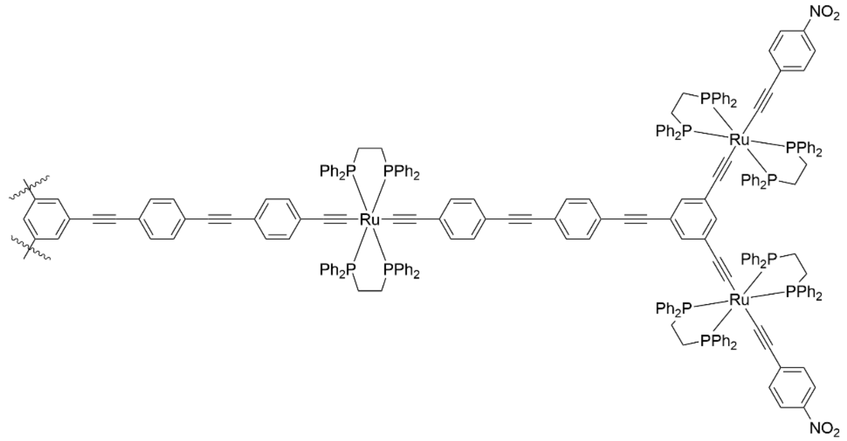

***Synthesis of 1,3,5-{trans-[{1,3-{trans-[(1,3-{trans-[(dppe)$_2$(1,4-O$_2$NC$_6$H$_4$C≡C)RuC≡C]}$_2$C$_6$H$_3$-5-C≡C-1,4-C$_6$H$_4$C≡C-1,4-C$_6$H$_4$C≡C)Ru(dppe)$_2$(C≡C)]}$_2$C$_6$H$_3$-5-(C≡C-1,4-C$_6$H$_4$C≡C-1,4-C$_6$H$_4$C≡C)}Ru(dppe)$_2$(C≡C-1,4-C$_6$H$_4$C≡C)]}$_3$C$_6$H$_3$ (2G$_{12,02,01}$-NO$_2$)***.

Compound **2G$_{12,02,00}$-Cl** (0.240 g, 0.011 mmol) and 1,4-HC≡CC$_6$H$_4$NO$_2$ (0.028 g, 0.19 mmol) were added to distilled deoxygenated CH$_2$Cl$_2$ (60 mL), and NEt$_3$ (1 mL) was added to the solution. NaPF$_6$ (0.051 g, 0.31 mmol) was added to the flask and the reaction was stirred for 16 h at room temperature. The crude product was obtained by passing the mixture through a Celite pad and removing the solvent *in vacuo*. Further purification was conducted by successive precipitations from MeOH (2 × 15 mL) and *n*-pentane (15 mL) to afford a red powder identified as **2G$_{12,02,01}$-NO$_2$** (0.225 g, 0.0093 mmol, 89%). $^1$H NMR (400 MHz, CDCl$_3$, δ): 7.98-7.96 (d, *J* = 8.8 Hz, 24H; H$_{208}$), 7.72-7.52 (m, 336H; H$_{[Ru]\alpha-3}$, H$_{[Ru]\alpha-7}$, H$_{[Ru]\beta-3}$, H$_{[Ru]\beta-7}$, H$_{[Ru]\gamma-3}$, H$_{[Ru]\gamma-7}$), 7.36, 7.32, 7.26, 7.18-7.12 (m, 168H; H$_{[Ru]\alpha-5}$, H$_{[Ru]\alpha-9}$, H$_{[Ru]\beta-5}$, H$_{[Ru]\beta-9}$, H$_{[Ru]\beta-9}$, H$_{[Ru]\gamma-5}$, H$_{[Ru]\gamma-9}$), 6.98-6.92 (m, 336H; H$_{[Ru]\alpha-4}$, H$_{[Ru]\alpha-8}$, H$_{[Ru]\beta-4}$, H$_{[Ru]\beta-8}$, H$_{[Ru]\gamma-4}$, H$_{[Ru]\gamma-8}$), 6.82, 6.70-6.61 (m, 27H; H$_6$, H$_{12}$, H$_{25}$, H$_{26}$, H$_{119}$, H$_{120}$), 6.53-6.51 (d, *J* = 8.8 Hz, 24H; H$_{207}$), 2.69 (m, 168H; H$_{[Ru]\alpha-1}$, H$_{[Ru]\beta-1}$, H$_{[Ru]\gamma-1}$) ppm; $^{13}$C{$^1$H} NMR (176 MHz, CDCl$_3$, δ): 142.7 (C$_{209}$), 137.5 (C$_{206}$), 137.0, 136.4 (m, C$_{[Ru]\alpha-2}$, C$_{[Ru]\alpha-6}$, C$_{[Ru]\beta-2}$, C$_{[Ru]\beta-6}$, C$_{[Ru]\gamma-2}$, C$_{[Ru]\gamma-6}$), 134.5, 133.9 (m, C$_{[Ru]\alpha-3}$, C$_{[Ru]\alpha-7}$, C$_{[Ru]\beta-3}$, C$_{[Ru]\beta-7}$, C$_{[Ru]\gamma-3}$, C$_{[Ru]\gamma-7}$), 131.6, 131.0, 130.1 (C$_{207}$), 129.2, 128.9 (m, C$_{[Ru]\alpha-5}$, C$_{[Ru]\alpha-9}$, C$_{[Ru]\beta-5}$, C$_{[Ru]\beta-9}$, C$_{[Ru]\gamma-5}$, C$_{[Ru]\gamma-9}$), 127.4, 127.3 (m, C$_{[Ru]\alpha-4}$, C$_{[Ru]\alpha-8}$, C$_{[Ru]\beta-4}$, C$_{[Ru]\beta-8}$, C$_{[Ru]\gamma-4}$, C$_{[Ru]\gamma-8}$), 123.5 (C$_{208}$), 31.6 (m, C$_{[Ru]\alpha-1}$, C$_{[Ru]\beta-1}$, C$_{[Ru]\gamma-1}$).ppm; $^{31}$P{$^1$H} NMR (162 MHz, CDCl$_3$, δ): 53.6 (24P; P$_{[Ru]\beta}$), 53.4, (12P; P$_{[Ru]\alpha}$), 53.3 (48P; P$_{[Ru]\gamma}$) ppm; IR: $\nu$ = 2043 cm$^{-1}$ (C≡C); UV-vis (CH$_2$Cl$_2$, $\nu_{max}$ in cm$^{-1}$, [ε] in 10$^3$ M$^{-1}$ cm$^{-1}$): 20 400 [311.0], 23 550 [625.6], 29 950 [823.0]; Anal. calcd. for C$_{1476}$H$_{1170}$N$_{12}$O$_{24}$P$_{84}$Ru$_{21}$: C, 73.31; H, 4.88; N, 0.70%; found: C, 73.17; H, 5.00; N, 0.76%.

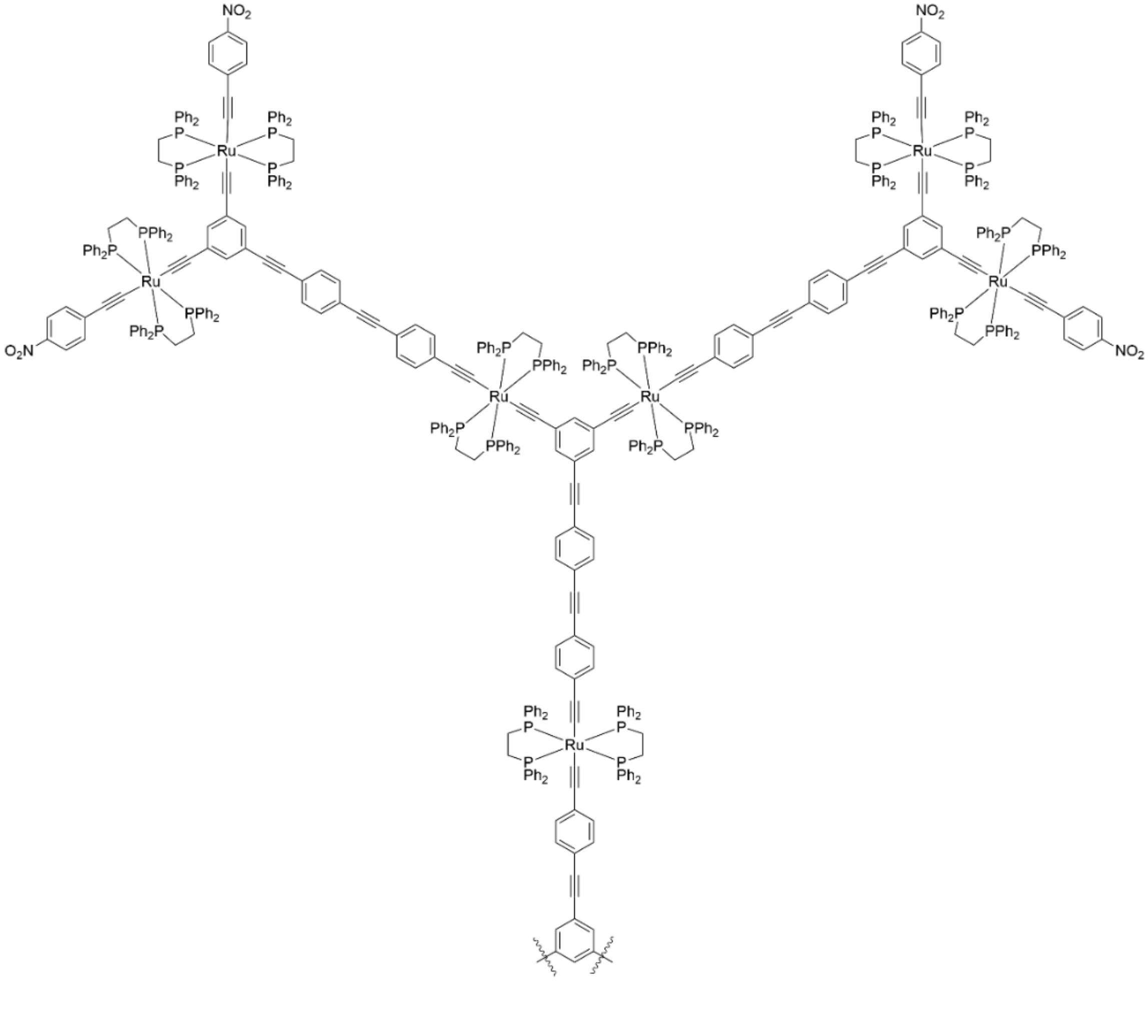

***Synthesis of 1,3,5-{trans-[{1,3-{trans-[(1,3-{trans-[(dppe)$_2$(1,4-O$_2$NC$_6$H$_4$C≡C)Ru(C≡C)]}$_2$-5-C$_6$H$_3$(C≡C-1,4-C$_6$H$_4$C≡C-1,4-C$_6$H$_4$C≡C-1,4-C$_6$H$_4$C≡C)Ru(dppe)$_2$(C≡C)]}$_2$C$_6$H$_3$-5-(C≡C-1,4-C$_6$H$_4$C≡C-1,4-C$_6$H$_4$C≡C)}Ru(dppe)$_2$(C≡C-1,4-C$_6$H$_4$C≡C-1,4-C$_6$H$_4$C≡C)]}$_3$C$_6$H$_3$ (2G$_{22,03,01}$-NO$_2$).***

Compound **1G$_{22,00}$-Cl** (0.050 g, 0.005 mmol) and compound **23** (0.087 g, 0.034 mmol) were added to distilled deoxygenated $CH_2Cl_2$ (50 mL), and $NEt_3$ (1 mL) was added to the solution. The solution was deoxygenated by degassing and backfilling with nitrogen three times. $NaPF_6$ (0.01 g, 0.06 mmol) was added to the flask and the reaction was stirred at room temperature for three days. The crude product was obtained by passing the solution through a Celite pad and removing the solvent *in vacuo*. Further purification was conducted by several successive precipitations from MeOH (2 × 15 mL)) and *n*-pentane (15 mL) to afford **2G$_{22,03,01}$-NO$_2$** as a red powder (0.108 g, 0.030 mmol, 87%). $^1$H NMR (600 MHz, $CDCl_3$, δ): 7.98-7.96 (d, *J* = 8.8 Hz, 24H; $H_{208}$), 7.71-7.70 (m, 96H; $H_{[Ru]\gamma\text{-}3}$ or $H_{[Ru]\gamma\text{-}7}$), 7.65-7.36 (m, 240H; $H_{[Ru]\alpha\text{-}3}$, $H_{[Ru]\alpha\text{-}7}$, $H_{[Ru]\beta\text{-}3}$, $H_{[Ru]\beta\text{-}7}$), 7.61 (m, 24H; $H_{119}$, $H_{120}$), 7.54-7.51 (m, 24H; $H_{113}$, $H_{114}$), 7.29 (m, 12H; $H_{11}$, $H_{20}$), 7.24 (m, 96H; $H_{[Ru]\gamma\text{-}3}$ or $H_{[Ru]\gamma\text{-}7}$), 7.17-7.12 (m, 168H; $H_{[Ru]\alpha\text{-}5}$, $H_{[Ru]\alpha\text{-}9}$, $H_{[Ru]\beta\text{-}5}$, $H_{[Ru]\beta\text{-}9}$, $H_{[Ru]\gamma\text{-}5}$, $H_{[Ru]\gamma\text{-}9}$), 6.82 (m, 6H; $H_{126}$), 6.79, 6.70-6.62 (m, 42H; $H_{125}$, $H_{12}$, $H_{19}$, $H_{107}$, $H_{31}$), 6.53-6.52 (d, *J* = 8.8 Hz, 24H; $H_{207}$), 2.69 (m, 144H; $H_{[Ru]\alpha\text{-}1}$, $H_{[Ru]\beta\text{-}1}$, $H_{[Ru]\gamma\text{-}1}$) ppm; $^{13}$C{$^1$H} NMR (151 MHz, $CDCl_3$, δ): 142.7 ($C_{209}$), 137.5 ($C_{206}$), 137.1, 136.4 ($C_{[Ru]\alpha\text{-}2}$, $C_{[Ru]\alpha\text{-}6}$, $C_{[Ru]\beta\text{-}2}$, $C_{[Ru]\beta\text{-}6}$, $C_{[Ru]\gamma\text{-}2}$, $C_{[Ru]\gamma\text{-}6}$), 134.5, 133.9 (m, $C_{[Ru]\alpha\text{-}3}$, $C_{[Ru]\alpha\text{-}7}$, $C_{[Ru]\beta\text{-}3}$, $C_{[Ru]\beta\text{-}7}$, $C_{[Ru]\gamma\text{-}3}$, $C_{[Ru]\gamma\text{-}7}$), 131.8, 131.0 ($C_{10}$, $C_{12}$, $C_{19}$, $C_{21}$, $C_{31}$, $C_{32}$, $C_{107}$), 130.1 ($C_{207}$), 129.8, 129.2, 128.9 (m, $C_{[Ru]\alpha\text{-}5}$, $C_{[Ru]\alpha\text{-}9}$, $C_{[Ru]\beta\text{-}5}$, $C_{[Ru]\beta\text{-}9}$, $C_{[Ru]\gamma\text{-}5}$, $C_{[Ru]\gamma\text{-}9}$), 128.7, 127.4, 127.2 ($C_{[Ru]\alpha\text{-}4}$, $C_{[Ru]\alpha\text{-}8}$, $C_{[Ru]\beta\text{-}4}$, $C_{[Ru]\beta\text{-}8}$, $C_{[Ru]\gamma\text{-}4}$,$C_{[Ru]\gamma\text{-}8}$), 123.5 ($C_{208}$), 118.7, 118.5 ($C_{202}$, $C_{205}$), 31.6 ($C_{[Ru]\alpha\text{-}1}$, $C_{[Ru]\beta\text{-}1}$, $C_{[Ru]\gamma\text{-}1}$) ppm; $^{31}$P{$^1$H} NMR (162 MHz, $CDCl_3$, δ): 53.6 (24P; $P_{[Ru]\beta}$), 53.3 (84P; $P_{[Ru]\alpha}$, $P_{[Ru]\gamma}$) ppm; IR: $\nu$ = 2043 cm$^{-1}$ (C≡C); UV-vis ($CH_2Cl_2$, $\nu_{max}$ in cm$^{-1}$, [ε] in 10$^3$ M$^{-1}$ cm$^{-1}$): 23 250 [615.7], 29 400 [1018.8]; Anal. calcd. for $C_{1548}H_{1206}N_{12}O_{24}P_{84}Ru_{21}$: C, 74.12; H, 4.85; N, 0.67%; found: C, 73.96; H, 4.86; N, 0.71%.

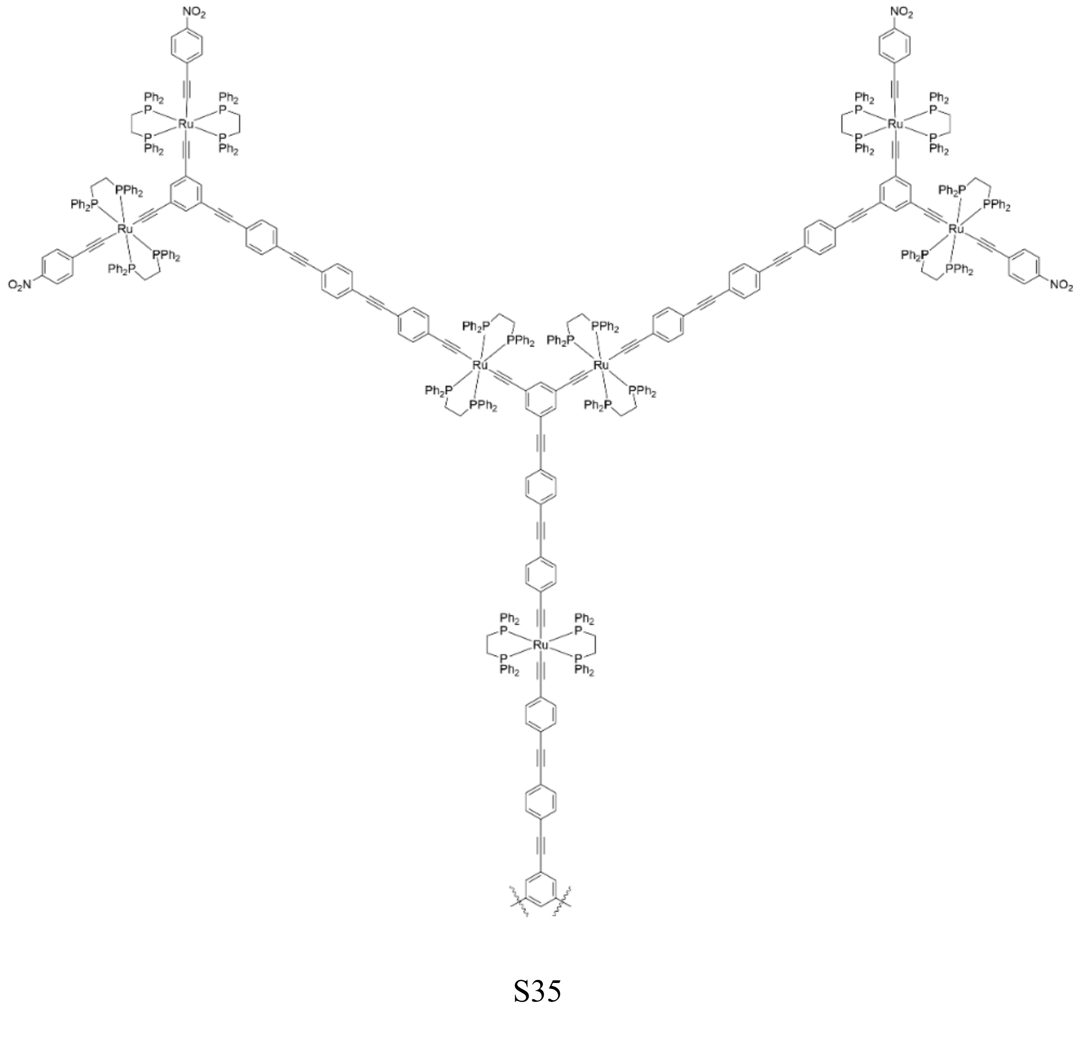

***Synthesis of 1,3,5-{trans-[1,3-{trans-[1,3-{trans-[1,3-{trans-[(dppe)$_2$(1,4-O$_2$NC$_6$H$_4$C≡C)Ru(C≡C)]}$_2$C$_6$H$_3$-5-(C≡C-1,4-C$_6$H$_4$C≡C-1,4-C$_6$H$_4$C≡C)Ru(dppe)$_2$(C≡C)]}$_2$C$_6$H$_3$-5-(C≡C-1,4-C$_6$H$_4$C≡C-1,4-C$_6$H$_4$C≡C-1,4-C$_6$H$_4$C≡C)Ru(dppe)$_2$(C≡C)]}$_2$C$_6$H$_3$-5-(C≡C-1,4-C$_6$H$_4$C≡C-1,4-C$_6$H$_4$C≡C)Ru(dppe)$_2$(C≡C-1,4-C$_6$H$_4$C≡C-1,4-C$_6$H$_4$C≡C)]}$_3$C$_6$H$_3$ (3G$_{22,03,02,01}$-NO$_2$).***

Compound **1G$_{22,00}$-Cl** (0.025 g, 0.0025 mmol) and **27** (0.114 g, 0.016 mmol) were added to distilled, deoxygenated $CH_2Cl_2$ (60 mL), and $NEt_3$ (1 mL) was added to the solution. $NaPF_6$ (0.104 g, 0.62 mmol) was added to the flask. The reaction mixture was stirred for 16 h at room temperature. The crude product was obtained by passing the mixture through a Celite pad and removing the solvent *in vacuo*. Further purification was conducted by several successive precipitations from MeOH (2 × 15 mL) and *n*-pentane (15 mL) to afford **3G$_{22,03,02,01}$-NO$_2$** as a red powder (0.024 g, 0.0021 mmol, 84%). $^1$H NMR (700 MHz, $CDCl_3$, δ): 7.98 (d, $J$ = 8.4 Hz, 48H; $H_{308}$), 7.71-7.51 (m, 720H; $H_{[Ru]\alpha\text{-}3}$, $H_{[Ru]\alpha\text{-}7}$, $H_{[Ru]\beta\text{-}3}$, $H_{[Ru]\beta\text{-}7}$, $H_{[Ru]\gamma\text{-}3}$, $H_{[Ru]\gamma\text{-}7}$, $H_{[Ru]\delta\text{-}3}$, $H_{[Ru]\delta\text{-}7}$), 7.37, 7.25, 7.18-7.12 (m, 360H; $H_{[Ru]\alpha\text{-}5}$, $H_{[Ru]\alpha\text{-}9}$, $H_{[Ru]\beta\text{-}5}$, $H_{[Ru]\beta\text{-}9}$, $H_{[Ru]\beta\text{-}9}$, $H_{[Ru]\gamma\text{-}5}$, $H_{[Ru]\gamma\text{-}9}$, $H_{[Ru]\delta\text{-}5}$, $H_{[Ru]\delta\text{-}9}$), 6.99-6.93 (m, 720H; $H_{[Ru]\alpha\text{-}4}$, $H_{[Ru]\alpha\text{-}8}$, $H_{[Ru]\beta\text{-}4}$, $H_{[Ru]\beta\text{-}8}$, $H_{[Ru]\gamma\text{-}4}$, $H_{[Ru]\gamma\text{-}8}$, $H_{[Ru]\delta\text{-}4}$, $H_{[Ru]\delta\text{-}8}$), 6.83-6.62 (m, 147H; $H_{12}$, $H_{19}$, $H_{31}$, $H_{32}$, $H_{125}$, $H_{126}$, $H_{219}$, $H_{220}$), 6.54-6.53 (d, $J$ = 8.4 Hz, 48H; $H_{307}$), 2.70 (m, 360H; $H_{[Ru]\alpha\text{-}1}$, $H_{[Ru]\beta\text{-}1}$, $H_{[Ru]\gamma\text{-}1}$, $H_{[Ru]\delta\text{-}1}$) ppm; $^{13}C\{^1H\}$ NMR (151 MHz, $CDCl_3$, δ): 142.7 ($C_{309}$),137.5 ($C_{306}$), 137.1, 136.4 (m, $C_{[Ru]\alpha\text{-}2}$, $C_{[Ru]\alpha\text{-}6}$, $C_{[Ru]\beta\text{-}2}$, $C_{[Ru]\beta\text{-}6}$, $C_{[Ru]\gamma\text{-}2}$, $C_{[Ru]\gamma\text{-}6}$, $C_{[Ru]\delta\text{-}2}$, $C_{[Ru]\delta\text{-}6}$), 134.5, 133.9 (m, $C_{[Ru]\alpha\text{-}3}$, $C_{[Ru]\alpha\text{-}7}$, $C_{[Ru]\beta\text{-}3}$, $C_{[Ru]\beta\text{-}7}$, $C_{[Ru]\gamma\text{-}3}$, $C_{[Ru]\gamma\text{-}7}$, $C_{[Ru]\delta\text{-}3}$, $C_{[Ru]\delta\text{-}7}$), 131.8, 131.6, 131.0, 130.11, 130.06 ($C_{307}$), 129.9, 129.2-128.9 (m, $C_{[Ru]\alpha\text{-}5}$, $C_{[Ru]\alpha\text{-}9}$, $C_{[Ru]\beta\text{-}5}$, $C_{[Ru]\beta\text{-}9}$, $C_{[Ru]\gamma\text{-}5}$, $C_{[Ru]\gamma\text{-}9}$, $C_{[Ru]\delta\text{-}5}$, $C_{[Ru]\delta\text{-}9}$), 127.4, 127.2 (m, $C_{[Ru]\alpha\text{-}4}$, $C_{[Ru]\alpha\text{-}8}$, $C_{[Ru]\beta\text{-}4}$, $C_{[Ru]\beta\text{-}8}$, $C_{[Ru]\gamma\text{-}4}$, $C_{[Ru]\gamma\text{-}8}$, $C_{[Ru]\delta\text{-}4}$, $C_{[Ru]\delta\text{-}8}$), 123.8 ($C_{308}$), 121.7, 118.7, 118.5, 116.5, 92.8, 89.5, 88.0, 31.6 (m, $C_{[Ru]\alpha\text{-}1}$, $C_{[Ru]\beta\text{-}1}$, $C_{[Ru]\gamma\text{-}1}$, $C_{[Ru]\delta\text{-}1}$).ppm; $^{31}P\{^1H\}$ NMR (162 MHz, $CDCl_3$, δ): 53.6 (72P; $P_{[Ru]\alpha}$, $P_{[Ru]\gamma}$), 53.3 (108P; $P_{[Ru]\alpha}$, $P_{[Ru]\delta}$) ppm; IR: $v$ = 2044 cm$^{-1}$ (C≡C); UV-vis ($CH_2Cl_2$, $v_{max}$ in cm$^{-1}$, [ε] in 10$^3$ M$^{-1}$ cm$^{-1}$): 23 400 [816.4], 29 400 [1283.2]; Anal. calcd. for $C_{3228}H_{2538}N_{24}O_{48}P_{180}Ru_{45}$: C, 73.77; H, 4.87; N, 0.64%; found: C, 73.67; H, 4.91; N, 0.65%.

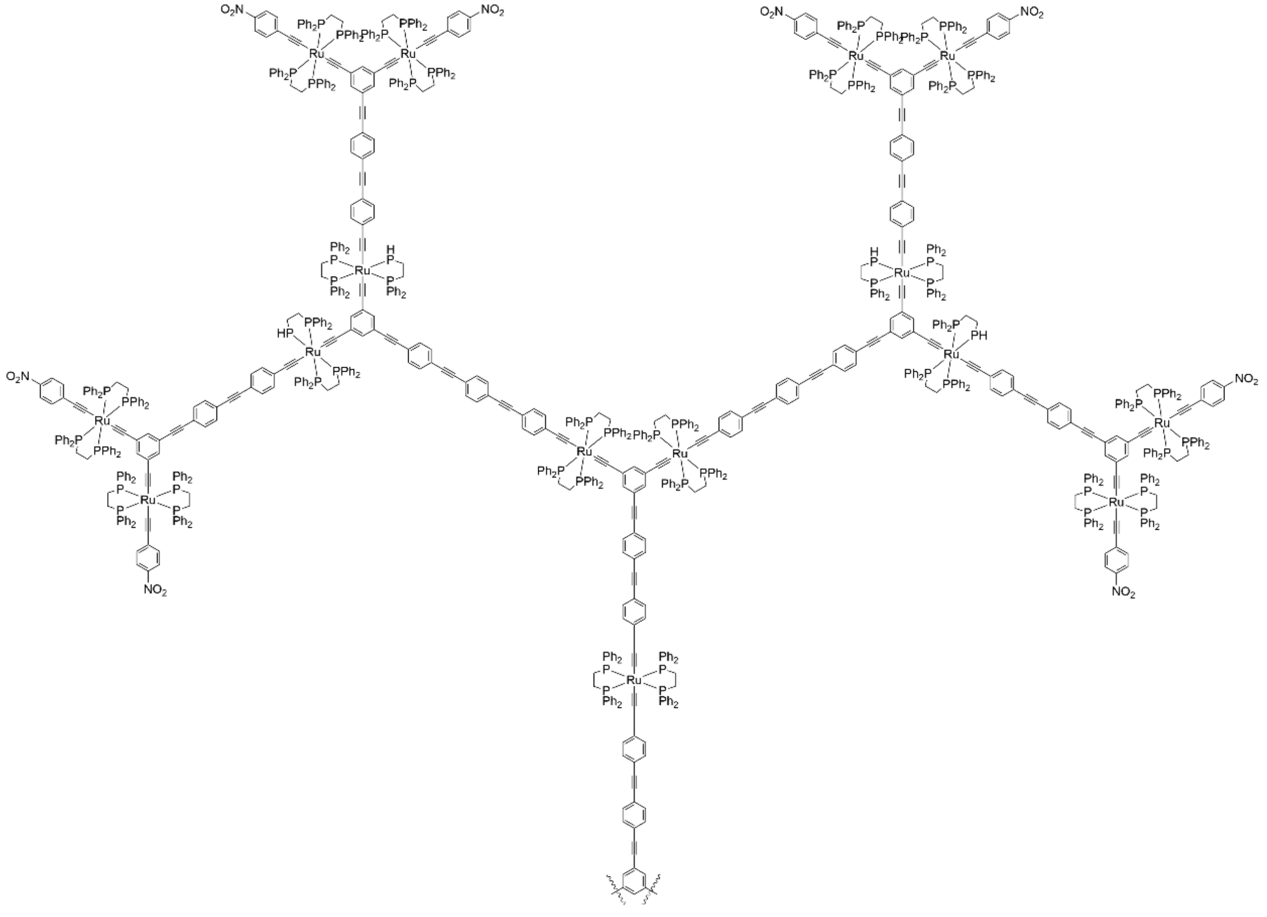

***Synthesis of 1,3,5-{trans-[1,3-{trans-[1,3-{trans-[(dppe)$_2$(1,4-IC$_6$H$_4$C≡C-1,4-C$_6$H$_4$C≡C)Ru(C≡C)]}$_2$C$_6$H$_3$-5-(C≡C-1,4-C$_6$H$_4$C≡C-2,6-Et$_2$-1,4-C$_6$H$_2$C≡C-1,4-C$_6$H$_4$C≡C)Ru(dppe)$_2$(C≡C)]}$_2$C$_6$H$_3$-5-(C≡C-1,4-C$_6$H$_4$C≡C-1,4-C$_6$H$_4$C≡C)Ru(dppe)$_2$(C≡C-1,4-C$_6$H$_4$C≡C-1,4-C$_6$H$_4$C≡C)]}$_3$C$_6$H$_3$ (36)***.

Compound **19** (0.106 g, 0.036 mmol) and **1G$_{22,00}$-Cl** (0.040 g, 0.0040 mmol) were added to distilled deoxygenated CH$_2$Cl$_2$ (60 mL), and NEt$_3$ (1 mL) was added. NaPF$_6$ (0.024 g, 0.144 mmol) was added to the flask and the reaction was heated at reflux for 24 h. The crude product was obtained by passing the reaction mixture through a Celite pad and removing the solvent *in vacuo*. Further purification was conducted by several successive precipitations from MeOH (2 × 15 mL) and *n*-pentane (15 mL), to afford a yellow powder identified as **36** (0.100 g, 0.033 mmol, 91%). $^{1}$H NMR (600 MHz, CDCl$_3$, δ): 7.71-7.69 (m, 24H; H$_{214}$), 7.66-7.36 (m, 336H; H$_{[Ru]α-3}$, H$_{[Ru]α-7}$, H$_{[Ru]β-3}$, H$_{[Ru]β-7}$, H$_{[Ru]γ-3}$, H$_{[Ru]γ-7}$), 7.31-7.28 (m, 36H; H$_{11}$, H$_{20}$, H$_{108}$, H$_{113}$, H$_{208}$), 7.16-7.11 (m, 168H; H$_{[Ru]α-5}$, H$_{[Ru]α-9}$, H$_{[Ru]β-5}$, H$_{[Ru]β-9}$, H$_{[Ru]γ-5}$, H$_{[Ru]γ-9}$), 6.97-6.93 (m, 336H; H$_{[Ru]α-4}$, H$_{[Ru]α-8}$, H$_{[Ru]β-4}$, H$_{[Ru]β-8}$, H$_{[Ru]γ-4}$, H$_{[Ru]γ-8}$), 6.80-6.61 (60H; H$_{12}$, H$_{19}$, H$_{32}$, H$_{107}$, H$_{31}$, H$_{125}$, H$_{126}$, H$_{207}$), 2.95 (m, 24H; H$_{s1}$), 2.71 (m, 144H; H$_{[Ru]α-1}$, H$_{[Ru]β-1}$, H$_{[Ru]γ-1}$), 1.38-1.37 (m, 36H; H$_{s2}$) ppm; $^{13}$C{$^{1}$H} NMR (151 MHz, CDCl$_3$, δ): 146.6 (C$_{114}$), 137.8, 137.6 (C$_{214}$), 137.3, 136.9 (m, C$_{[Ru]α-2}$, C$_{[Ru]α-6}$, C$_{[Ru]β-2}$, C$_{[Ru]β-6}$, C$_{[Ru]γ-2}$, C$_{[Ru]γ-6}$), 134.5, 134.2 (m, C$_{[Ru]α-3}$, C$_{[Ru]α-7}$, C$_{[Ru]β-3}$, C$_{[Ru]β-7}$, C$_{[Ru]γ-3}$, C$_{[Ru]γ-7}$), 133.2, 133.1 (C$_{207}$), 131.8, 131.5 (C$_{119}$, C$_{208}$), 131.0 (C$_{213}$), 130.1 (C$_{125}$), 129.8 (C$_{126}$), 129.0, 128.7 (m, C$_{[Ru]α-5}$, C$_{[Ru]α-9}$, C$_{[Ru]β-5}$, C$_{[Ru]β-9}$, C$_{[Ru]γ-5}$, C$_{[Ru]γ-9}$), 128.5 (C$_{113}$), 127.3, 127.2 (C$_{[Ru]α-4}$, C$_{[Ru]α-8}$, C$_{[Ru]β-4}$, C$_{[Ru]β-8}$, C$_{[Ru]γ-4}$, C$_{[Ru]γ-8}$), 124.3, 123.6 (C$_{209}$), 122.7, 117.6, 117.0 (C$_{124}$, C$_{201}$),116.4 (C$_{202}$, C$_{205}$), 94.6, 93.5 (C$_{210}$), 92.4 (C$_{111}$), 91.5, 90.6, 89.4, 88.7 (C$_{9}$, C$_{22}$), 31.7 (C$_{[Ru]α-1}$, C$_{[Ru]β-1}$, C$_{[Ru]γ-1}$), 28.2 (C$_{s1}$), 14.9 (C$_{s2}$) ppm; $^{31}$P{$^{1}$H} NMR (162 MHz, CDCl$_3$, δ): 53.6 (s, P$_{[Ru]α}$,), 53.4 (s, P$_{[Ru]β}$, P$_{[Ru]γ}$) ppm; IR: $\nu$ = 2045 cm$^{-1}$ (C≡C); UV-vis (CH$_2$Cl$_2$, $\nu_{max}$ in cm$^{-1}$, [ε] in 10$^{3}$ M$^{-1}$ cm$^{-1}$): 27 450 [1240.0]; Anal. calcd. for C$_{1668}$H$_{1302}$I$_{12}$P$_{84}$Ru$_{21}$: C, 72.60; H, 4.76%; found: C, 72.56; H, 4.64%.

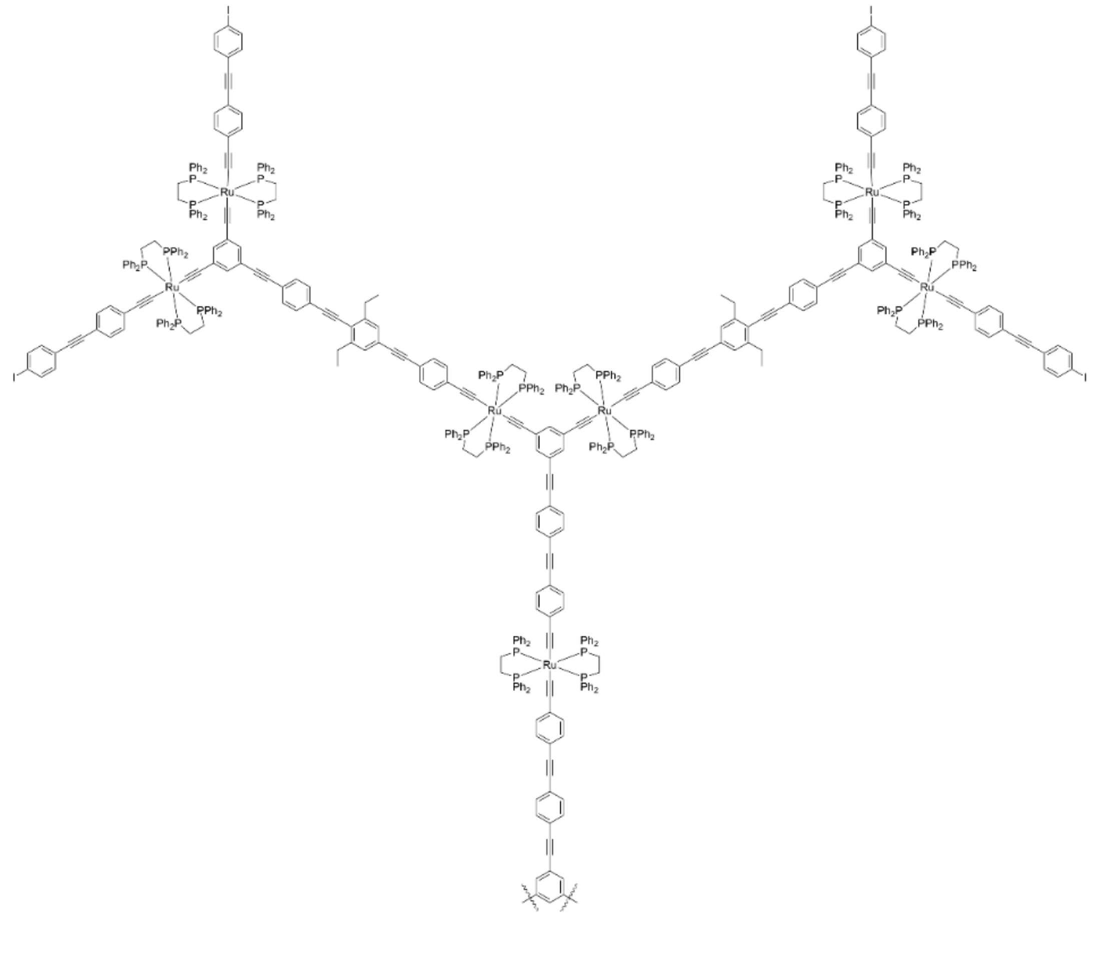

***Synthesis of 1,3,5-{trans-[1,3-{trans-[1,3-{trans-[1,3-{trans-[(dppe)$_2$(Cl)Ru(C≡C)]}$_2$C$_6$H$_3$-5-(C≡C-1,4-C$_6$H$_4$C≡C-1,4-C$_6$H$_4$C≡C)Ru(dppe)$_2$(C≡C)]}$_2$C$_6$H$_3$-5-(C≡C-1,4-C$_6$H$_4$C≡C-2,6-Et$_2$-1,4-C$_6$H$_2$C≡C-1,4-C$_6$H$_4$C≡C)Ru(dppe)$_2$(C≡C)]}$_2$C$_6$H$_3$-5-(C≡C-1,4-C$_6$H$_4$C≡C-1,4-C$_6$H$_4$C≡C)Ru(dppe)$_2$(C≡C-1,4-C$_6$H$_4$C≡C-1,4-C$_6$H$_4$C≡C)]}$_3$C$_6$H$_3$ (3G$_{22,03,02,00}$-Cl).***

Compound **36** (0.050 g, 0.0018 mmol) and 1,3-{*trans*-[(dppe)$_2$(Cl)Ru(C≡C)]}$_2$-5-(HC≡C)C$_6$H$_3$ (**10**) (0.049 g, 0.024 mmol) were added to distilled deoxygenated $CH_2Cl_2$ (100 mL) and $NEt_3$ (20 mL). The solution was deoxygenated by degassing and backfilling with nitrogen three times. Catalytic amounts of Pd(PPh$_3$)$_4$ and [Cu(NCMe)$_4$]PF$_6$ were added to the solution. The reaction was stirred at room temperature for three days. The crude product was obtained by pouring the reaction mixture into stirring MeOH and collected by filtration with a sintered funnel. Further purification was conducted by several successive precipitations from MeOH (2 × 25 mL) and Et$_2$O (25 mL) and washing several times with *n*-pentane (3 × 10 mL), to afford a yellow powder identified as compound **3G$_{22,03,02,00}$-Cl** (0.045 g, 0.0010 mmol, 50%). $^1$H NMR (400 MHz, CDCl$_3$, δ): 7.65-7.36 (m, 720H; $H_{[Ru]\alpha\text{-}3}$, $H_{[Ru]\alpha\text{-}7}$, $H_{[Ru]\beta\text{-}3}$, $H_{[Ru]\beta\text{-}7}$, $H_{[Ru]\gamma\text{-}3}$, $H_{[Ru]\gamma\text{-}7}$, $H_{[Ru]\delta\text{-}3}$, $H_{[Ru]\delta\text{-}7}$), 7.17-7.16 (m, 360H; $H_{[Ru]\alpha\text{-}5}$, $H_{[Ru]\alpha\text{-}9}$, $H_{[Ru]\beta\text{-}5}$, $H_{[Ru]\beta\text{-}9}$, $H_{[Ru]\beta\text{-}9}$, $H_{[Ru]\gamma\text{-}5}$, $H_{[Ru]\gamma\text{-}9}$, $H_{[Ru]\delta\text{-}5}$, $H_{[Ru]\delta\text{-}9}$), 7.11-6.97 (m, 720H; $H_{[Ru]\alpha\text{-}4}$, $H_{[Ru]\alpha\text{-}8}$, $H_{[Ru]\beta\text{-}4}$, $H_{[Ru]\beta\text{-}8}$, $H_{[Ru]\gamma\text{-}4}$, $H_{[Ru]\gamma\text{-}8}$, $H_{[Ru]\delta\text{-}4}$, $H_{[Ru]\delta\text{-}8}$), 6.95-6.64 (m, 12H; $H_{220}$), 6.60-6.44 (m, 24H; $H_{219}$), 2.93 (m, 24H; $H_{s1}$), 2.70 (m, 168H; $H_{[Ru]\alpha\text{-}1}$, $H_{[Ru]\beta\text{-}1}$, $H_{[Ru]\gamma\text{-}1}$, $H_{[Ru]\delta\text{-}1}$), 1.36 (m, 36H; $H_{s2}$) ppm; $^{31}$P{$^1$H} NMR (162 MHz, CDCl$_3$, δ): 53.6 (84P; $P_{[Ru]\alpha}$, $P_{[Ru]\beta}$, $P_{[Ru]\gamma}$), 49.9 (96P; $P_{[Ru]\delta}$) ppm; IR: $\nu$ = 2049 cm $^{-1}$ (C≡C); UV-vis (CH$_2$Cl$_2$, $\nu_{max}$ in cm$^{-1}$, [ε] in 10$^3$ M$^{-1}$ cm$^{-1}$): 23 900 [1089.9], 29 150 [1754.6]; Anal. calcd. for $C_{3060}H_{2490}Cl_{24}P_{180}Ru_{45}$: C, 73.16; H, 5.00%; found: C, 73.37; H, 5.06%.

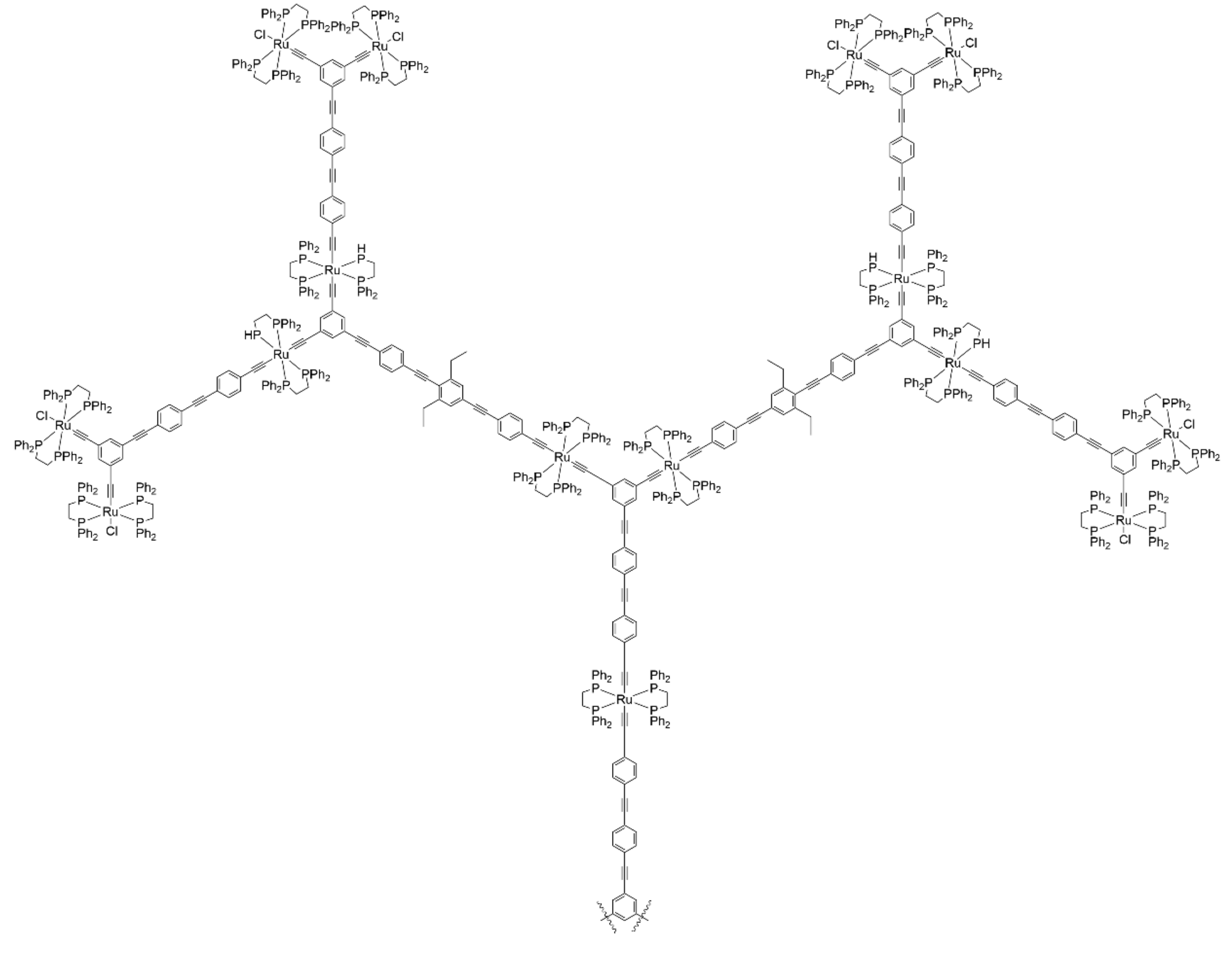

***Synthesis of trans-[Ru(C≡CPh)(C≡C-1,4-$C_6H_4$C≡C-1,4-$C_6H_4$C≡CPh)(dppe)$_2$] (1-M-3(dppe)).***

*trans*-[Ru(C≡CPh)Cl(dppe)$_2$] (0.250 g, 0.242 mmol) and 1,4-HC≡C$C_6H_4$-1,4-$C_6H_4$C≡CPh (0.088 mL, 0.290 mmol) were added to freshly distilled $CH_2Cl_2$ (60 mL) and triethylamine (1 mL) was added to the solution. $NaPF_6$ (0.122 g, 0.725 mmol) was added to the flask and the reaction was stirred at room temperature overnight. The crude product was obtained by passing the reaction mixture through a Celite pad and removing the solvent *in vacuo*. Further purification was conducted by precipitation from MeOH (2 × 20 mL) and *n*-pentane (2 × 20 mL), to afford compound **1-M-3(dppe)** as a yellow powder (0.228 g, 0.09 mmol, 90%). $^1$H NMR (700 MHz, $C_6D_6$, δ): 7.78-7.59 (m, 16H; $H_{[Ru]-3}$, $H_{[Ru]-7}$), 7.57, 7.50-7.49 (m, 2H; $H_{22}$), 7.42-7.37 (m, 4H; $H_{16}$, $H_{17}$), 7.27 (m, 2H; $H_2$), 7.09-7.08 (m, 1H; $H_1$), 7.03-7.02 (m, 3H; $H_{23}$, $H_{24}$), 7.00-6.99 (m, 8H; $H_{[Ru]-5}$, $H_{[Ru]-9}$), 6.97-6.96 (m, 2H; $H_3$), 6.94-6.91 (m, 16H; $H_{[Ru]-4}$, $H_{[Ru]-8}$), 2.59 (m, 8H; $H_{[Ru]-1}$) ppm; $^{13}$C{$^1$H} NMR (176 MHz, $C_6D_6$, δ): 137.9 ($C_{[Ru]-2}$ or $C_{[Ru]-6}$), 137.6 ($C_{[Ru]-6}$ or $C_{[Ru]-2}$), 134.9 ($C_{[Ru]-3}$ or $C_{[Ru]-7}$), 134.7 9 ($C_{[Ru]-7}$ or $C_{[Ru]-3}$), 132.0, 131.8, 131.6 ($C_{16}$, $C_{17}$), 131.3 ($C_{22}$), 130.6, 129.1 ($C_{[Ru]-5}$ or $C_{[Ru]-9}$), 129.0 ($C_{[Ru]-5}$ or $C_{[Ru]-9}$), 128.7, 128.4, 127.5 ($C_{[Ru]-4}$, $C_{[Ru]-8}$), 124.6, 124.0, 123.8, 123.2 ($C_4$, $C_9$, $C_{12}$), 118.1, 118.0, 117.8 ($C_5$, $C_6$, $C_7$, $C_8$), 93.7, 91.8, 90.2 ($C_{13}$, $C_{14}$, $C_{19}$, $C_{20}$), 31.9 ($C_{[Ru]-1}$) ppm; $^{31}$P{$^1$H} NMR (162 MHz, $C_6D_6$, δ): 53.6 (s, $P_{[Ru]}$,) ppm; IR: $\nu$ = 2055 cm$^{-1}$ (C≡C); UV-vis ($CH_2Cl_2$, $\nu_{max}$ in cm$^{-1}$, [ε] in $10^3$ M$^{-1}$ cm$^{-1}$): 24 100 [407.6], 31 060 [559.4]; HR ESI MS *m/z*: calcd. for $C_{84}H_{67}I_{12}P_4Ru$: 1301.3260; found: 1301.3225 ([M+H]$^+$); Anal. calcd. for $C_{84}H_{66}I_{12}P_4Ru$: C, 77.58; H, 5.12%; found: C, 72.13; H, 5.12%.

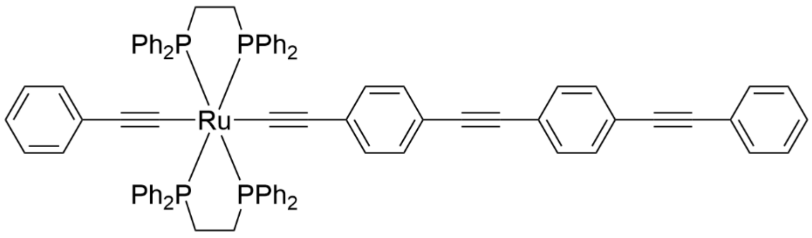

## NMR spectra

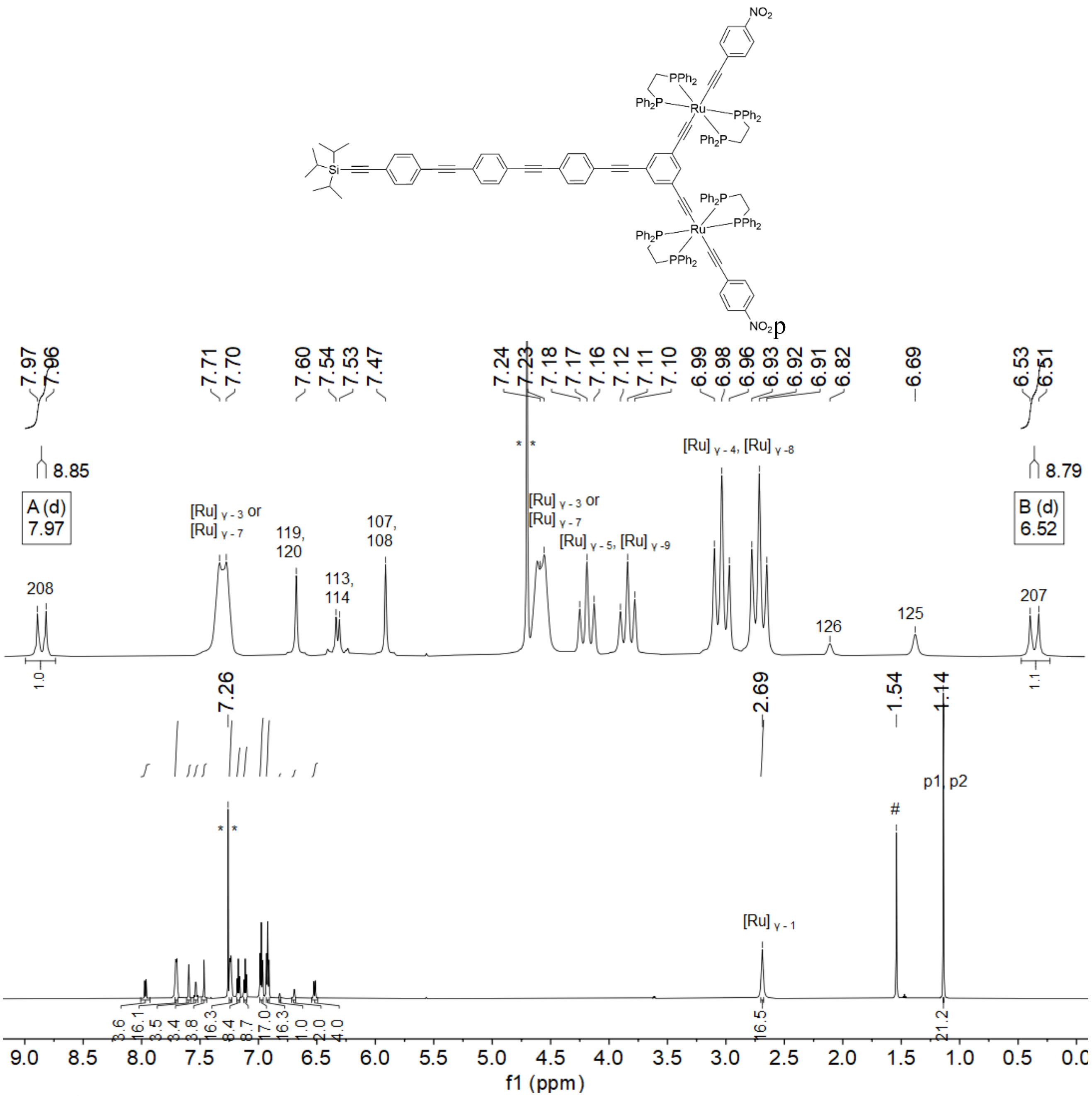

**Fig. S2** $^{1}$H NMR spectrum of **22** recorded in $CDCl_3$ at 700 MHz. The peak marked as ** corresponds to the $CHCl_3$ signal. The peak marked as # corresponds to residual water.

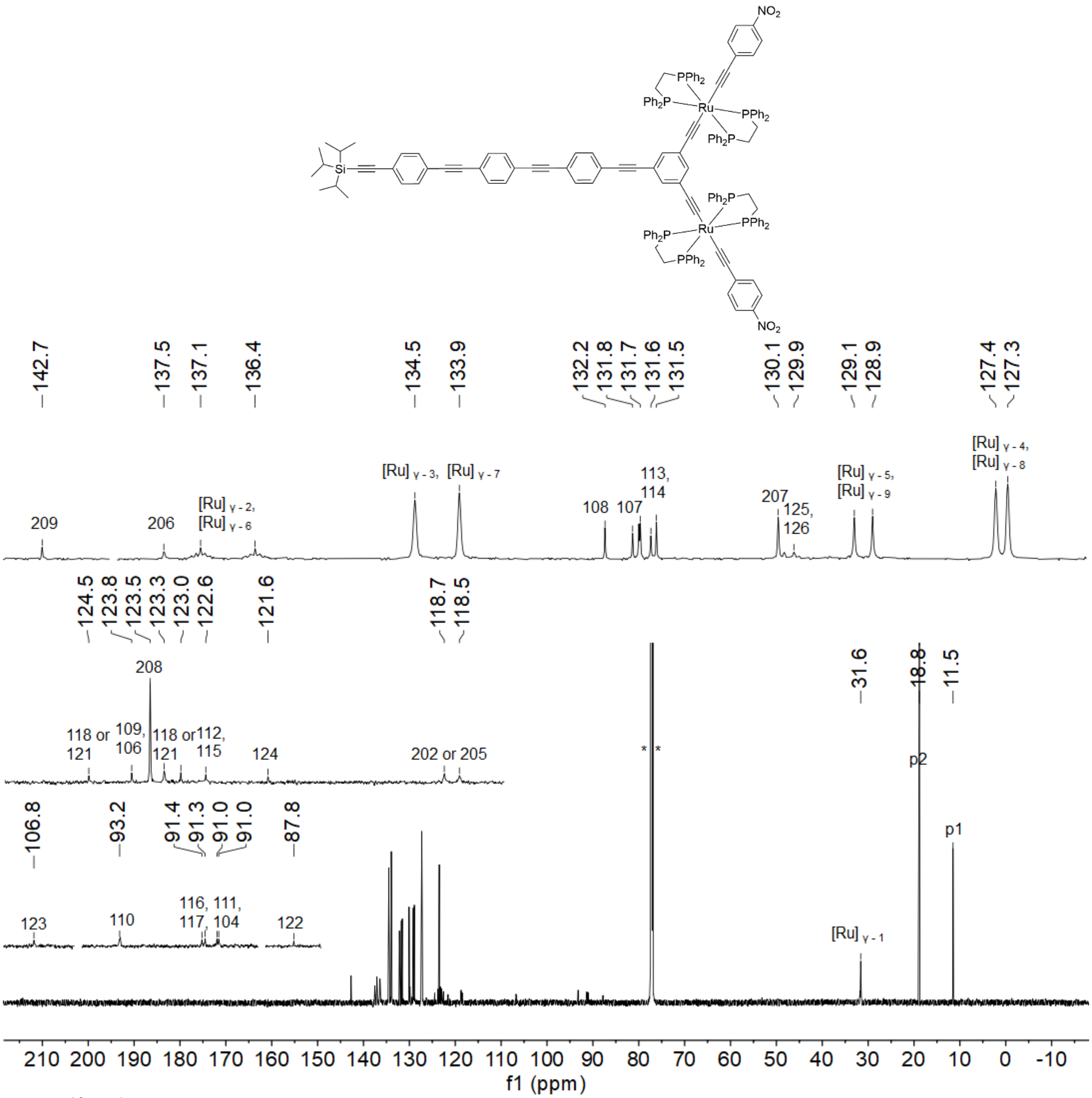

**Fig. S3** $^{13}C\{^1H\}$ NMR spectrum of **22** recorded in $CDCl_3$ at 176 MHz. The peak marked as ** corresponds to $CDCl_3$.

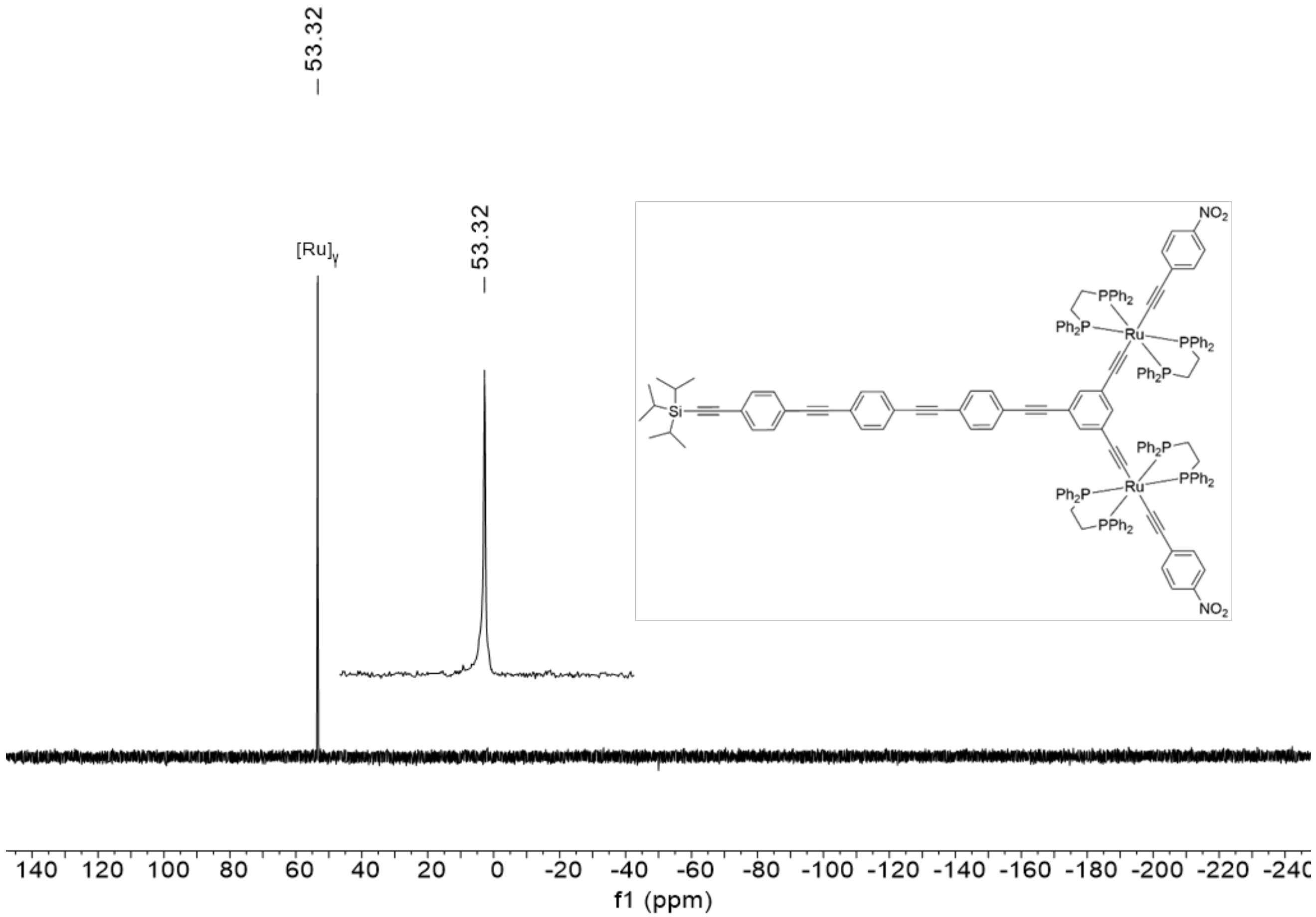


**Fig. S4** $^{31}P\{^{1}H\}$ NMR spectrum of **22** recorded in $CDCl_3$ at 162 MHz.

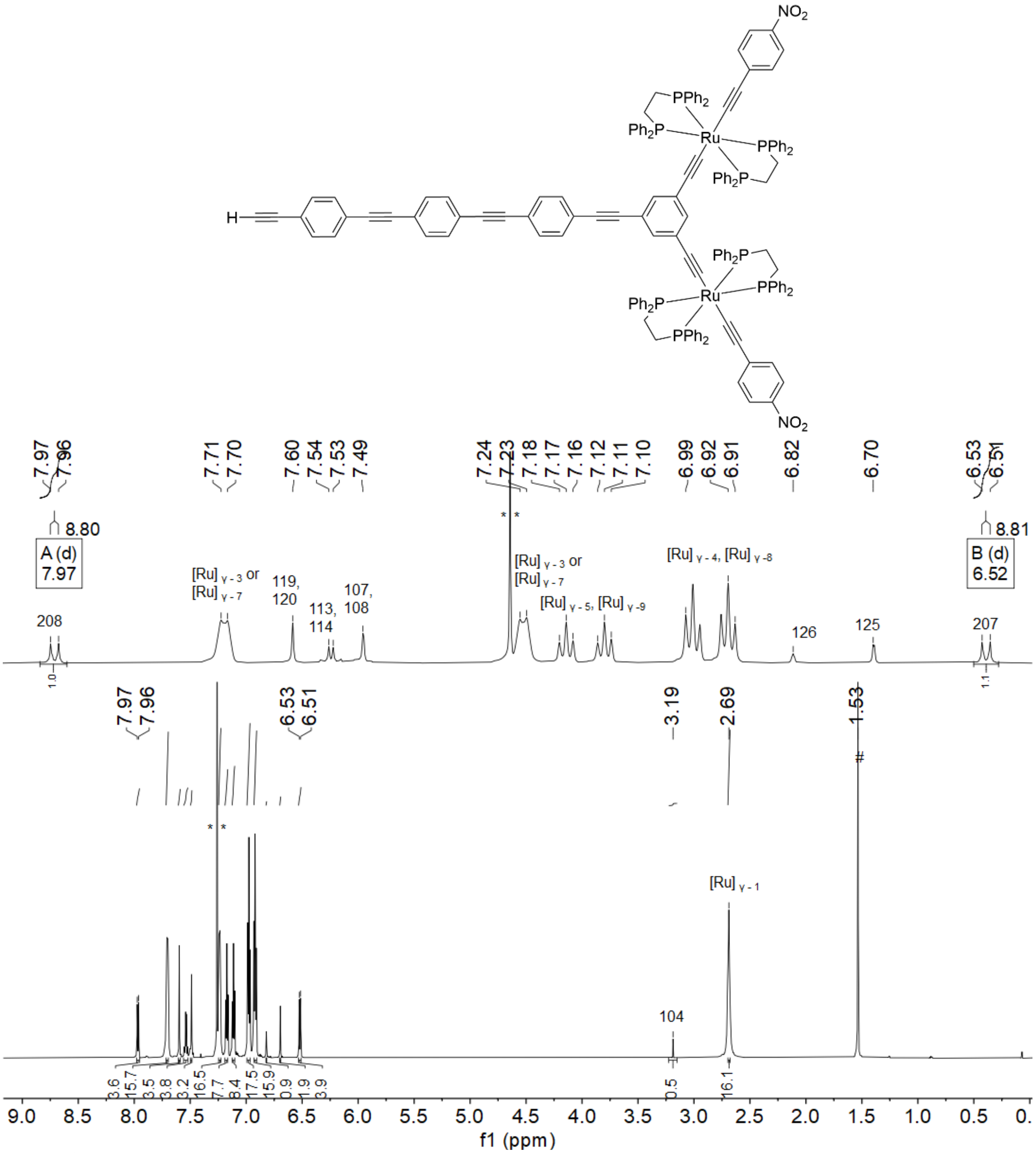


**Fig. S5** $^{1}$H NMR spectrum of **23** recorded in $CDCl_3$ at 700 MHz. The peak marked as ** corresponds to $CHCl_3$. The peak marked as # corresponds to residual water.

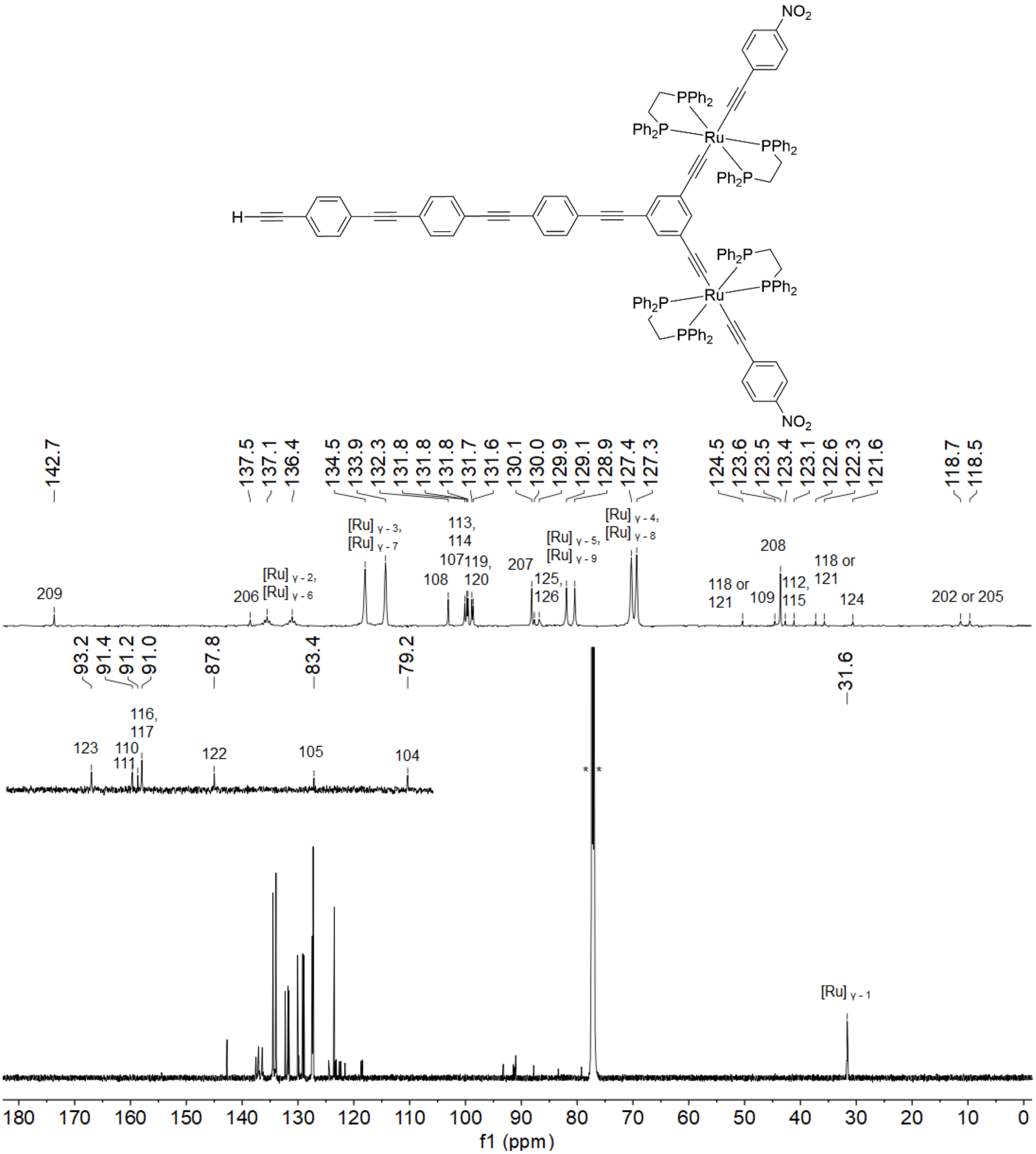


**Fig. S6** $^{13}C\{^{1}H\}$ NMR spectrum of **23** recorded in $CDCl_3$ at 151 MHz. The peak marked as ** corresponds to $CDCl_3$.

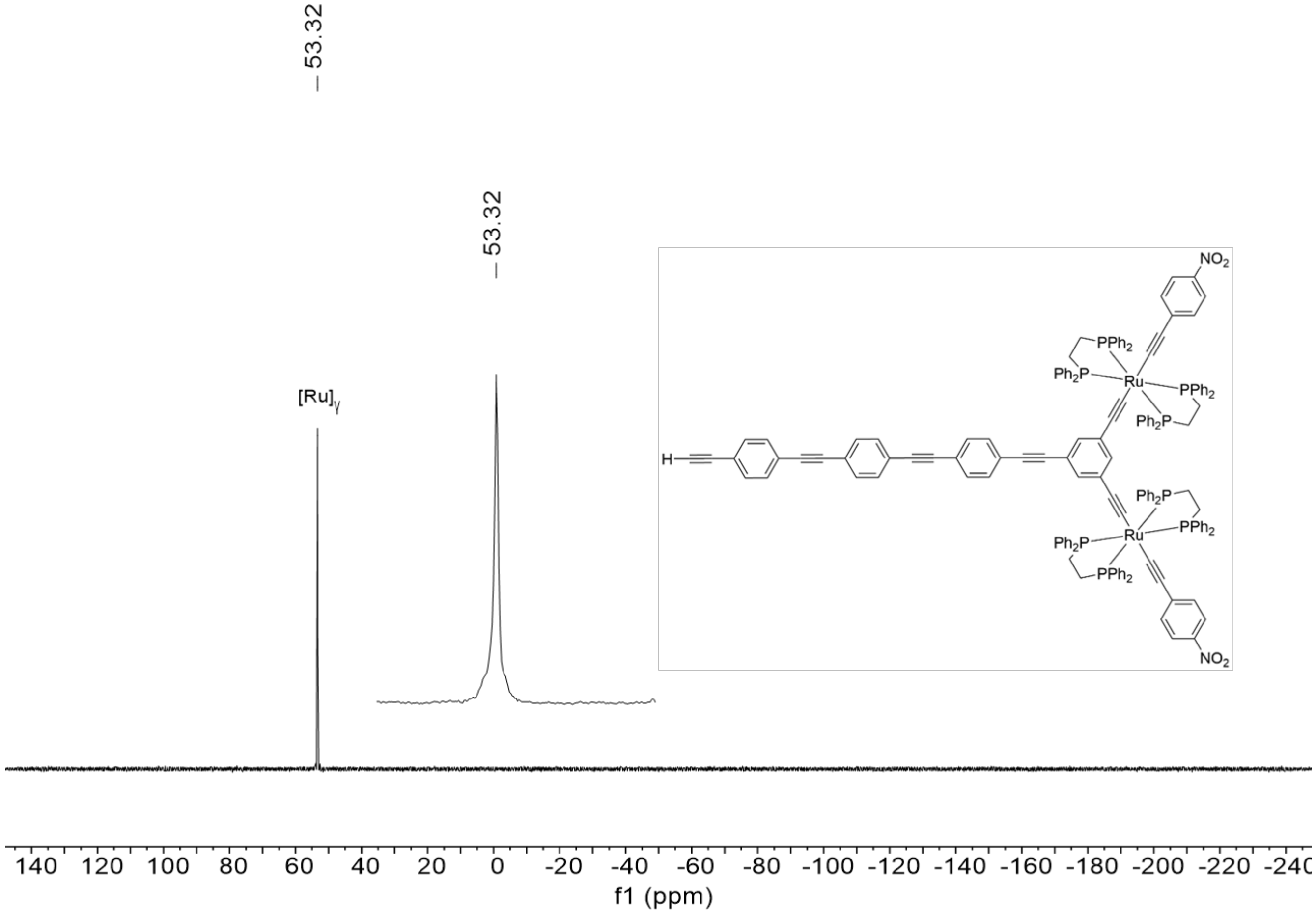


**Fig. S7** $^{31}P\{^{1}H\}$ NMR spectrum of **23** recorded in $CDCl_3$ at 162 MHz.

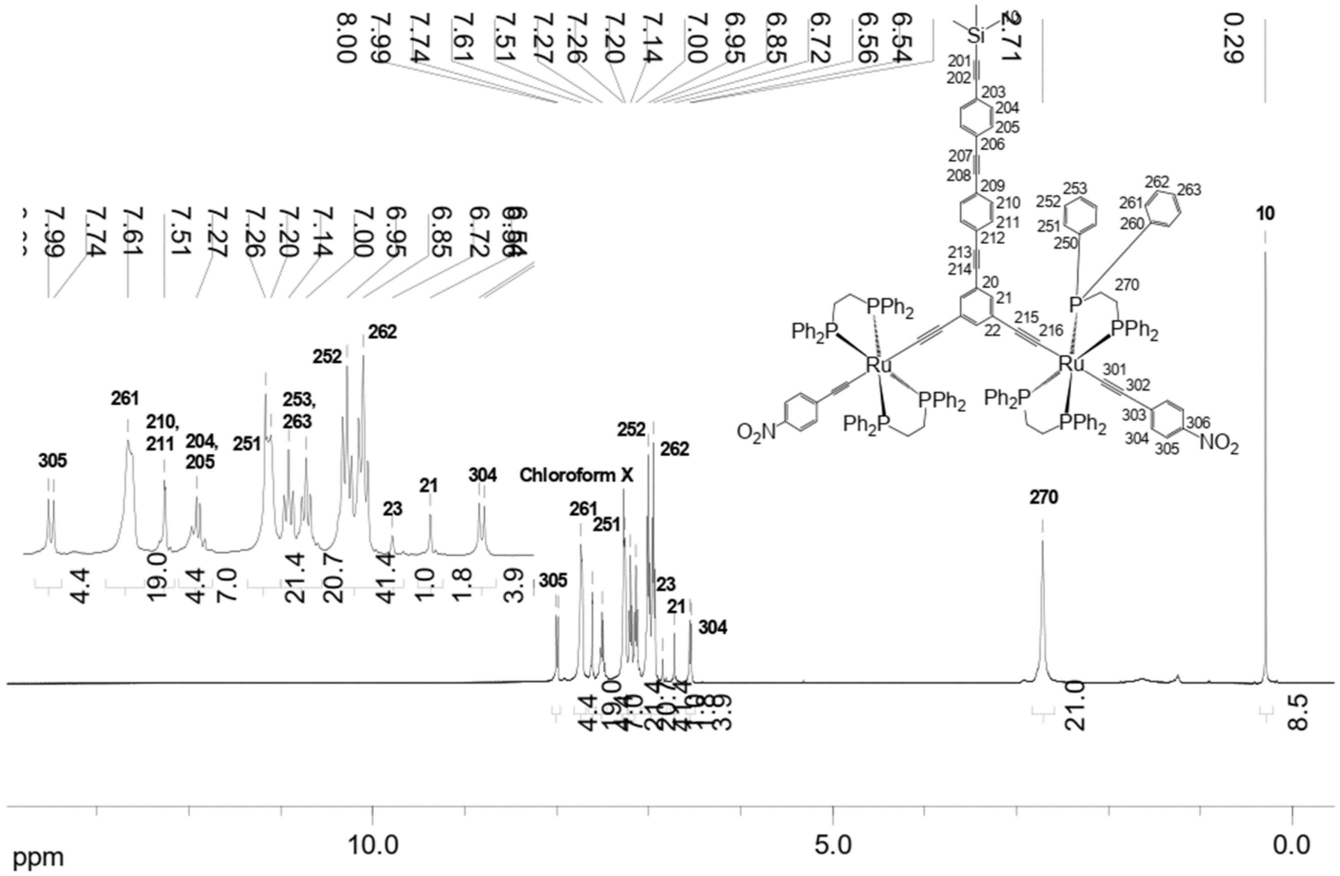

**Fig. S8** $^{1}H$ NMR spectrum of **14** recorded in $CDCl_3$ at 500 MHz.

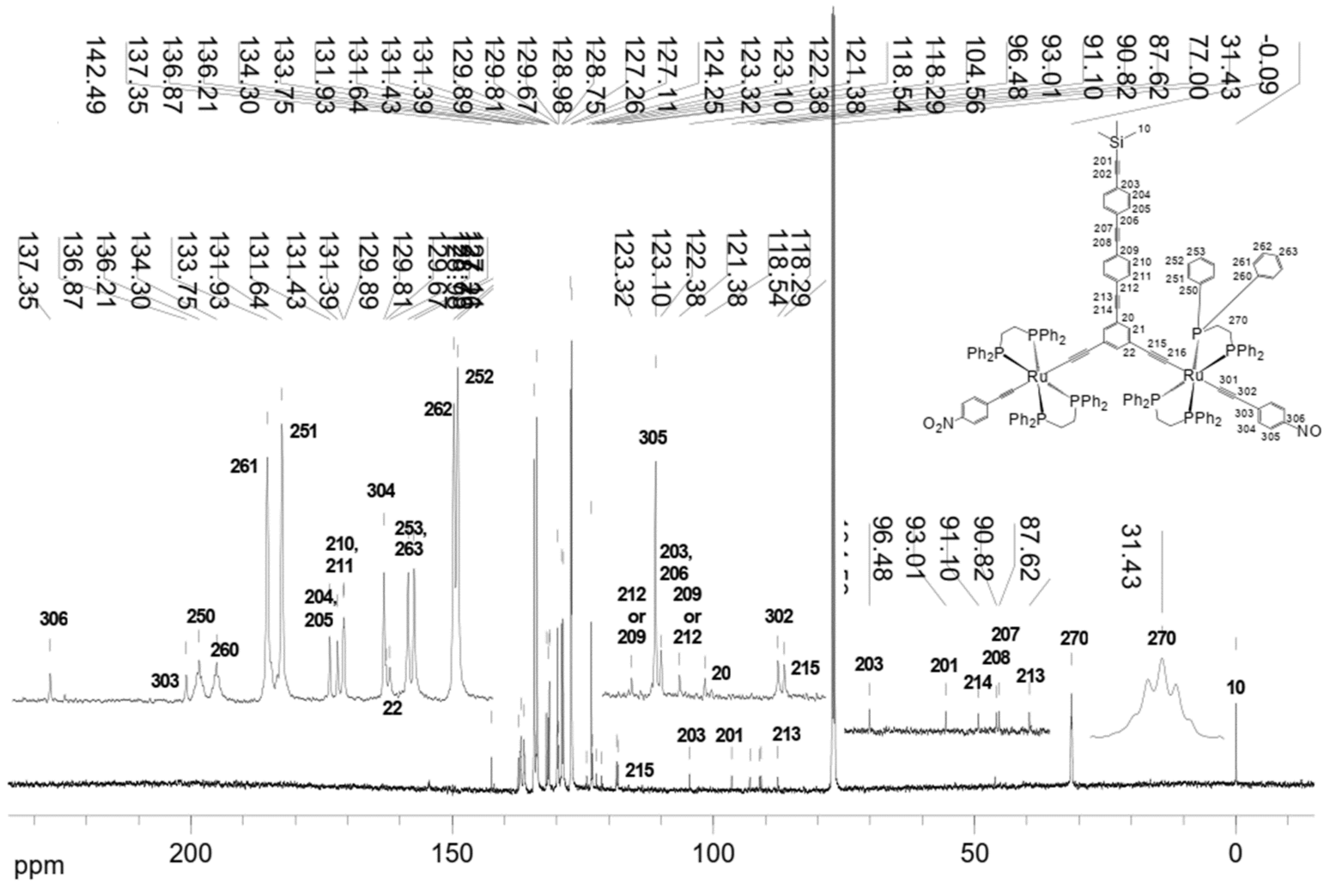

**Fig. S9** $^{13}C\{^{1}H\}$ NMR spectrum of **14** recorded in $CDCl_3$ at 126 MHz.

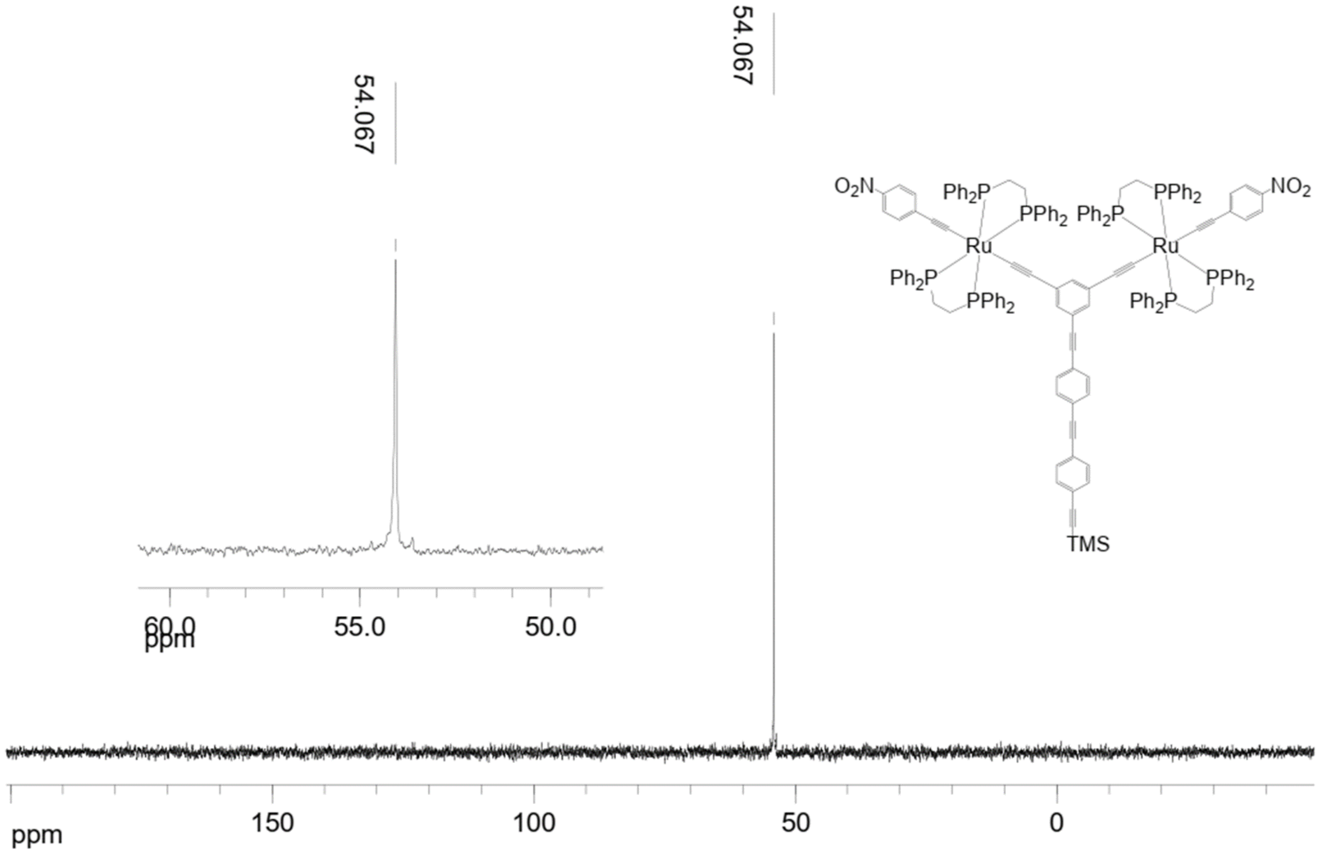


**Fig. S10** $^{31}P\{^1H\}$ NMR spectrum of **14** recorded in $CDCl_3$ at 202 MHz.

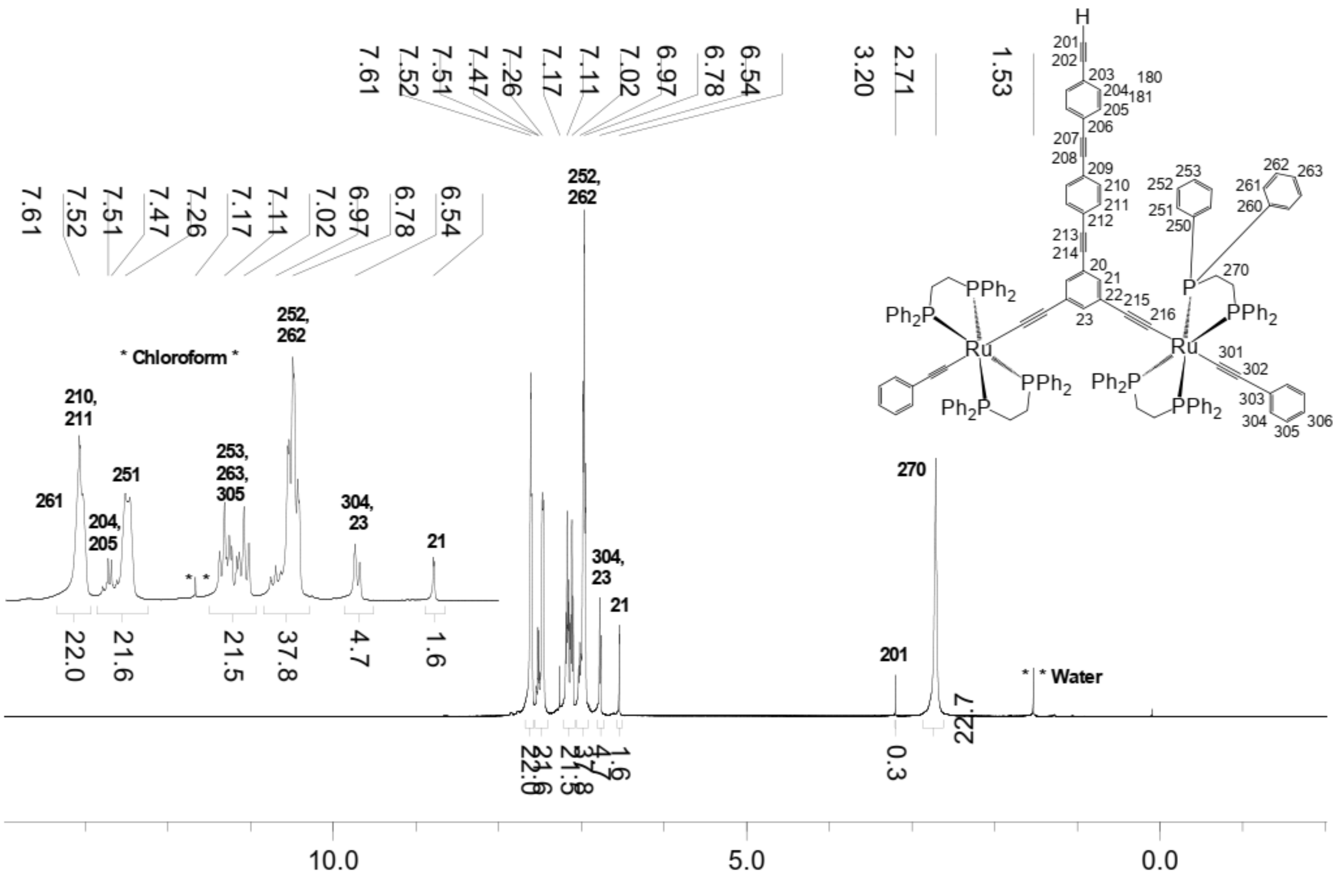


**Fig. S11** $^{1}$H NMR spectrum of **15** recorded in $CDCl_3$ at 500 MHz.

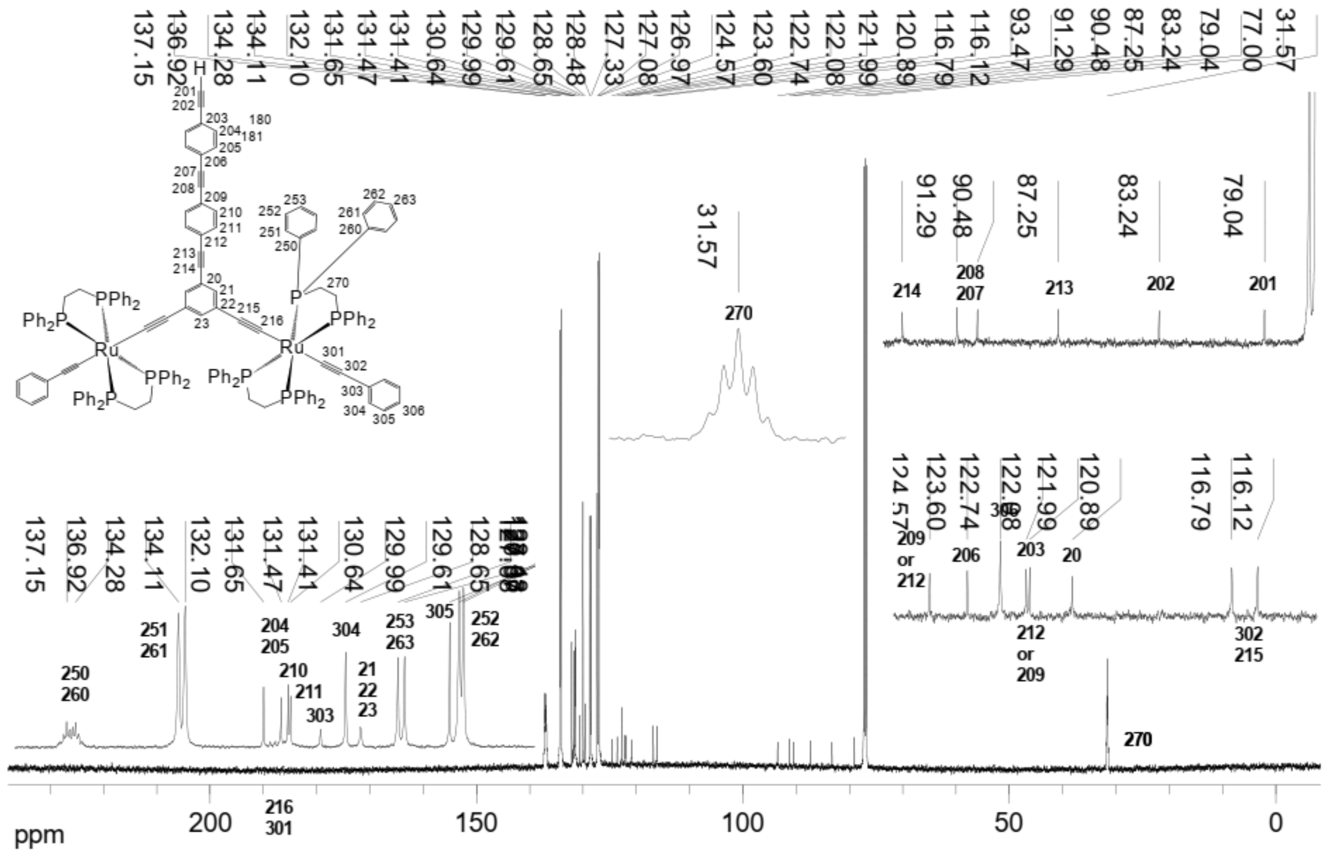


**Fig. S12** $^{13}C\{^{1}H\}$ NMR spectrum of **15** recorded in $CDCl_3$ at 126 MHz.

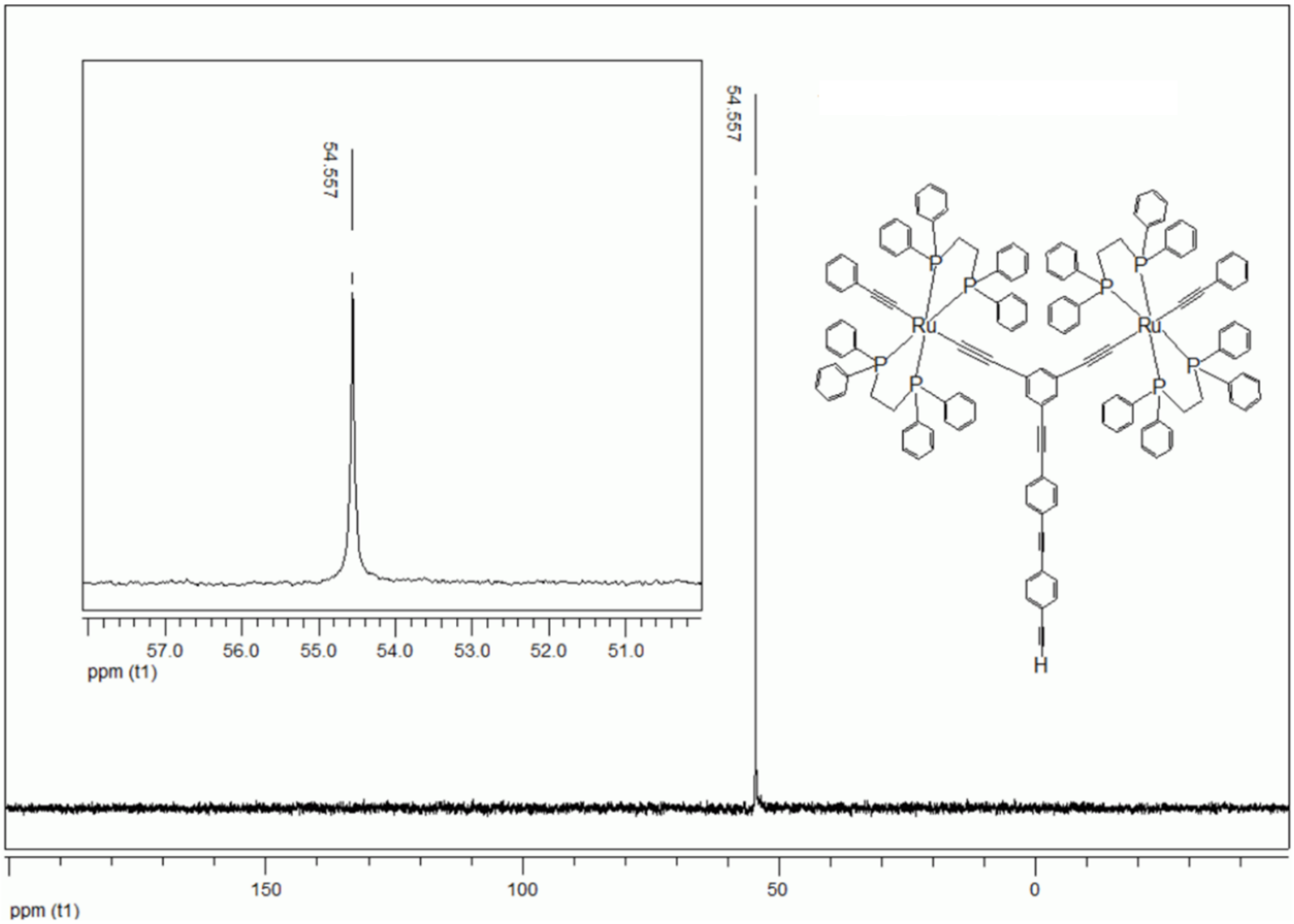


**Fig. S13** $^{31}P\{^1H\}$ NMR spectrum of **15** recorded in $CDCl_3$ at 202 MHz.

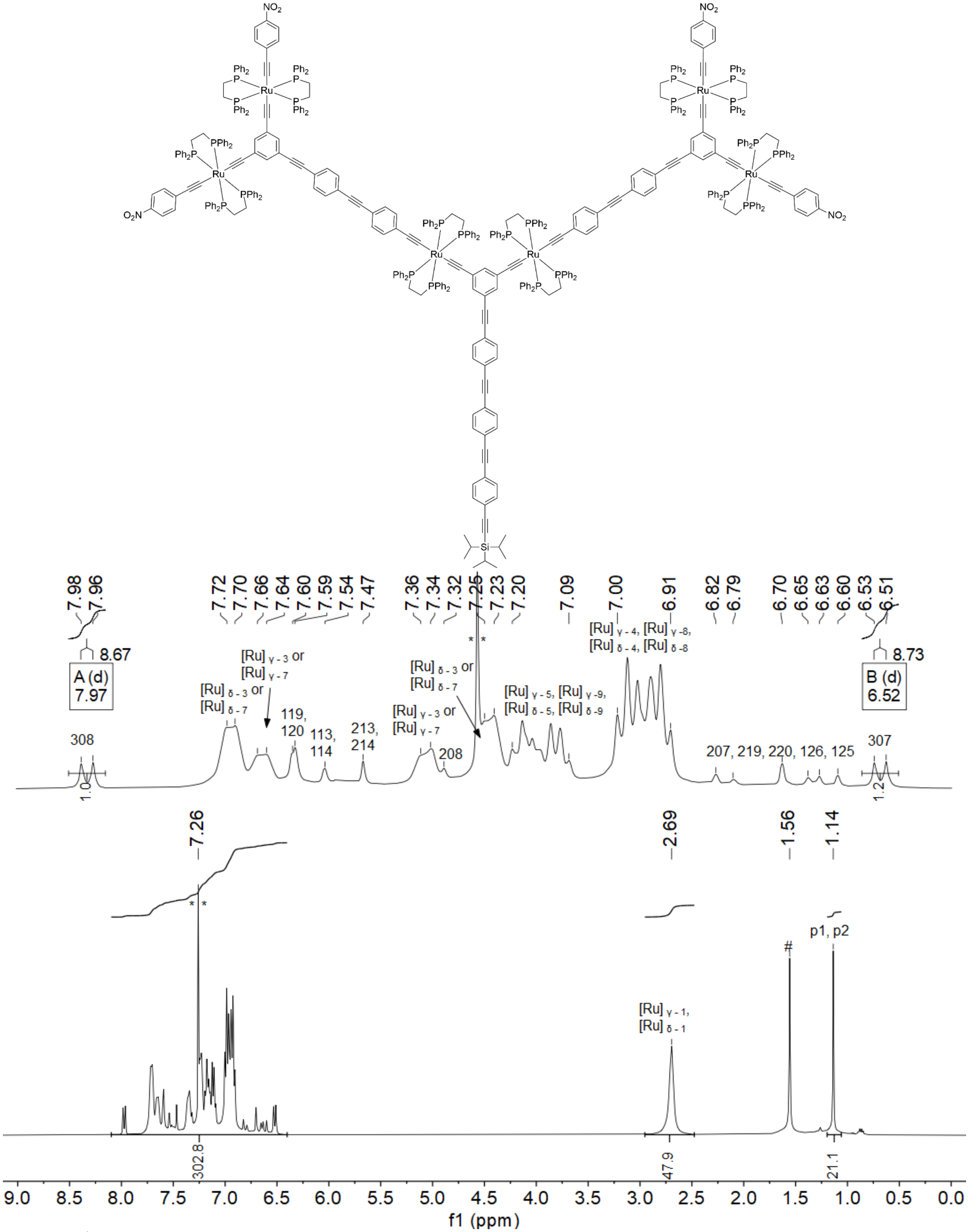


**Fig. S14** $^{1}H$ NMR spectrum of **26** recorded in $CDCl_3$ at 400 MHz. The peak marked as ** corresponds to $CHCl_3$. The peak marked as # corresponds to residual water.

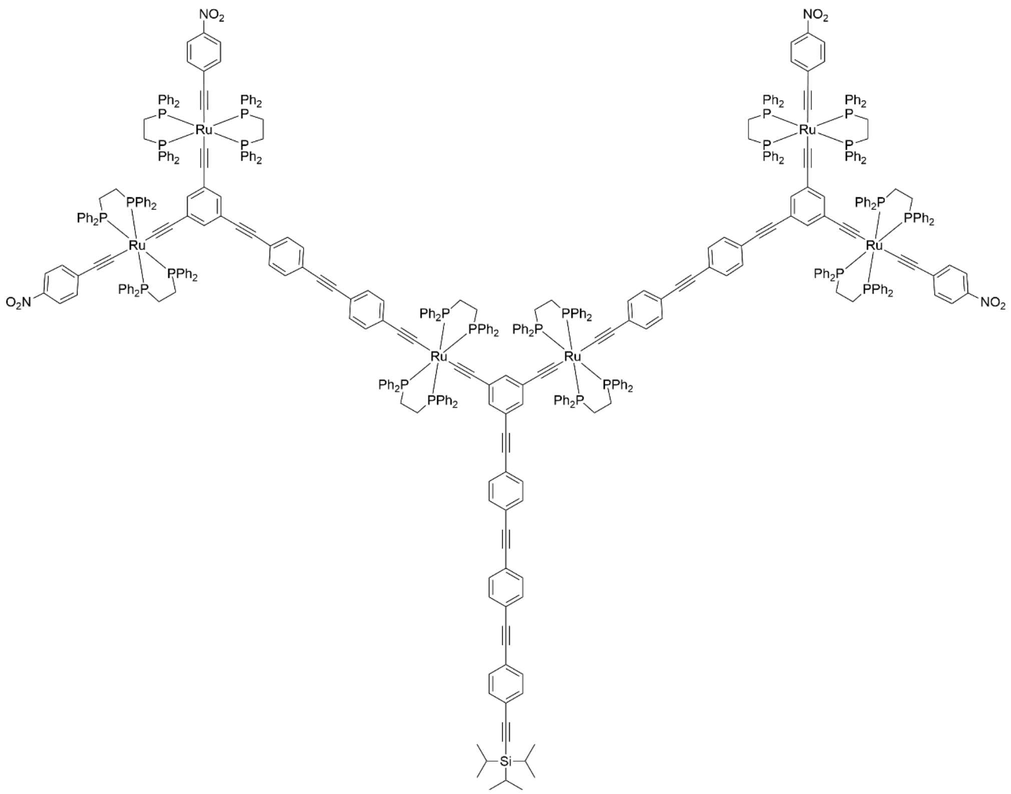

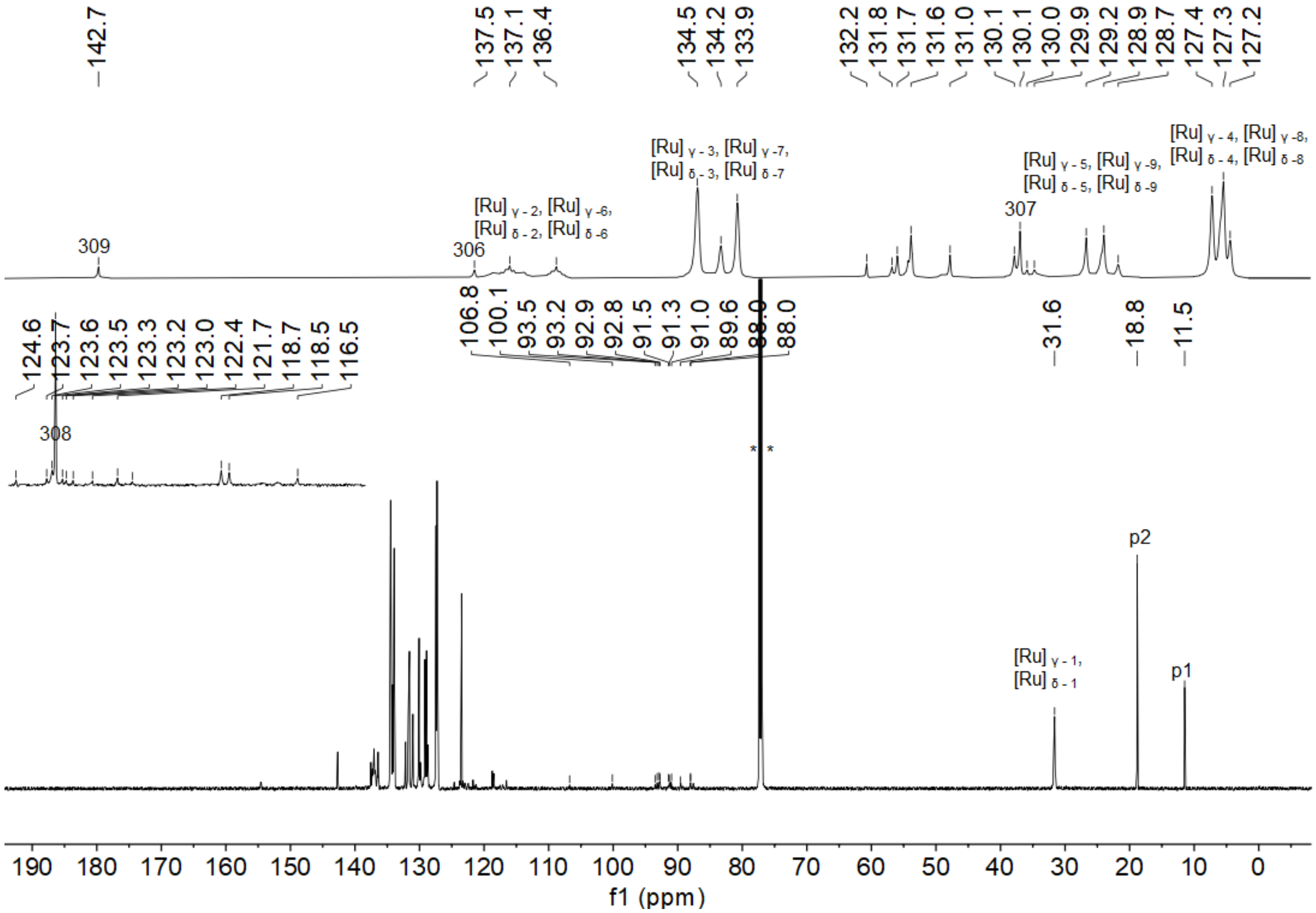

**Fig. S15** $^{13}C\{^{1}H\}$ NMR spectrum of **26** recorded in $CDCl_3$ at 176 MHz. The peak marked as ** corresponds to $CDCl_3$.

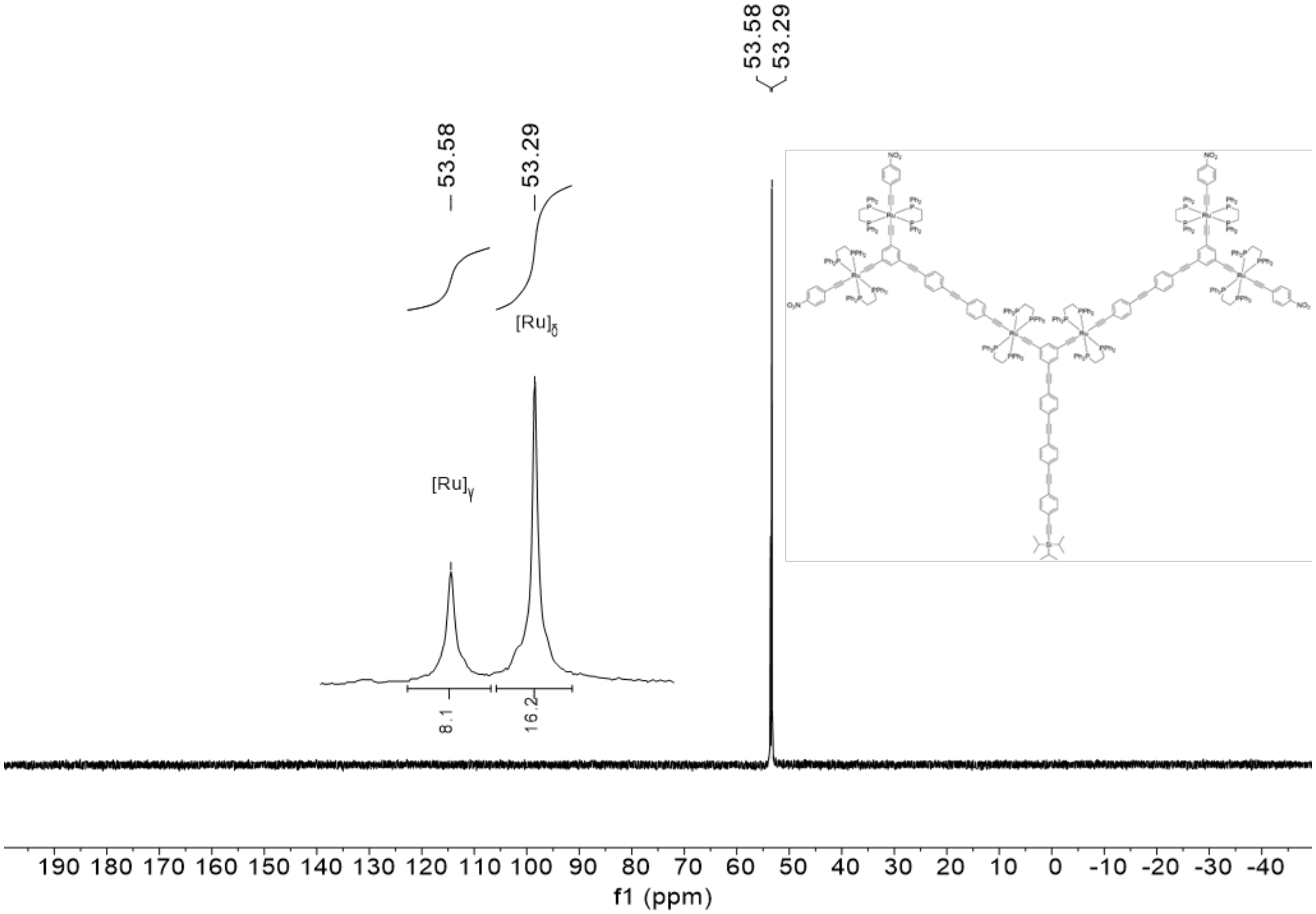


**Fig. S16** $^{31}P\{^{1}H\}$ NMR spectrum of **26** recorded in $CDCl_3$ at 162 MHz.

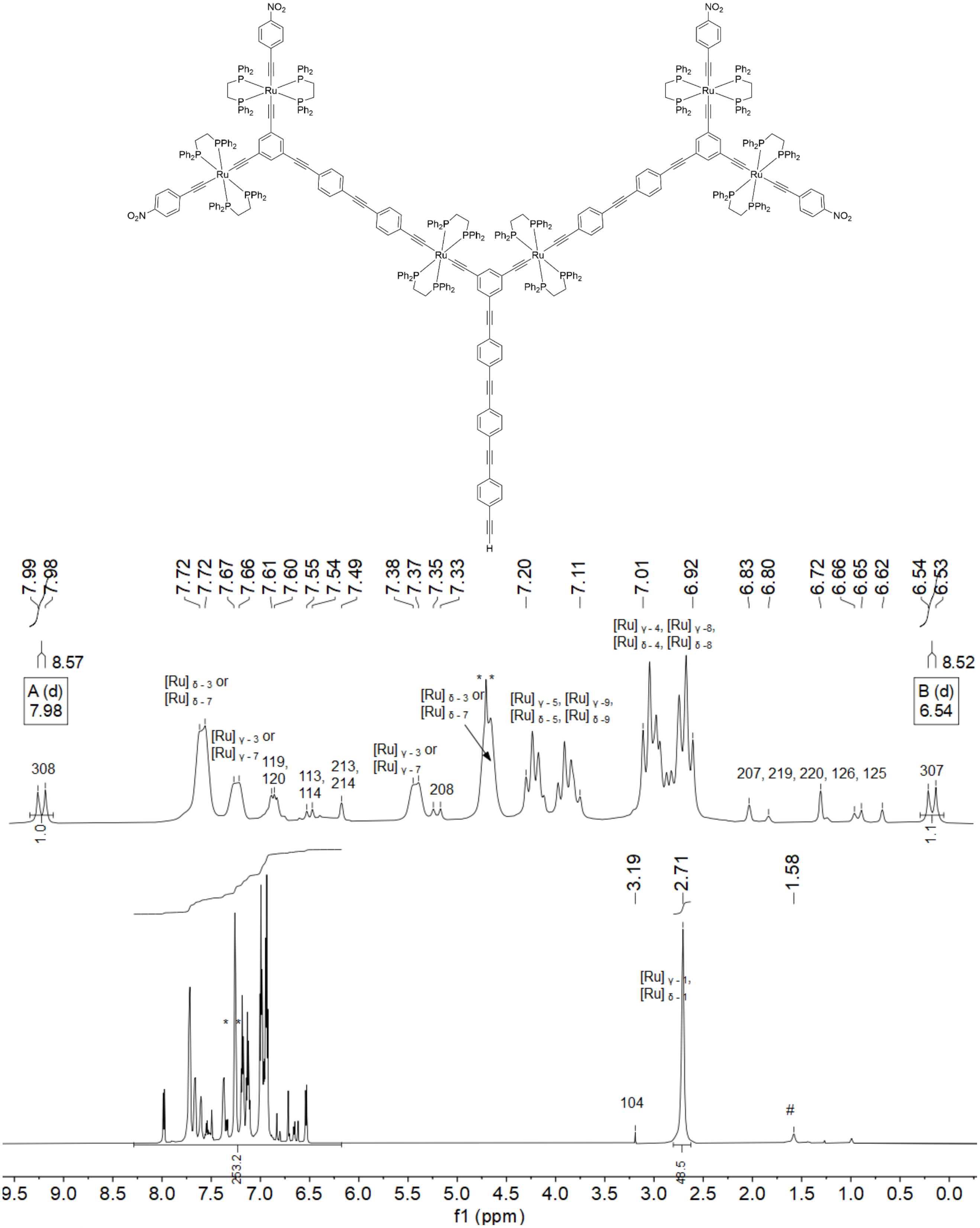


**Fig. S17** $^{1}$H NMR spectrum of **27** recorded in $CDCl_3$ at 700 MHz. The peak marked as ** corresponds to $CHCl_3$. The peak marked as # corresponds to residual water.

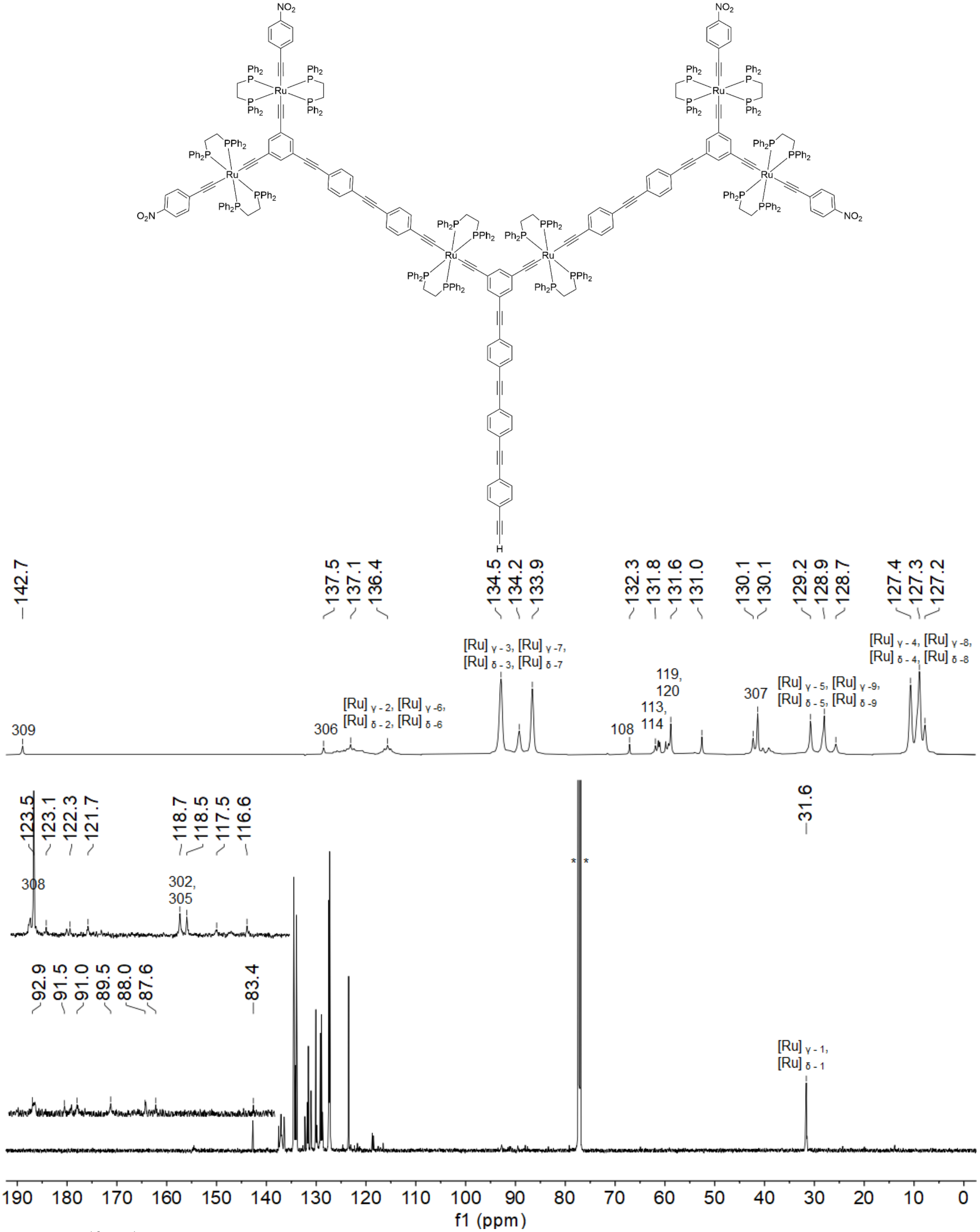


**Fig. S18** $^{13}C\{^1H\}$ NMR spectrum of **27** recorded in $CDCl_3$ at 176 MHz. The peak marked as ** corresponds to $CDCl_3$.

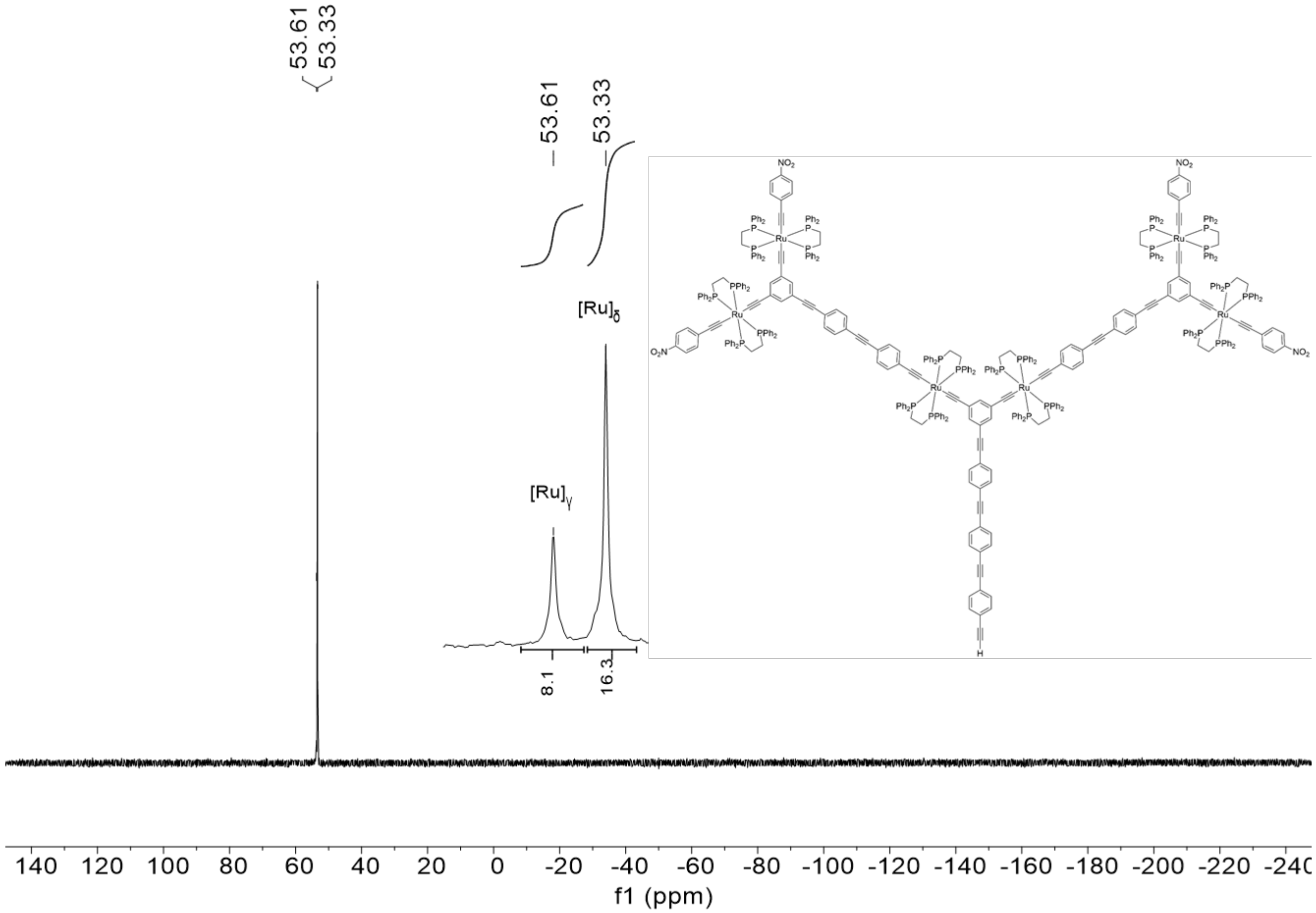


**Fig. S19** $^{31}P\{^1H\}$ NMR spectrum of **27** recorded in $CDCl_3$ at 162 MHz.

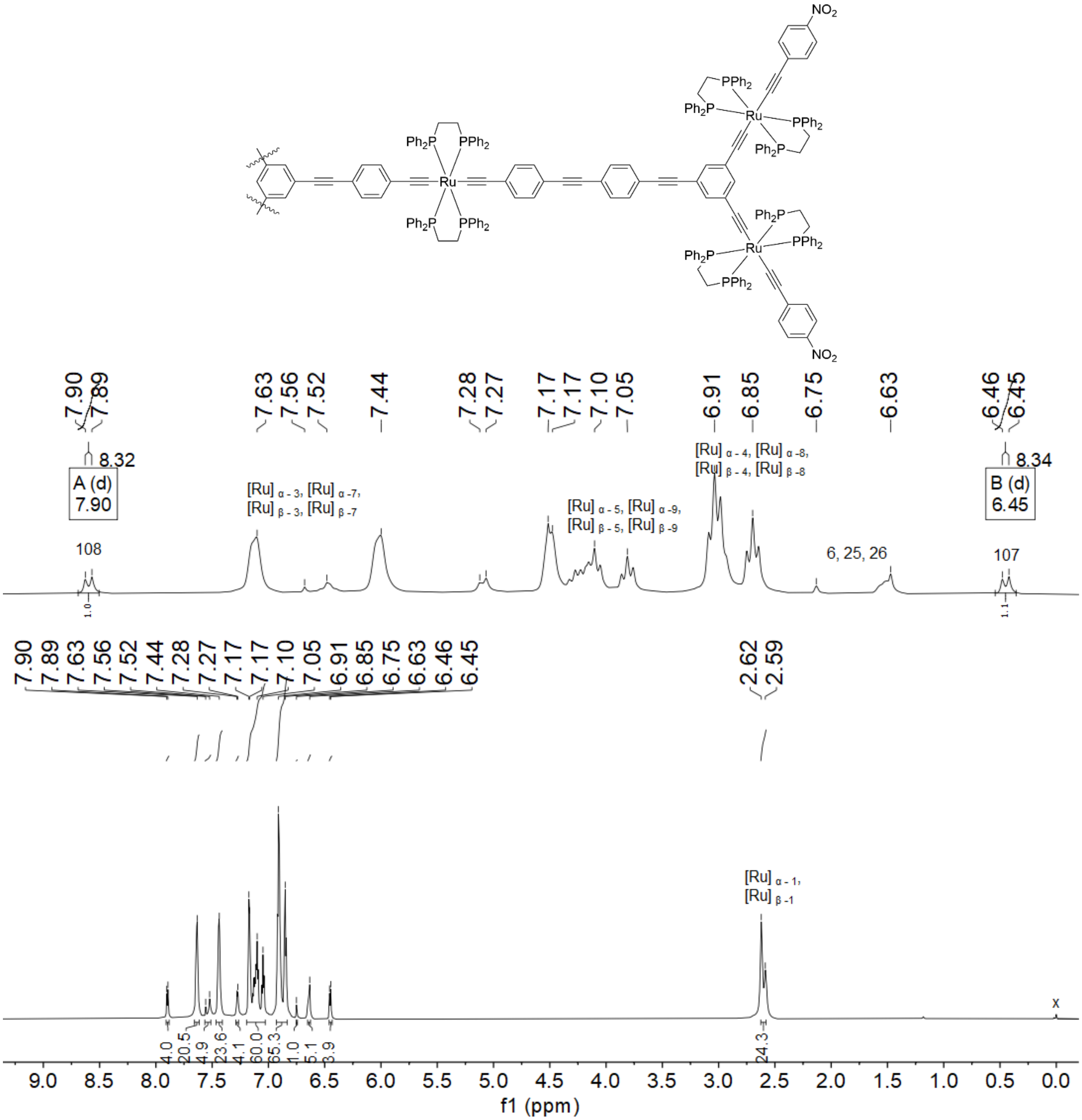


**Fig. S20** $^{1}$H NMR spectrum of **1G$_{12,01}$-NO$_2$** recorded in $CDCl_3$ at 800 MHz. The peak marked as x corresponds to silicone grease.

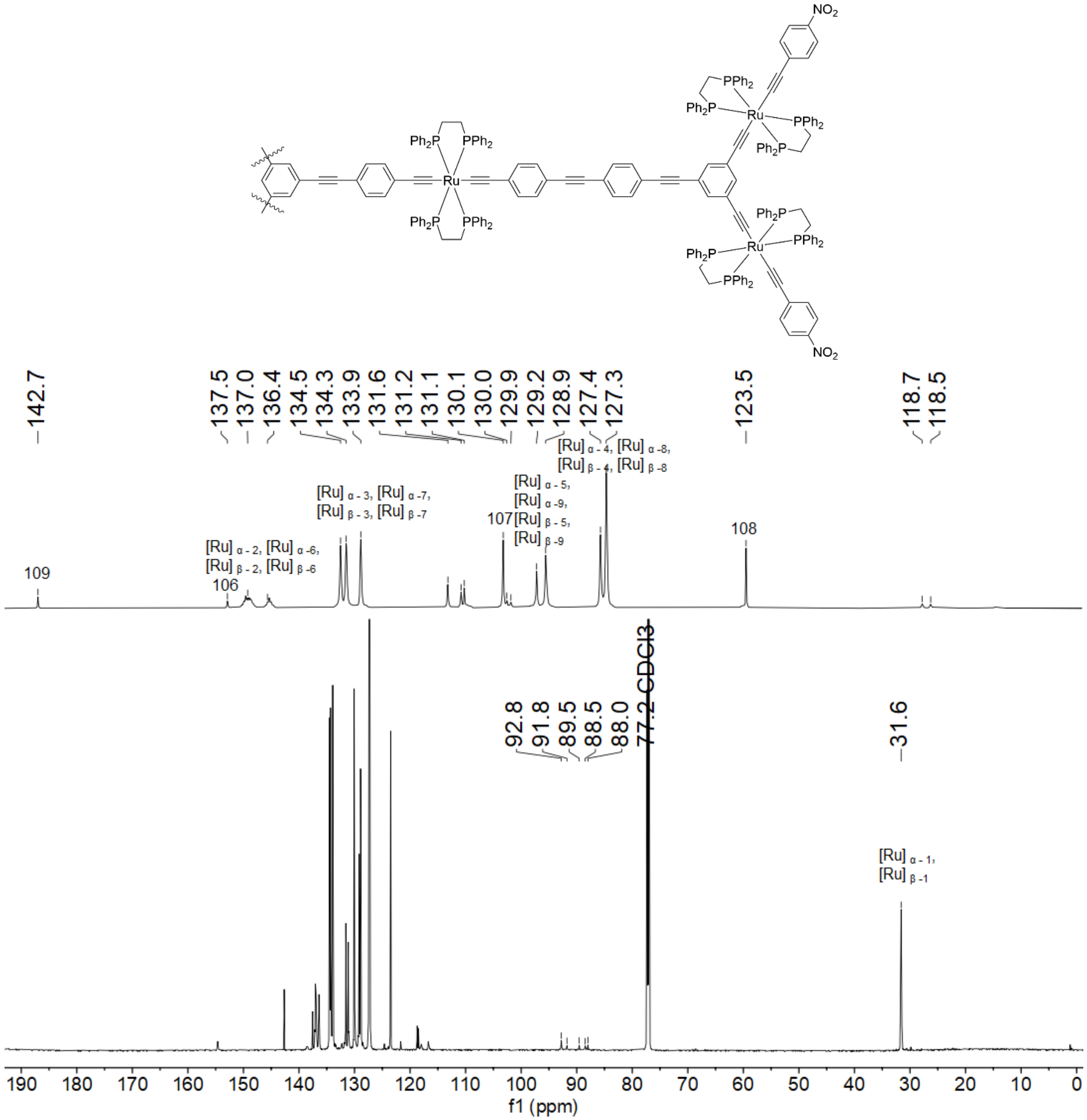


**Fig. S21** $^{13}C\{^{1}H\}$ NMR spectrum of **$1G_{12,01}$-$NO_2$** recorded in $CDCl_3$ at 202 MHz.

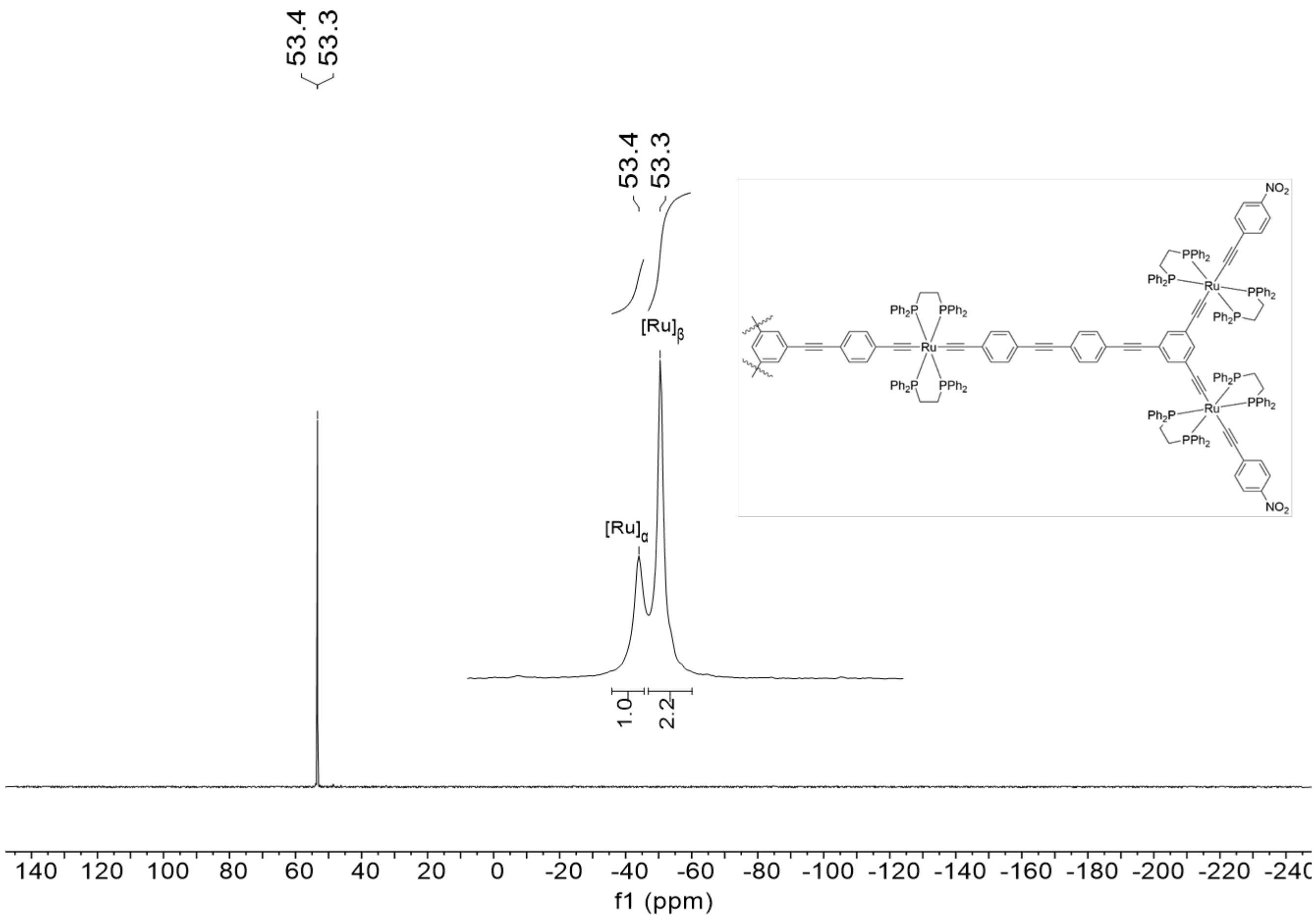


**Fig. S22** $^{31}P\{^{1}H\}$ NMR spectrum of **$1G_{12,01}$-$NO_2$** recorded in $CDCl_3$ at 162 MHz.

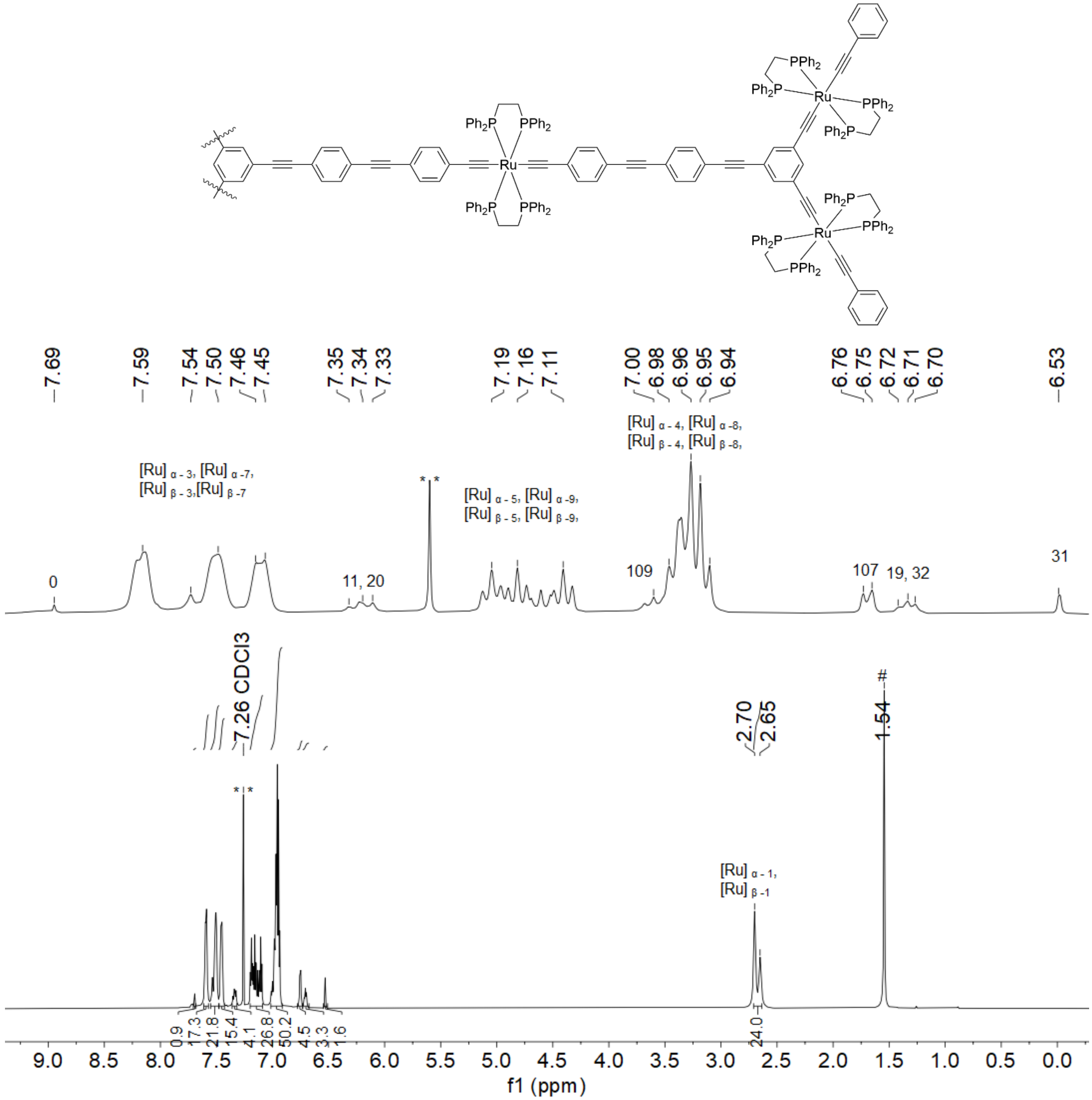

**Fig. S23** $^{1}$H NMR spectrum of **$1G_{22,01}$** recorded in $CDCl_3$ at 600 MHz. The peak marked as ** corresponds to $CHCl_3$. The peak marked as # corresponds to residual water.

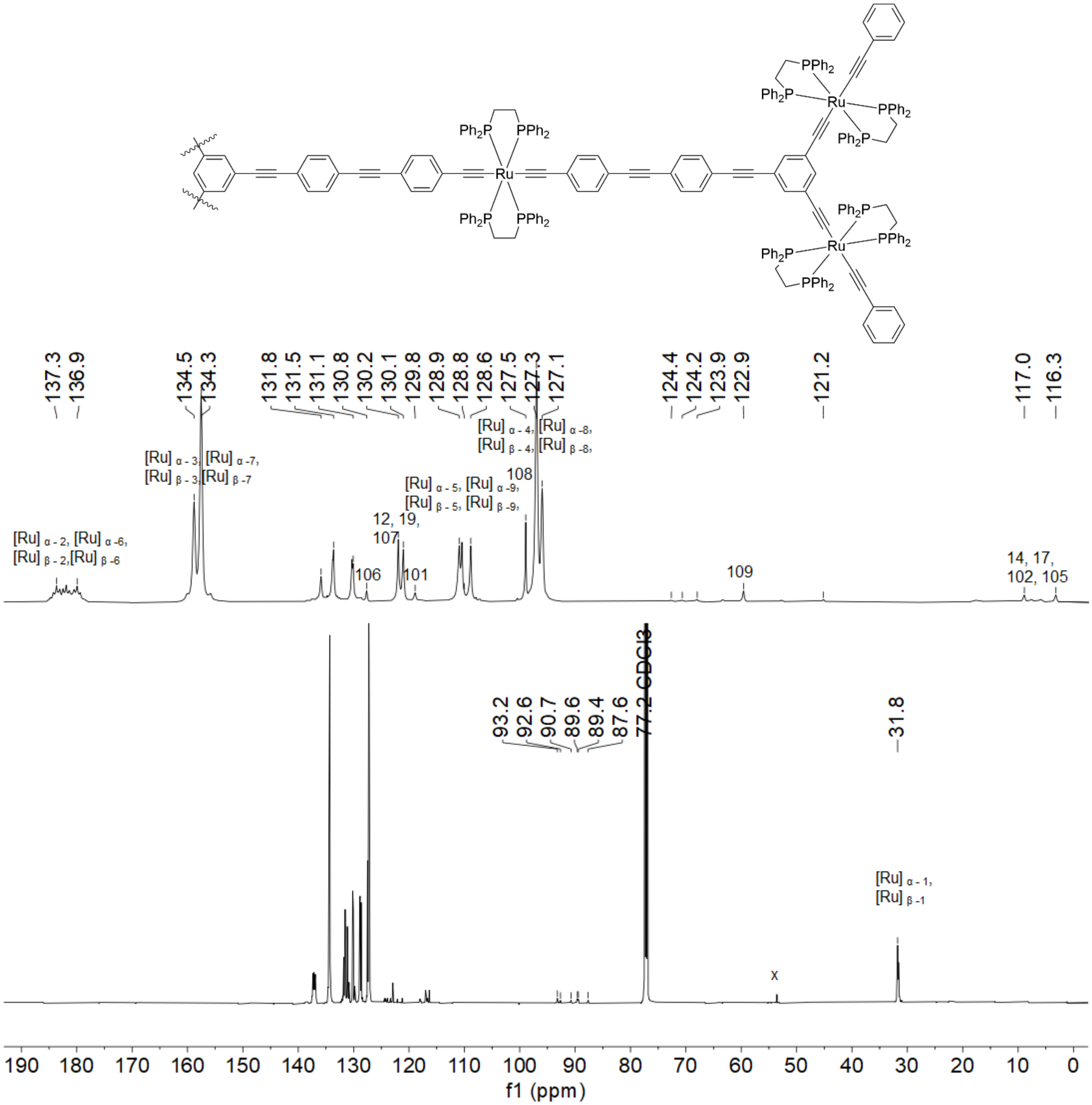

**Fig. S24** $^{13}C\{^1H\}$ NMR spectrum of **$1G_{22,01}$** recorded in $CDCl_3$ at 151 MHz. The peak marked as x corresponds to residual $CH_2Cl_2$.

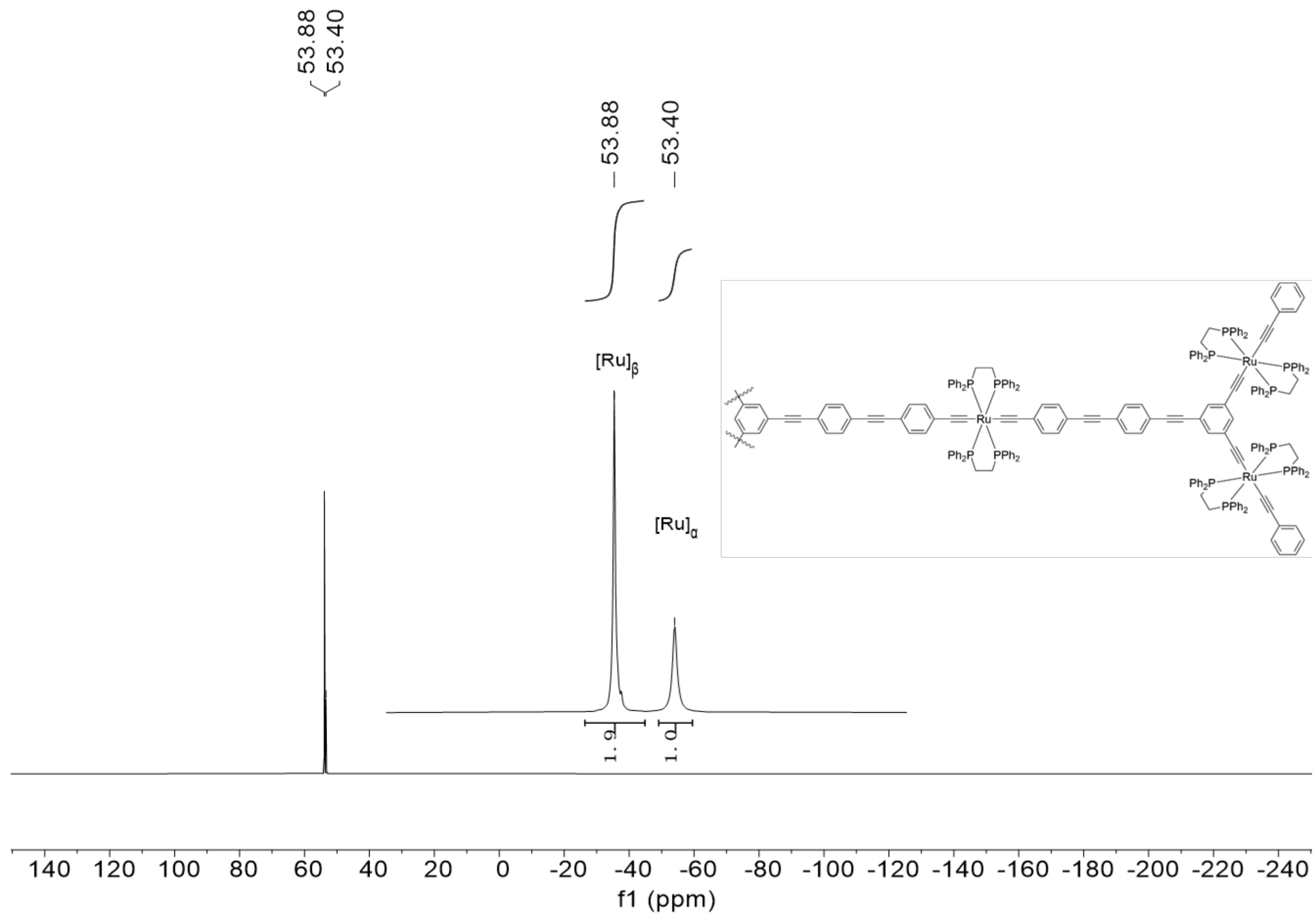


**Fig. S25** $^{31}P\{^1H\}$ NMR spectrum of $\mathbf{1G_{22,01}}$ recorded in $CDCl_3$ at 283 MHz.

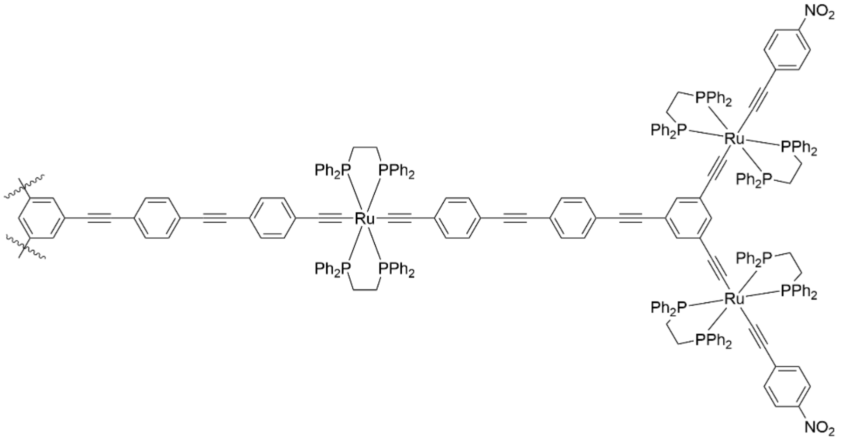

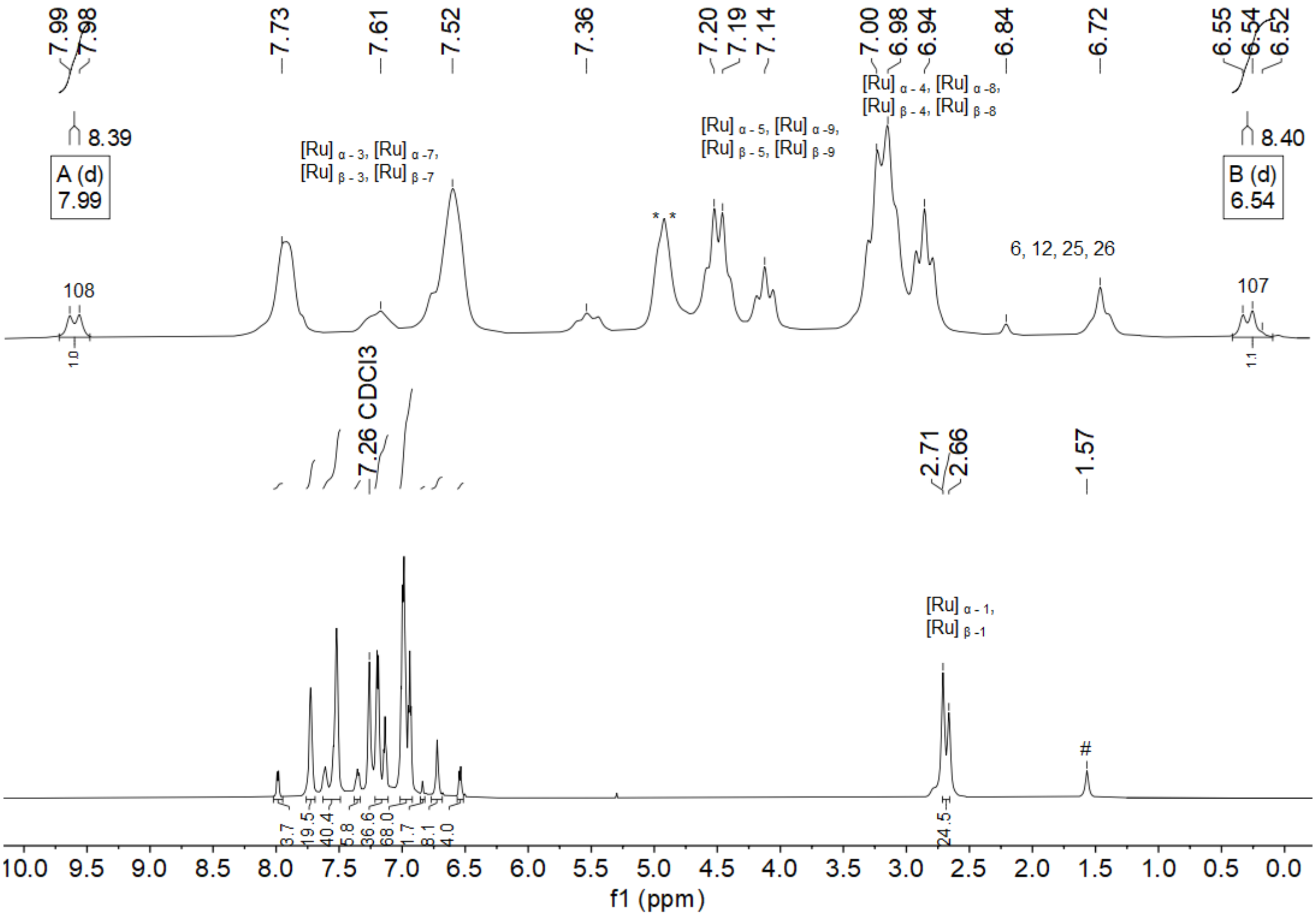


**Fig. S26** $^{1}$H NMR spectrum of **$1G_{22,01}$-$NO_2$** recorded in $CDCl_3$ at 700 MHz.

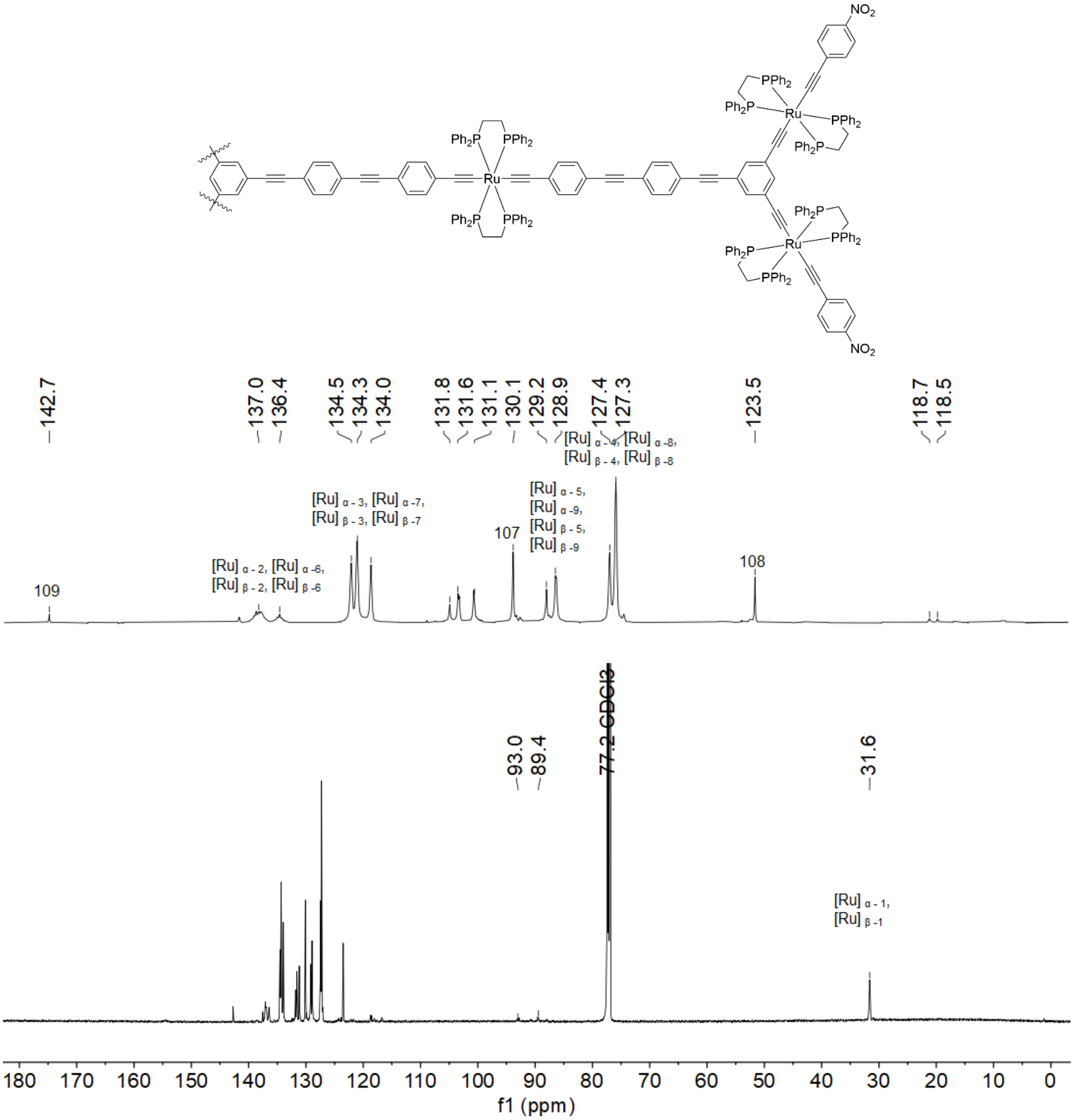


**Fig. S27** $^{13}C\{^{1}H\}$ NMR spectrum of **$1G_{22,01}$-$NO_2$** recorded in $CDCl_3$ at 151 MHz.

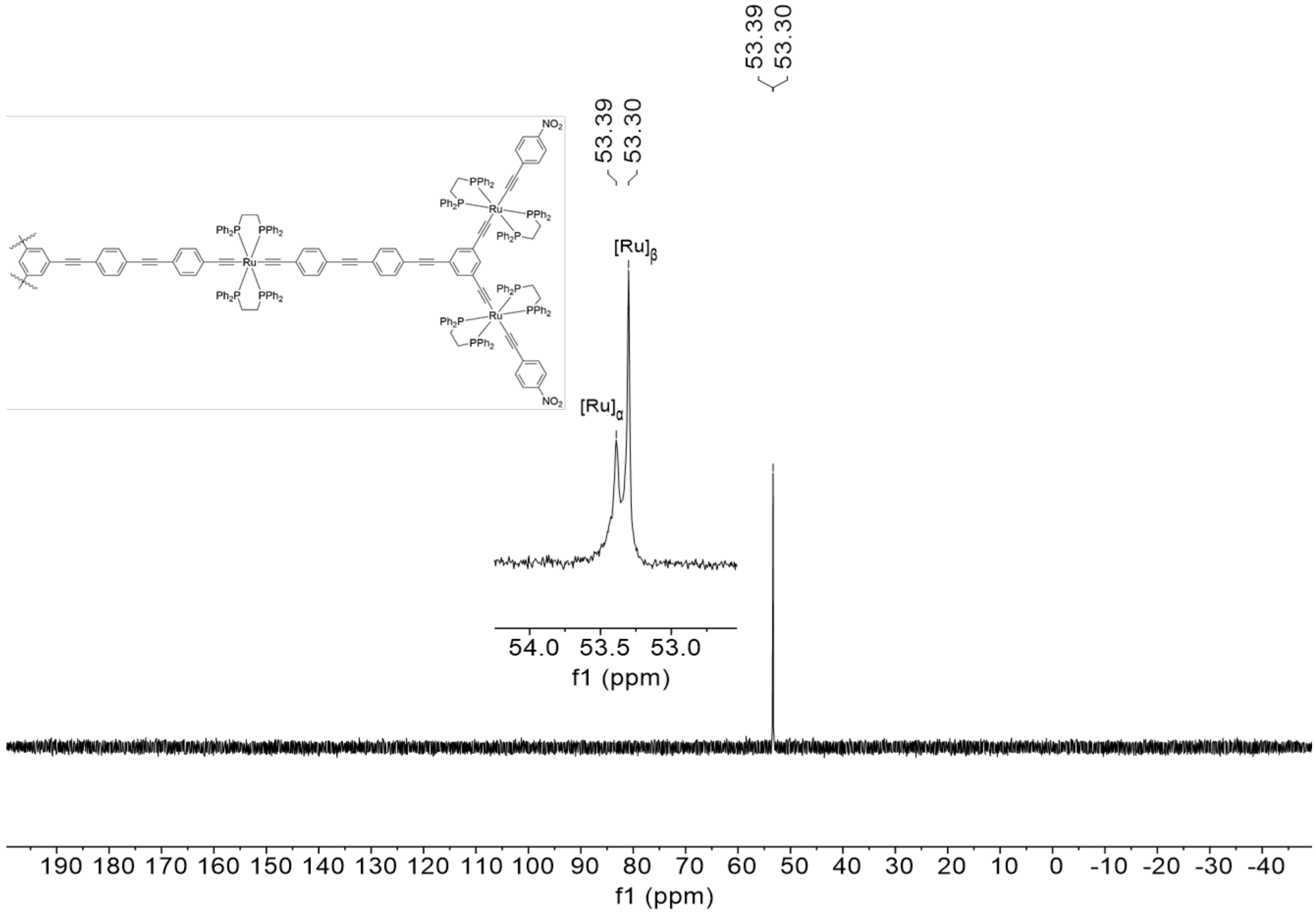


**Fig. S28** $^{31}P\{^1H\}$ NMR spectrum of **$1G_{22,01}$-$NO_2$** recorded in $CDCl_3$ at 162 MHz.

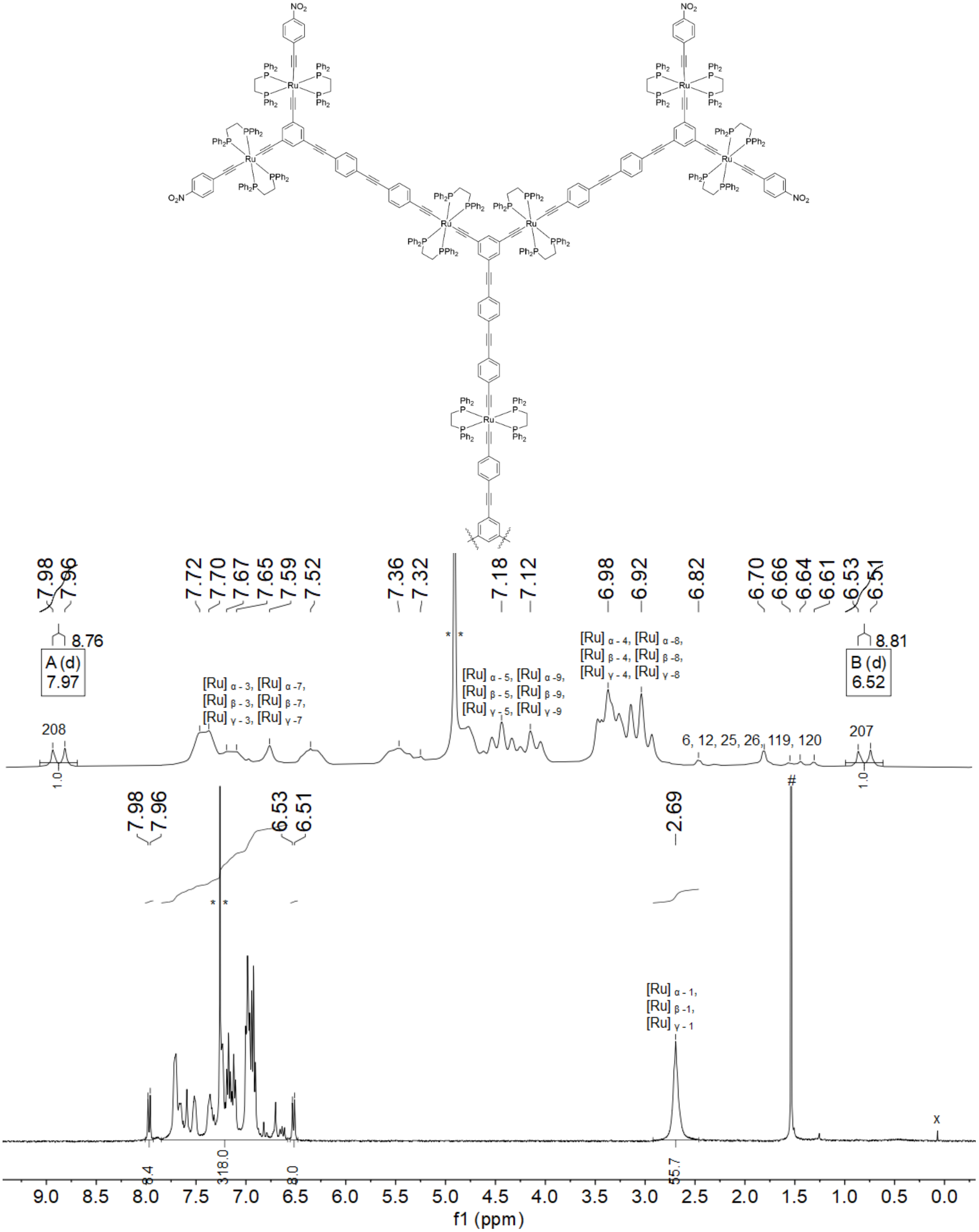


**Fig. S29** $^1$H NMR spectrum of **$2G_{12,02,01}$-$NO_2$** recorded in $CDCl_3$ at 400 MHz. The peak marked as * * corresponds to $CHCl_3$. The peak marked as # corresponds to residual water. The peak marked as x corresponds to silicone grease.

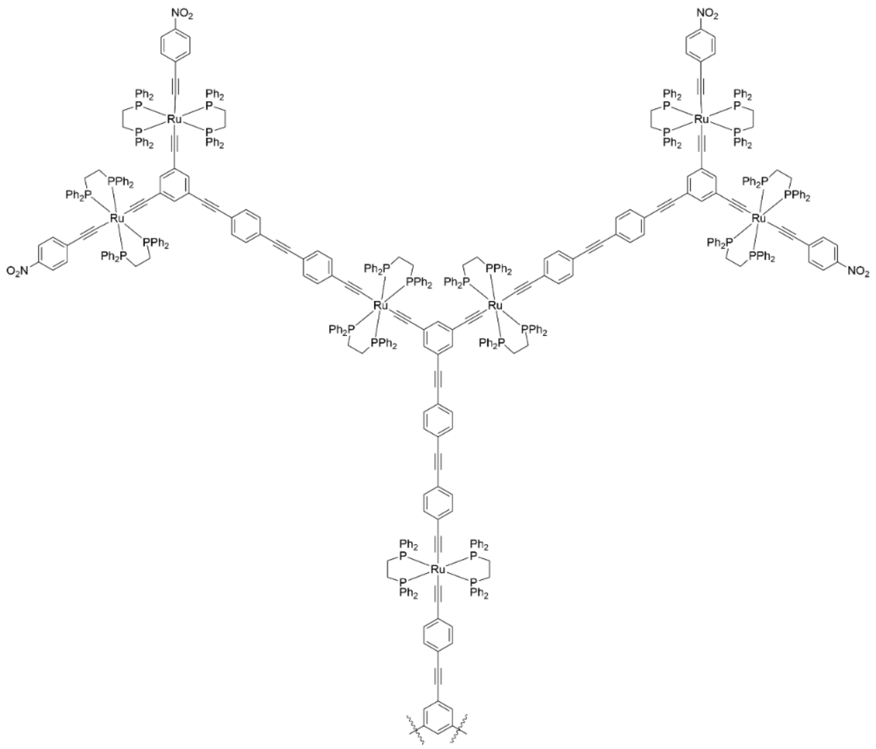


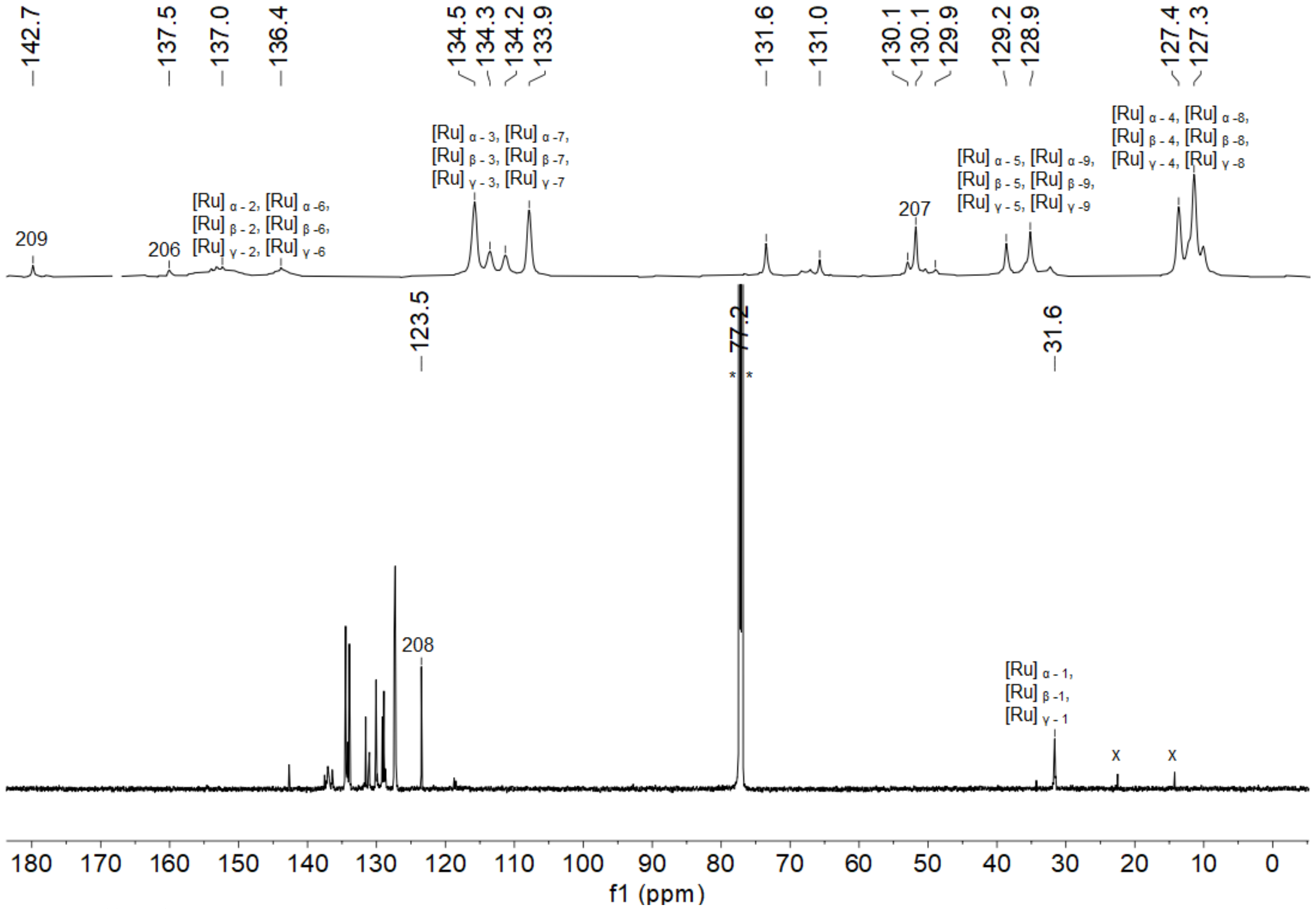


**Fig. S30** $^{13}C\{^{1}H\}$ NMR spectrum of **$2G_{12,02,01}$-$NO_2$** recorded in $CDCl_3$ at 176 MHz. The peak marked as ** corresponds to $CDCl_3$. The peaks marked as x corresponds to residual *n*-pentane.

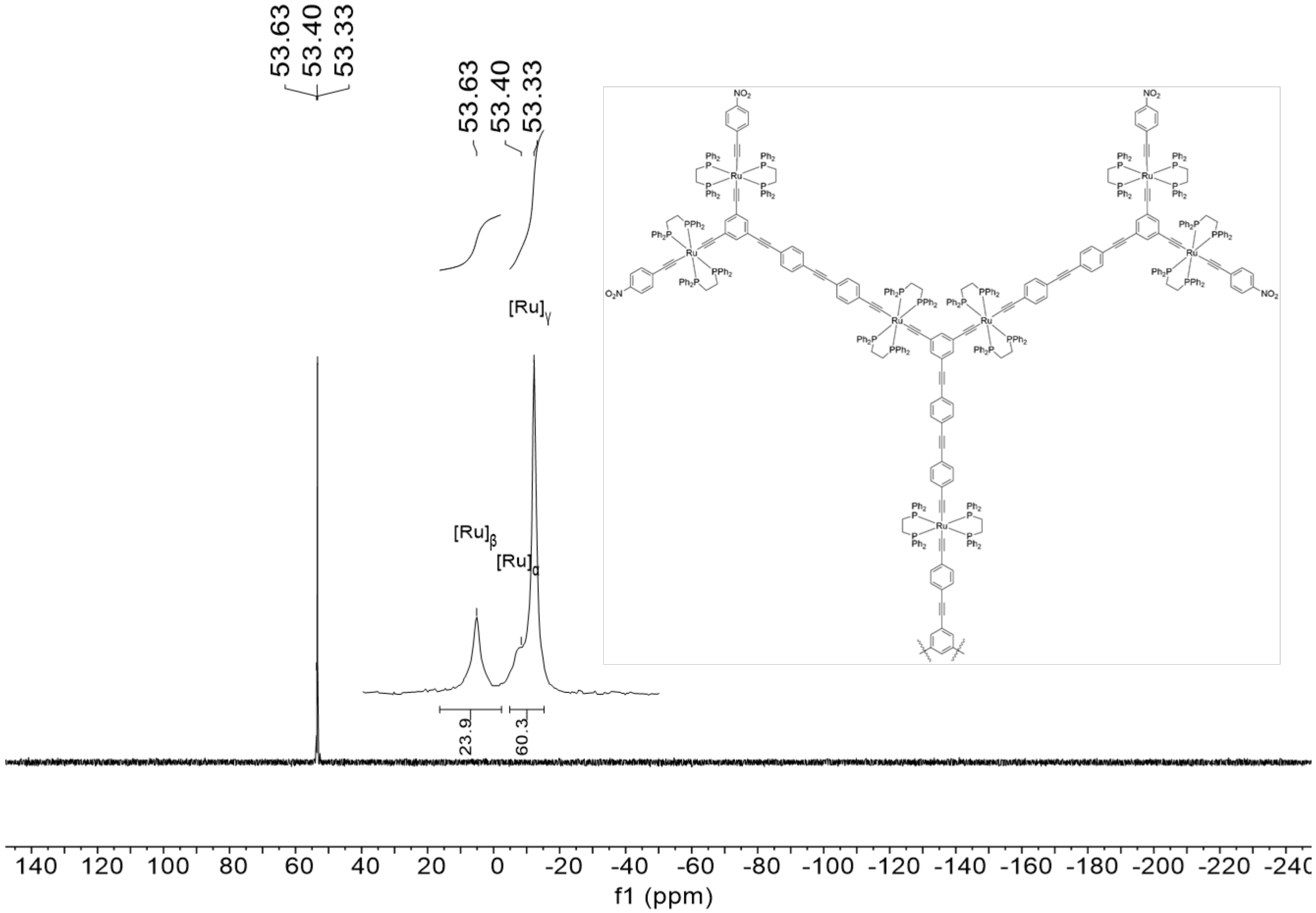


**Fig. S31** $^{31}P\{^{1}H\}$ NMR spectrum of **$2G_{12,02,01}$-$NO_2$** recorded in $CDCl_3$ at 162 MHz.

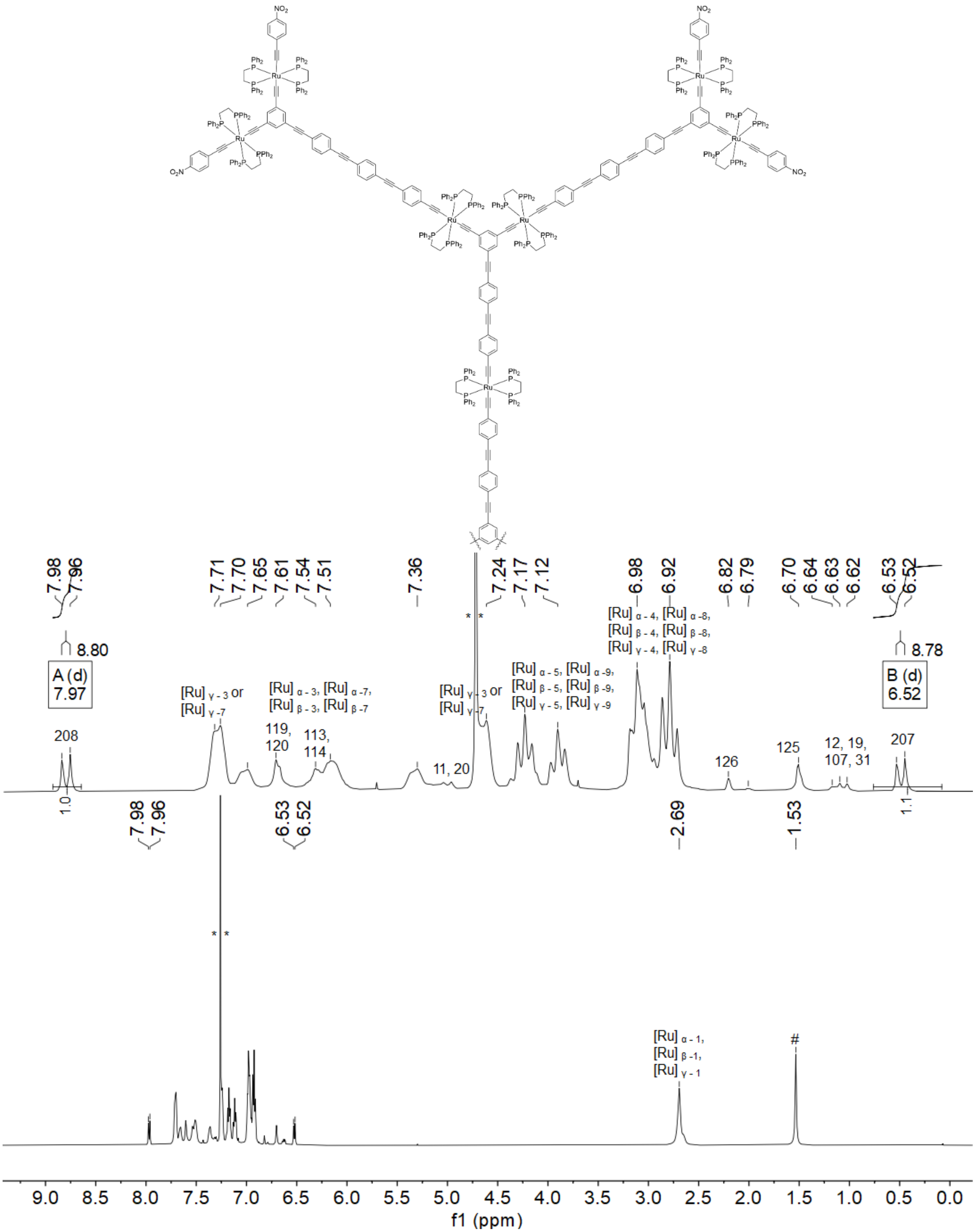


**Fig. S32** $^{1}$H NMR spectrum of **$2G_{22,03,01}$-$NO_2$** recorded in $CDCl_3$ at 600 MHz. The peak marked as ** corresponds to residual $CHCl_3$. The peak marked as # corresponds to residual water.

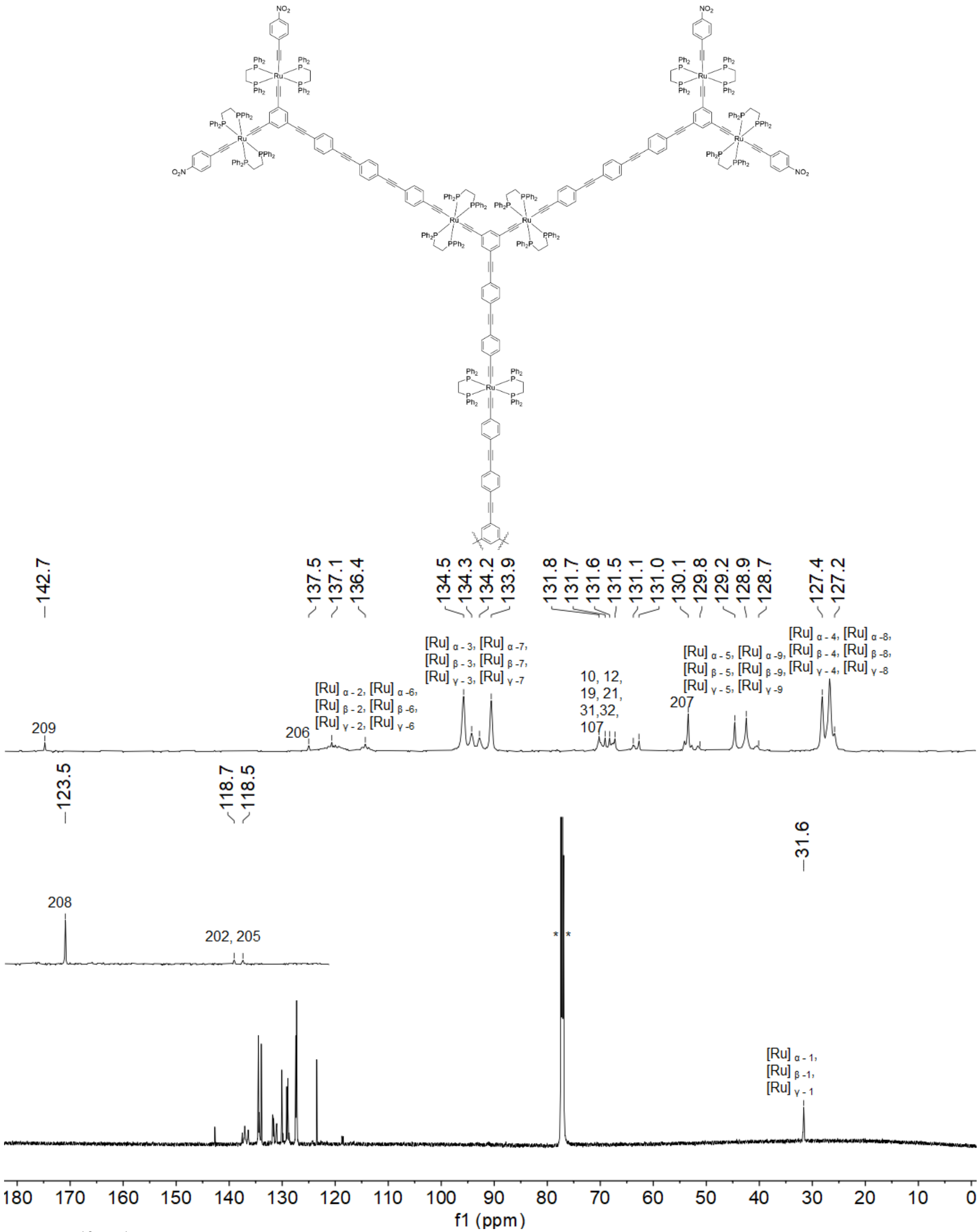


**Fig. S33** $^{13}C\{^{1}H\}$ NMR spectrum of **$2G_{22,03,01}$-$NO_2$** recorded in $CDCl_3$ at 151 MHz. The peak marked as ** corresponds to $CDCl_3$.

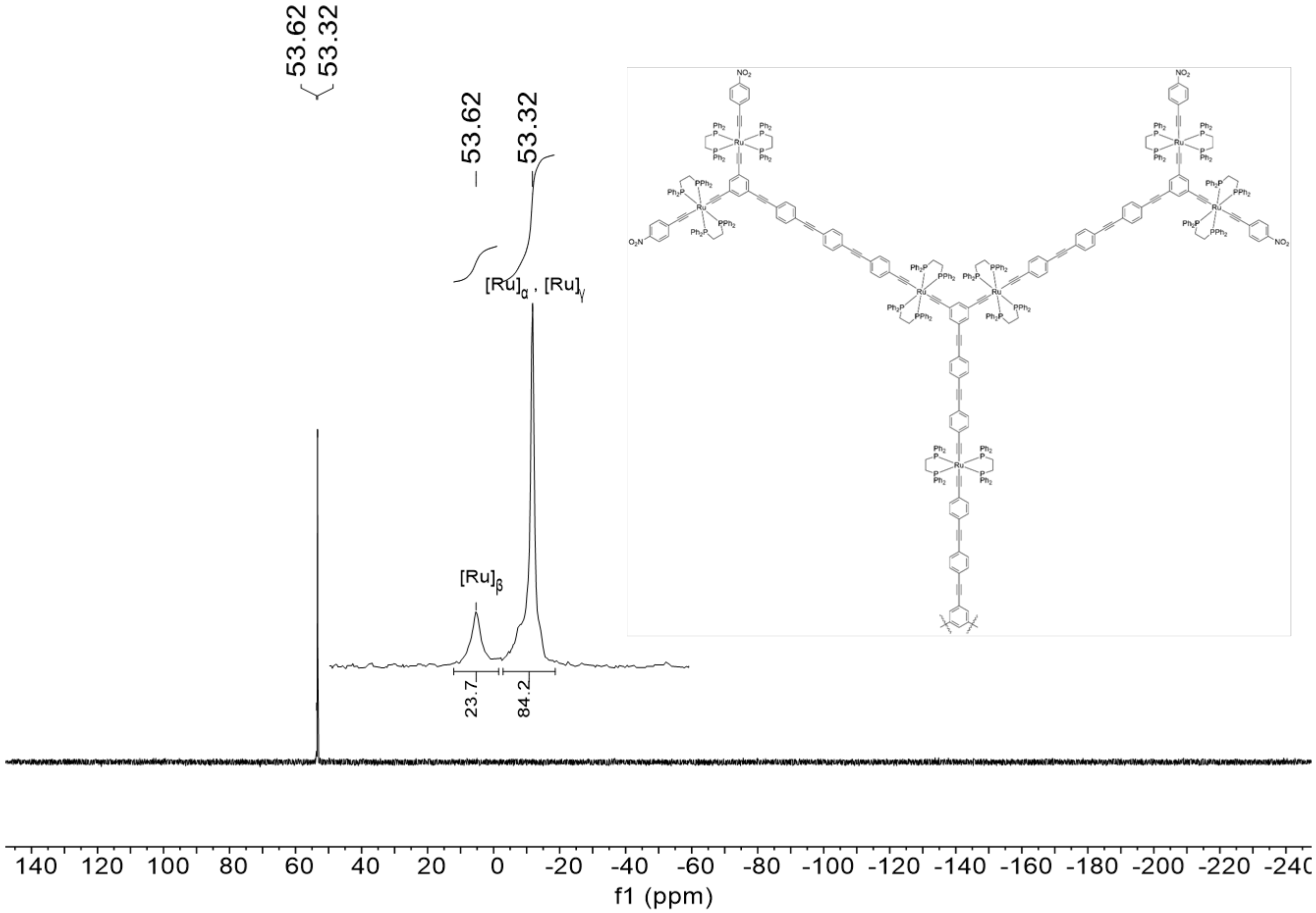


**Fig. S34** $^{31}P\{^{1}H\}$ NMR spectrum of $\mathbf{2G_{22,03,01}\text{-}NO_2}$ recorded in $CDCl_3$ at 162 MHz.

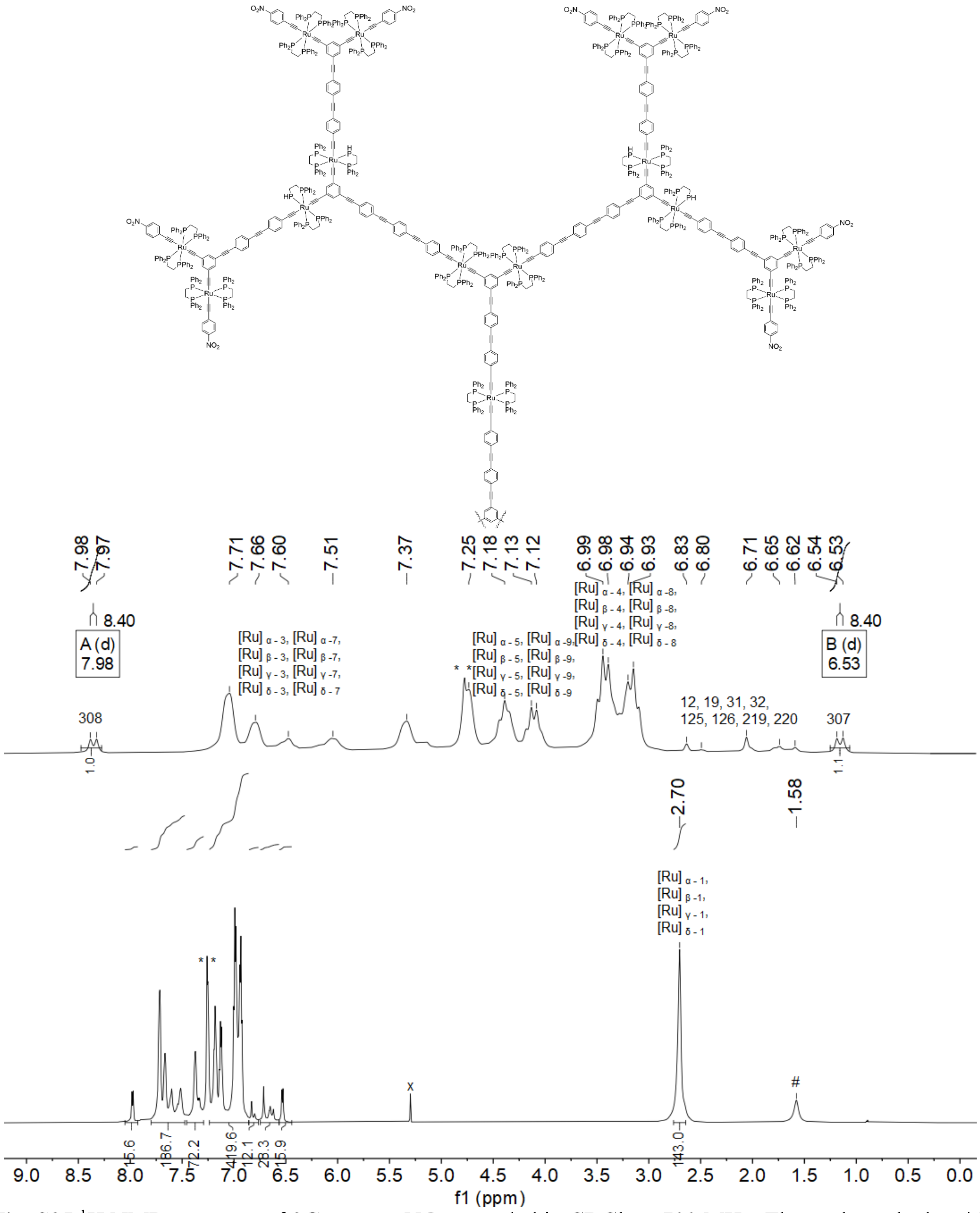


**Fig. S35** $^1$H NMR spectrum of **3G$_{22,03,02,01}$-NO$_2$** recorded in $CDCl_3$ at 700 MHz. The peak marked as ** corresponds to $CHCl_3$. The peak marked as # corresponds to residual water. The peak marked as x corresponds to residual $CH_2Cl_2$.

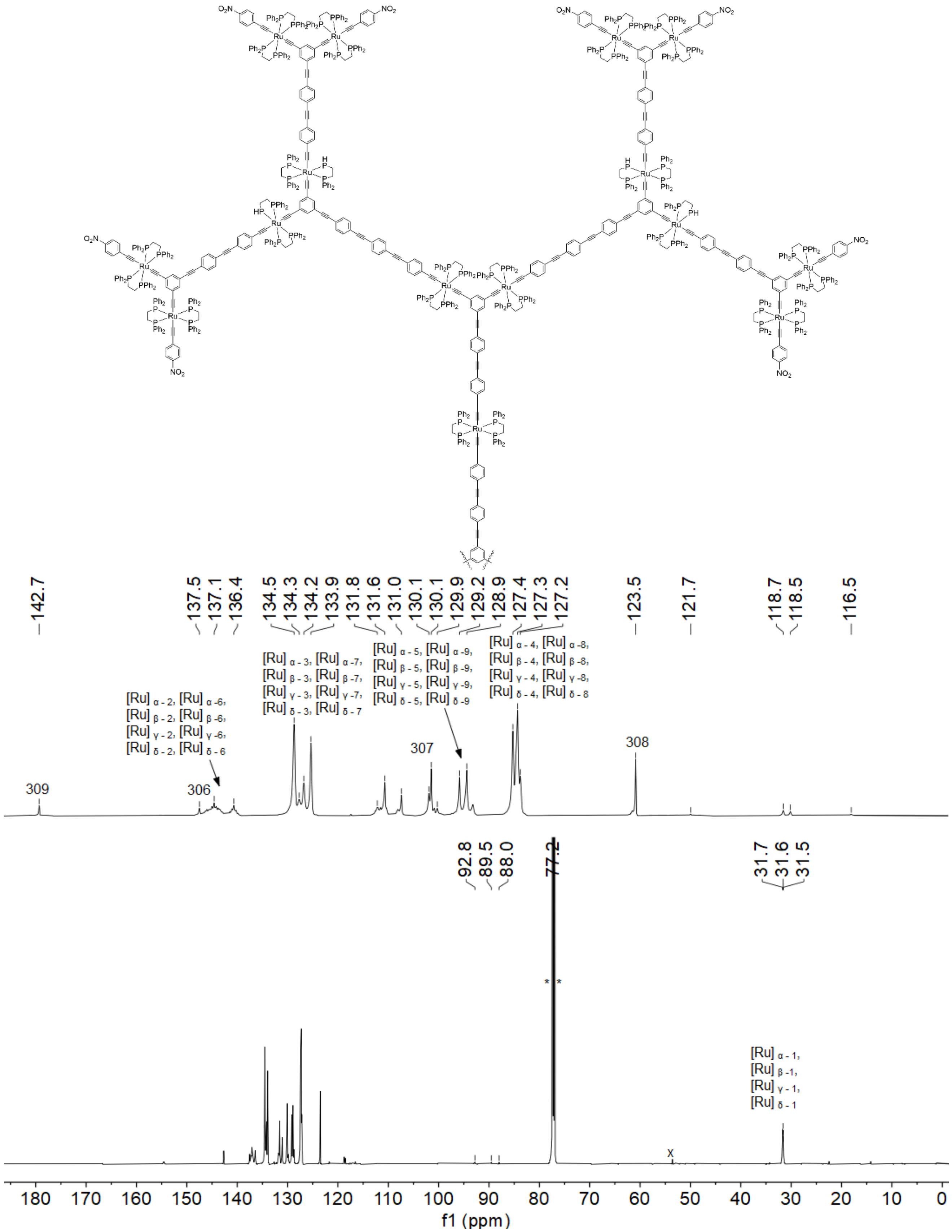


**Fig. S36** $^{13}C\{^1H\}$ NMR spectrum of **$3G_{22,03,02,01}$-$NO_2$** recorded in $CDCl_3$ at 151 MHz. The peak marked as ** corresponds to $CDCl_3$. The peak marked as x corresponds to residual $CH_2Cl_2$.

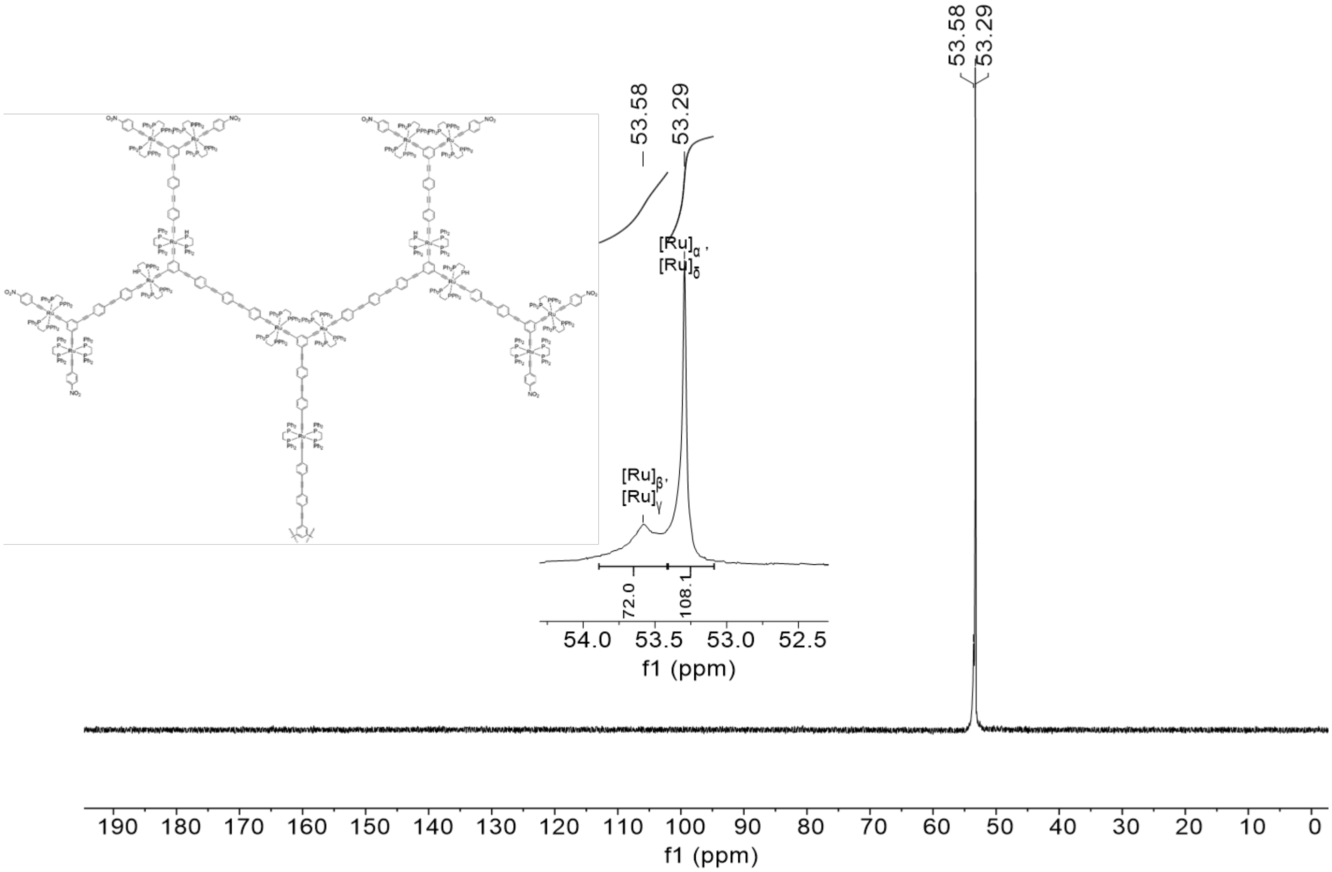

**Fig. S37** $^{31}P\{^1H\}$ NMR spectrum of **$3G_{22,03,02,01}$-$NO_2$** recorded in $CDCl_3$ at 162 MHz.

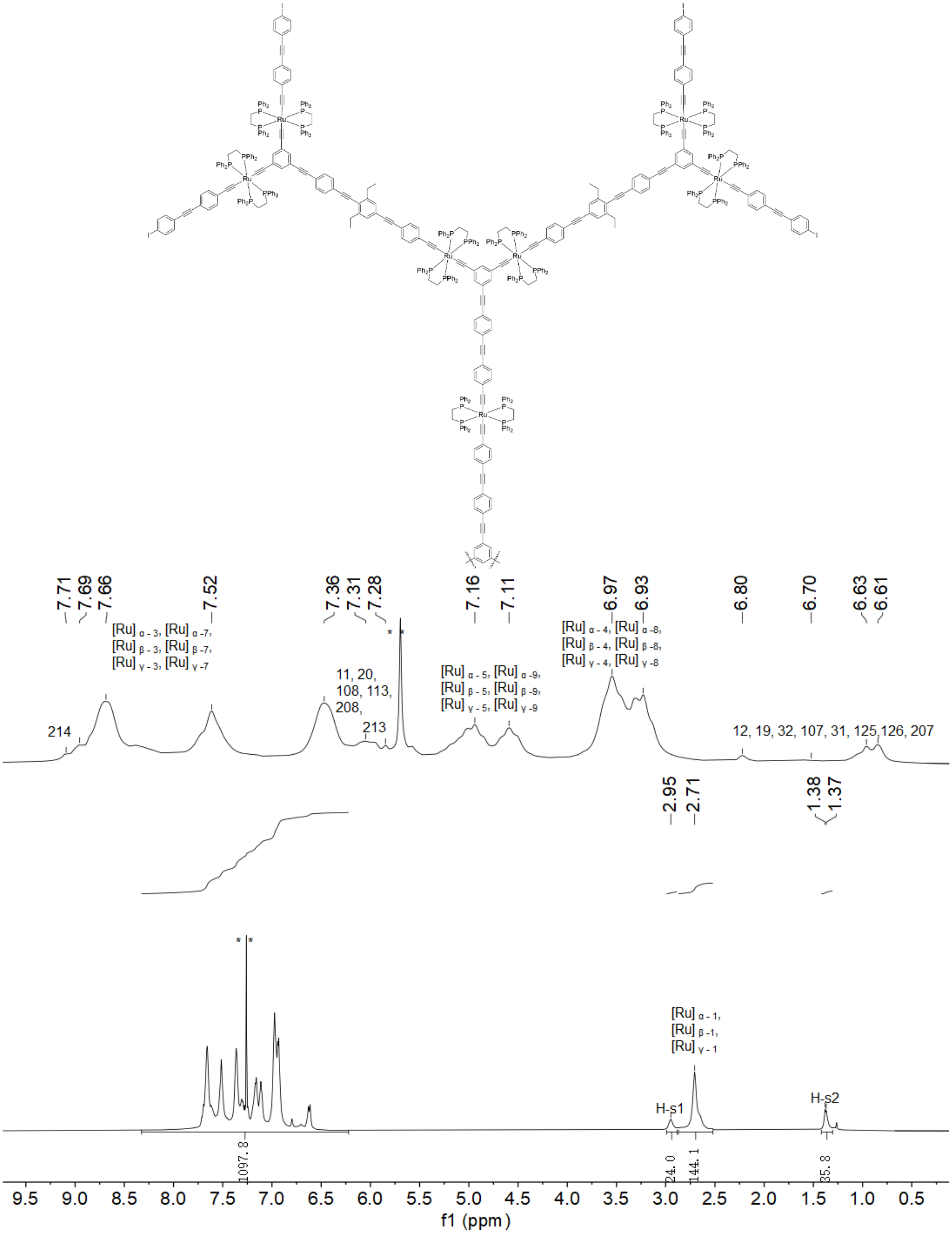


**Fig. S38** $^{1}$H NMR spectrum of **36** recorded in $CDCl_3$ at 600 MHz. The peak marked as ** corresponds to $CHCl_3$.

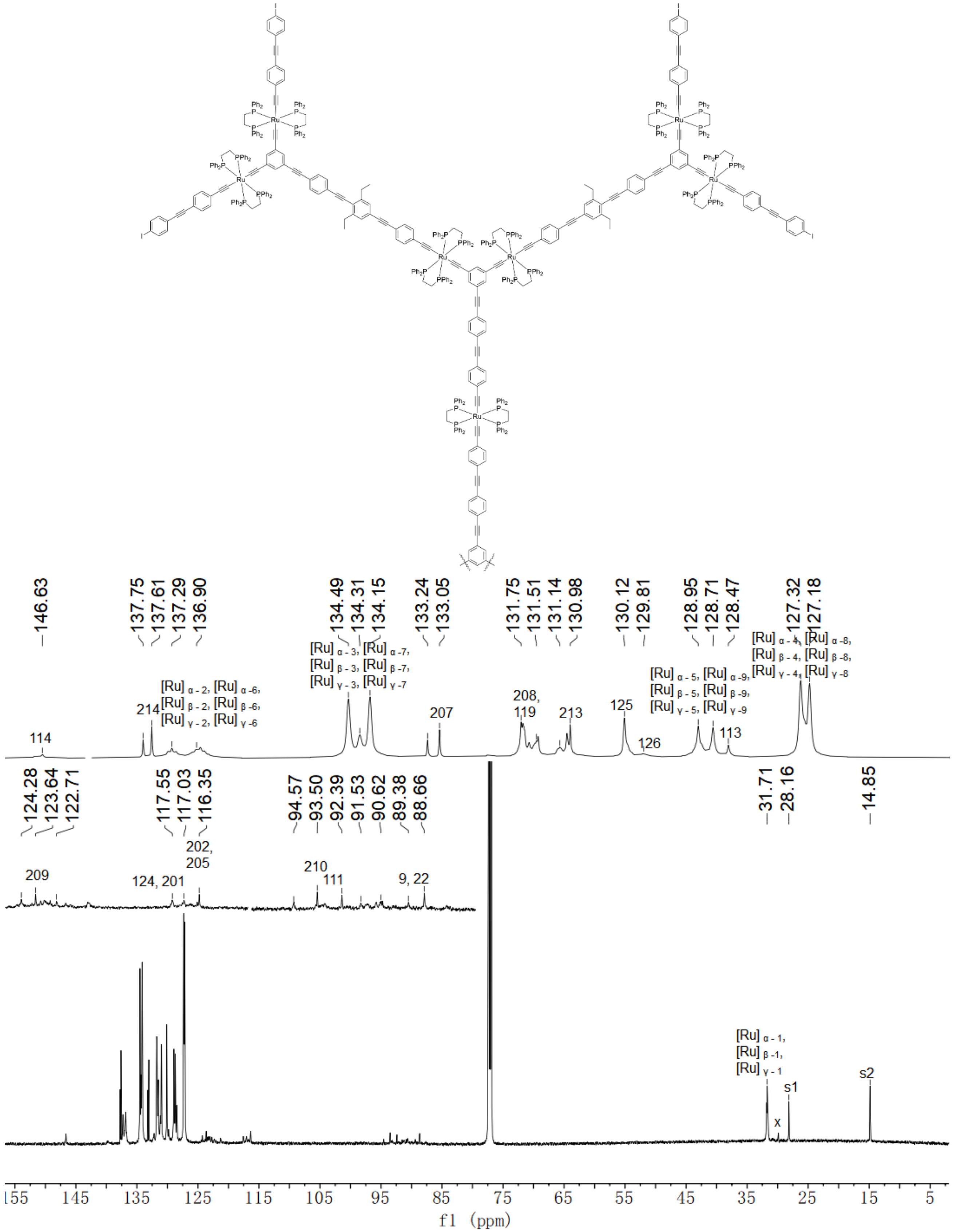


**Fig. S39** $^{13}C\{^1H\}$ NMR spectrum of **36** recorded in $CDCl_3$ at 151 MHz. The peak marked as ** corresponds to $CDCl_3$. The peak marked as x corresponds to grease.

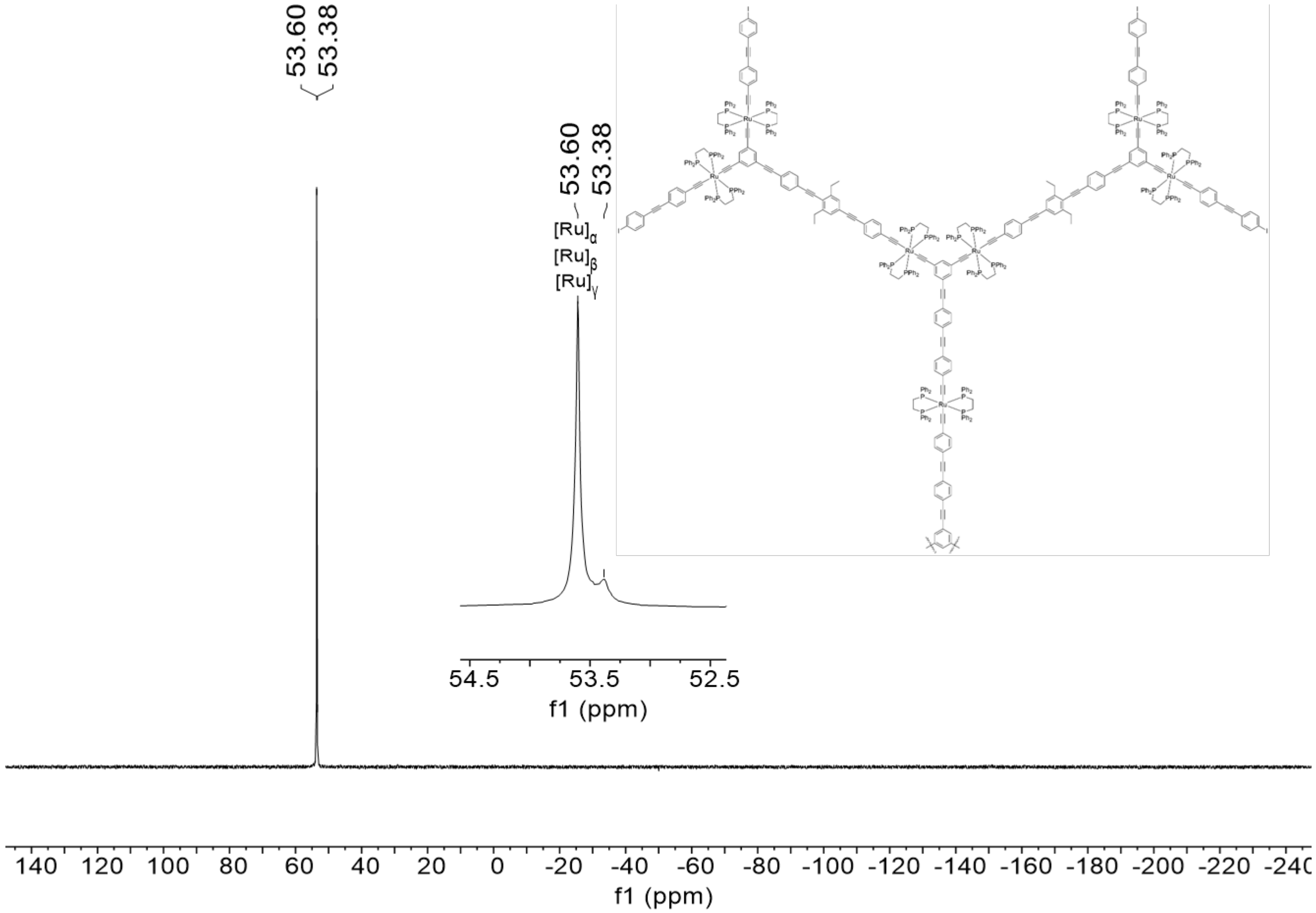


**Fig. S40** $^{31}P\{^1H\}$ NMR spectrum of **36** recorded in $CDCl_3$ at 162 MHz.

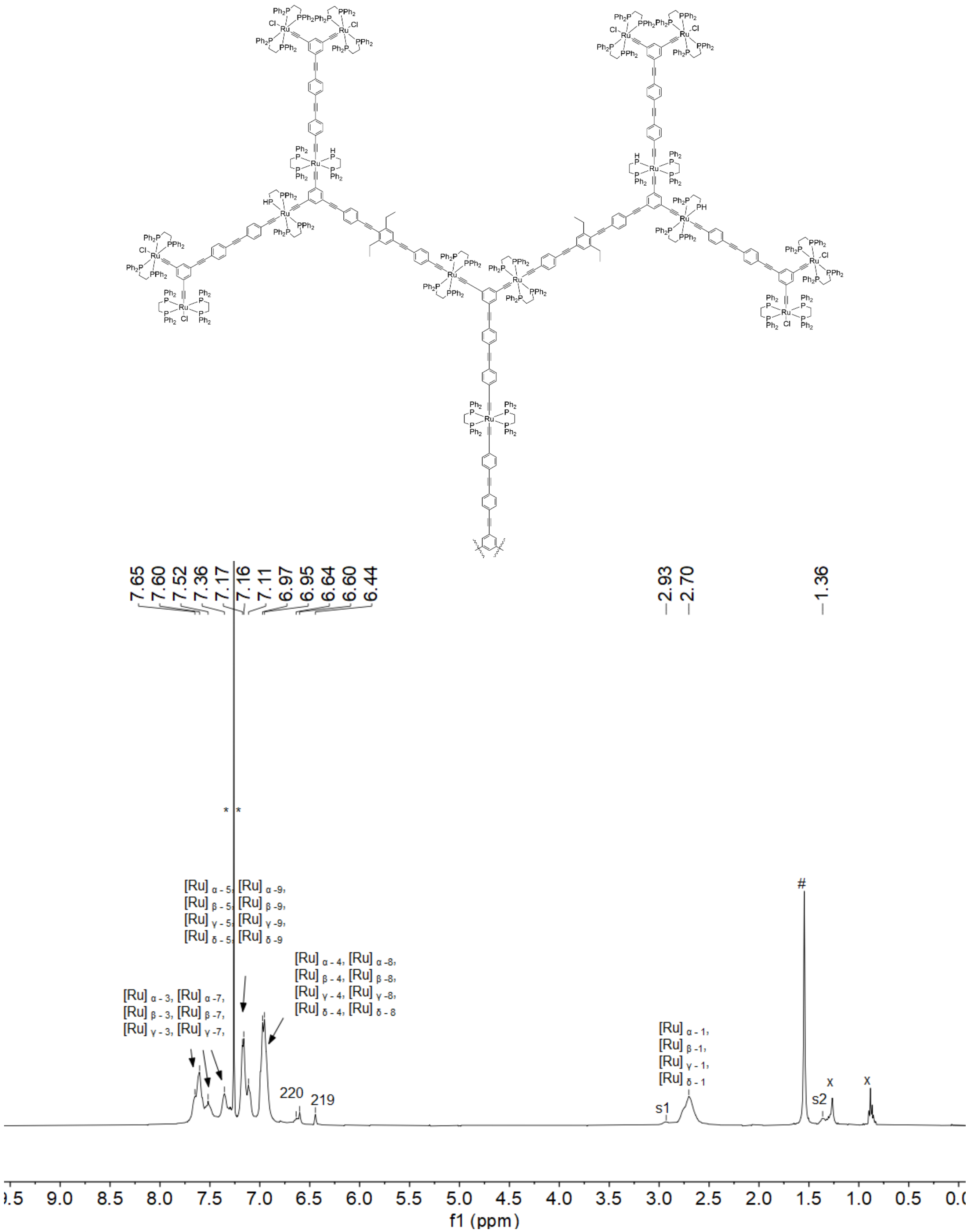


**Fig. S41** $^1$H NMR spectrum of **$3G_{22,03,02,00}$-Cl** recorded in $CDCl_3$ at 400 MHz. The peak marked as ** corresponds to $CHCl_3$. The peak marked as # corresponds to residual water. The peak marked as x corresponds to residual *n*-pentane.

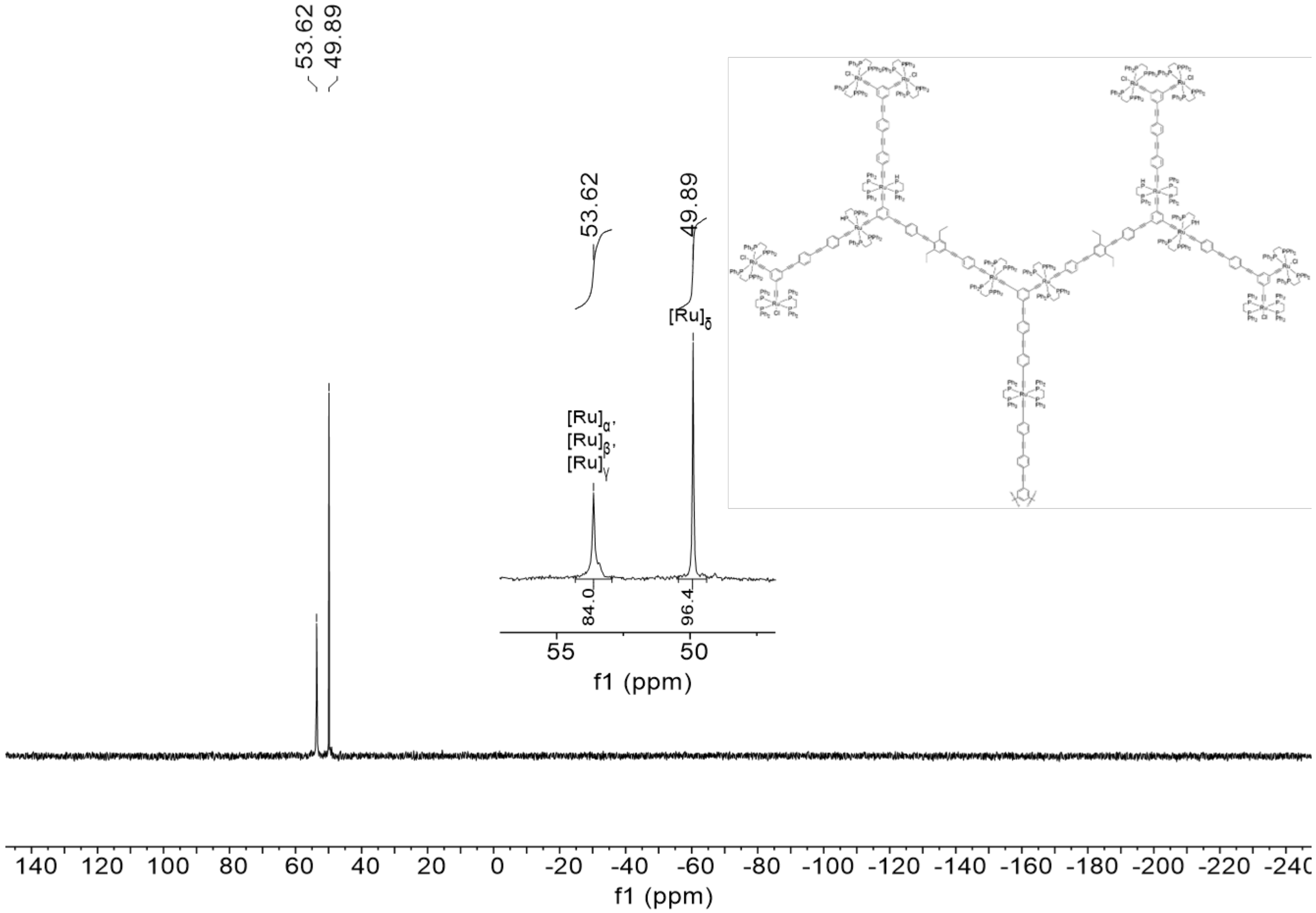


**Fig. S42** $^{31}P\{^{1}H\}$ NMR spectrum of **$3G_{22,03,02,00}$-Cl** recorded in $CDCl_3$ at 162 MHz.

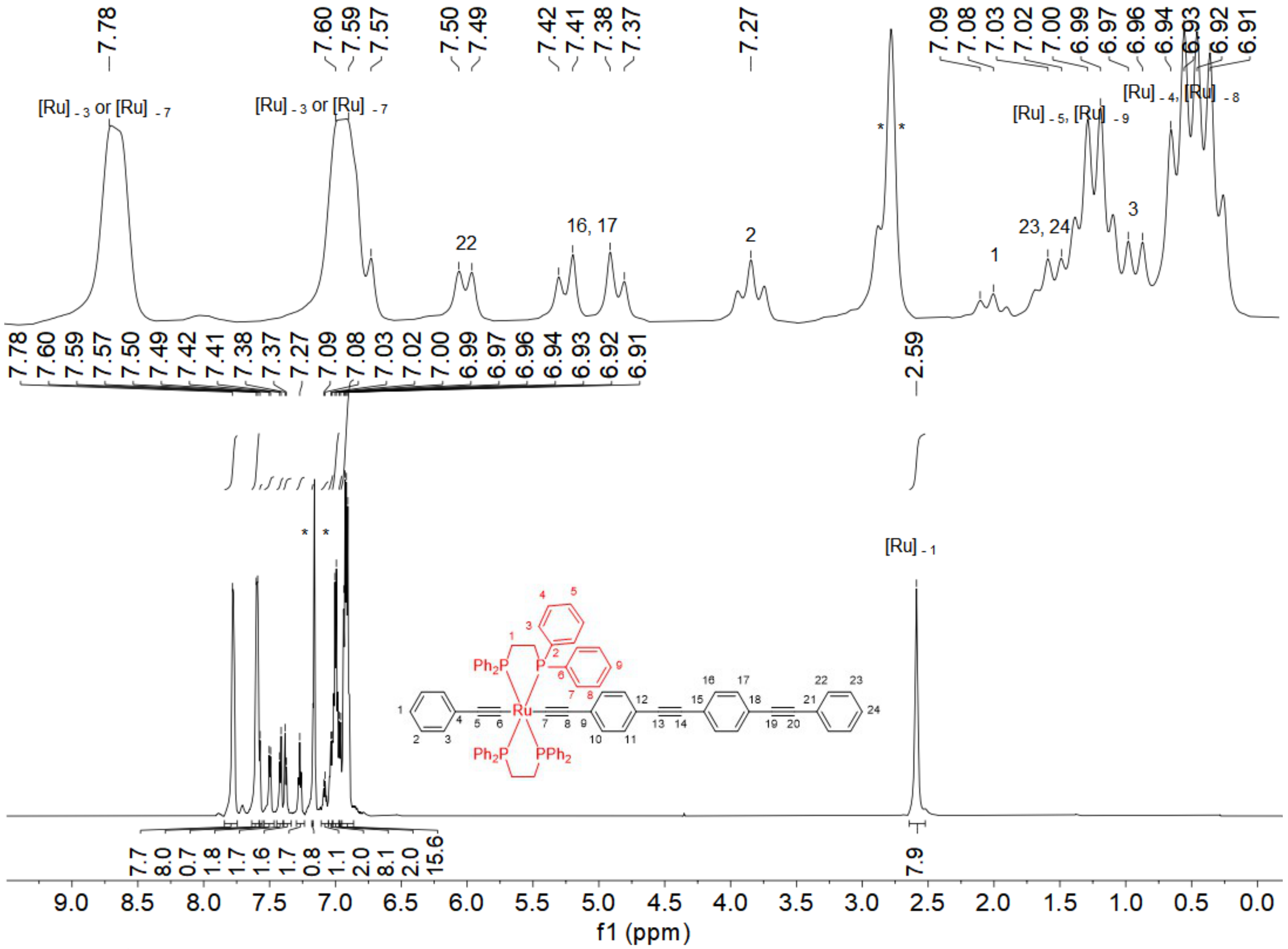

**Fig. S43** $^{1}$H NMR spectrum of **1-M-3(dppe)** recorded in $C_6D_6$ at 700 MHz. The peak marked as ** corresponds to $C_6D_5H$.

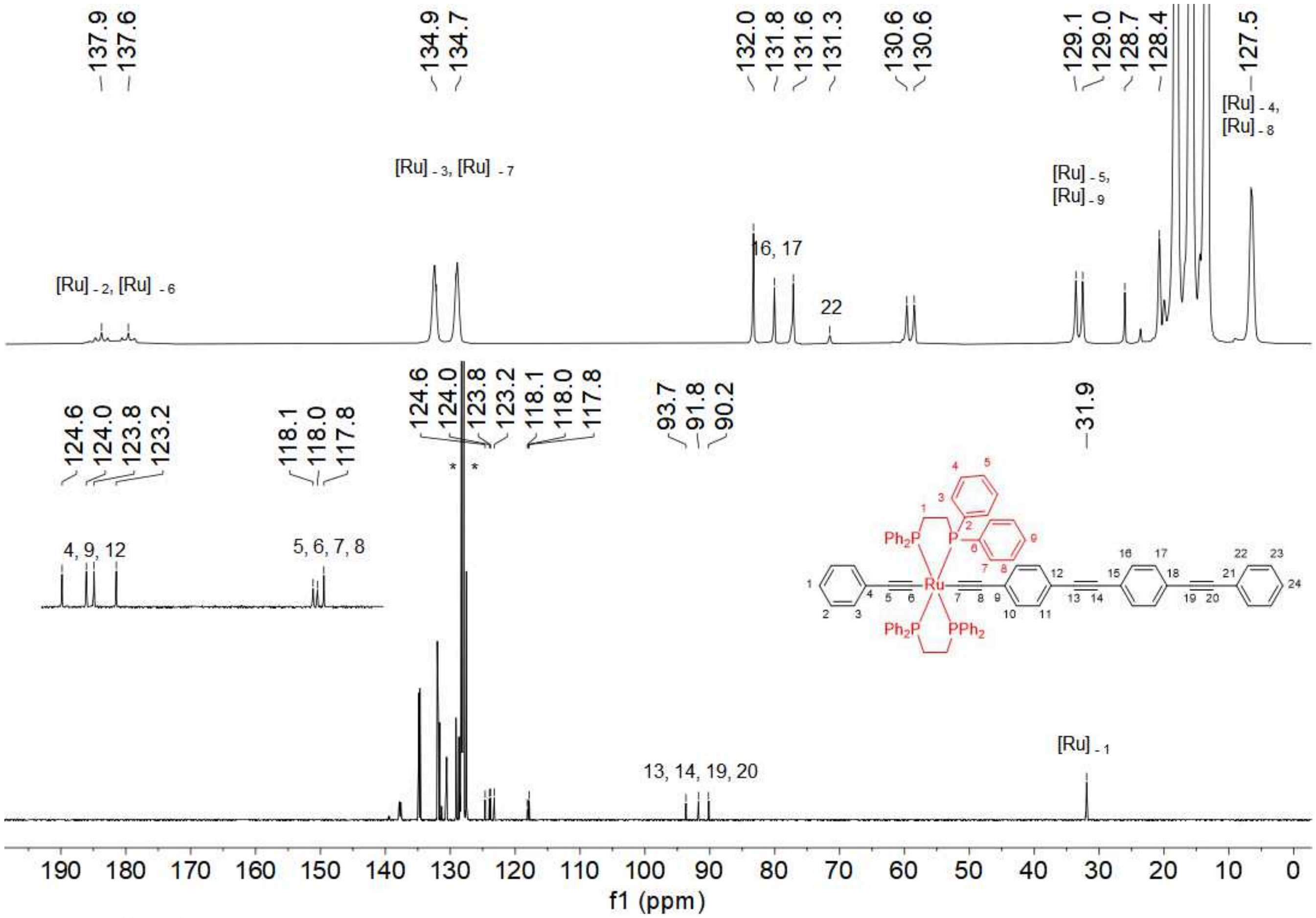


**Fig. S44** $^{13}C\{^{1}H\}$ NMR spectrum of **1-M-3(dppe)** recorded in $C_6D_6$ at 176 MHz. The peak marked as ** corresponds to $C_6D_5H$.

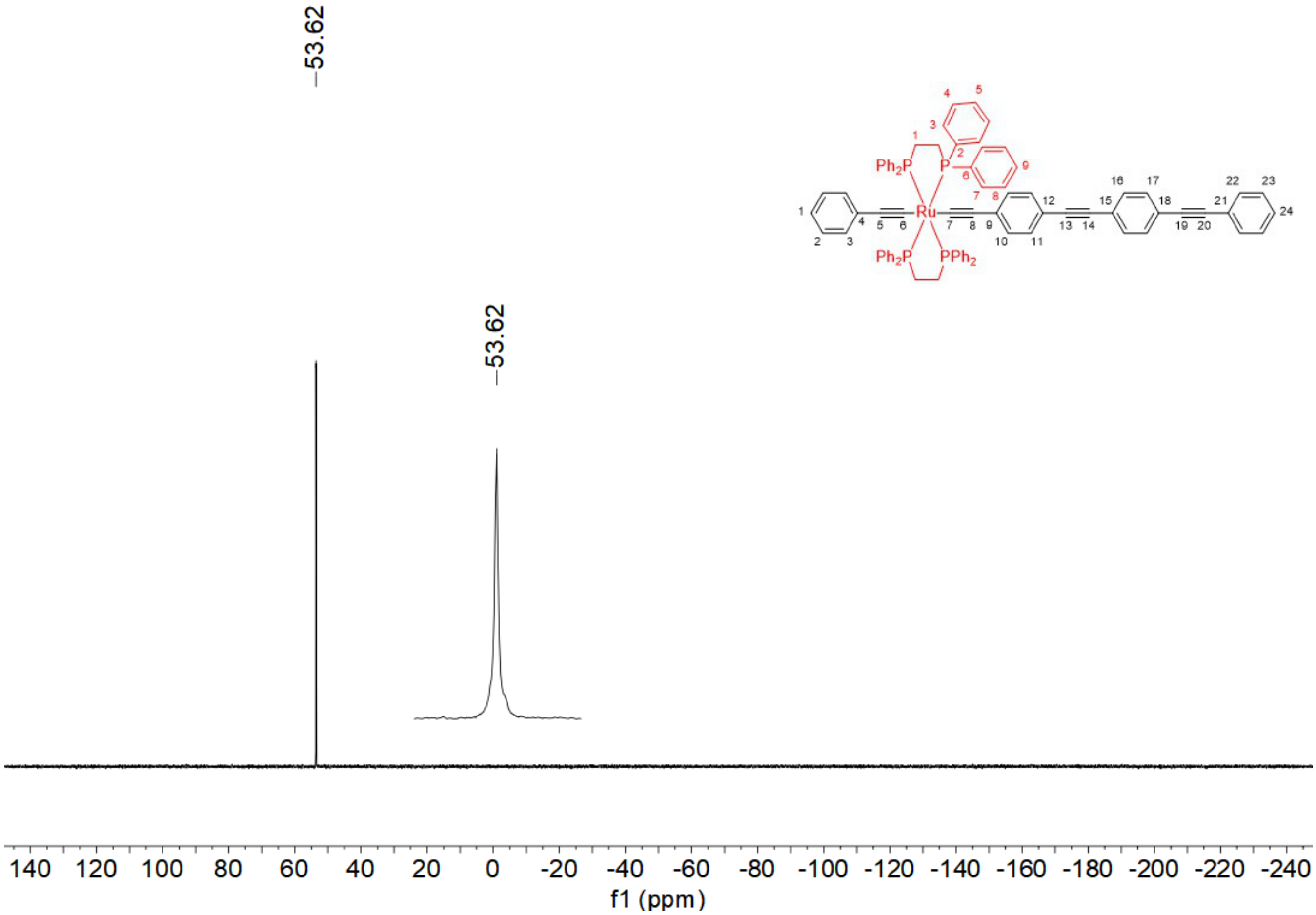


**Fig. S45** $^{31}P\{^{1}H\}$ NMR spectrum of **1-M-3(dppe)** recorded in $C_6D_6$ at 162 MHz.

## DOSY, SEC, TEM and MS studies

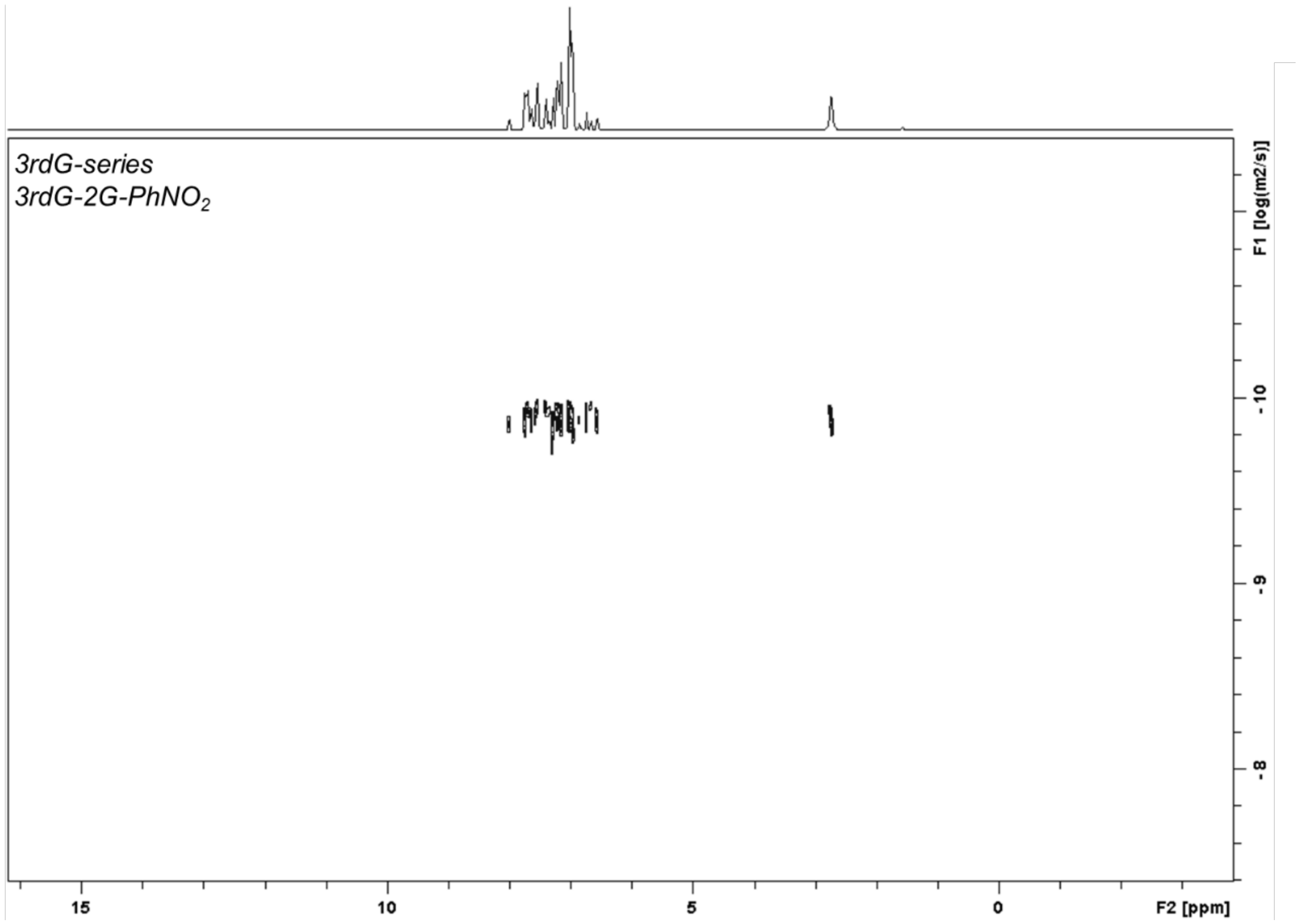


**Fig. S46** DOSY spectrum of **$2G_{22,03,01}$-$NO_2$** in $CDCl_3$ at 600 MHz and 298.0 K.

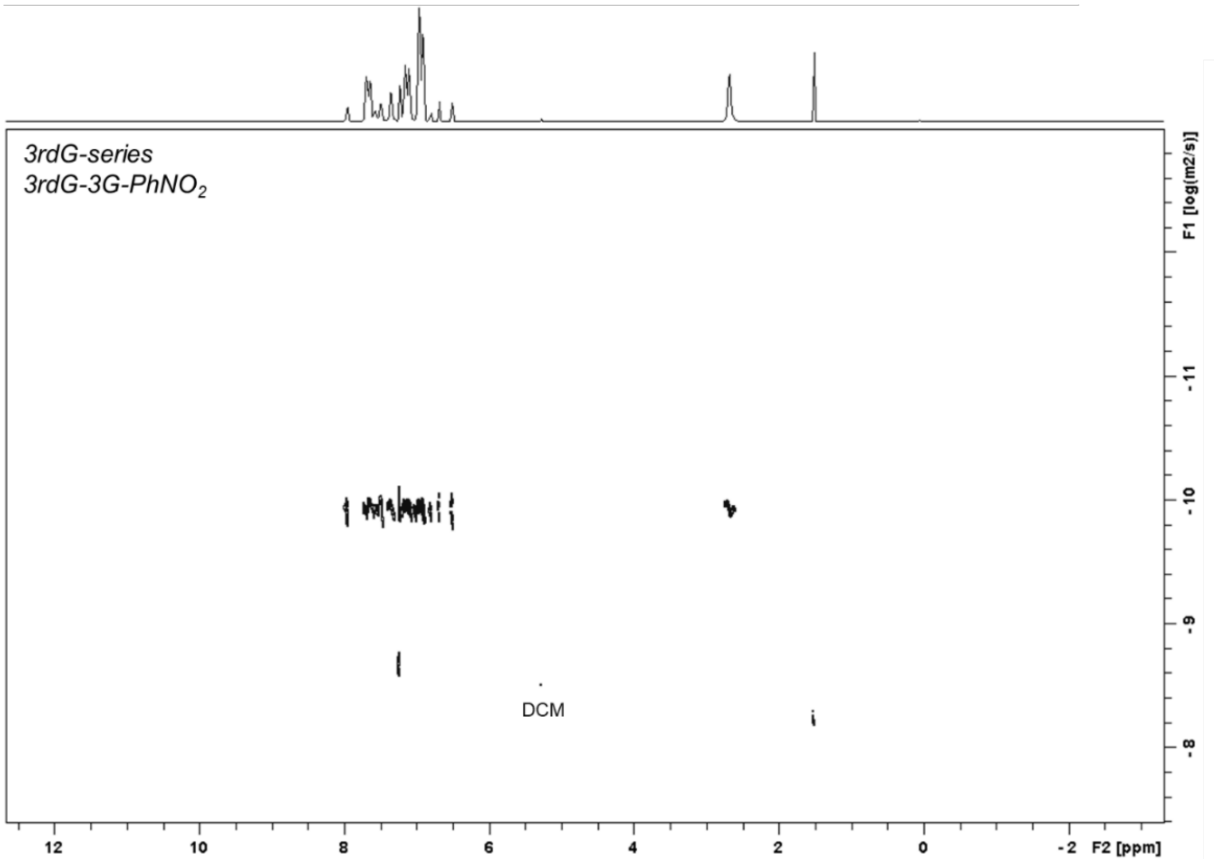

**Fig. S47** DOSY spectrum of $\mathbf{3G_{22,03,02,01}\text{-}NO_2}$ in $CDCl_3$ at 600 MHz and 298.0 K.

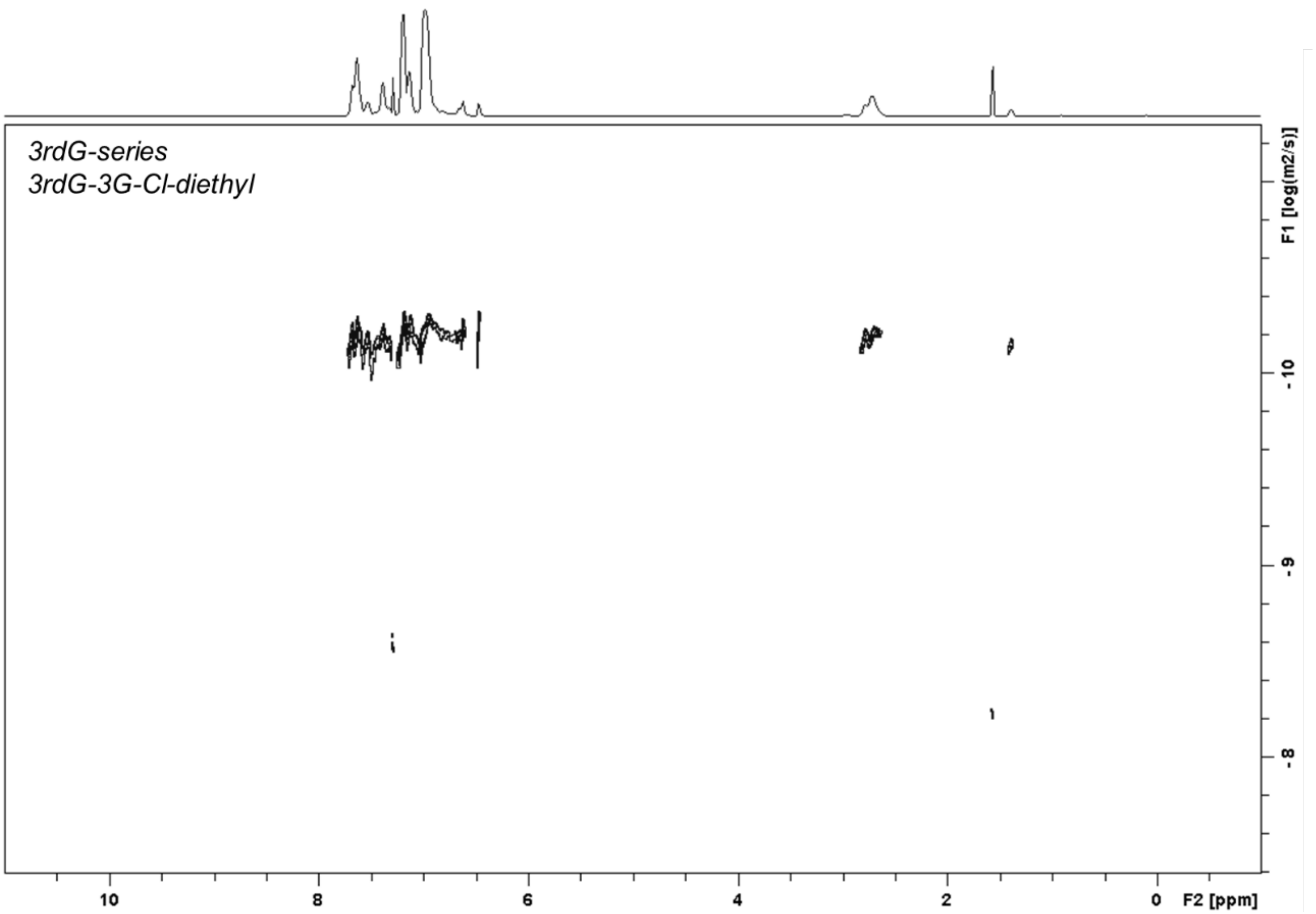

**Fig. S48** DOSY spectrum of **$3G_{22,03,02,00}$-Cl** in $CDCl_3$ at 600 MHz and 298.0 K.

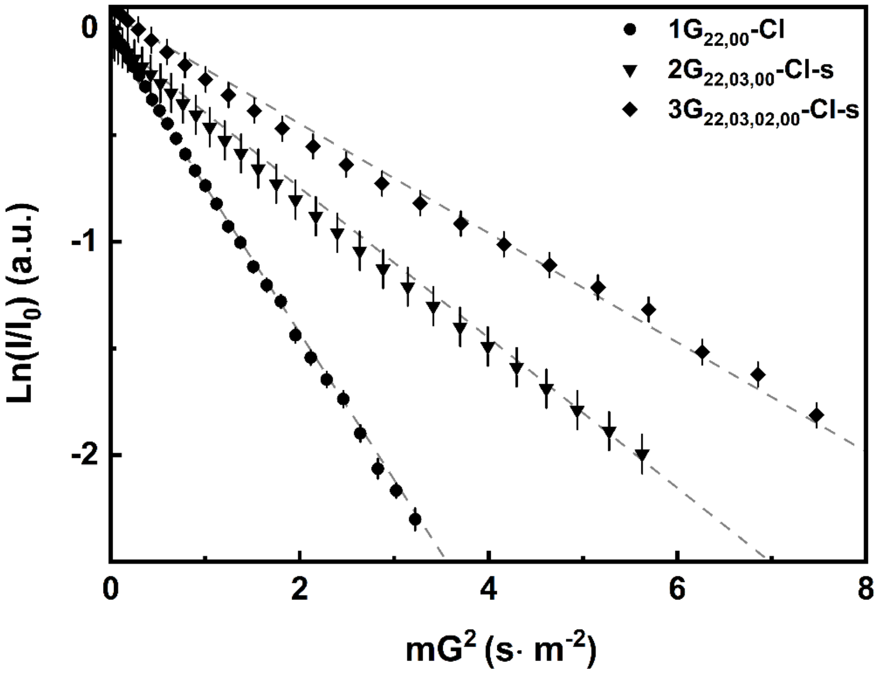


**Fig. S49** Stejskal-Tanner plots of the first-, second-, and third-generation dendrimers **$1G_{22,00}$-Cl**, **$2G_{22,03,00}$-Cl-s**, and **$3G_{22,03,02,00}$-Cl-s** in $CDCl_3$.

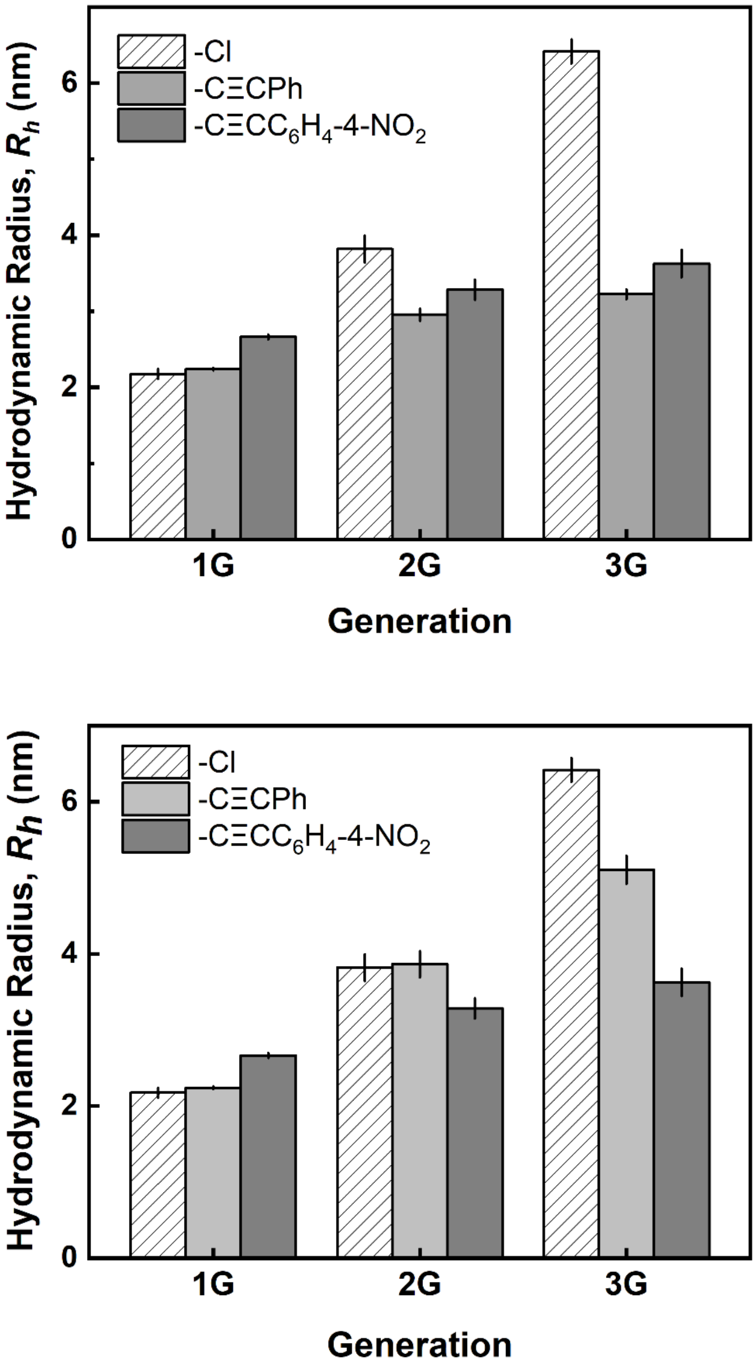


**Fig. S50** Hydrodynamic radii of first- (**1G**), second- (**2G**), and third- (**3G**) generation dendrimers with chlorido, phenylethynyl, and 4-nitrophenylethynyl peripheral groups in $CDCl_3$. In some cases, the dendrimers possess solubilizing substituents. **upper**) The second- and third-generation dendrimers with chlorido peripheral groups contain solubilizing substituents. **lower**) The second- and third- generation dendrimers with chlorido or phenylethynyl peripheral groups contain solubilizing substituents.

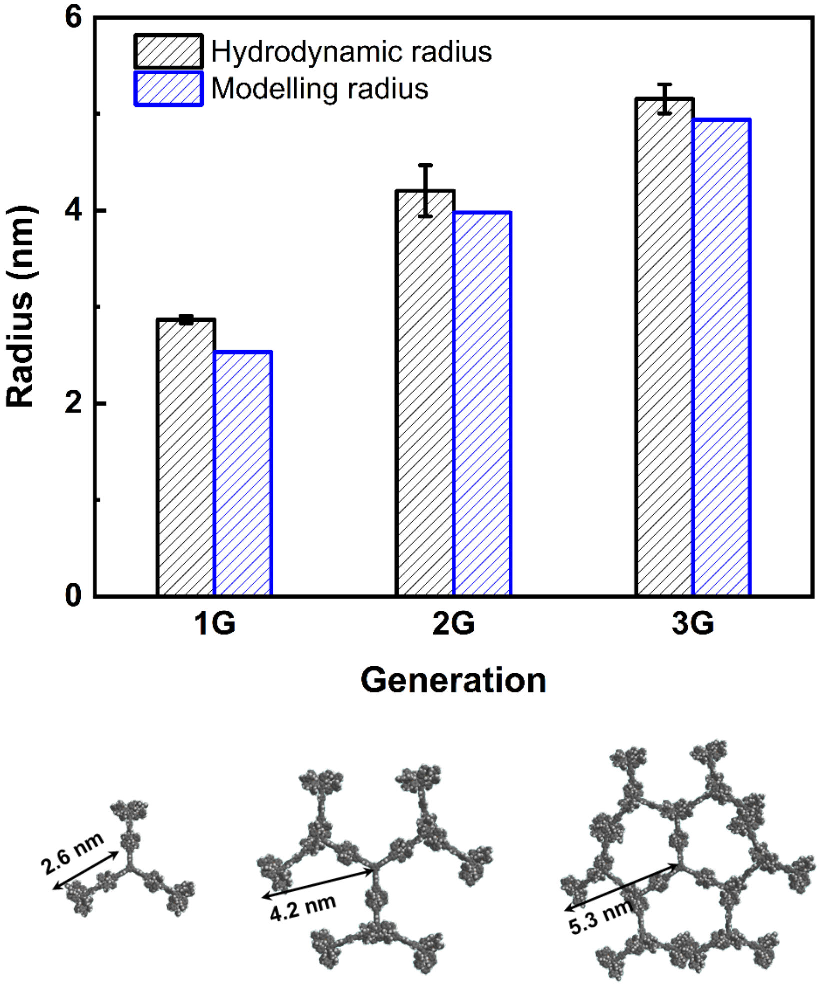


**Fig. S51** Comparison of hydrodynamic radius vs. modelled gyration radius of first- (**1G**$_{22,01}$), second- (**2G**$_{22,03,01}$), and third- (**3G**$_{22,03,02,01}$) generation dendrimers in $CDCl_3$.

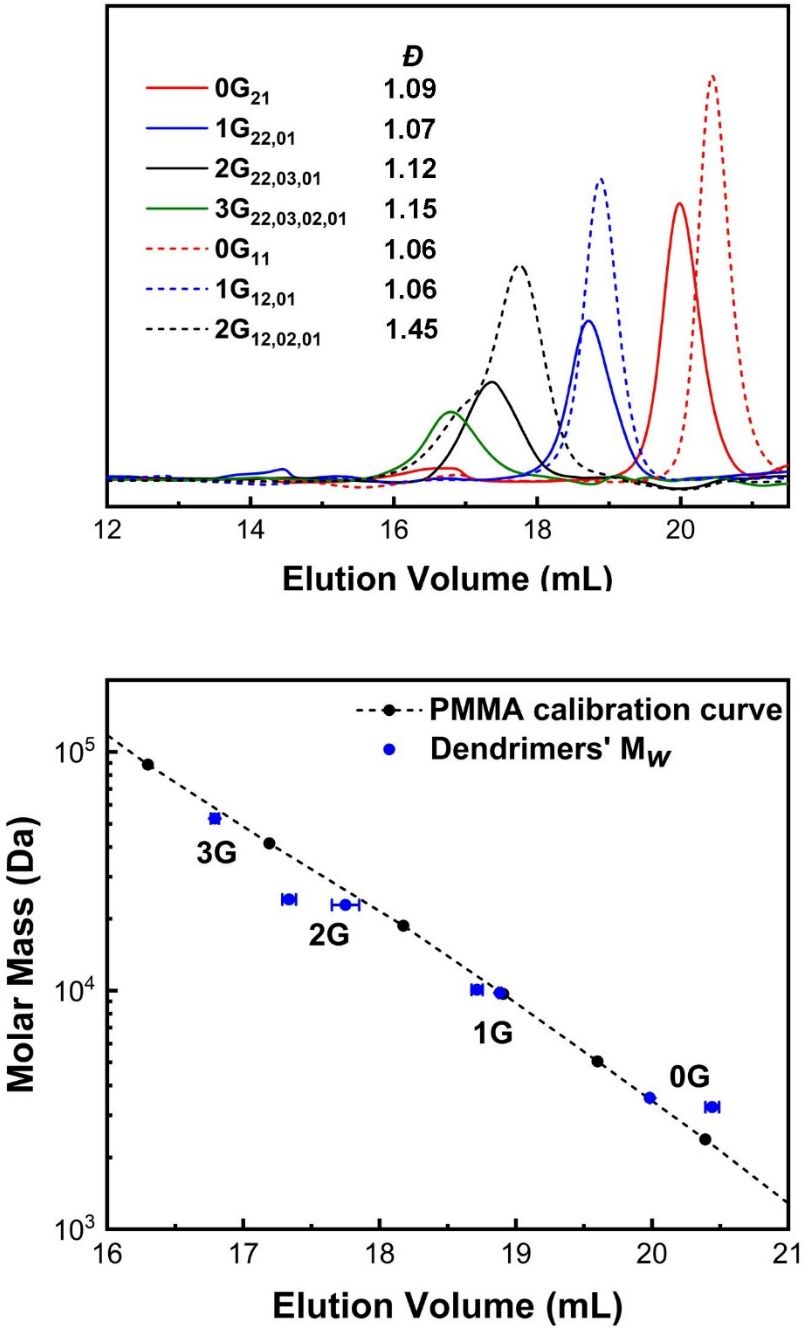


**Fig. S52** SEC plot of average molecular mass vs elution volume for the linear poly(methyl methacrylate) standards compared with the molecular masses of the dendrimers plotted vs their experimental SEC elution volumes. The low concentrations used to obtain the SEC traces are known to result in signal fluctuations.[42-46]

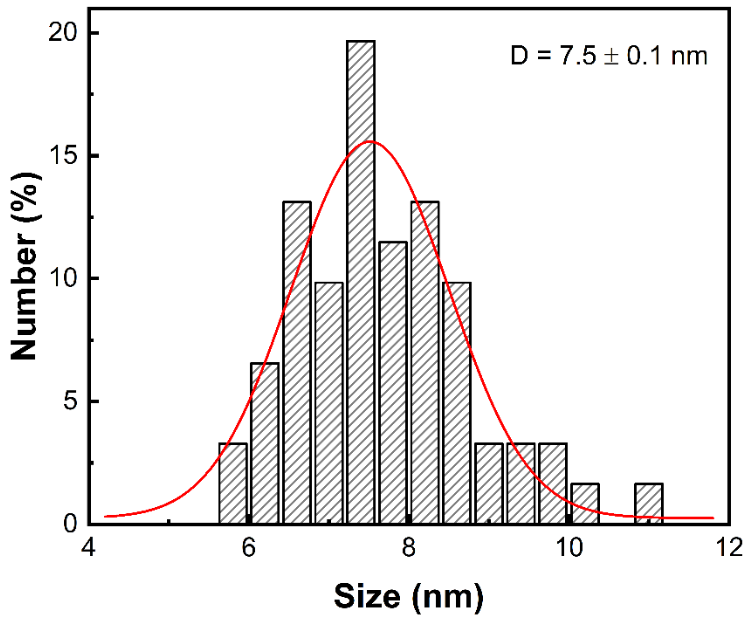


**Fig. S53** TEM size distribution of dendrimer $\mathbf{2G_{22,02,01}}$ is centered about diameter ca. 7.5 nm.

## UV-vis-NIR studies

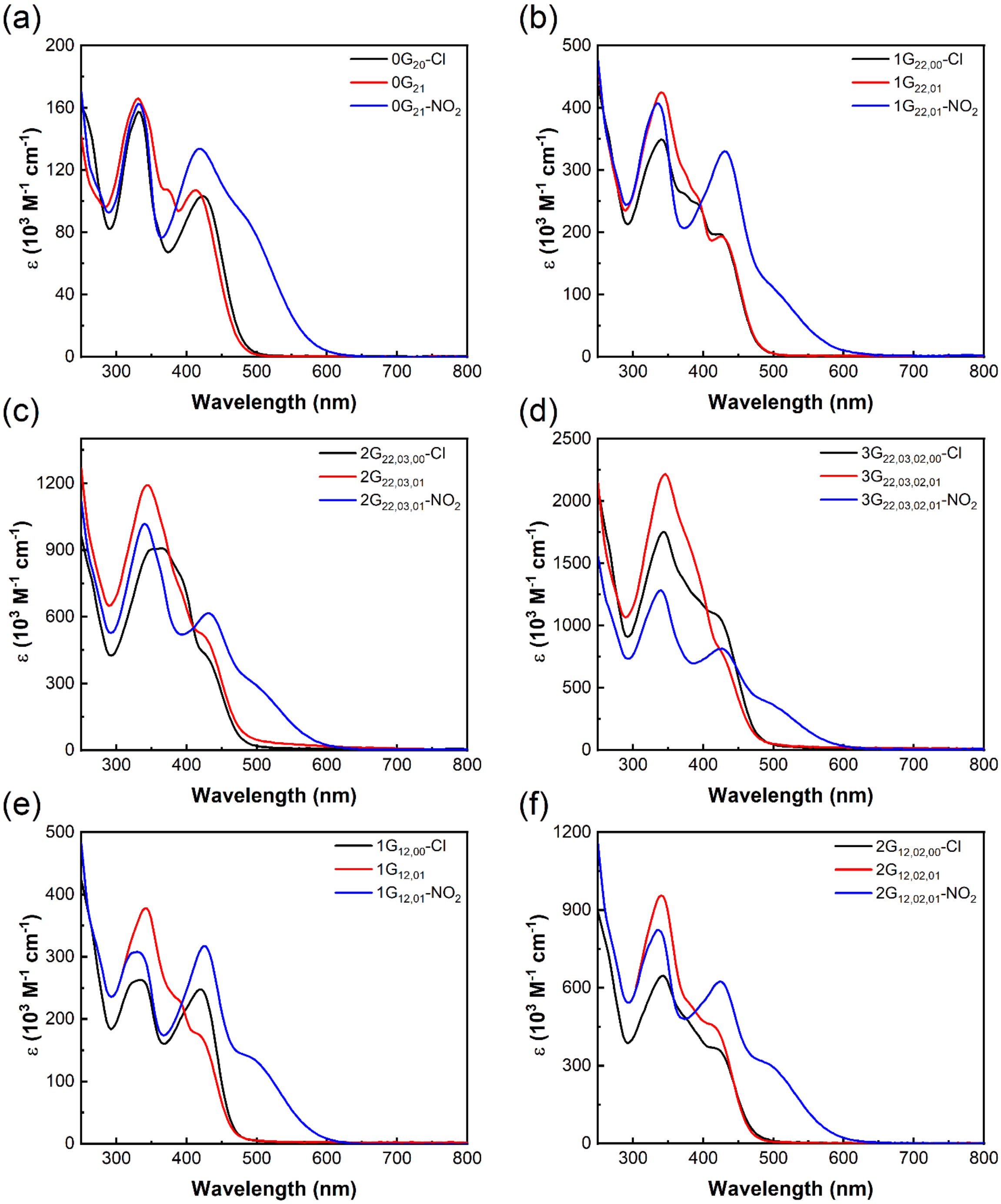


**Fig. S54** UV-vis-NIR spectra of the dendrimers. (a) 0th-generation dendrimers with different peripheral groups (chlorido, phenylalkynyl, 4-nitrophenylalkynyl); (b) and (e) 1st-generation dendrimers with different peripheral groups; (c) and (f) 2nd-generation dendrimers with different peripheral groups; (d) 3rd-generation dendrimers with different peripheral groups.

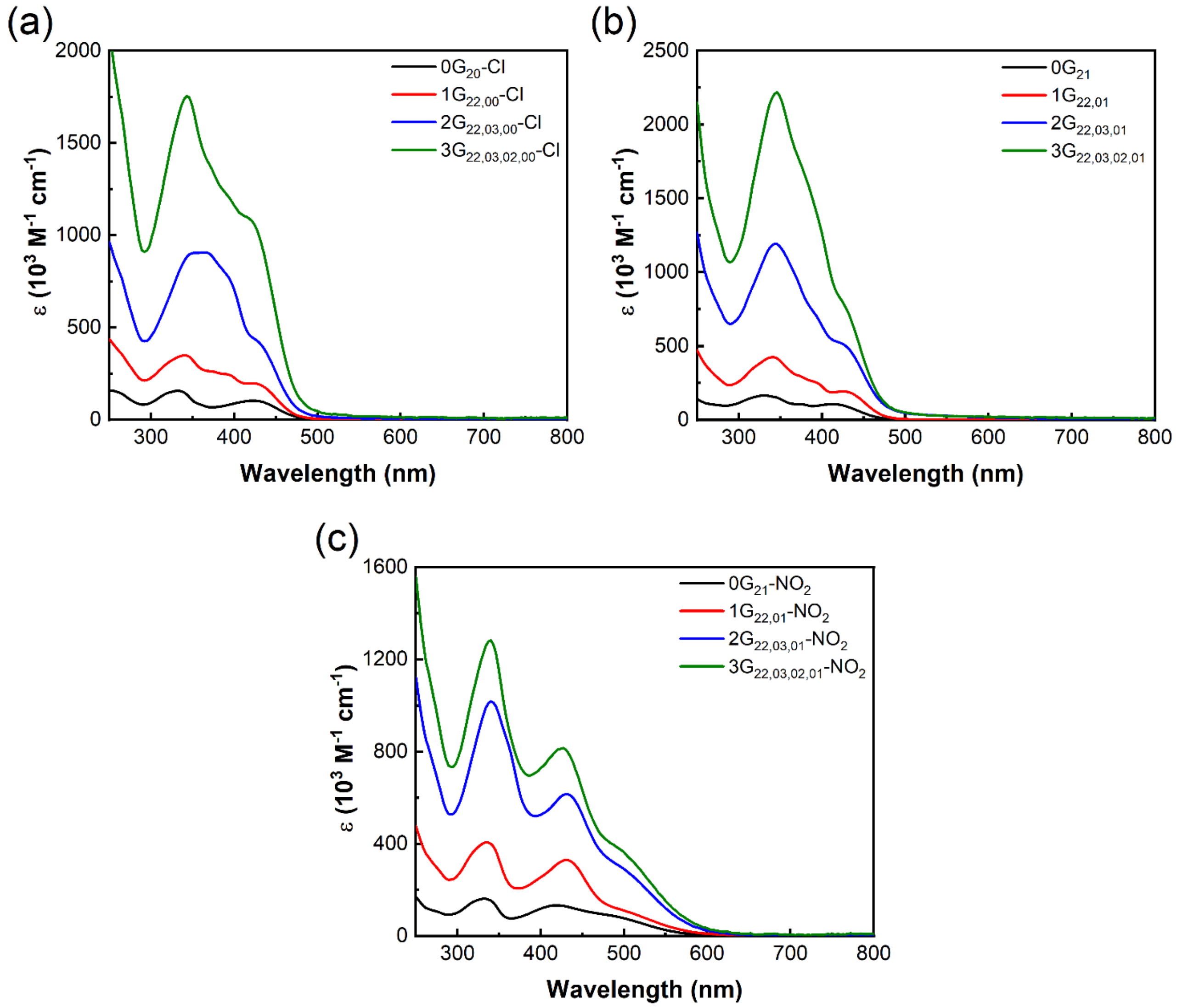


**Fig. S55** UV-vis-NIR spectra of ruthenium dendrimers. (a) Dendrimers with chlorido peripheral groups; (b) dendrimers with phenylalkynyl peripheral groups; (c) dendrimers with 4-nitrophenylalkynyl peripheral groups.

**Table S2.** Linear optical absorption and nonlinear absorption cross-section maxima of dendrimer synthesis intermediates.[a]

| Complex | $\lambda_{, max}$[b] [$\varepsilon$][c] | $\sigma_2$[d] ($\lambda_{max}$)[b] | $\sigma_2$/M[e] ($\lambda_{max}$)[b] | $\sigma_3$[f] ($\lambda_{max}$)[b] | $\sigma_3$/M[g] ($\lambda_{max}$)[b] | $\sigma_4$[h] ($\lambda_{max}$)[b] | $\sigma_4$/M[i] ($\lambda_{max}$)[b] | $\sigma_5$[j] ($\lambda_{max}$)[b] | $\sigma_5$/M[g] ($\lambda_{max}$)[b] |
|---|---|---|---|---|---|---|---|---|---|
| **16** | 346 [124] | 1600 (850) | 0.65 (850) | 2700 (1250) | 1.09 (1250) | n/a | n/a | n/a | n/a |
| **17** | 349 [118] | 1200 (750) | 0.47 (750) | 2600 (1250) | 1.03 (1250) | n/a | n/a | n/a | n/a |
| **18** | 358 [185] | 1800 (900) | 0.58 (900) | 3800 (1250) | 1.22 (1250) | n/a | n/a | n/a | n/a |
| **20** | 346 [150] | 2400 (900) | 0.67 (900) | 4500 (1250) | 1.73 (1250) | n/a | n/a | n/a | n/a |
| **22** | 346 [138]<br>484 [46] | 3100 (850) | 1.15 (850) | 6100 (1250) | 2.27 (1250) | n/a | n/a | n/a | n/a |
| **24** | 343 [331] | 17800 (950) | 2.47 (950) | 8200 (1250) | 1.14 (1250) | n/a | n/a | n/a | n/a |
| **26** | 340 [293]<br>424 [130] | 32300 (900) | 4.44 (900) | 13800 (1250) | 1.90 (1250) | n/a | n/a | n/a | n/a |
| **35** | 349 [662] | 38400 (950) | 3.01 (950) | 22000 (1300) | 1.72 (1300) | 1700 (1730) | 0.13 (1730) | n/a | n/a |
| **36** | 364 [1240] | 112600 (1100) | 4.08 (1100) | 76300 (1250) | 2.77 (1250) | 1300 (1650) | 0.047 (1650) | n/a | n/a |

[a] $CH_2Cl_2$. [b] nm. [c] $10^4$ L mol$^{-1}$ cm$^{-1}$. [d] GM = $10^{-50}$ cm$^4$ s photon$^{-1}$. [e] GM mol g$^{-1}$ = $10^{-50}$ cm$^4$ s mol g$^{-1}$ photon$^{-1}$. [f] $10^{-80}$ cm$^6$ s$^2$ photon$^{-2}$. [g] $10^{-80}$ cm$^6$ s$^2$ mol g$^{-1}$ photon$^{-2}$. [h] $10^{-110}$ cm$^8$ s$^3$ photon$^{-3}$. [i] $10^{-110}$ cm$^8$ s$^3$ mol g$^{-1}$ photon$^{-3}$. [j] $10^{-140}$ cm$^{10}$ s$^4$ photon$^{-4}$. [k] $10^{-140}$ cm$^{10}$ s$^4$ mol g$^{-1}$ photon$^{-4}$.

## Computational studies - general comments.

To rationalize the linear and nonlinear absorption observations, DFT and TD-DFT calculations were undertaken on model complexes (Fig. 2). For the sake of computational expediency, model complexes **1-M-n**, **2-M-n** and **3-M-3** possess 1,2-bis(phosphino)ethane bidentate diphosphine co-ligands in place of the 1,2-bis(diphenylphosphino)ethane of the experimental complexes. The molecular structure optimizations were performed with the Gaussian 16 program.[47] The geometry optimizations of the investigated structures were performed at the PBE0/6-31G(d) level of theory (with no symmetry constraints on nuclear coordinates), with the D3BJ dispersion correction, and with the polarizable continuum model (PCM) of solvent $CH_2Cl_2$. Frequency calculations were carried out at the same level of theory to identify all the stationary points as minima (zero imaginary frequencies) and to provide the thermal correction to free energies at 298.15 K and 1 atm.

**xyz Coordinates for Calculated Structures**

**Table S3.** Geometry optimization and frequency calculation information for **1-M-1**.

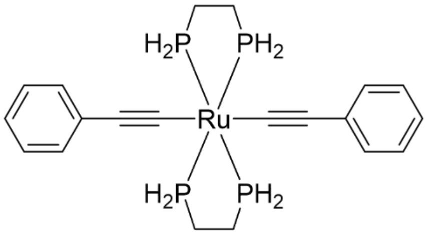


| | | | |
|---|---|---|---|
| SCF: E(RPBE1PBE) = -2236.58978578 a.u. | | | |
| Electronic spatial extent (a.u.): <R**2> = 15879.9658 | | | |
| Dipole moment (field-independent basis, Debye): | | | |
| X = -0.0014 | Y = -0.0000 | Z = 0.0000 | Tot = 0.0014 |
| Dipole = -5.60082123D-04 | -3.01034083D-06 | 8.24271756D-06 | |
| Cartesian Forces: Max 0.000001202 | | RMS 0.000000314 | |
| Number of Imaginary Freq. = 0 | | | |
| Symmetry c1 | | | |

| Atom | X | Y | Z |
|---|---|---|---|
| Ru | 0.000056 | 0.000001 | 0.000011 |
| P | 0.022695 | 1.708209 | -1.53762 |
| P | -0.02282 | 1.708014 | 1.537848 |
| C | 0.354381 | 3.319816 | -0.67553 |
| H | 0.045802 | 4.168108 | -1.29388 |
| H | 1.440891 | 3.377383 | -0.53845 |
| C | -0.35521 | 3.31958 | 0.675977 |
| H | -0.047 | 4.167935 | 1.294424 |
| H | -1.44174 | 3.376668 | 0.538904 |
| P | -0.02272 | -1.70802 | -1.53783 |
| P | 0.022819 | -1.70821 | 1.537638 |
| C | 0.354742 | -3.31976 | 0.675555 |
| H | 0.0463 | -4.1681 | 1.293905 |
| H | 1.441259 | -3.37716 | 0.538461 |
| C | -0.35486 | -3.31963 | -0.67595 |
| H | -0.04654 | -4.16794 | -1.2944 |
| H | -1.44139 | -3.37689 | -0.53886 |
| C | -2.04722 | -0.00003 | 0.000012 |
| C | -3.28149 | -3.5E-05 | 0.00001 |
| C | 2.04733 | 0.000032 | -2E-06 |
| C | 3.281595 | 0.000038 | -0.00001 |
| C | -4.70711 | -3.9E-05 | 0.000004 |
| C | -5.43457 | 1.019274 | -0.64592 |
| C | -5.43457 | -1.01936 | 0.645914 |
| C | -6.82495 | 1.016965 | -0.64419 |
| H | -4.88972 | 1.813368 | -1.14979 |
| C | -6.82495 | -1.01706 | 0.644158 |
| H | -4.88972 | -1.81345 | 1.14979 |

| | | | |
|---|---|---|---|
| C | -7.52962 | -5.3E-05 | -2.4E-05 |
| H | -7.36299 | 1.815119 | -1.14988 |
| H | -7.36299 | -1.81523 | 1.149833 |
| C | 4.707218 | 0.000043 | -2.2E-05 |
| C | 5.434676 | -1.01928 | -0.64593 |
| C | 5.434682 | 1.019368 | 0.645878 |
| C | 6.825056 | -1.01698 | -0.64419 |
| H | 4.889826 | -1.81338 | -1.14979 |
| C | 6.825062 | 1.017066 | 0.64413 |
| H | 4.889836 | 1.813466 | 1.149744 |
| C | 7.529733 | 0.000043 | -3.5E-05 |
| H | 7.363093 | -1.81515 | -1.14987 |
| H | 7.363104 | 1.815229 | 1.149799 |
| H | 8.616366 | 0.000043 | -0.00004 |
| H | 1.152495 | -1.9916 | -2.27195 |
| H | -0.95564 | -1.74949 | -2.59748 |
| H | 0.955723 | 1.750007 | -2.59716 |
| H | -1.1525 | 1.991603 | -2.27184 |
| H | 1.152418 | 1.991748 | 2.271888 |
| H | -0.95567 | 1.749382 | 2.59756 |
| H | -1.15238 | -1.99175 | 2.271803 |
| H | 0.955801 | -1.7499 | 2.597221 |
| H | -8.61626 | -5.9E-05 | -3.4E-05 |

**Table S4.** Geometry optimization and frequency calculation information for **1-M-2**.

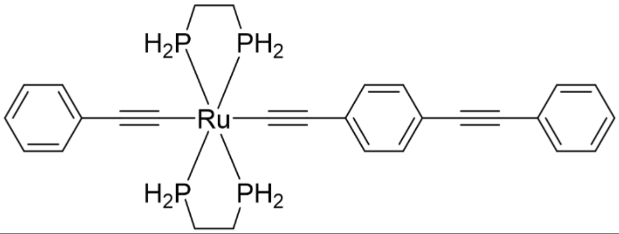

| SCF: E(RPBE1PBE) = -2543.44466856 a.u. |
|---|
| Electronic spatial extent (a.u.): <R**2> = 39676.9411 |
| Dipole moment (field-independent basis, Debye): |
| X = 1.5806 Y = -0.0001 Z = -0.0002 Tot = 1.5806 |
| Dipole = 6.21850067D-01 -3.34226743D-05 -8.36510854D-05 |
| Cartesian Forces: Max 0.000005021 RMS 0.0000013484 |
| Number of Imaginary Freq = 0 |
| Symmetry c1 |

| Atom | X | Y | Z |
|---|---|---|---|
| Ru | 2.116516 | 0.000001 | 0.000022 |
| P | 2.14513 | -1.80244 | 1.426865 |
| P | 2.110222 | -1.60776 | -1.64403 |
| C | 2.50561 | -3.35035 | 0.465813 |
| H | 2.208108 | -4.24104 | 1.027345 |
| H | 3.593441 | -3.38167 | 0.330728 |
| C | 1.801409 | -3.27547 | -0.88656 |
| H | 2.126043 | -4.07786 | -1.55582 |
| H | 0.715448 | -3.35954 | -0.75899 |
| P | 2.110226 | 1.607762 | 1.644067 |
| P | 2.144876 | 1.802435 | -1.42683 |
| C | 2.505176 | 3.350417 | -0.46581 |
| H | 2.20748 | 4.241047 | -1.02732 |
| H | 3.593013 | 3.381923 | -0.33081 |
| C | 1.801093 | 3.27542 | 0.886618 |
| H | 2.12565 | 4.07787 | 1.555859 |
| H | 0.715109 | 3.359314 | 0.759129 |
| C | 0.075144 | -1.7E-05 | 0.000121 |
| C | -1.16022 | -7E-06 | 0.000168 |
| C | 4.164711 | 0.000006 | -2.6E-05 |
| C | 5.398732 | -1.4E-05 | -3.2E-05 |
| C | -2.58057 | -1.9E-05 | 0.000111 |
| C | -3.31172 | -1.02442 | 0.638302 |
| C | -3.31167 | 1.024402 | -0.6381 |
| C | -4.69609 | -1.02616 | 0.639135 |
| H | -2.7696 | -1.82321 | 1.136705 |
| C | -4.69605 | 1.026176 | -0.63898 |
| H | -2.76952 | 1.82318 | -1.13648 |
| C | -5.41712 | 0.000016 | 0.000064 |
| H | -5.23871 | -1.82495 | 1.136795 |

| | | | |
|---|---|---|---|
| H | -5.23863 | 1.824984 | -1.13666 |
| C | 6.824441 | 0.000001 | -7.1E-05 |
| C | 7.551544 | 1.018677 | 0.646999 |
| C | 7.551549 | -1.01865 | -0.64718 |
| C | 8.941922 | 1.016378 | 0.64522 |
| H | 7.006679 | 1.812217 | 1.151706 |
| C | 8.941928 | -1.0163 | -0.64546 |
| H | 7.006694 | -1.8122 | -1.15187 |
| C | 9.646473 | 0.00005 | -0.00013 |
| H | 9.480003 | 1.813949 | 1.151735 |
| H | 9.480015 | -1.81385 | -1.152 |
| H | 10.7331 | 0.000067 | -0.00016 |
| C | -6.83647 | 0.000049 | 0.000057 |
| C | -8.05381 | 0.00007 | 0.000045 |
| C | -9.47551 | 0.000009 | -0.00003 |
| C | -10.1895 | -1.03033 | 0.637966 |
| C | -10.1895 | 1.03029 | -0.63809 |
| C | -11.5792 | -1.02591 | 0.635116 |
| H | -9.64244 | -1.82794 | 1.13192 |
| C | -11.5792 | 1.025766 | -0.63537 |
| H | -9.64248 | 1.827943 | -1.13199 |
| C | -12.279 | -9.8E-05 | -0.00016 |
| H | -12.1193 | -1.82749 | 1.131415 |
| H | -12.1194 | 1.827312 | -1.13172 |
| H | -13.3654 | -0.00014 | -0.00021 |
| H | 3.289749 | 1.824692 | 2.392996 |
| H | 1.177896 | 1.593874 | 2.704937 |
| H | 3.069213 | -1.89899 | 2.490458 |
| H | 0.966736 | -2.14621 | 2.129659 |
| H | 3.289683 | -1.82451 | -2.3931 |
| H | 1.177759 | -1.59398 | -2.70478 |
| H | 0.966419 | 2.146038 | -2.12959 |
| H | 3.068915 | 1.899099 | -2.49045 |

**Table S5.** Geometry optimization and frequency calculation information for **1-M-3**.

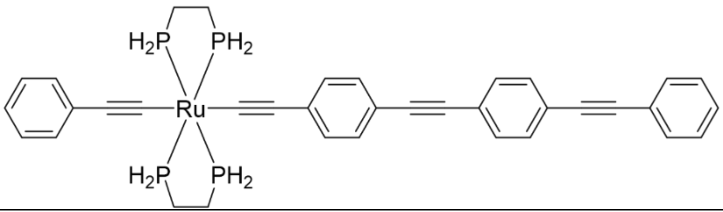

| SCF: E(RPBE1PBE) = -2850.29817880 a.u. |
|---|
| Electronic spatial extent (a.u.): <R**2> = 85104.1293 |
| Dipole moment (field-independent basis, Debye): |
| X = -2.1803 Y = 0.0000 Z = 0.0001 Tot = 2.1803 |
| Dipole = -8.57795049D-01 1.55899463D-05 3.21222933D-05 |
| Cartesian Forces: Max 0.000005425 RMS 0.000001645 |
| Number of Imaginary Freq = 0 |
| Symmetry c1 |

| Atom | X | Y | Z |
|---|---|---|---|
| Ru | -4.62235 | 0.000017 | 0.000038 |
| P | -4.65695 | -1.88032 | -1.32231 |
| P | -4.62382 | -1.51177 | 1.732887 |
| C | -5.03793 | -3.36692 | -0.27628 |
| H | -4.74942 | -4.29142 | -0.78543 |
| H | -6.12639 | -3.37771 | -0.14326 |
| C | -4.33588 | -3.22389 | 1.071723 |
| H | -4.67263 | -3.98228 | 1.784903 |
| H | -3.25091 | -3.32987 | 0.952225 |
| P | -4.62383 | 1.511804 | -1.73281 |
| P | -4.65697 | 1.880345 | 1.322387 |
| C | -5.03797 | 3.36695 | 0.276364 |
| H | -4.74947 | 4.291447 | 0.785516 |
| H | -6.12643 | 3.377726 | 0.143331 |
| C | -4.33592 | 3.223933 | -1.07164 |
| H | -4.67268 | 3.982315 | -1.78482 |
| H | -3.25094 | 3.32993 | -0.95215 |
| C | -2.58195 | 0.000016 | 0.000045 |
| C | -1.34638 | 0.000008 | 0.000032 |
| C | -6.67082 | 0.000007 | 0.000019 |
| C | -7.90472 | 0 | -1.5E-05 |
| C | 0.073327 | 0.000005 | 0.000023 |
| C | 0.804443 | -1.04839 | -0.59886 |
| C | 0.804454 | 1.048399 | 0.598886 |
| C | 2.188434 | -1.05037 | -0.59961 |
| H | 0.262128 | -1.86541 | -1.06643 |
| C | 2.188445 | 1.050375 | 0.599615 |
| H | 0.262149 | 1.865418 | 1.066467 |
| C | 2.909238 | 0.000002 | 0.000001 |
| H | 2.73126 | -1.86748 | -1.06626 |
| H | 2.731278 | 1.867483 | 1.066265 |

| | | | |
|---|---|---|---|
| C | -9.33055 | -1.9E-05 | -0.00006 |
| C | -10.0574 | 0.627201 | -1.03121 |
| C | -10.0575 | -0.62726 | 1.031042 |
| C | -11.4478 | 0.625763 | -1.02869 |
| H | -9.51235 | 1.115301 | -1.83493 |
| C | -11.4478 | -0.62588 | 1.028418 |
| H | -9.51243 | -1.11534 | 1.834797 |
| C | -12.1523 | -7.4E-05 | -0.00016 |
| H | -11.9859 | 1.11663 | -1.83594 |
| H | -11.986 | -1.11676 | 1.83563 |
| H | -13.2389 | -9.3E-05 | -0.0002 |
| C | 4.327292 | -2E-06 | -7E-06 |
| C | 5.545225 | -4E-06 | -1.4E-05 |
| C | 6.96337 | -7E-06 | -1.6E-05 |
| C | 7.681243 | -1.05803 | -0.58969 |
| C | 7.681248 | 1.058007 | 0.589663 |
| C | 9.065431 | -1.05797 | -0.58956 |
| H | 7.137467 | -1.87889 | -1.04714 |
| C | 9.065437 | 1.057944 | 0.589537 |
| H | 7.137478 | 1.878869 | 1.047118 |
| C | 9.782347 | -1.7E-05 | -1.1E-05 |
| H | 9.609348 | -1.87883 | -1.04694 |
| H | 9.609358 | 1.878799 | 1.046922 |
| H | -5.80296 | 1.671263 | -2.49658 |
| H | -3.68701 | 1.447458 | -2.78795 |
| H | -5.57558 | -2.02904 | -2.38458 |
| H | -3.47767 | -2.27444 | -1.99655 |
| H | -5.80296 | -1.67125 | 2.496644 |
| H | -3.68702 | -1.44741 | 2.788042 |
| H | -3.4777 | 2.274479 | 1.996636 |
| H | -5.57561 | 2.029056 | 2.384651 |
| C | 11.20165 | -1.9E-05 | -1.2E-05 |
| C | 12.41839 | -0.00002 | -1.2E-05 |
| C | 13.84009 | -1.5E-05 | -1.2E-05 |
| C | 14.55234 | 1.063619 | 0.582175 |
| C | 14.55235 | -1.06364 | -0.58222 |
| C | 15.94185 | 1.058923 | 0.57954 |
| H | 14.00473 | 1.886256 | 1.032393 |
| C | 15.94186 | -1.05892 | -0.57961 |
| H | 14.00475 | -1.88628 | -1.03243 |
| C | 16.64071 | 0.000009 | -3.8E-05 |
| H | 16.48254 | 1.885611 | 1.031913 |
| H | 16.48255 | -1.88559 | -1.03199 |
| H | 17.7271 | 0.000018 | -4.6E-05 |

**Table S6.** Geometry optimization and frequency calculation information for **1-M-4**.

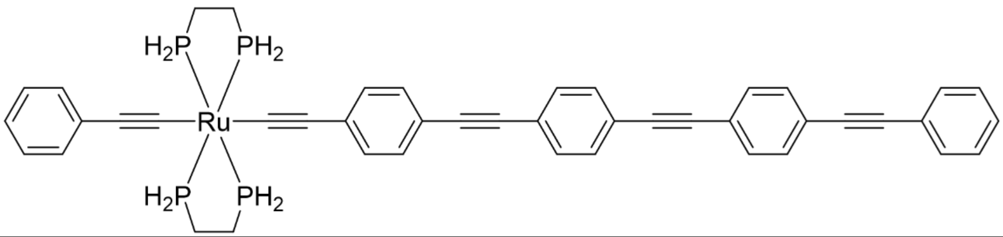


| SCF: E(RPBE1PBE) = -3157.15142138 a.u. |
|---|
| Electronic spatial extent (a.u.): <R**2> = 157116.1005 |
| Dipole moment (field-independent basis, Debye): |
| X = 2.3115 Y = -0.0001 Z = 0.0001 Tot = 2.3115 |
| Dipole = 9.09415353D-01 -2.24445916D-05 5.74936034D-05 |
| Cartesian Forces: Max 0.000001899 RMS 0.000000490 |
| Number of Imaginary Freq = 0 |
| Symmetry c1 |

| Atom | X | Y | Z |
|---|---|---|---|
| Ru | 7.369829 | 0.000014 | 0.000001 |
| P | 7.405553 | 1.856428 | -1.356145 |
| P | 7.370045 | 1.542878 | 1.705093 |
| C | 7.783993 | 3.361832 | -0.336493 |
| H | 7.495526 | 4.276805 | -0.862576 |
| H | 8.872252 | 3.375785 | -0.20216 |
| C | 7.080284 | 3.242287 | 1.012919 |
| H | 7.414831 | 4.014125 | 1.712558 |
| H | 5.995281 | 3.344202 | 0.890065 |
| P | 7.370091 | -1.542852 | -1.705089 |
| P | 7.405397 | -1.856402 | 1.356153 |
| C | 7.783831 | -3.361824 | 0.336526 |
| H | 7.495289 | -4.276783 | 0.862592 |
| H | 8.872098 | -3.375826 | 0.202263 |
| C | 7.080213 | -3.242248 | -1.01293 |
| H | 7.414771 | -4.014102 | -1.712547 |
| H | 5.995198 | -3.344116 | -0.890145 |
| C | 5.329669 | 0.00004 | -0.000061 |
| C | 4.094047 | 0.000057 | -0.000085 |
| C | 9.418358 | -0.000029 | 0.00006 |
| C | 10.652246 | -0.000054 | 0.000096 |
| C | 2.674501 | 0.000067 | -0.000114 |
| C | 1.943285 | 1.146411 | -0.37932 |
| C | 1.943258 | -1.14627 | 0.379066 |
| C | 0.559371 | 1.148493 | -0.379733 |
| H | 2.485441 | 2.040042 | -0.675582 |
| C | 0.559344 | -1.148336 | 0.379428 |
| H | 2.485393 | -2.039906 | 0.675348 |
| C | -0.161436 | 0.000083 | -0.000166 |

| | | | |
|---|---|---|---|
| H | 0.016542 | 2.04187 | -0.675206 |
| H | 0.016493 | -2.041706 | 0.674881 |
| C | 12.078092 | -0.00009 | 0.000139 |
| C | 12.804961 | -0.637474 | -1.024738 |
| C | 12.804931 | 0.63725 | 1.025065 |
| C | 14.195355 | -0.636062 | -1.022143 |
| H | 12.259915 | -1.133458 | -1.823619 |
| C | 14.195325 | 0.635758 | 1.022561 |
| H | 12.259861 | 1.133263 | 1.823912 |
| C | 14.89976 | -0.000172 | 0.000232 |
| H | 14.733523 | -1.134903 | -1.824462 |
| H | 14.733469 | 1.134568 | 1.824916 |
| H | 15.986386 | -0.000203 | 0.000267 |
| C | -1.57931 | 0.000091 | -0.000184 |
| C | -2.797295 | 0.000099 | -0.000195 |
| C | -4.215146 | 0.000085 | -0.000186 |
| C | -4.932908 | 1.153641 | -0.370448 |
| C | -4.932878 | -1.153484 | 0.370097 |
| C | -6.316876 | 1.153686 | -0.37037 |
| H | -4.388976 | 2.048277 | -0.657608 |
| C | -6.316845 | -1.153554 | 0.370055 |
| H | -4.388922 | -2.048109 | 0.657245 |
| C | -7.033446 | 0.00006 | -0.000147 |
| H | -6.860967 | 2.048309 | -0.657455 |
| H | -6.860914 | -2.048186 | 0.657156 |
| H | 8.548984 | -1.717622 | -2.465871 |
| H | 6.433427 | -1.496644 | -2.761306 |
| H | 8.326315 | 1.98588 | -2.419057 |
| H | 6.227686 | 2.23797 | -2.039917 |
| H | 8.548894 | 1.717596 | 2.465956 |
| H | 6.433308 | 1.496705 | 2.761248 |
| H | 6.22747 | -2.237886 | 2.039852 |
| H | 8.326088 | -1.985893 | 2.419122 |
| C | -8.451854 | 0.000047 | -0.000124 |
| C | -9.669067 | 0.000036 | -0.000101 |
| C | -11.087566 | 0.000018 | -0.000067 |
| C | -11.80364 | -1.156207 | 0.36262 |
| C | -11.80369 | 1.156222 | -0.362719 |
| C | -13.187811 | -1.156095 | 0.362559 |
| H | -11.259398 | -2.052538 | 0.64375 |
| C | -13.187861 | 1.156073 | -0.362585 |
| H | -11.259486 | 2.052567 | -0.643878 |
| C | -13.903931 | -0.00002 | 0.000007 |
| H | -13.732049 | -2.052434 | 0.643675 |
| H | -13.732137 | 2.052397 | -0.643672 |
| C | -15.323163 | -0.000039 | 0.000049 |

| | | | |
|---|---|---|---|
| C | -16.539807 | -0.000049 | 0.000087 |
| C | -17.961466 | -0.000066 | 0.000139 |
| C | -18.673323 | -1.158452 | 0.359029 |
| C | -18.673378 | 1.158303 | -0.358696 |
| C | -20.062801 | -1.1533 | 0.35746 |
| H | -18.125583 | -2.054148 | 0.636489 |
| C | -20.062855 | 1.15312 | -0.357015 |
| H | -18.125679 | 2.054011 | -0.6362 |
| C | -20.761469 | -0.000097 | 0.000251 |
| H | -20.603594 | -2.053373 | 0.636306 |
| H | -20.60369 | 2.053182 | -0.635818 |
| H | -21.8479 | -0.00011 | 0.000295 |

**Table S7.** Geometry optimization and frequency calculation information for **2-M-2**.

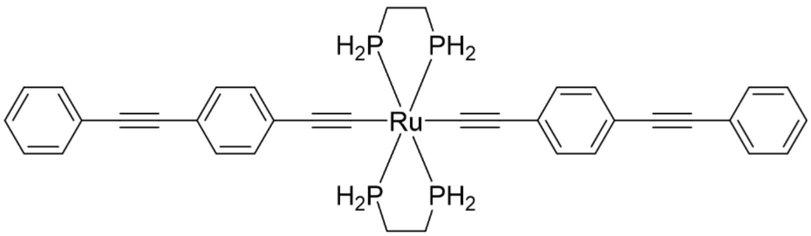

| SCF: E(RPBE1PBE) = -2850.29945708 a.u. |
|---|
| Electronic spatial extent (au): <R**2> = 74049.4957 |
| Dipole moment (field-independent basis, Debye): |
| X = -0.0001 Y = -0.0000 Z = 0.0001 Tot = 0.0001 |
| Dipole = -2.67690256D-05 -2.21759391D-06 -2.21759391D-06 |
| Cartesian Forces: Max 0.000001688 RMS 0.000000405 |
| Number of Imaginary Freq = 0 |
| Symmetry c1 |

| Atom | X | Y | Z |
|---|---|---|---|
| Ru | 0.000002 | 0.000028 | 0.000012 |
| P | -0.01684 | -1.71007 | -1.53876 |
| P | 0.017039 | -1.71008 | 1.538778 |
| C | -0.3518 | -3.32082 | -0.67722 |
| H | -0.04029 | -4.16898 | -1.29422 |
| H | -1.43885 | -3.37896 | -0.54492 |
| C | 0.352273 | -3.32076 | 0.677227 |
| H | 0.04092 | -4.16898 | 1.294225 |
| H | 1.439341 | -3.3787 | 0.544936 |
| P | 0.016871 | 1.71014 | -1.53875 |
| P | -0.01708 | 1.710123 | 1.53879 |
| C | -0.35231 | 3.32081 | 0.677243 |
| H | -0.04099 | 4.169025 | 1.294253 |
| H | -1.43938 | 3.378734 | 0.544925 |
| C | 0.351786 | 3.320886 | -0.67718 |
| H | 0.040285 | 4.169043 | -1.29419 |
| H | 1.43884 | 3.379035 | -0.54487 |
| C | 2.042629 | 0.000102 | 0.00003 |
| C | 3.277712 | 0.000114 | 0.000031 |
| C | -2.04262 | -4.6E-05 | -9E-06 |
| C | -3.27771 | -6.2E-05 | -0.00002 |
| C | 4.698256 | 0.000097 | 0.000024 |
| C | 5.428975 | -1.02252 | -0.64115 |
| C | 5.429012 | 1.022693 | 0.641184 |
| C | 6.813382 | -1.02429 | -0.64201 |
| H | 4.886832 | -1.81987 | -1.1418 |
| C | 6.813419 | 1.024428 | 0.642022 |
| H | 4.886898 | 1.82006 | 1.141845 |
| C | 7.534322 | 0.00006 | 0.000002 |
| H | 7.35599 | -1.82165 | -1.14193 |

| | | | |
|---|---|---|---|
| H | 7.356055 | 1.821771 | 1.141939 |
| C | -4.69825 | -0.00007 | -2.4E-05 |
| C | -5.42899 | 1.022513 | -0.64123 |
| C | -5.42899 | -1.02265 | 0.641184 |
| C | -6.8134 | 1.024264 | -0.64207 |
| H | -4.88687 | 1.819857 | -1.14191 |
| C | -6.81339 | -1.0244 | 0.642038 |
| H | -4.88686 | -1.81999 | 1.141868 |
| C | -7.53432 | -0.00007 | -1.5E-05 |
| H | -7.35602 | 1.821599 | -1.14201 |
| H | -7.35601 | -1.82174 | 1.141989 |
| C | 8.953706 | 0.000037 | -8E-06 |
| C | 10.17101 | 0.000011 | -1.9E-05 |
| C | 11.59273 | -2.2E-05 | -2.8E-05 |
| C | 12.30666 | -1.02994 | -0.63876 |
| C | 12.30672 | 1.029854 | 0.638704 |
| C | 13.69628 | -1.02552 | -0.63596 |
| H | 11.75954 | -1.82723 | -1.1332 |
| C | 13.69634 | 1.025346 | 0.635906 |
| H | 11.75965 | 1.827165 | 1.133153 |
| C | 14.39607 | -0.00011 | -2.8E-05 |
| H | 14.23647 | -1.82676 | -1.13279 |
| H | 14.23658 | 1.826557 | 1.132746 |
| H | 15.48248 | -0.00014 | -2.5E-05 |
| H | -1.1631 | 1.990323 | -2.26575 |
| H | 0.944458 | 1.750725 | -2.60274 |
| H | -0.94439 | -1.75064 | -2.60278 |
| H | 1.163157 | -1.99026 | -2.26573 |
| H | -1.16294 | -1.99046 | 2.26571 |
| H | 0.944567 | -1.75052 | 2.602822 |
| H | 1.16288 | 1.990506 | 2.265749 |
| H | -0.94463 | 1.750541 | 2.602815 |
| C | -8.9537 | -0.00007 | -1.1E-05 |
| C | -10.171 | -6.3E-05 | -8E-06 |
| C | -11.5927 | -5.4E-05 | -0.00001 |
| C | -12.3067 | -1.02994 | 0.638733 |
| C | -12.3067 | 1.029838 | -0.63876 |
| C | -13.6963 | -1.02546 | 0.635937 |
| H | -11.7596 | -1.82723 | 1.133192 |
| C | -13.6963 | 1.025382 | -0.63597 |
| H | -11.7596 | 1.827121 | -1.13322 |
| C | -14.3961 | -3.2E-05 | -1.4E-05 |
| H | -14.2365 | -1.82667 | 1.132788 |
| H | -14.2365 | 1.826605 | -1.13282 |
| H | -15.4825 | -2.4E-05 | -1.4E-05 |

**Table S8.** Geometry optimization and frequency calculation information for **2-M-3**.

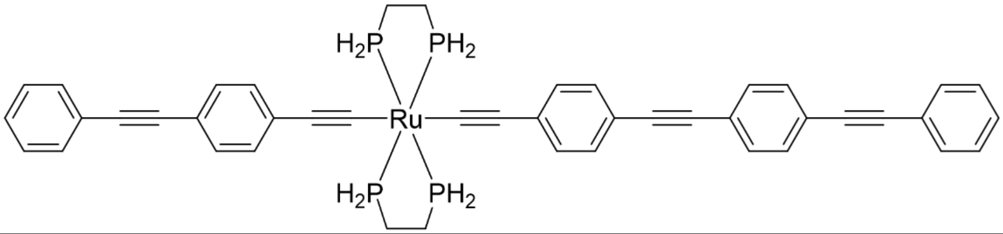


| |
|---|
| SCF: E(RPBE1PBE) = -3157.15295781 a.u.<br>Electronic spatial extent (a.u.): <R**2> = 133987.0590<br>Dipole moment (field-independent basis, Debye):<br>X = -0.4484 Y = 0.0000 Z = 0.0000 Tot = 0.4484<br>Dipole = -1.76412369D-01 1.95953388D-05 1.36528119D-05<br>Cartesian Forces: Max 0.000001391 RMS 0.000000328<br>Number of Imaginary Freq = 0 |
| Symmetry c1 |

| Atom | X | Y | Z |
|---|---|---|---|
| Ru | -2.45831 | 0.000157 | 0.000005 |
| P | -2.47533 | 1.803624 | 1.428032 |
| P | -2.44572 | 1.60938 | -1.644 |
| C | -2.8173 | 3.355552 | 0.466893 |
| H | -2.50804 | 4.24221 | 1.028391 |
| H | -3.90477 | 3.401227 | 0.33296 |
| C | -2.11534 | 3.272388 | -0.88618 |
| H | -2.43076 | 4.078998 | -1.55476 |
| H | -1.02829 | 3.342687 | -0.75975 |
| P | -2.44571 | -1.60907 | 1.644015 |
| P | -2.47532 | -1.80331 | -1.42802 |
| C | -2.81728 | -3.35524 | -0.46688 |
| H | -2.50803 | -4.2419 | -1.02838 |
| H | -3.90475 | -3.40091 | -0.33295 |
| C | -2.11532 | -3.27207 | 0.886192 |
| H | -2.43074 | -4.07868 | 1.554769 |
| H | -1.02828 | -3.34237 | 0.759763 |
| C | -0.41678 | 0.000159 | 0.000003 |
| C | 0.818465 | 0.000151 | 0.000002 |
| C | -4.50114 | 0.000132 | 0.000006 |
| C | -5.73619 | 0.000098 | 0.000007 |
| C | 2.238368 | 0.000135 | -3E-06 |
| C | 2.969044 | 1.026022 | 0.636737 |
| C | 2.969013 | -1.02577 | -0.63675 |
| C | 4.353077 | 1.027879 | 0.637648 |
| H | 2.426636 | 1.825436 | 1.133691 |
| C | 4.353045 | -1.02767 | -0.63767 |
| H | 2.426579 | -1.82517 | -1.1337 |
| C | 5.073693 | 0.000097 | -1.2E-05 |
| H | 4.895956 | 1.827322 | 1.133859 |

| | | | |
|---|---|---|---|
| H | 4.895899 | -1.82712 | -1.13388 |
| C | -7.15675 | 0.000043 | 0.000007 |
| C | -7.88741 | -1.02152 | 0.642877 |
| C | -7.88749 | 1.021544 | -0.64286 |
| C | -9.27182 | -1.02334 | 0.643717 |
| H | -7.34528 | -1.81806 | 1.144836 |
| C | -9.2719 | 1.023243 | -0.64371 |
| H | -7.34544 | 1.818135 | -1.14482 |
| C | -9.99277 | -0.00008 | 0.000005 |
| H | -9.8144 | -1.81989 | 1.14494 |
| H | -9.81456 | 1.81975 | -1.14493 |
| C | 6.491804 | 0.000074 | -1.5E-05 |
| C | 7.709696 | 0.000047 | -1.9E-05 |
| C | 9.127876 | 0.00001 | -2.4E-05 |
| C | 9.845698 | 1.03231 | 0.63359 |
| C | 9.845638 | -1.03233 | -0.63364 |
| C | 11.22989 | 1.032194 | 0.6335 |
| H | 9.301934 | 1.833219 | 1.12515 |
| C | 11.22983 | -1.03229 | -0.63355 |
| H | 9.301828 | -1.83321 | -1.1252 |
| C | 11.94674 | -7.1E-05 | -2.5E-05 |
| H | 11.77384 | 1.833054 | 1.125014 |
| H | 11.77374 | -1.83319 | -1.12507 |
| H | -3.6272 | -1.83821 | 2.386015 |
| H | -1.51912 | -1.58484 | 2.709431 |
| H | -3.40052 | 1.908532 | 2.489719 |
| H | -1.29424 | 2.132311 | 2.132771 |
| H | -3.62722 | 1.838518 | -2.386 |
| H | -1.51914 | 1.585154 | -2.70942 |
| H | -1.29422 | -2.13199 | -2.13275 |
| H | -3.4005 | -1.90822 | -2.48971 |
| C | -11.4122 | -0.00014 | 0.000004 |
| C | -12.6295 | -0.00021 | 0.000005 |
| C | -14.0512 | -0.00028 | -1E-06 |
| C | -14.7652 | 1.029209 | -0.63933 |
| C | -14.7651 | -1.02985 | 0.639325 |
| C | -16.1548 | 1.024668 | -0.63653 |
| H | -14.2181 | 1.826252 | -1.13423 |
| C | -16.1547 | -1.02547 | 0.636519 |
| H | -14.2179 | -1.82684 | 1.134227 |
| C | -16.8545 | -0.00044 | -0.00001 |
| H | -16.6951 | 1.82558 | -1.13383 |
| H | -16.6949 | -1.82644 | 1.133811 |
| H | -17.9409 | -0.0005 | -1.4E-05 |
| C | 13.36604 | -0.00012 | -2.3E-05 |
| C | 14.58278 | -0.00016 | -1.8E-05 |

| | | | |
|---|---|---|---|
| C | 16.00447 | -0.00021 | -2E-06 |
| C | 16.71674 | 1.034951 | 0.631405 |
| C | 16.71668 | -1.03542 | -0.63139 |
| C | 18.10625 | 1.030306 | 0.628604 |
| H | 16.16916 | 1.83559 | 1.119702 |
| C | 18.10619 | -1.03087 | -0.62855 |
| H | 16.16906 | -1.83602 | -1.1197 |
| C | 18.80506 | -0.00031 | 0.000038 |
| H | 18.64696 | 1.834837 | 1.119273 |
| H | 18.64686 | -1.83544 | -1.1192 |
| H | 19.89145 | -0.00035 | 0.000055 |

**Table S9.** Geometry optimization and frequency calculation information for **3-M-3**.

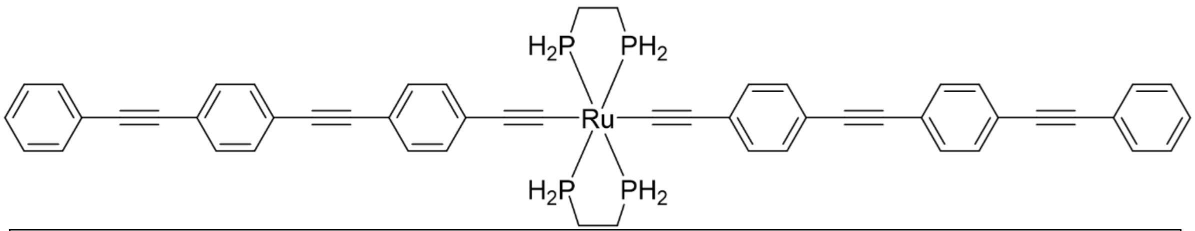

| SCF: E(RPBE1PBE) = -3464.00641359 a.u. |
|---|
| Electronic spatial extent (a.u.): <R**2> = 212665.6439 |
| Dipole moment (field-independent basis, Debye): |
| X = -0.0002 Y = 0.0002 Z = 0.0052 Tot = 0.0052 |
| Dipole = -7.07068252D-05 6.34322965D-05 2.03305896D-03 |
| Cartesian Forces: Max 0.000001732 RMS 0.000000304 |
| Symmetry c1 |

| Atom | X | Y | Z |
|---|---|---|---|
| Ru | 0.000004 | 0.000752 | 0.002768 |
| P | -0.00637 | -1.7099 | -1.53608 |
| P | 0.0257 | -1.70941 | 1.541782 |
| C | -0.32878 | -3.32314 | -0.67434 |
| H | -0.0098 | -4.16871 | -1.29106 |
| H | -1.4154 | -3.39048 | -0.5429 |
| C | 0.37433 | -3.31727 | 0.680594 |
| H | 0.069364 | -4.16792 | 1.297429 |
| H | 1.461921 | -3.36638 | 0.549062 |
| P | 0.006249 | 1.711517 | -1.53595 |
| P | -0.02558 | 1.710805 | 1.541906 |
| C | -0.37433 | 3.318721 | 0.680869 |
| H | -0.06933 | 4.169328 | 1.297742 |
| H | -1.46193 | 3.367807 | 0.549432 |
| C | 0.328668 | 3.324706 | -0.67413 |
| H | 0.009606 | 4.170309 | -1.29076 |
| H | 1.415303 | 3.392081 | -0.54277 |
| C | 2.041877 | 0.008686 | 0.002479 |
| C | 3.27707 | 0.011203 | 0.002098 |
| C | -2.04187 | -0.00725 | 0.002605 |
| C | -3.27706 | -0.00989 | 0.002277 |
| C | 4.697011 | 0.011918 | 0.001271 |
| C | 5.427356 | -1.01287 | -0.63757 |
| C | 5.427853 | 1.036802 | 0.639314 |
| C | 6.811371 | -1.01479 | -0.63902 |
| H | 4.88474 | -1.81149 | -1.13558 |
| C | 6.811916 | 1.038655 | 0.639673 |
| H | 4.885702 | 1.835432 | 1.137801 |
| C | 7.532255 | 0.01188 | 0.000131 |
| H | 7.354037 | -1.81345 | -1.13672 |
| H | 7.354981 | 1.837278 | 1.13699 |

| | | | |
|---|---|---|---|
| C | -4.697 | -0.01082 | 0.001498 |
| C | -5.42754 | 1.013751 | -0.63747 |
| C | -5.42766 | -1.03574 | 0.639708 |
| C | -6.81155 | 1.015424 | -0.6389 |
| H | -4.88507 | 1.812392 | -1.1356 |
| C | -6.81172 | -1.03783 | 0.640086 |
| H | -4.88536 | -1.8342 | 1.138302 |
| C | -7.53225 | -0.01128 | 0.000398 |
| H | -7.35436 | 1.813914 | -1.13671 |
| H | -7.35464 | -1.83648 | 1.137525 |
| C | 8.950377 | 0.01135 | -0.00021 |
| C | 10.16826 | 0.010151 | -0.00063 |
| C | 11.58645 | 0.008077 | -0.00121 |
| C | 12.30226 | -1.0252 | -0.63549 |
| C | 12.30618 | 1.038962 | 0.632514 |
| C | 13.68644 | -1.02749 | -0.63588 |
| H | 11.75694 | -1.82499 | -1.12715 |
| C | 13.69038 | 1.036532 | 0.631942 |
| H | 11.76393 | 1.840591 | 1.124571 |
| C | 14.40528 | 0.003285 | -0.00217 |
| H | 14.22884 | -1.82911 | -1.12786 |
| H | 14.23584 | 1.836296 | 1.123569 |
| H | -1.17556 | 1.982488 | -2.2633 |
| H | 0.934006 | 1.758777 | -2.59946 |
| H | -0.93421 | -1.75706 | -2.59953 |
| H | 1.17538 | -1.98085 | -2.26352 |
| H | -1.15392 | -1.99791 | 2.266035 |
| H | 0.951248 | -1.74292 | 2.607729 |
| H | 1.154094 | 1.999274 | 2.26607 |
| H | -0.95103 | 1.744223 | 2.607945 |
| C | -8.95037 | -0.01103 | 0.000048 |
| C | -10.1683 | -0.01011 | -0.00039 |
| C | -11.5864 | -0.0084 | -0.001 |
| C | -12.3059 | -1.03934 | 0.632927 |
| C | -12.3025 | 1.024558 | -0.63553 |
| C | -13.6901 | -1.03728 | 0.632307 |
| H | -11.7635 | -1.84071 | 1.125173 |
| C | -13.6867 | 1.026469 | -0.63595 |
| H | -11.7574 | 1.824389 | -1.12733 |
| C | -14.4053 | -0.00437 | -0.00206 |
| H | -14.2354 | -1.83709 | 1.124087 |
| H | -14.2293 | 1.827838 | -1.12813 |
| C | 15.82458 | 0.000674 | -0.00257 |
| C | 17.04131 | -0.00189 | -0.00298 |
| C | 18.463 | -0.00505 | -0.00348 |
| C | 19.17266 | -1.04198 | -0.63491 |

| | | | |
|---|---|---|---|
| C | 19.17781 | 1.028651 | 0.627439 |
| C | 20.56217 | -1.04055 | -0.63259 |
| H | 18.62306 | -1.84146 | -1.12284 |
| C | 20.5673 | 1.020892 | 0.624121 |
| H | 18.6322 | 1.830618 | 1.11577 |
| C | 21.26358 | -0.01142 | -0.00448 |
| H | 21.10085 | -1.84644 | -1.12326 |
| H | 21.11 | 1.824314 | 1.114415 |
| H | 22.34996 | -0.01389 | -0.00486 |
| C | -15.8246 | -0.00216 | -0.00253 |
| C | -17.0413 | 0.000043 | -0.00299 |
| C | -18.463 | 0.002753 | -0.00358 |
| C | -19.1775 | -1.03102 | 0.62754 |
| C | -19.1729 | 1.039315 | -0.6353 |
| C | -20.567 | -1.02371 | 0.624134 |
| H | -18.6317 | -1.8327 | 1.116093 |
| C | -20.5625 | 1.037444 | -0.63306 |
| H | -18.6236 | 1.838853 | -1.12339 |
| C | -21.2636 | 0.008236 | -0.00475 |
| H | -21.1095 | -1.82719 | 1.114583 |
| H | -21.1014 | 1.843043 | -1.12396 |
| H | -22.35 | 0.010362 | -0.0052 |

## Computational studies - 1PA calculations.

The electronic transitions were calculated for all optimized structures. The performance of the hybrid functional PBE0 and the Coulomb-attenuated hybrid functional CAM-B3LYP theory methods were assessed in the calculation of low-lying charge transfer (CT) excitation energies of the metal alkynyl complexes. The 6-311G(d,p) basis set was used for the ligands whereas the SDD basis set and associated pseudopotential of Stuttgart/Dresden were used for the transition metal. $CH_2Cl_2$ solvent corrections were taken into account by means of the PCM solvation model. The 50 lowest energy excitation states were chosen for electronic structure calculations. The 3D isosurface plots of the model compounds were constructed using the Visual Molecular Dynamics (VMD) program.[48] Multiwfn[49] was used to conduct cube file manipulations. Natural transition orbitals (NTOs) analysis was performed with the assistance of Multiwfn.

**Table S10.** Low-energy linear absorption bands for the model complexes and their corresponding experimental complexes.[a]

| Complex | PBE0 | | CAM-B3LYP | | Experiment[b] | |
|---|---|---|---|---|---|---|
| | $\lambda$ (nm) | $f$ | $\lambda$ (nm) | $f$ | $\lambda_{max}$ (nm) | $\varepsilon$ ($10^4$ $M^{-1}cm^{-1}$) |
| **1-M-1** | 316 | 0.946 | 285 | 1.704 | 328 | 3.4 |
| | 302 | 0.797 | | | | |
| **1-M-2** | 402 | 1.673 | 349 | 2.215 | 385 | 3.6 |
| | 310 | 0.344 | | | | |
| | 301 | 0.477 | | | | |
| **1-M-3** | 454 | 2.276 | 380 | 3.113 | 415 | 4.1 |
| | 355 | 0.717 | 280 | 0.773 | | |
| | 301 | 0.608 | | | | |
| **1-M-4** | 484 | 2.491 | 393 | 4.037 | n/a | n/a |
| | 393 | 1.227 | | | | |
| | 302 | 0.647 | | | | |
| **2-M-2** | 409 | 3.044 | 353 | 4.042 | n/a | n/a |
| | 374 | 0.504 | | | | |
| **2-M-3** | 455 | 2.565 | 380 | 3.687 | n/a | n/a |
| | 398 | 1.051 | 348 | 1.281 | | |
| | 356 | 0.611 | | | | |
| **3-M-3** | 461 | 4.123 | 383 | 5.949 | n/a | n/a |

| | | | | |
|---|---|---|---|---|
| | 413 | 0.527 | | |
| | 355 | 1.258 | | |

[a] The first 50 singlet states were calculated. Only transitions with oscillator strengths (*f*) greater than 0.3 are reported. [b] $Ph_2PCH_2CH_2PPh_2$ (dppe) ligands.

**Table S11.** TD-DFT studies of the model complexes (PEB0 functional and 6-311G(d,p)/SDD basis sets). The first 50 singlet states were calculated. Only transitions with oscillator strengths (*f*) greater than 0.3 are reported. Wavenumbers (*v*) are in $cm^{-1}$.

| Complex | State | *v* | *f* | Major contribution (%) |
|---|---|---|---|---|
| **1-M-1** | $S_2$ | 31610 | 0.946 | HOMO → LUMO (92) |
| | $S_5$ | 33152 | 0.797 | HOMO-1 → LUMO+1 (87) |
| | | | | HOMO-3 → LUMO (7) |
| **1-M-2** | $S_3$ | 24889 | 1.673 | HOMO → LUMO (93) |
| | $S_{15}$ | 32282 | 0.344 | HOMO → LUMO+1 (80) |
| | $S_{20}$ | 33276 | 0.477 | HOMO-1 → LUMO+1 (82) |
| **1-M-3** | $S_1$ | 22032 | 2.276 | HOMO → LUMO (90) |
| | $S_3$ | 28183 | 0.717 | HOMO-2 → LUMO (89) |
| | | | | HOMO-4 → LUMO (6) |
| | $S_{11}$ | 33213 | 0.608 | HOMO-1 → LUMO+2 (88) |
| **1-M-4** | $S_1$ | 20656 | 2.491 | HOMO → LUMO (85) |
| | | | | HOMO-2 → LUMO (5) |
| | $S_3$ | 25429 | 1.227 | HOMO-2 → LUMO (85) |
| | $S_{12}$ | 33152 | 0.647 | HOMO-1 → LUMO+3 (77) |
| | | | | HOMO-5 → LUMO (11) |
| **2-M-2** | $S_1$ | 24476 | 3.044 | HOMO → LUMO (87) |
| | | | | HOMO-1 → LUMO+1 (9) |
| | $S_4$ | 26734 | 0.504 | HOMO-1 → LUMO+1 (81) |
| | | | | HOMO → LUMO (7) |
| | $S_9$ | 33551 | 0.366 | HOMO-3 → LUMO (83) |
| **2-M-3** | $S_1$ | 21995 | 2.565 | HOMO → LUMO (79) |
| | | | | HOMO-1 → LUMO (13) |

| | | | | |
|---|---|---|---|---|
| | $S_3$ | 25130 | 1.051 | HOMO → LUMO+1 (84) |
| | | | | HOMO-1 → LUMO+1 (10) |
| | $S_5$ | 28079 | 0.611 | HOMO-2 → LUMO (84) |
| **3-M-3** | $S_1$ | 21704 | 4.123 | HOMO → LUMO (79) |
| | | | | HOMO-1 → LUMO+1 (14) |
| | $S_4$ | 24210 | 0.527 | HOMO-1 → LUMO+1 (77) |
| | | | | HOMO → LUMO (14) |
| | $S_6$ | 28148 | 1.258 | HOMO-2 → LUMO+1 (68) |
| | | | | HOMO-3 → LUMO (20) |
| | $S_9$ | 30559 | 0.386 | HOMO → LUMO+2 (72) |
| | | | | HOMO-1 → LUMO+3 (11) |
| | | | | HOMO-3 → LUMO (7) |

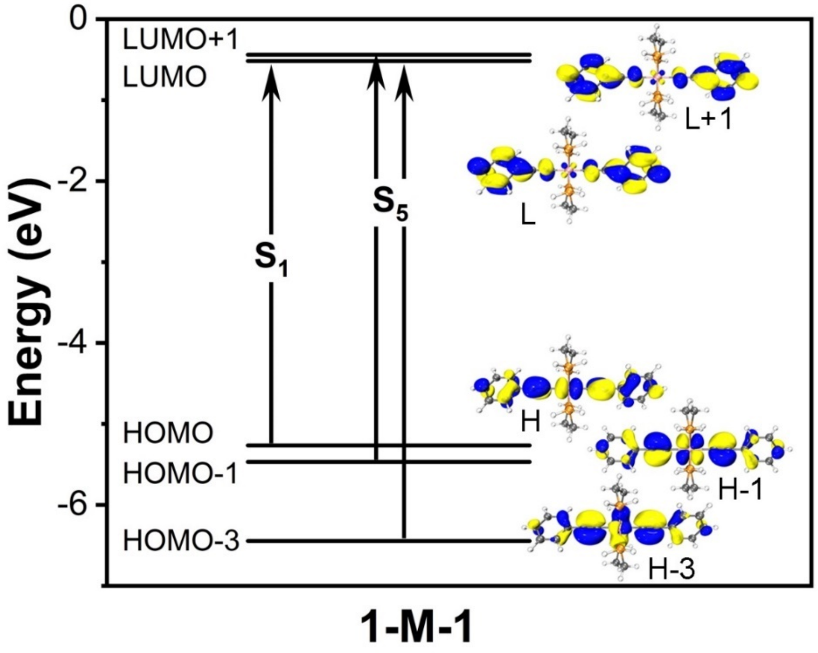


**Fig. S56** Molecular orbital diagram of the model complex **1-M-1** computed at the PBE0/6-311(d,p)/SDD level of theory and plotted with isovalue 0.03 au.

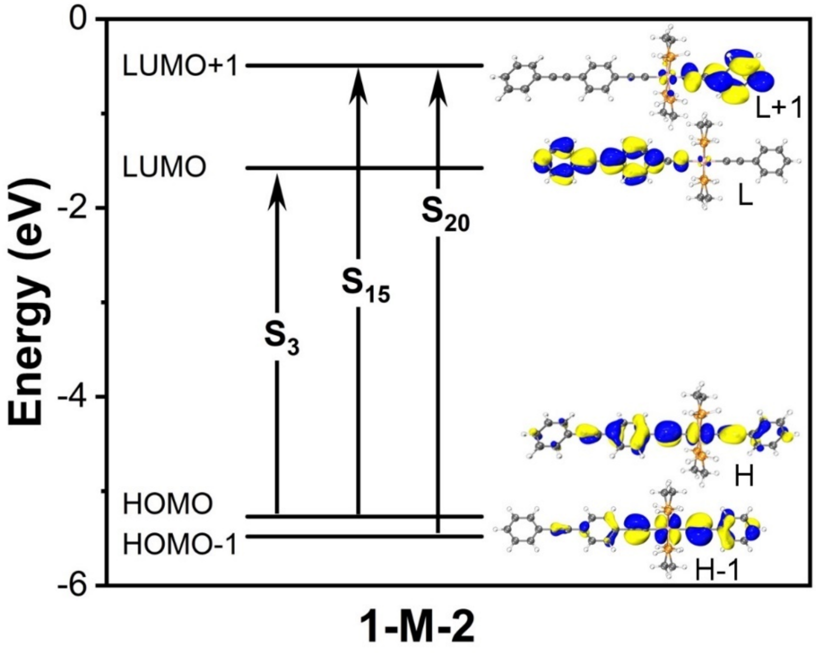


**Fig. S57** Molecular orbital diagram of the model complex **1-M-2** computed at the PBE0/6-311(d,p)/SDD level of theory and plotted with isovalue 0.03 au.

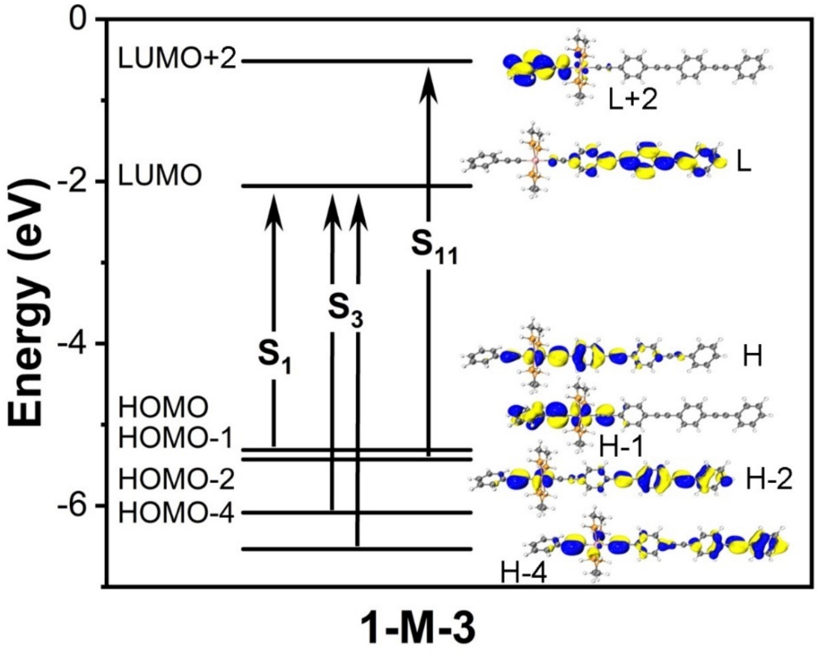


**Fig. S58** Molecular orbital diagram of the model complex **1-M-3** computed at the PBE0/6-311(d,p)/SDD level of theory and plotted with isovalue 0.03 au.

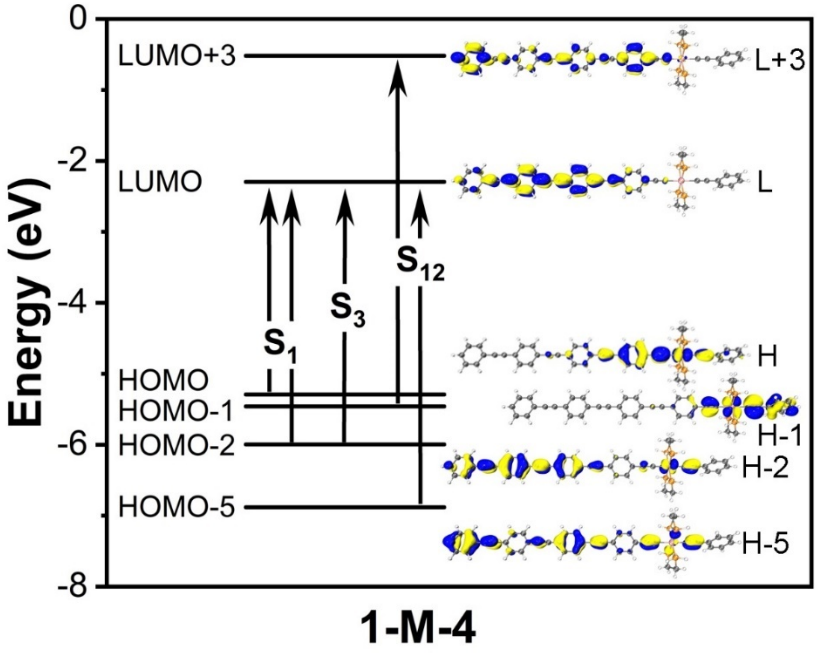


**Fig. S59** Molecular orbital diagram of the model complex **1-M-4** computed at the PBE0/6-311(d,p)/SDD level of theory and plotted with isovalue 0.03 au.

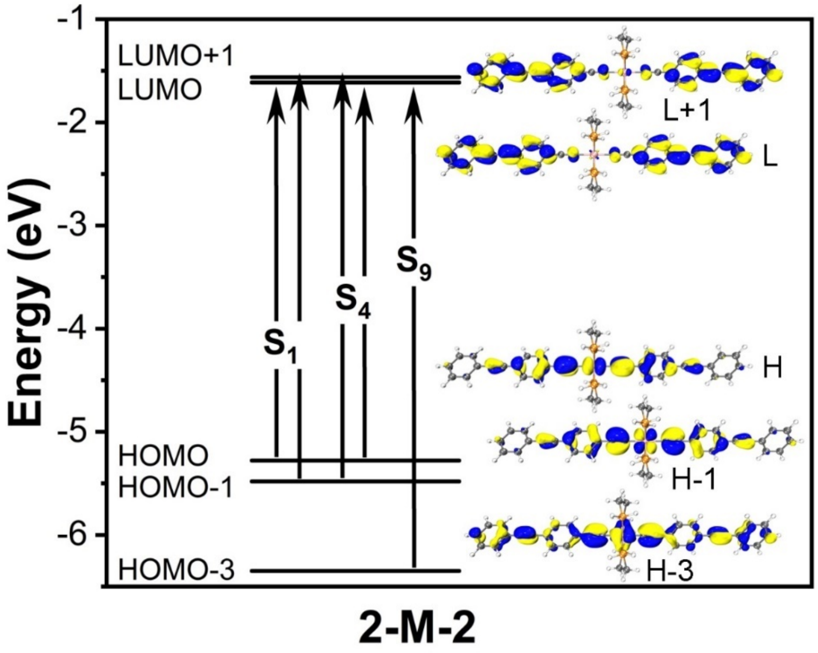


**Fig. S60** Molecular orbital diagram of the model complex **2-M-2** computed at the PBE0/6-311(d,p)/SDD level of theory and plotted with isovalue 0.03 au.

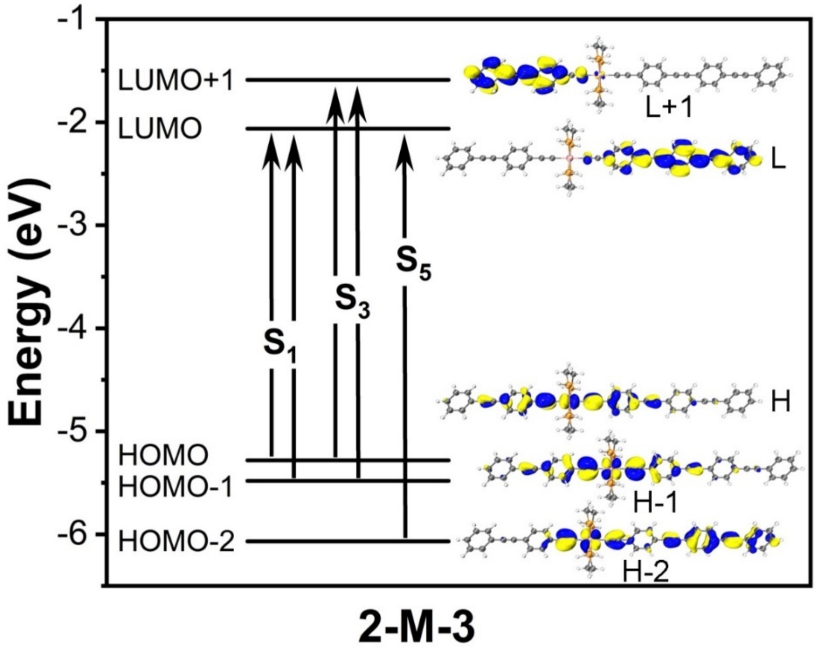


**Fig. S61** Molecular orbital diagram of the model complex **2-M-3** computed at the PBE0/6-311(d,p)/SDD level of theory and plotted with isovalue 0.03 au.

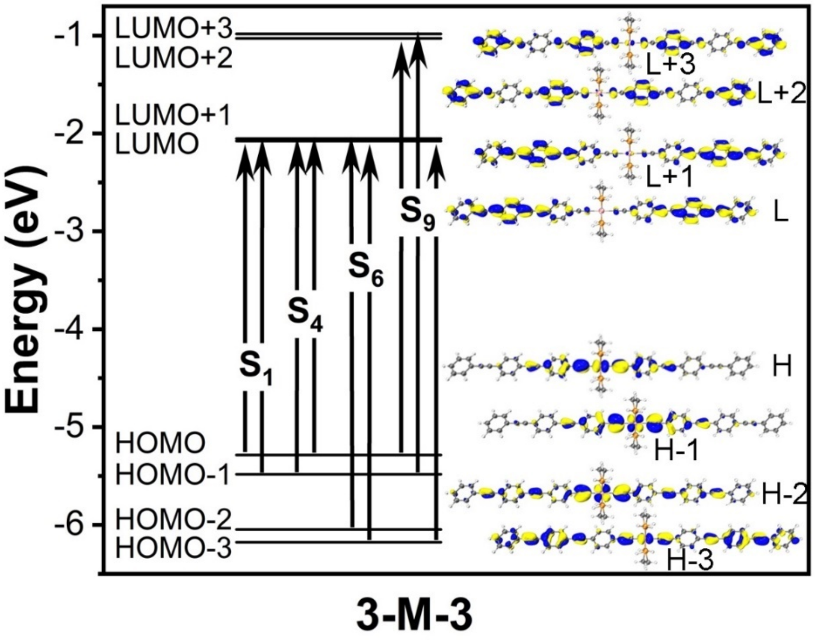


**Fig. S62** Molecular orbital diagram of the model complex **3-M-3** computed at the PBE0/6-311(d,p)/SDD level of theory and plotted with isovalue 0.03 au.

| | | Electron | Hole |
| --- | --- | --- | --- |
| **1-M-1** | NTOs $S_2 \leftarrow S_0$ | 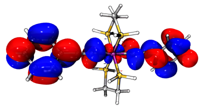 | 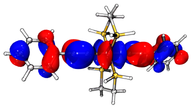 |
| | NTOs $S_5 \leftarrow S_0$ | 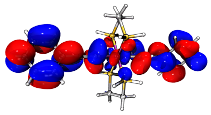 | 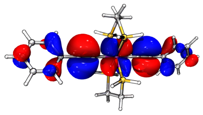 |
| **1-M-2** | NTOs $S_1 \leftarrow S_0$ | 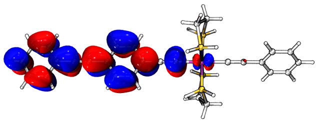 | 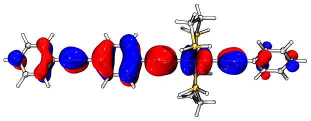 |
| | NTOs $S_2 \leftarrow S_0$ | 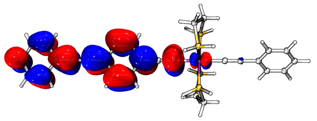 | 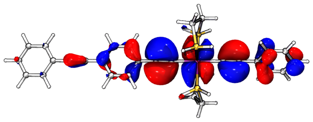 |
| **1-M-3** | NTOs $S_1 \leftarrow S_0$ | 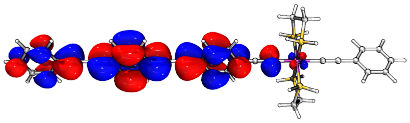 | 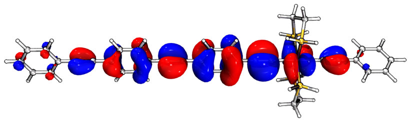 |
| | NTOs $S_3 \leftarrow S_0$ | 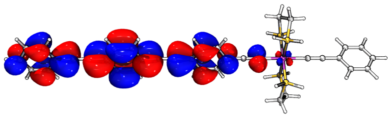 | 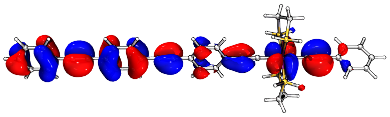 |
| **1-M-4** | NTOs $S_1 \leftarrow S_0$ | 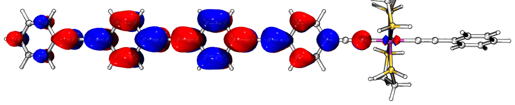 | 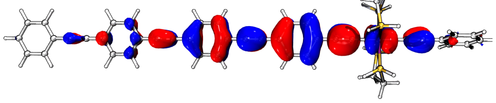 |
| | NTOs $S_3 \leftarrow S_0$ | 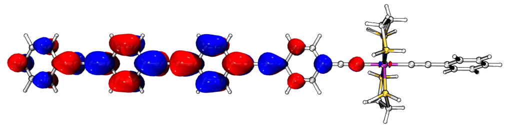 | 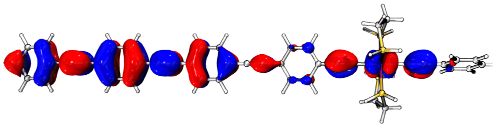 |

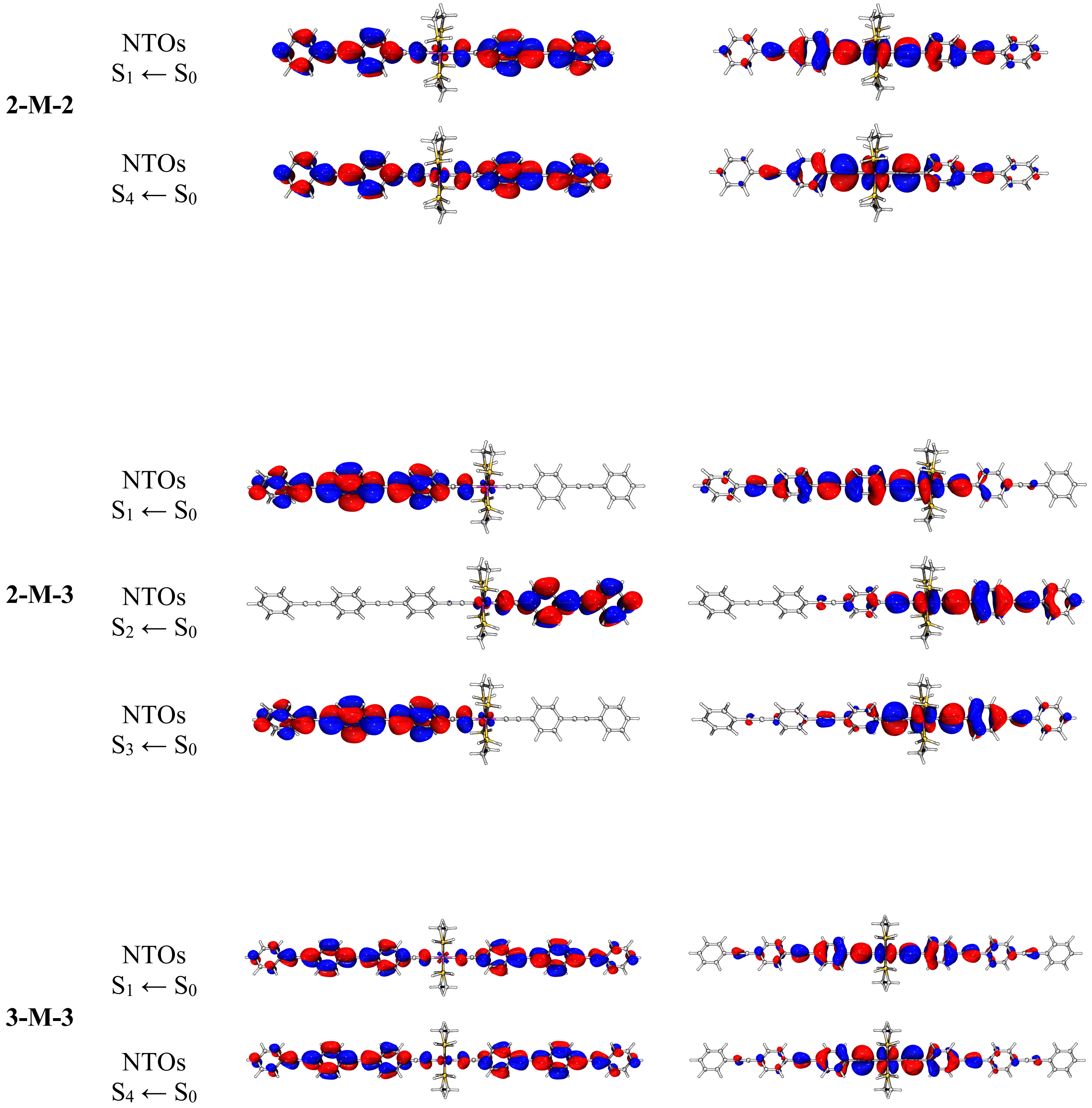


**Fig. S63** TD-DFT-derived natural transition orbitals (isovalue = 0.02 e/bohr$^3$) of the model complexes.

## Z-scan studies

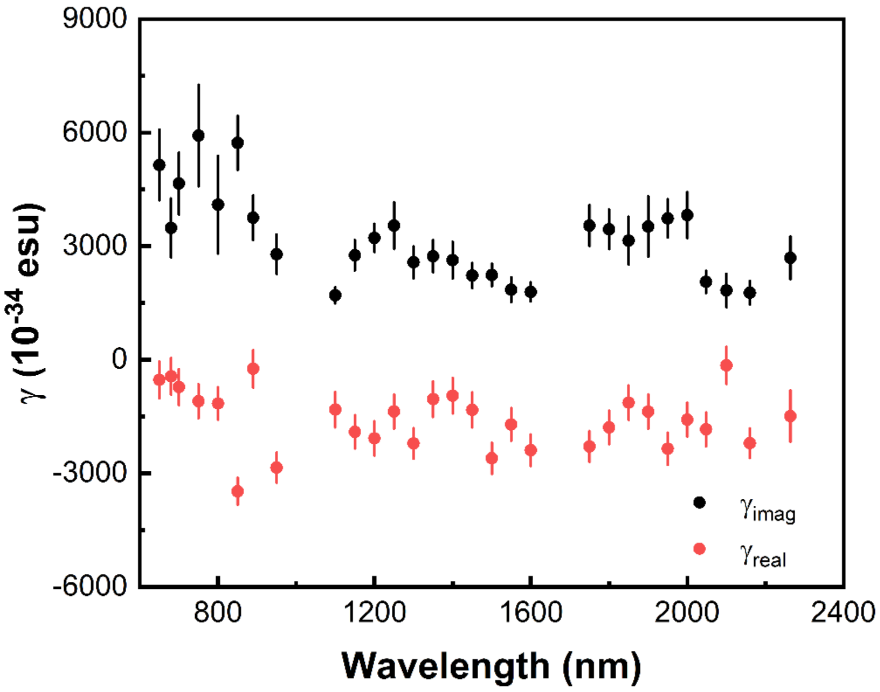


**Fig. S64** Wavelength dependence of the cubic NLO coefficients of **3G$_{22,03,02,01}$-NO$_2$**.

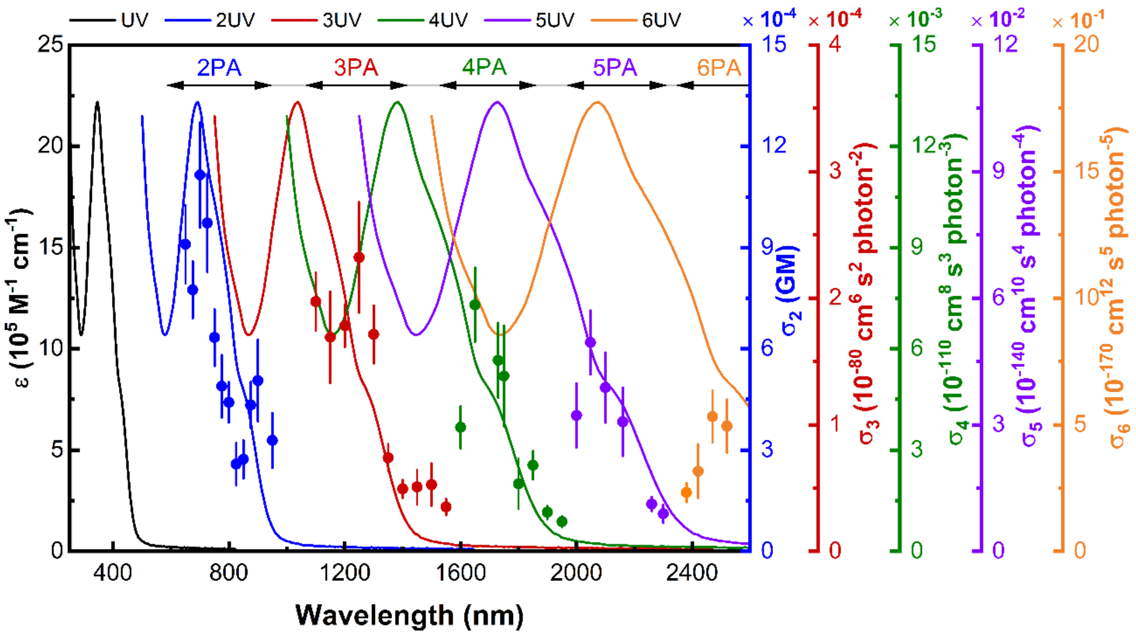


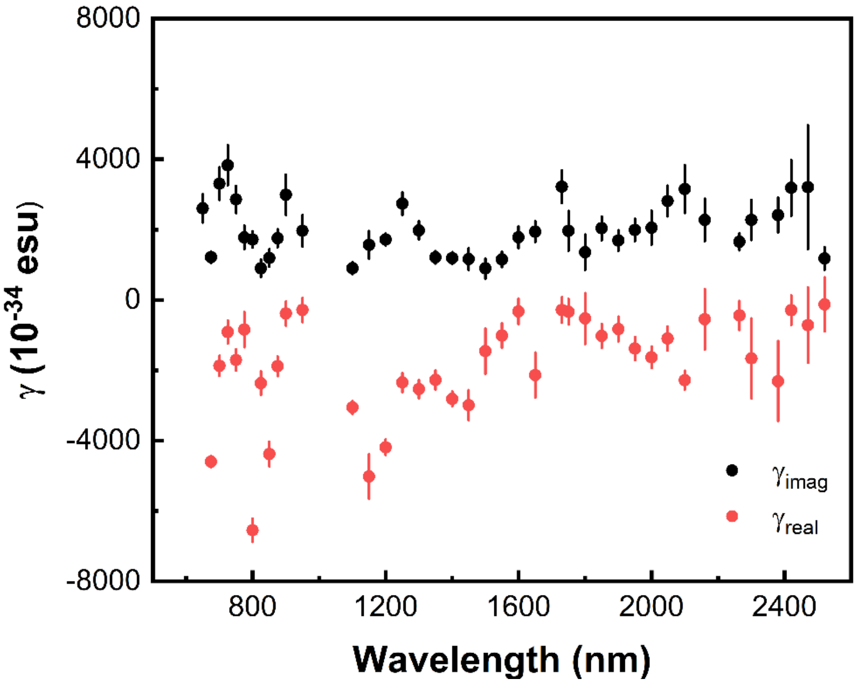


**Figure S65.** Wavelength dependence of the nonlinear absorption (top) and cubic NLO coefficients (bottom) of **3G$_{22,03,02,01}$-s**.

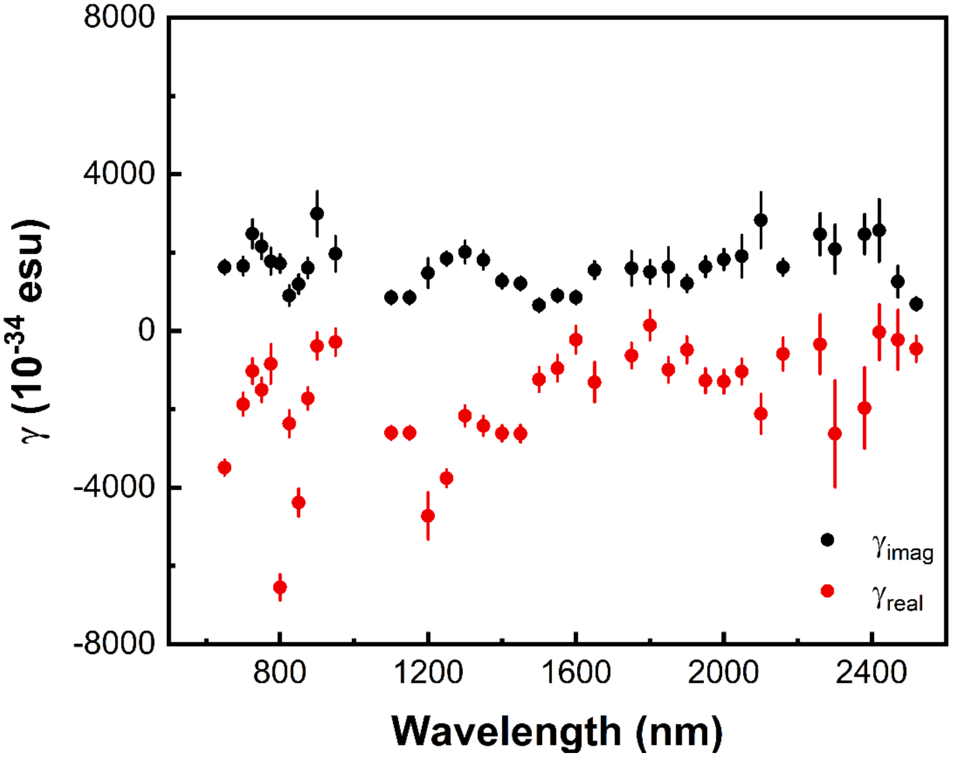


**Figure S66.** Wavelength dependence of the cubic NLO coefficients of **3G$_{22,03,02,01}$**.

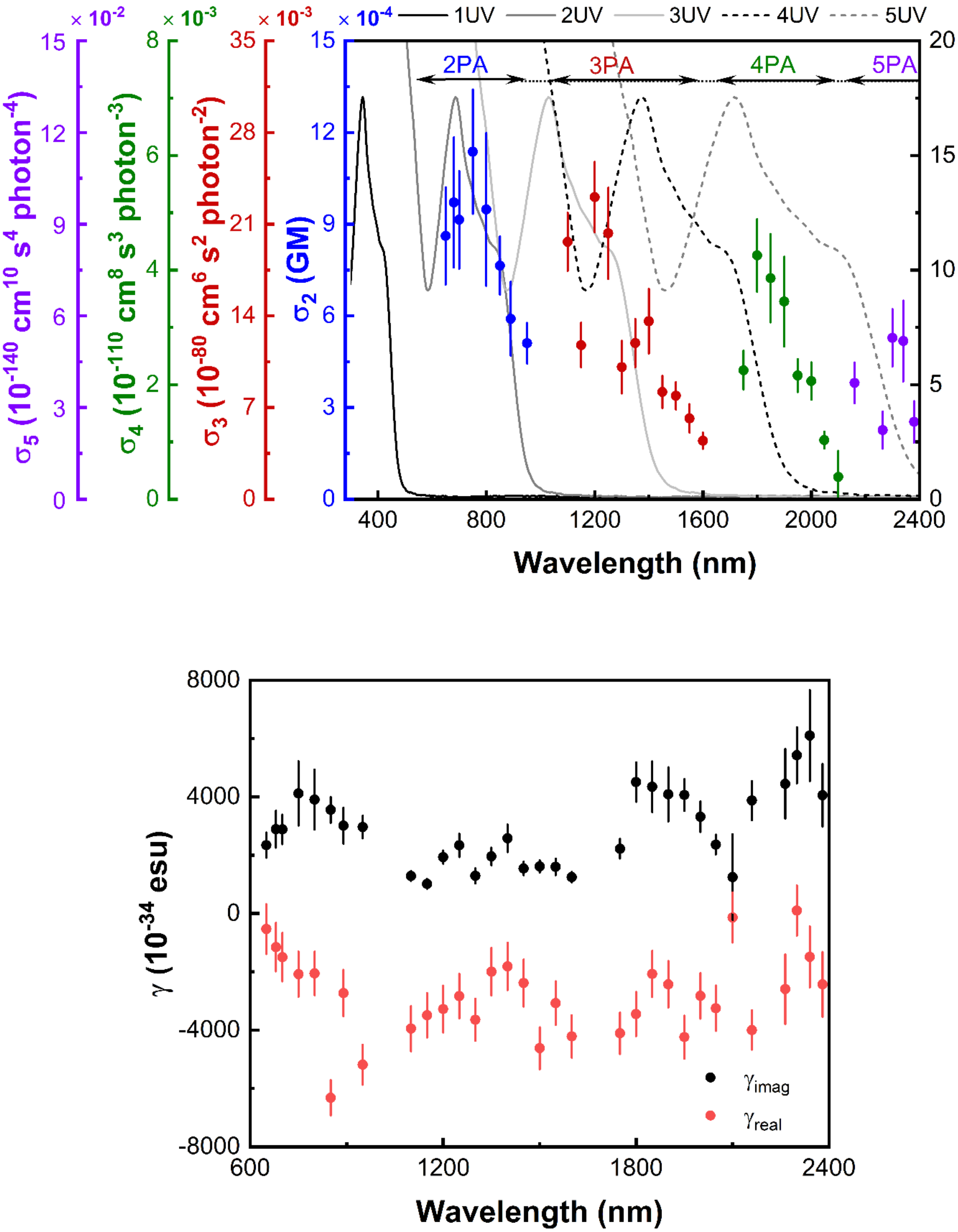


**Fig. S67** Wavelength dependence of the nonlinear absorption (top) and cubic NLO coefficients (bottom) of **3G$_{22,03,02,00}$-Cl**.

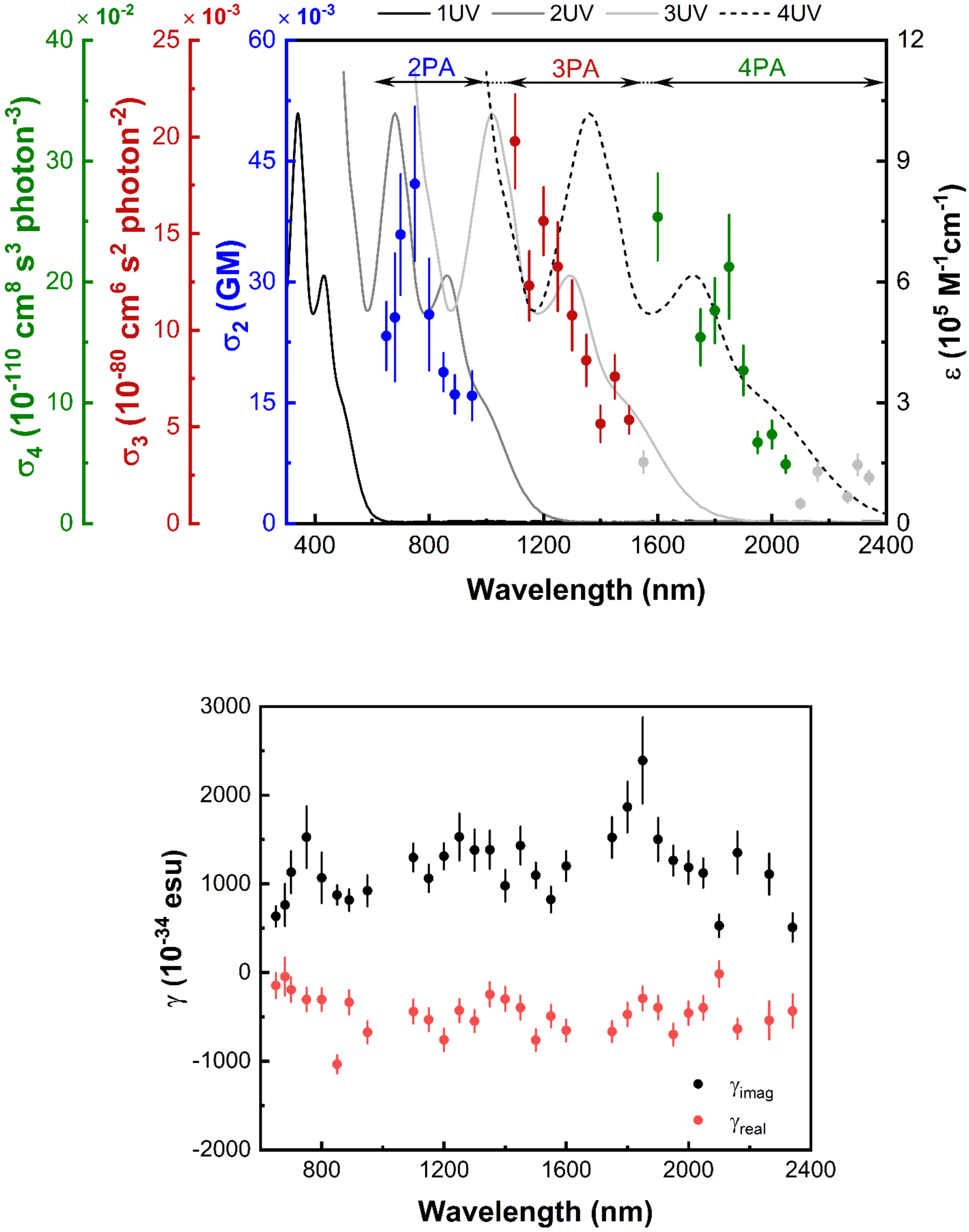


**Fig. S68** Wavelength dependence of the nonlinear absorption (top) and cubic NLO coefficients (bottom) of **$2G_{22,03,01}$-$NO_2$**.

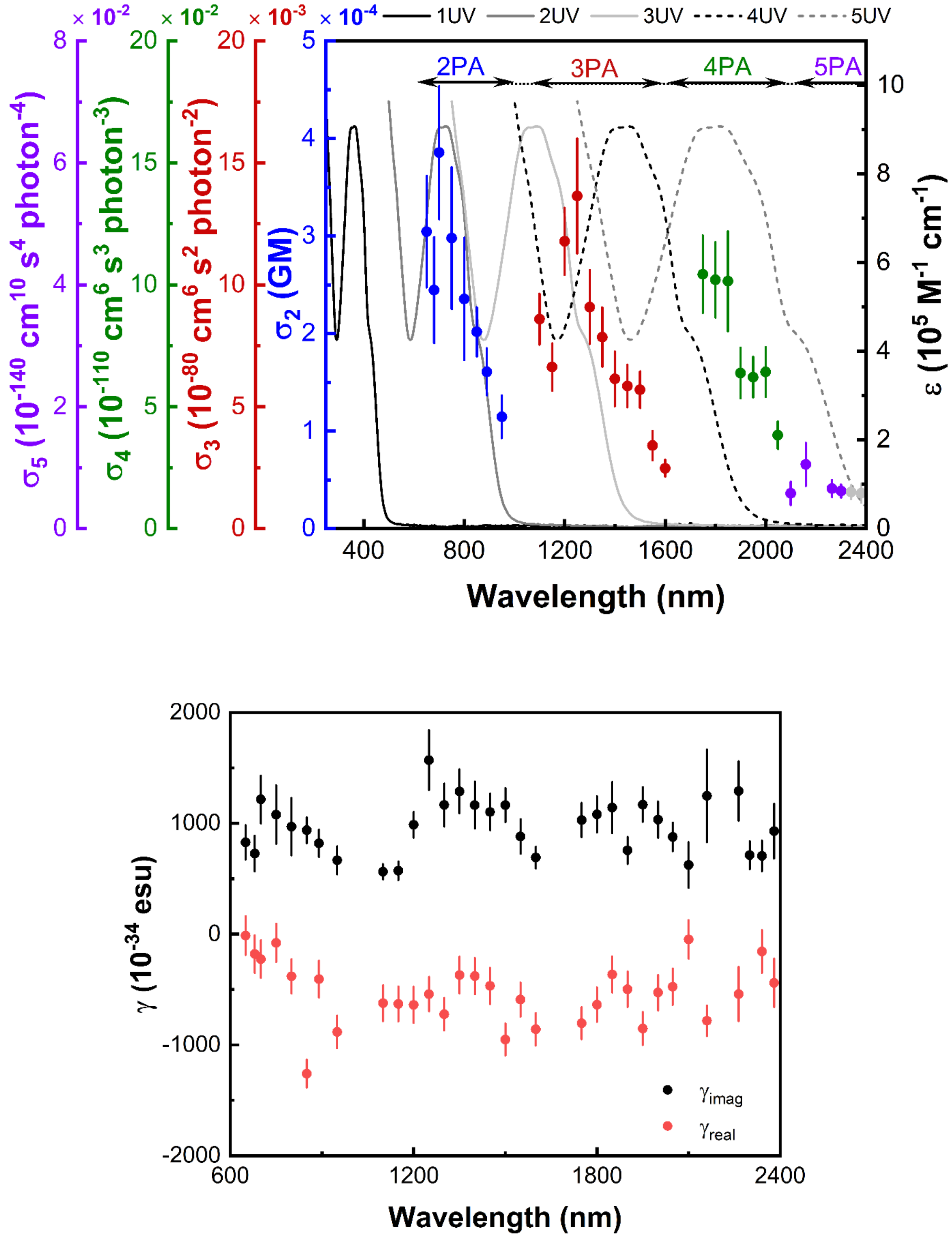


**Fig. S69** Wavelength dependence of the nonlinear absorption (top) and cubic NLO coefficients (bottom) of **$2G_{22,03,00}$-Cl**.

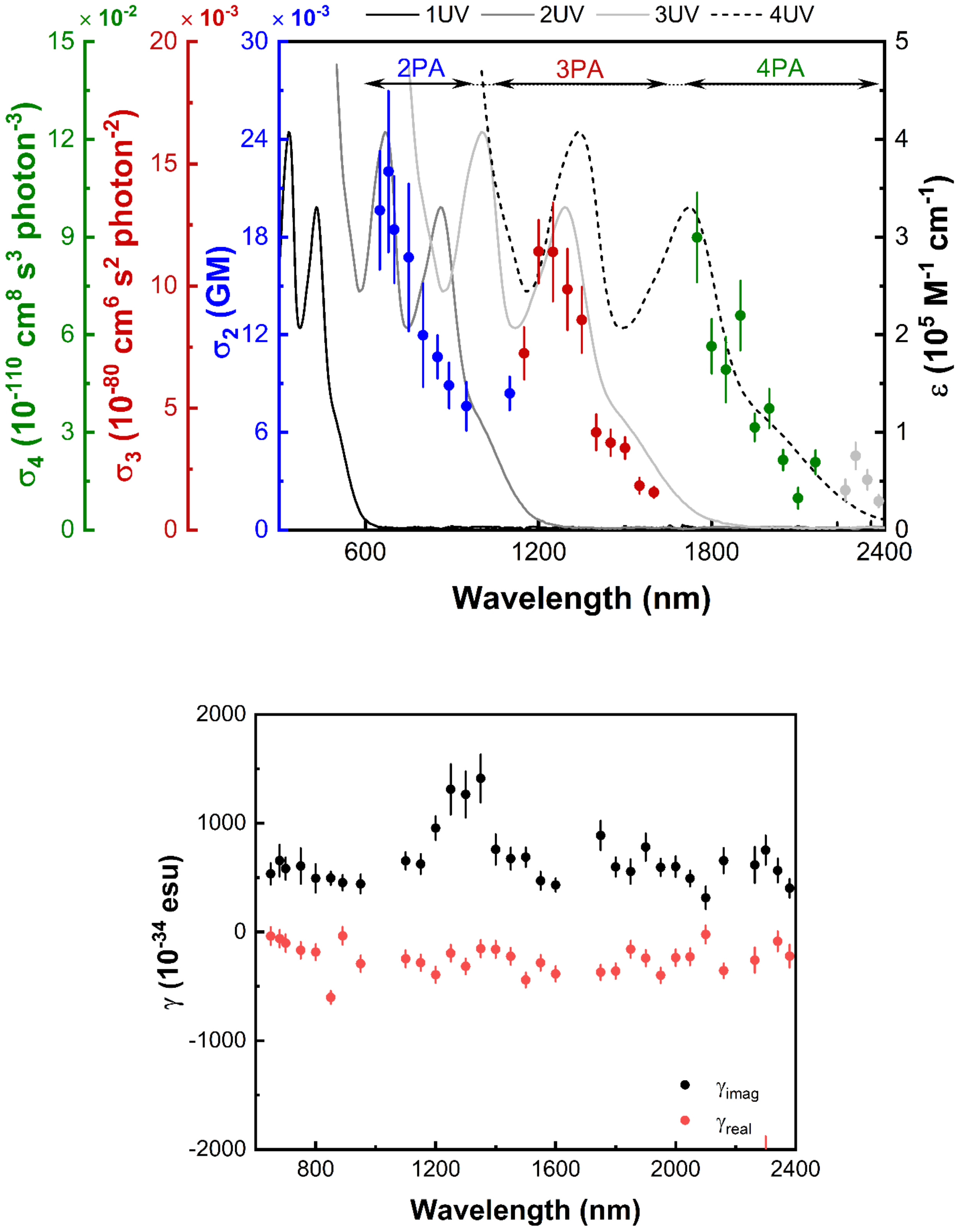


**Fig. S70** Wavelength dependence of the nonlinear absorption (top) and cubic NLO coefficients (bottom) of **1G$_{22,01}$-NO$_2$**.

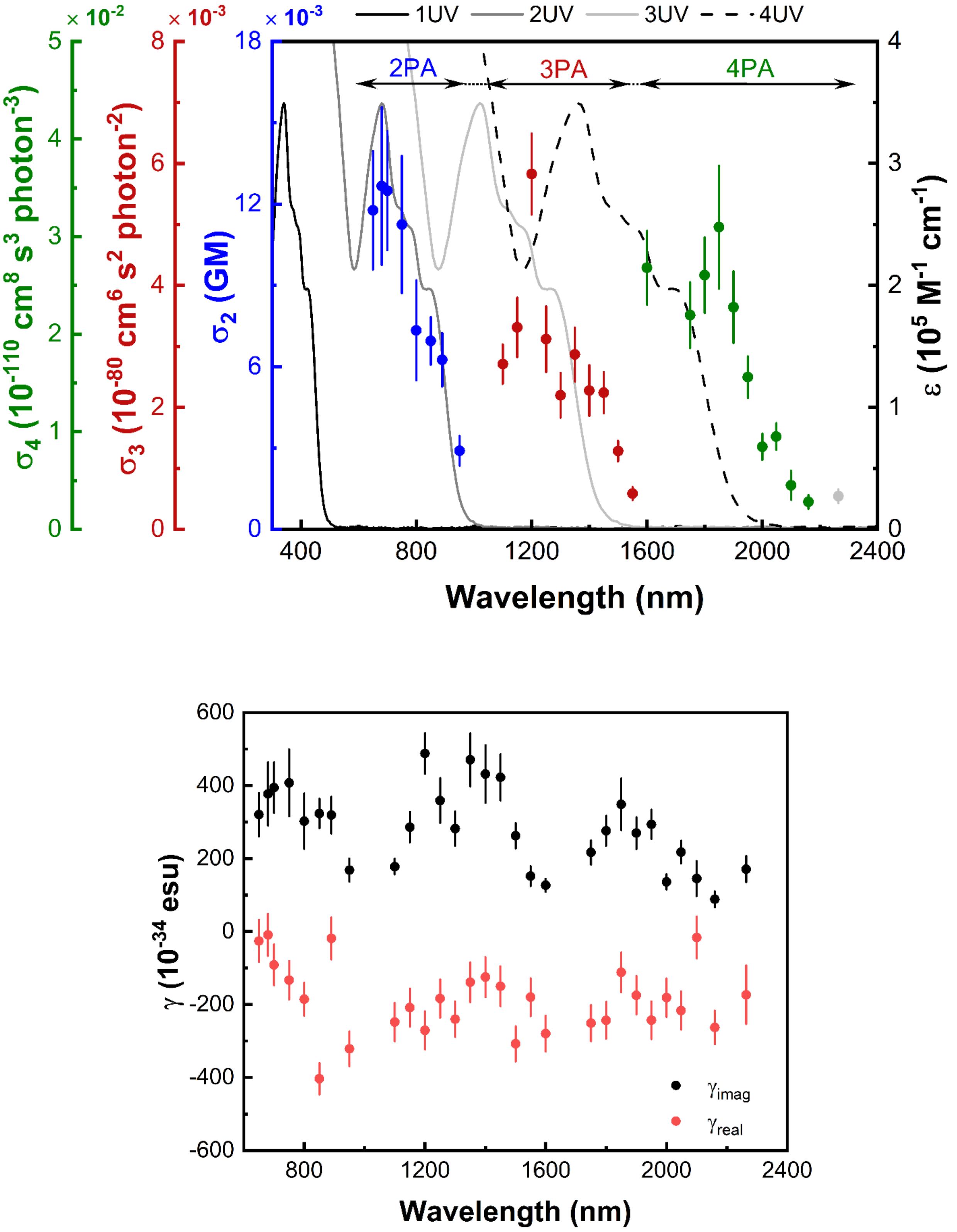


**Fig. S71** Wavelength dependence of the nonlinear absorption (top) and cubic NLO coefficients (bottom) of **1G22,00-Cl**.

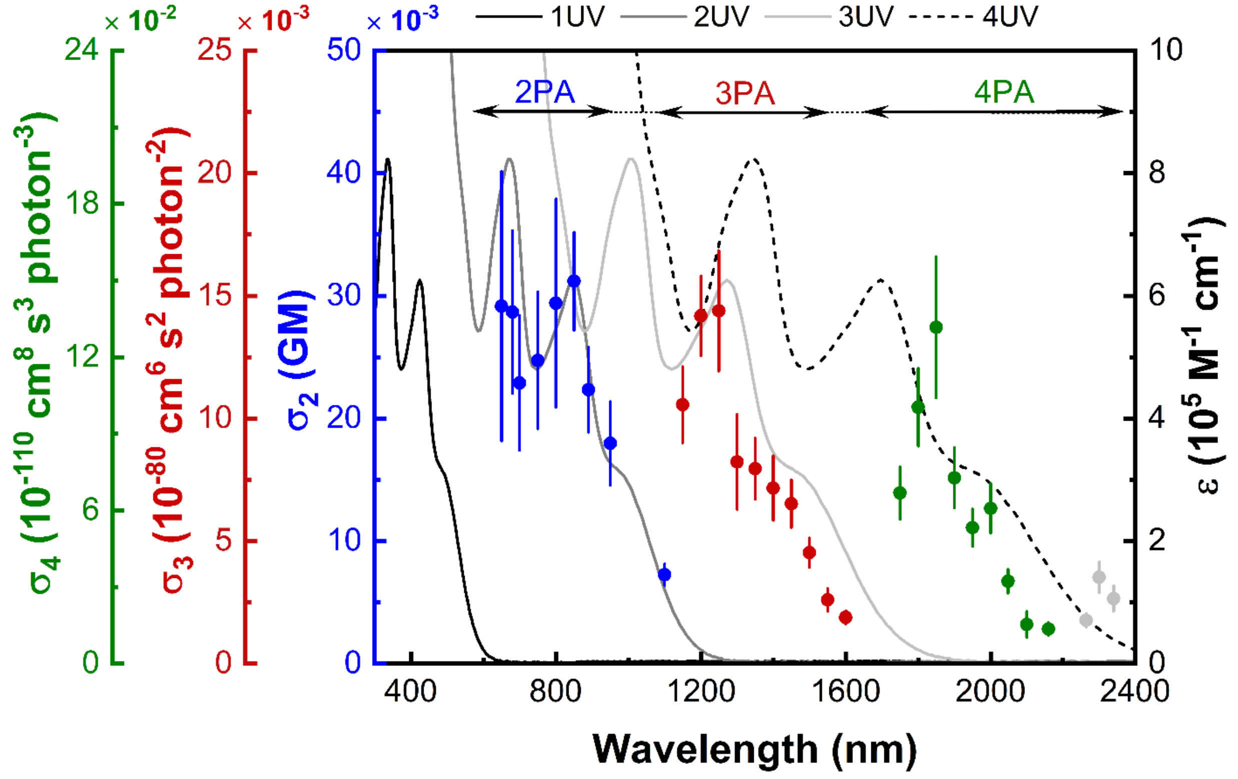


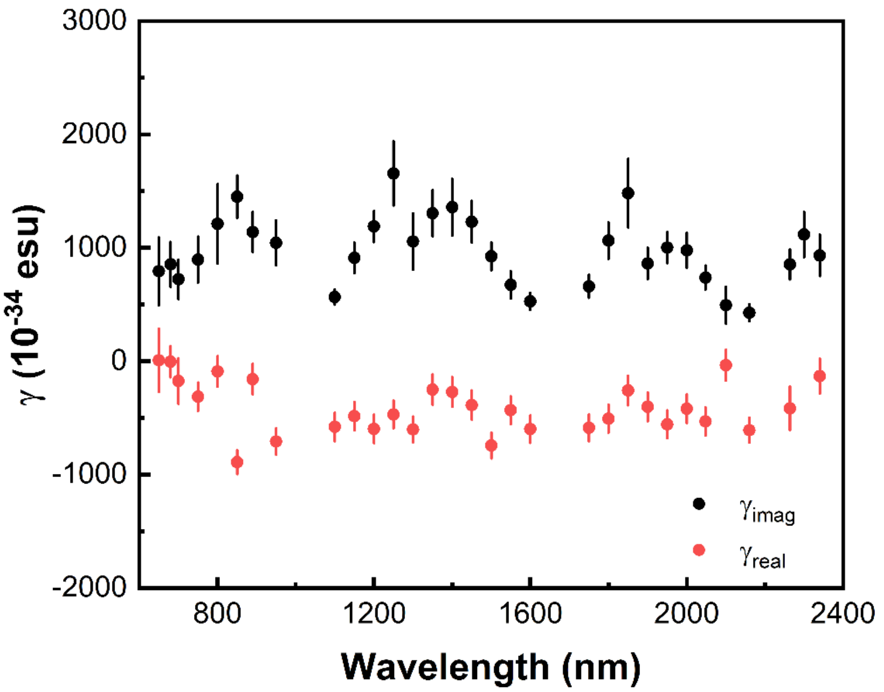


**Fig. S72** Wavelength dependence of the nonlinear absorption (top) and cubic NLO coefficients (bottom) of **$2G_{12,02,01}$-$NO_2$**.

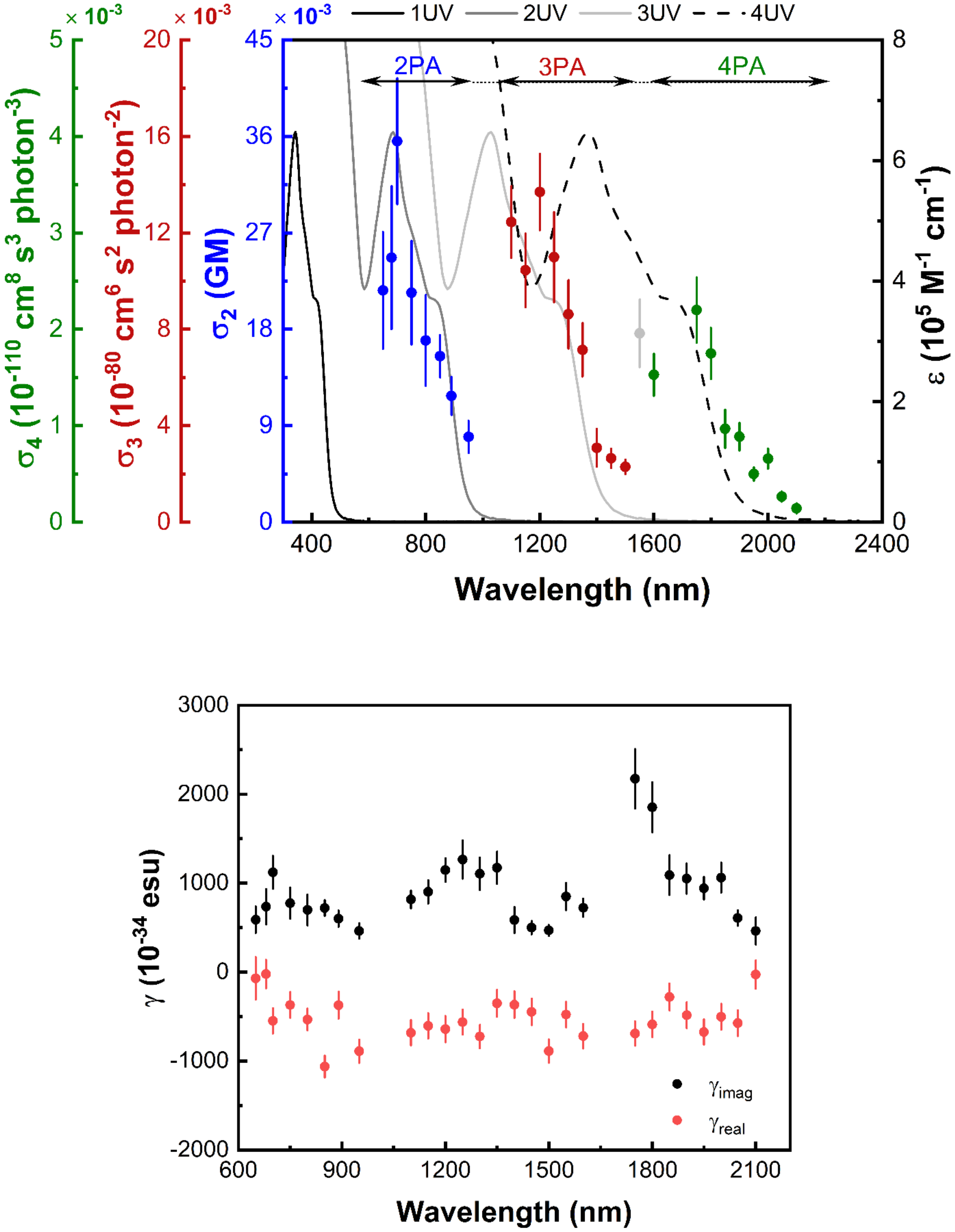


**Fig. S73** Wavelength dependence of the nonlinear absorption (top) and cubic NLO coefficients (bottom) of **2G$_{12,02,00}$-Cl**.

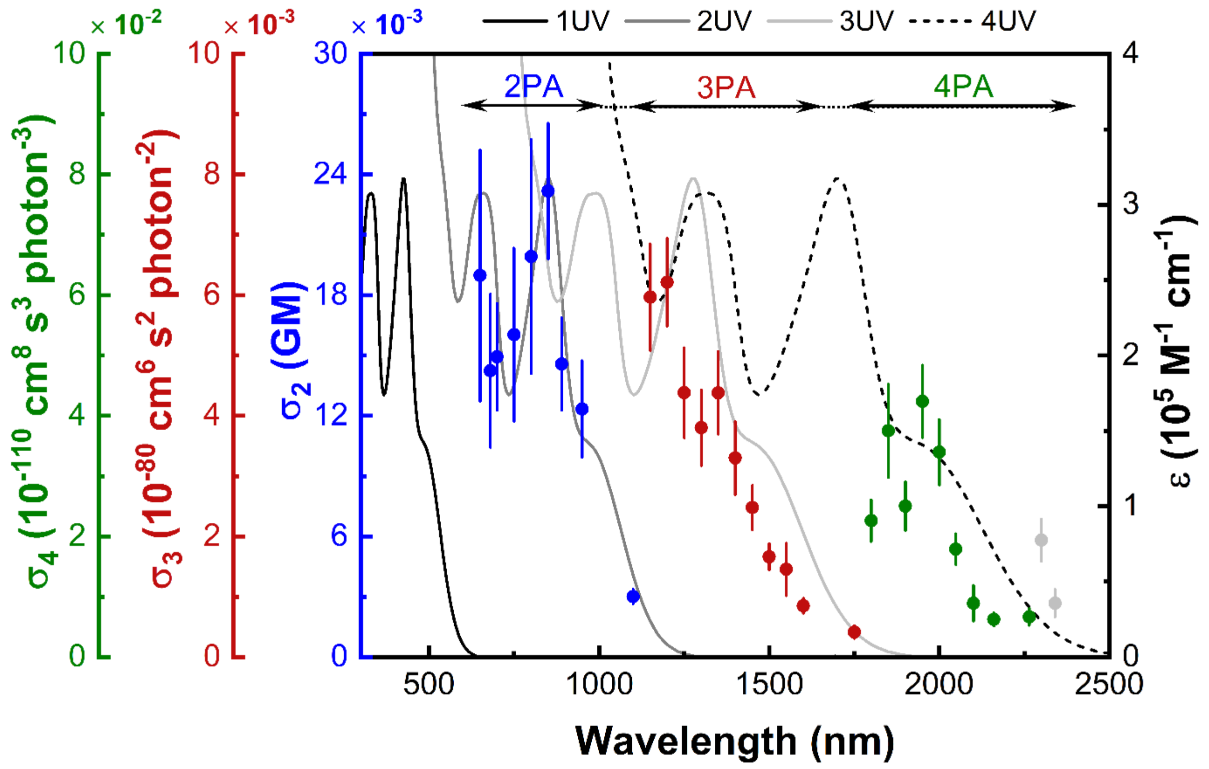


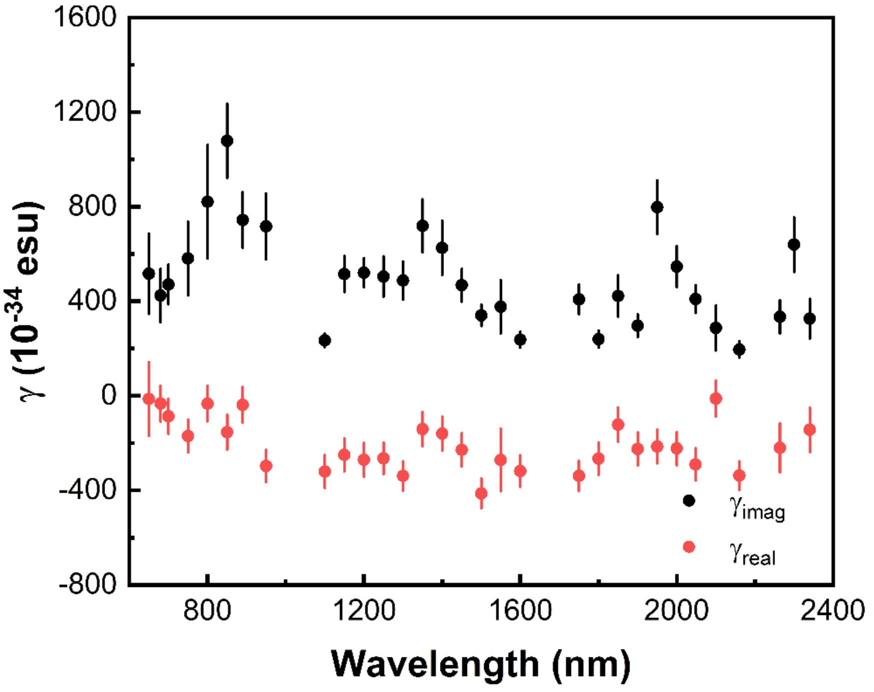


**Fig. S74** Wavelength dependence of the nonlinear absorption (top) and cubic NLO coefficients (bottom) of **1G$_{12,01}$-NO$_2$**.

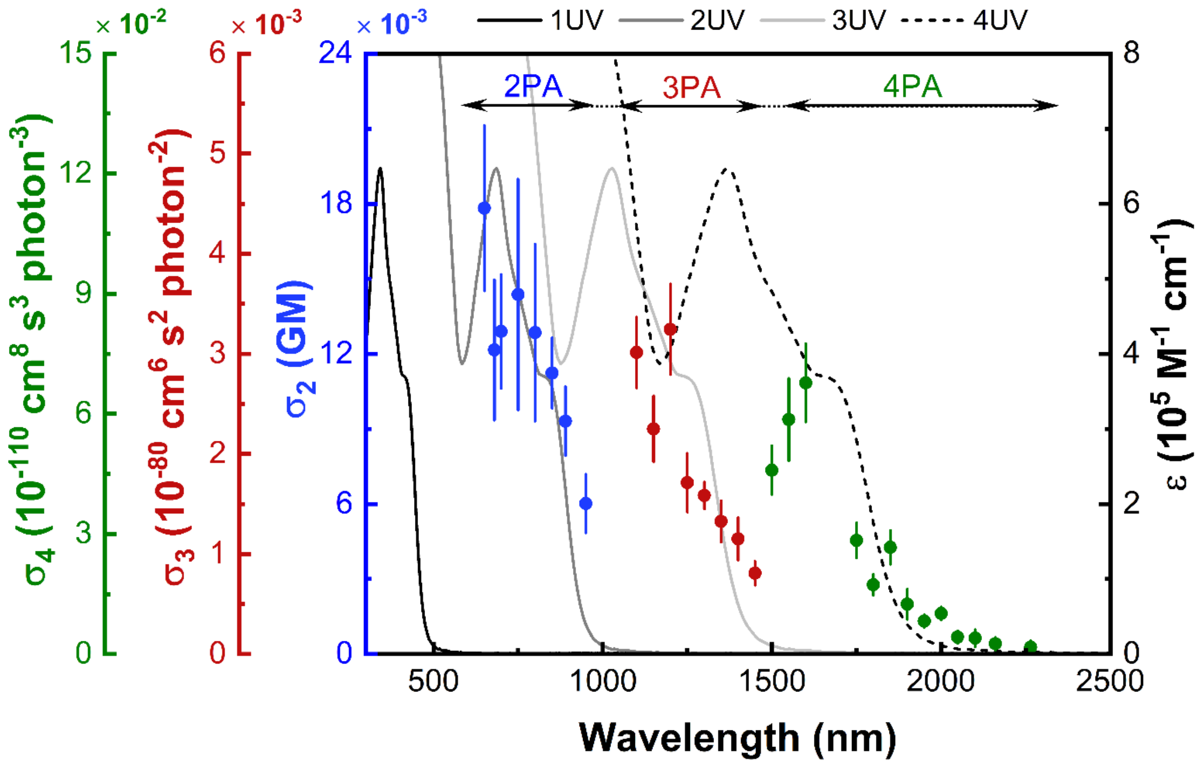


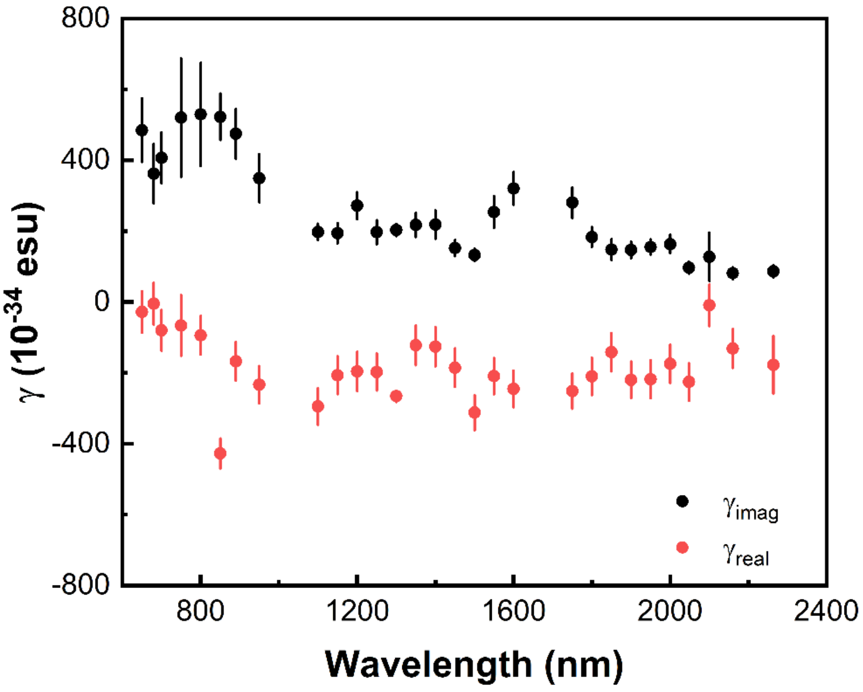


**Fig. S75** Wavelength dependence of the nonlinear absorption (top) and cubic NLO coefficients (bottom) of **1G$_{12,00}$-Cl**.

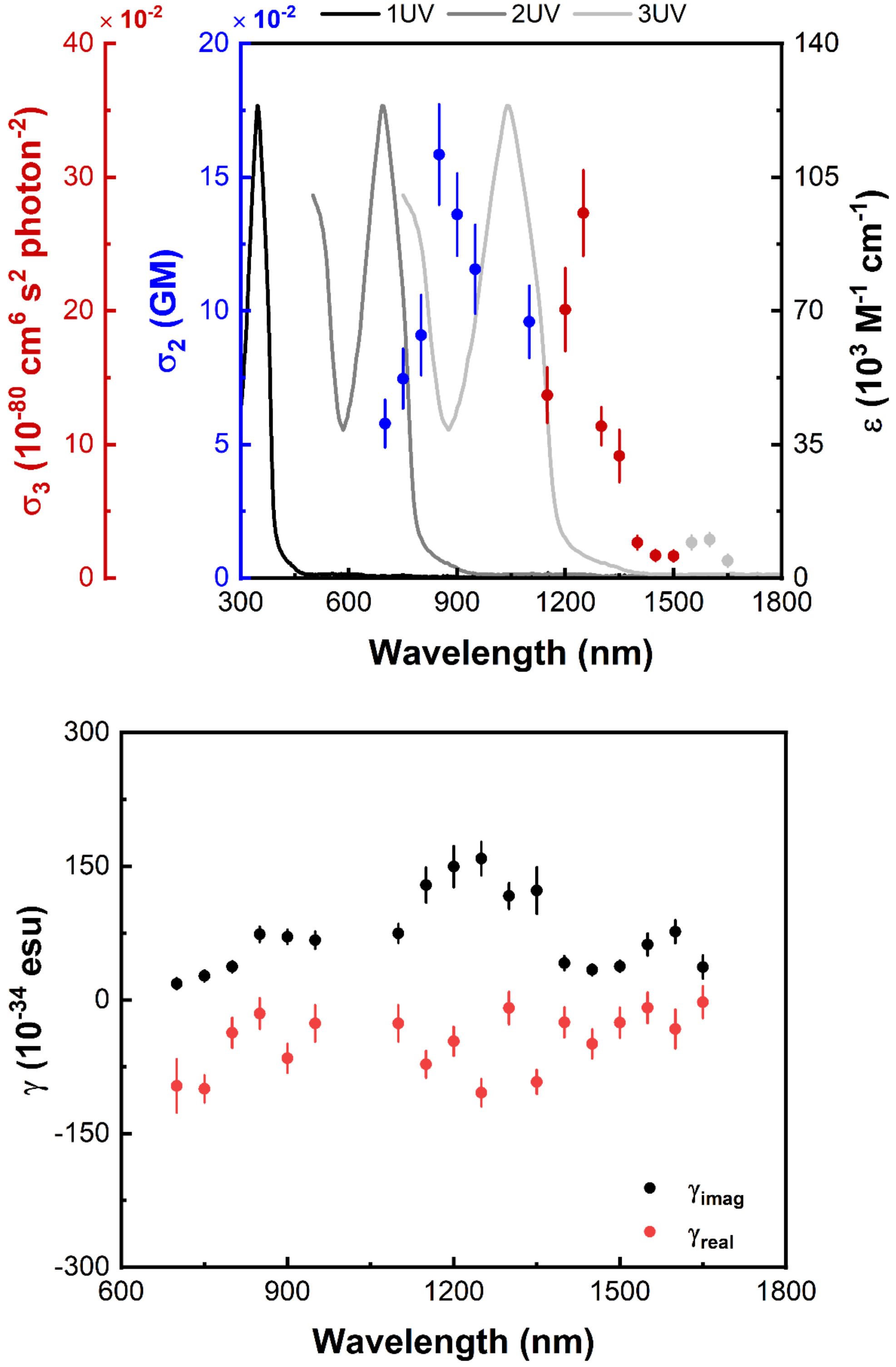


**Fig. S76** Wavelength dependence of the nonlinear absorption (top) and cubic NLO coefficients (bottom) of compound **16**.

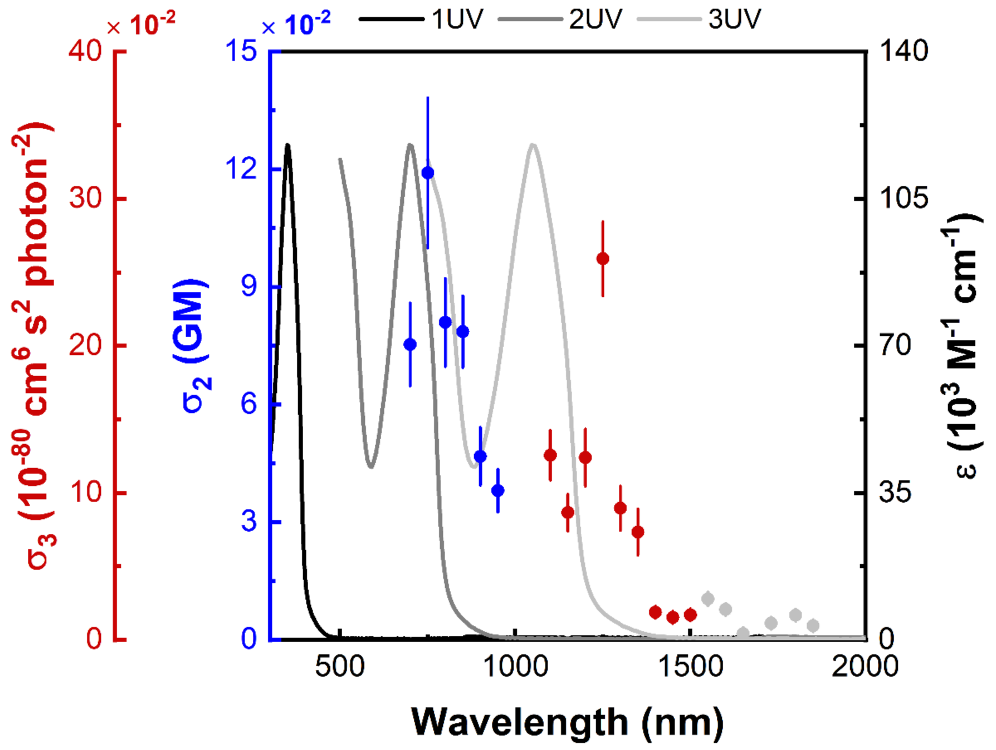


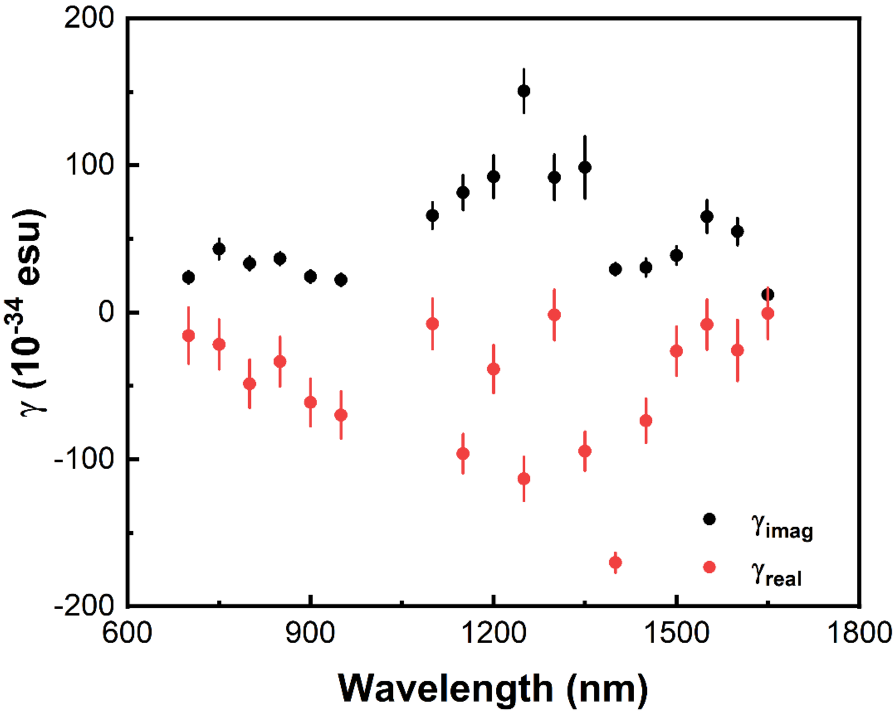


**Fig. S77** Wavelength dependence of the nonlinear absorption (top) and cubic NLO coefficients (bottom) of compound **17**.

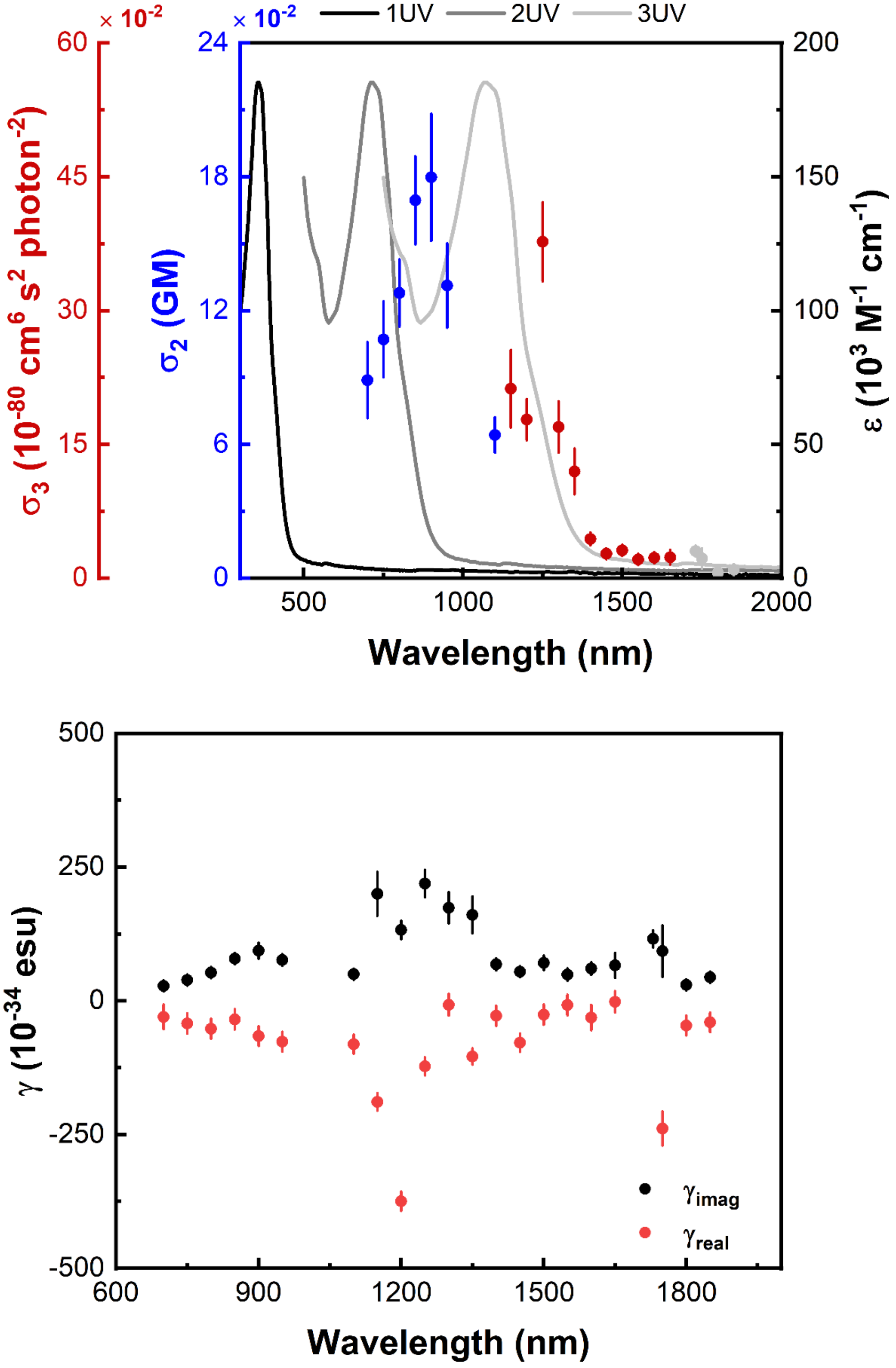


**Fig. S78** Wavelength dependence of the nonlinear absorption (top) and cubic NLO coefficients (bottom) of compound **18**.

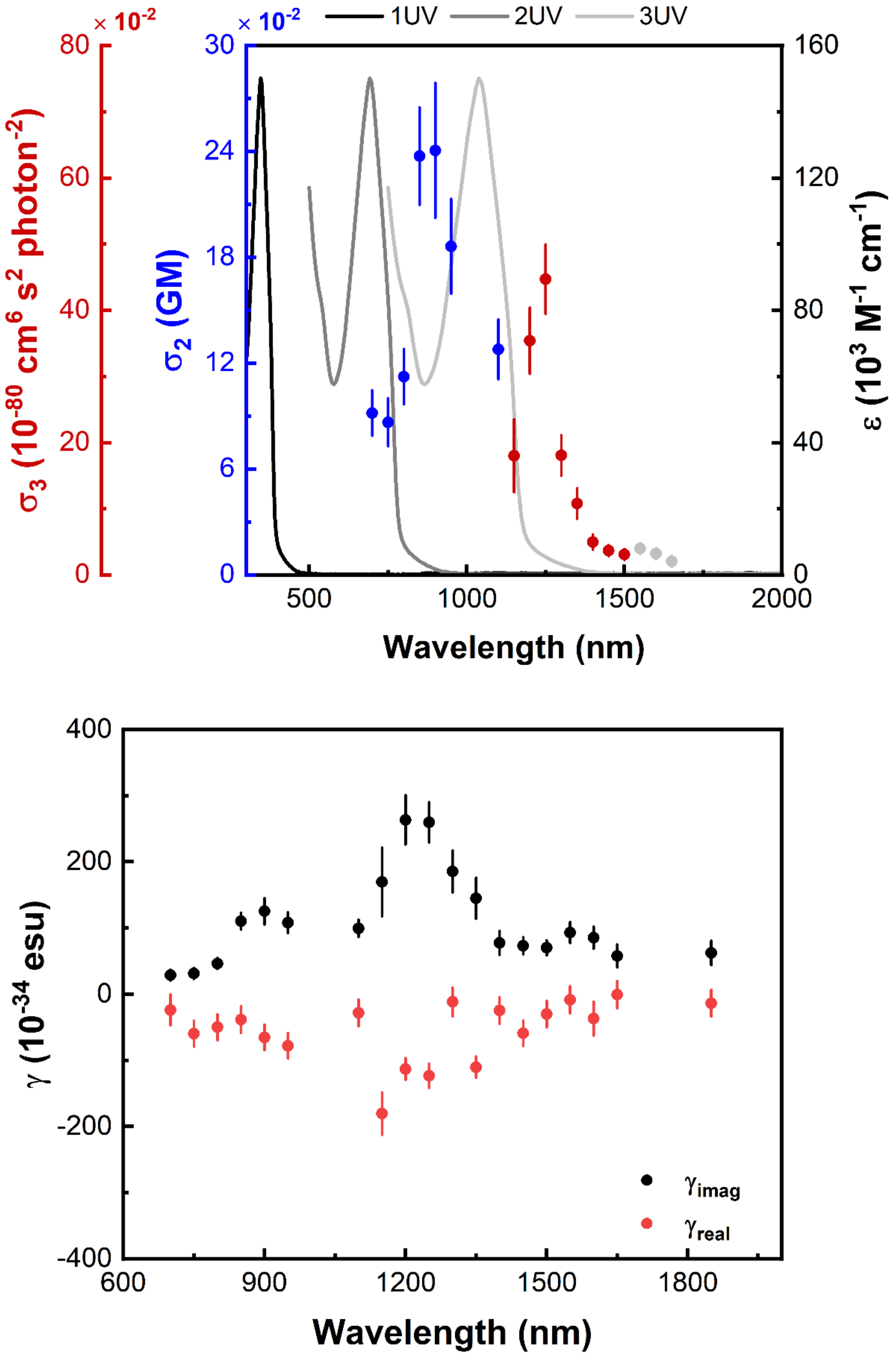


**Fig. S79** Wavelength dependence of the nonlinear absorption (top) and cubic NLO coefficients (bottom) of compound **20**.

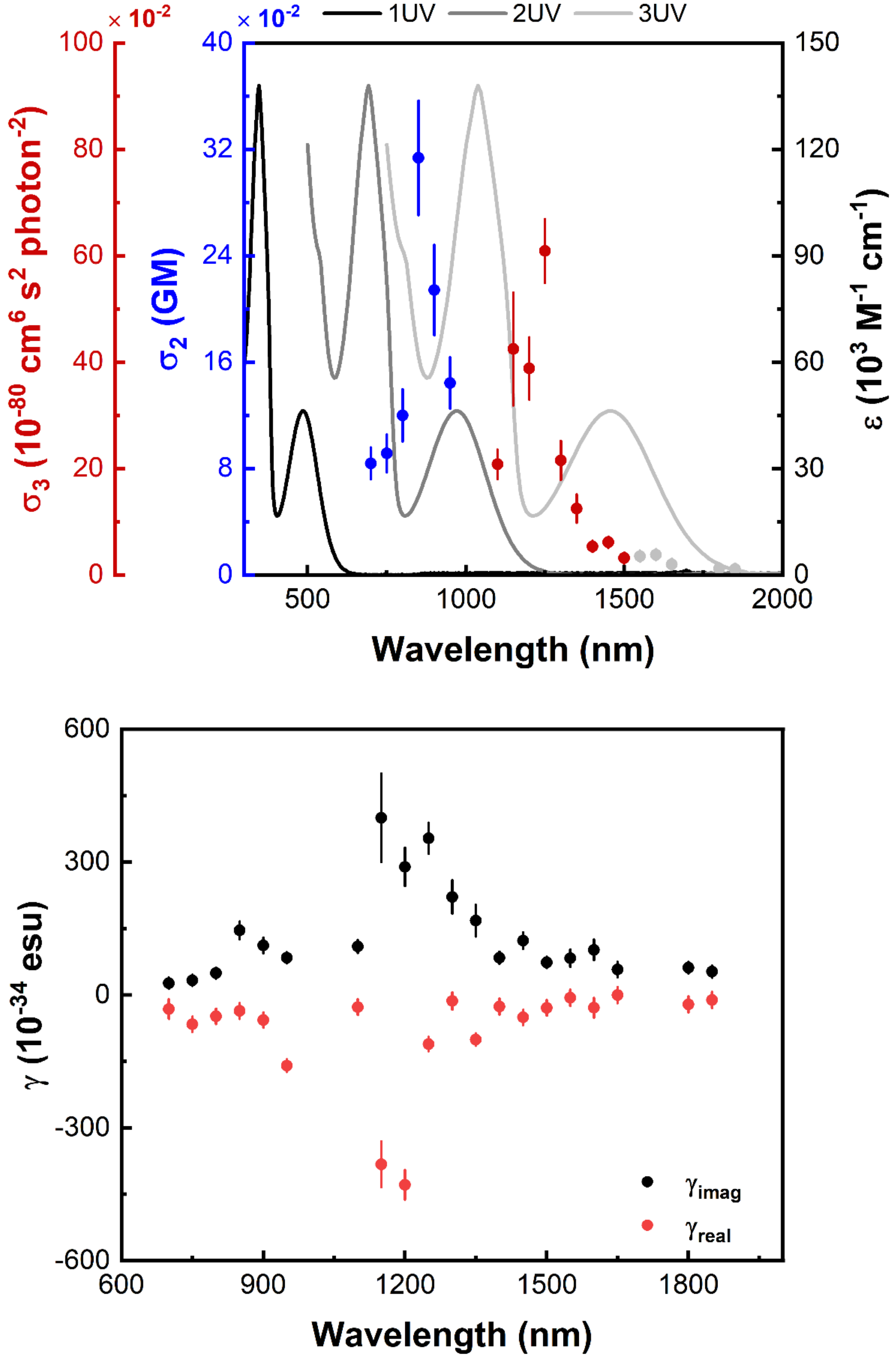


**Fig. S80** Wavelength dependence of the nonlinear absorption (top) and cubic NLO coefficients (bottom) of compound **22**.

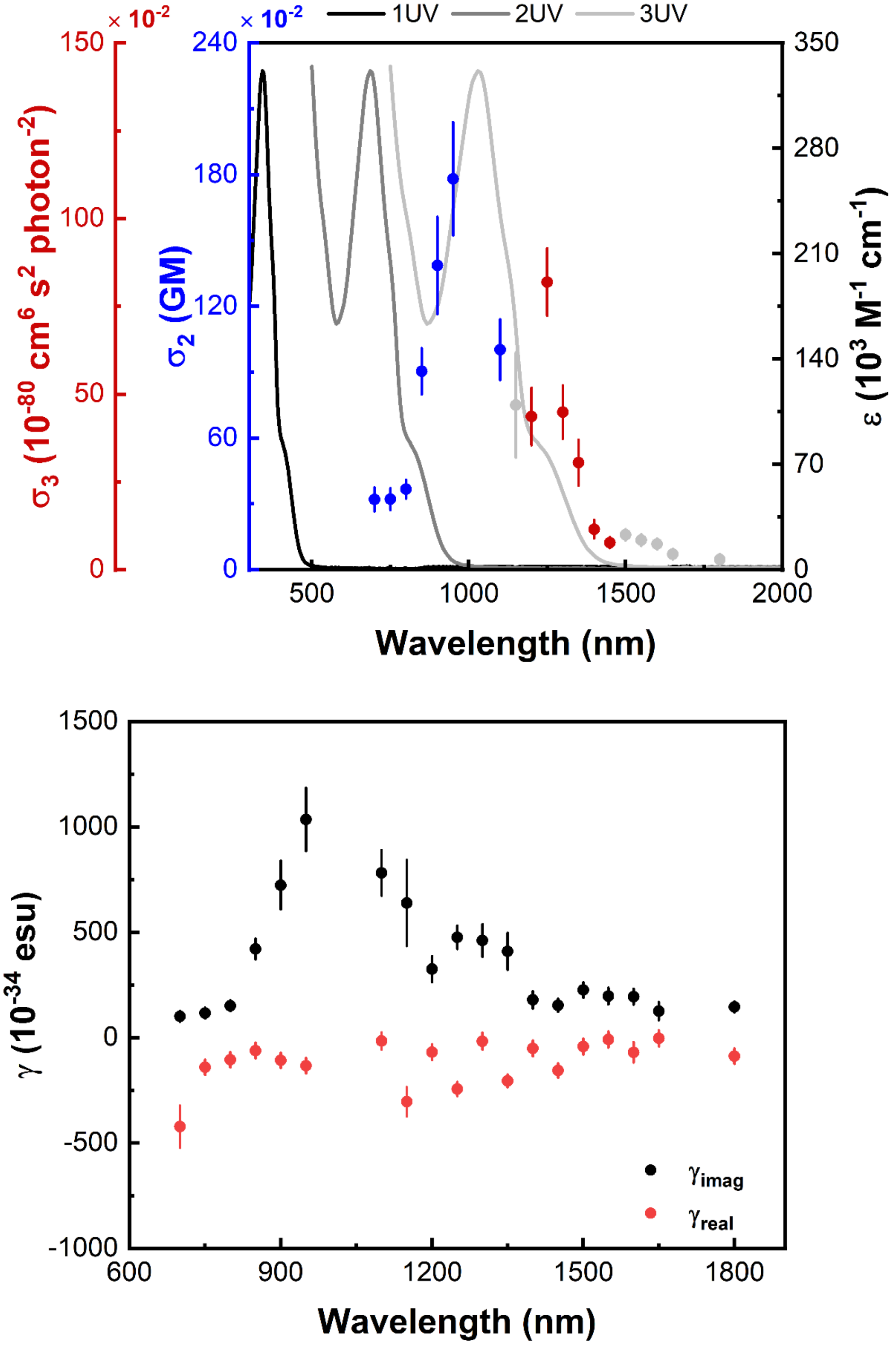


**Fig. S81** Wavelength dependence of the nonlinear absorption (top) and cubic NLO coefficients (bottom) of compound **24**.

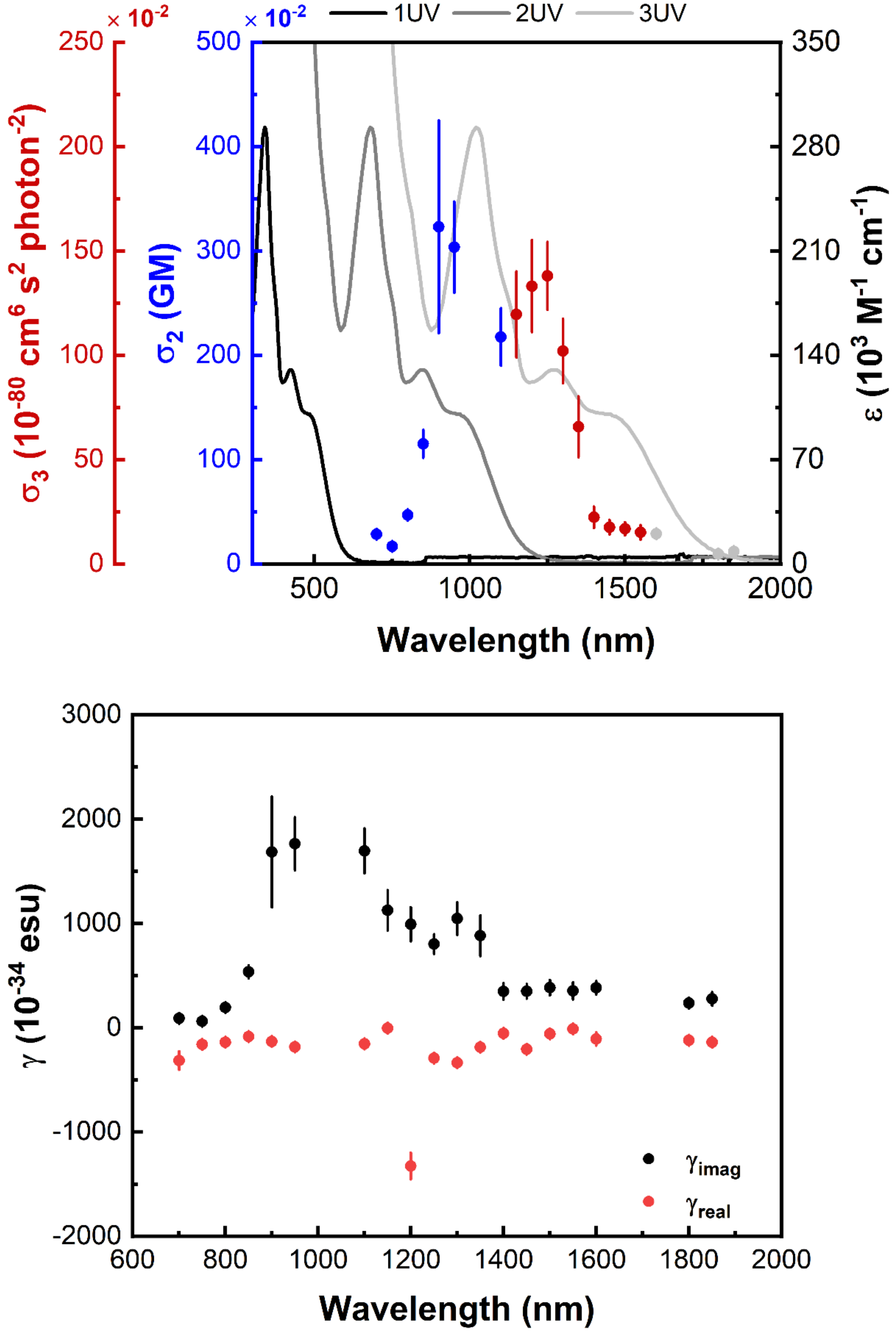


**Fig. S82** Wavelength dependence of the nonlinear absorption (top) and cubic NLO coefficients (bottom) of compound **26**.

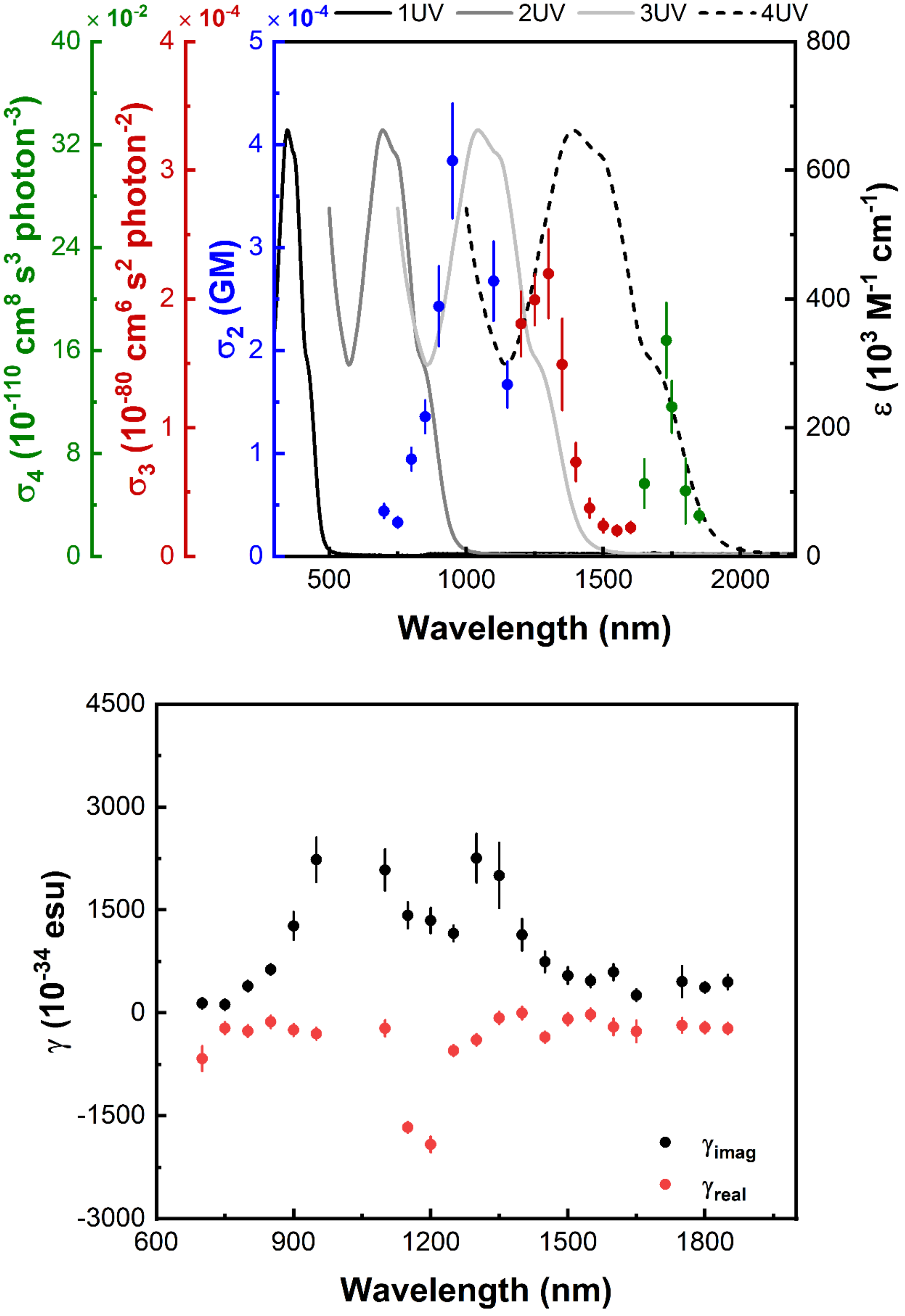


**Fig. S83** Wavelength dependence of the nonlinear absorption (top) and cubic NLO coefficients (bottom) of compound **35**.

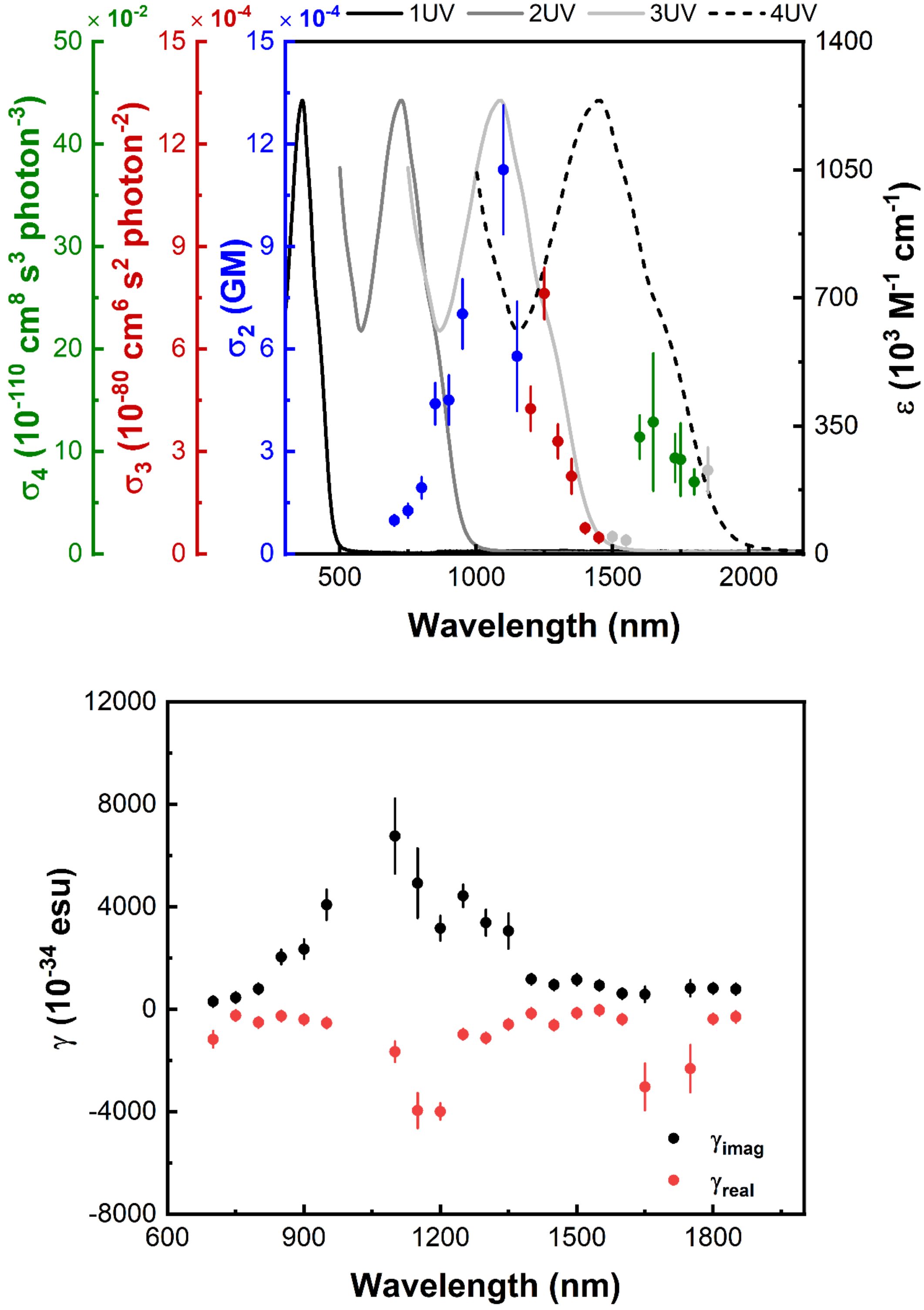


**Fig. S84** Wavelength dependence of the nonlinear absorption (top) and cubic NLO coefficients (bottom) of compound **36**.

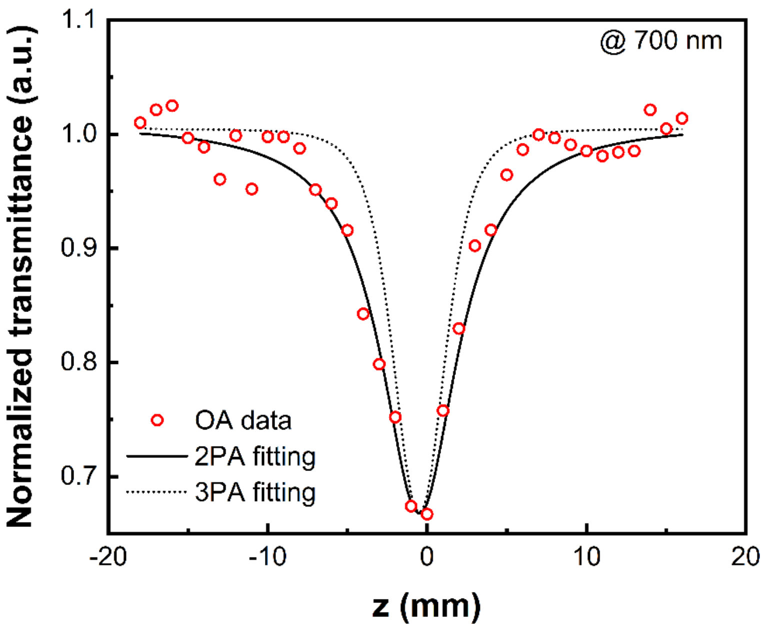


**Figure S85**. The OA Z-scan data obtained at 700 nm for **3G$_{22,03,02,01}$-s** (red circles, 0.26 wt.% in $CH_2Cl_2$) and theoretical curves calculated for $w_0$ = 18 μm (determined from closed-aperture scans for pure solvent) assuming 2PA (solid line) or 3PA (short dot line).

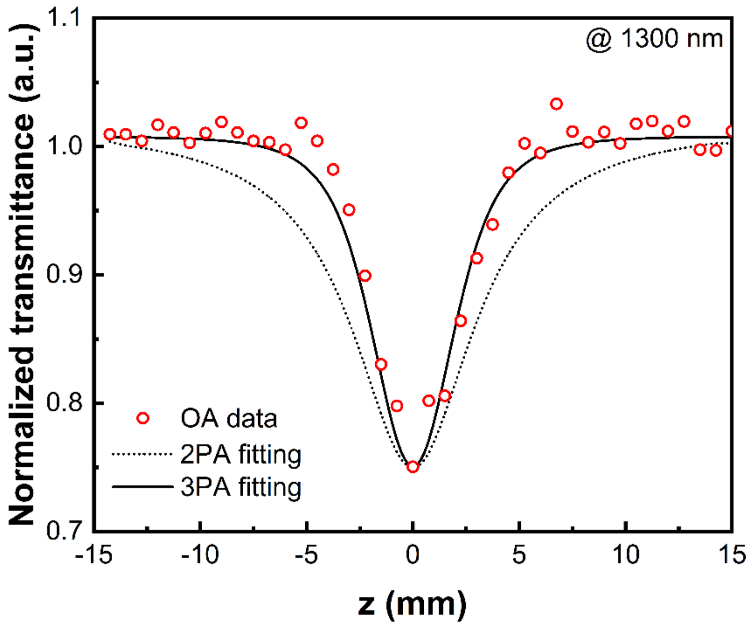


**Figure S86**. The OA Z-scan data obtained at 1300 nm for **3G$_{22,03,02,01}$-s** (red circles, 0.26 wt.% in $CH_2Cl_2$) and theoretical curves calculated for $w_0$ = 35 μm (determined from closed-aperture scans for pure solvent) assuming 2PA (short dot line) or 3PA (solid line).

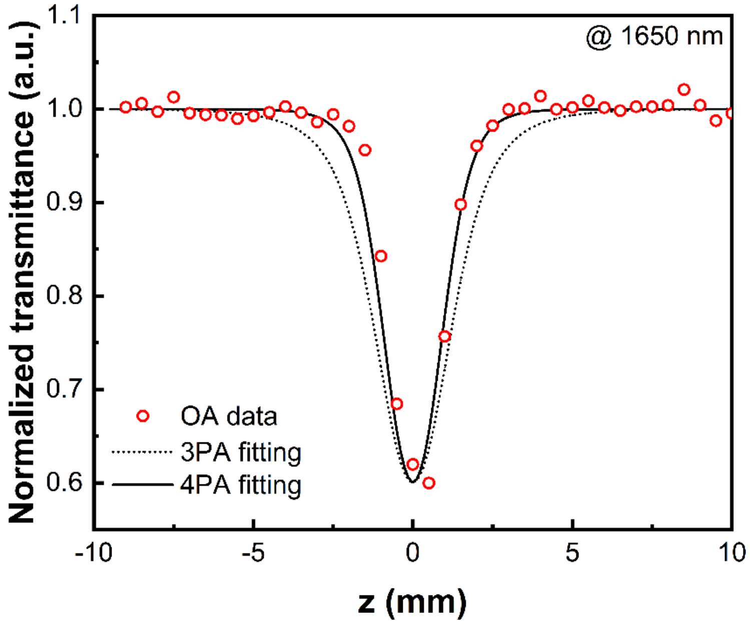


**Figure S87**. The OA Z-scan data obtained at 1650 nm for **3G$_{22,03,02,01}$-s** (red circles, 0.26 wt.% in $CH_2Cl_2$) and theoretical curves calculated for $w_0$ = 38 μm (determined from closed-aperture scans for pure solvent) assuming 3PA (short dot line) or 4PA (solid line).

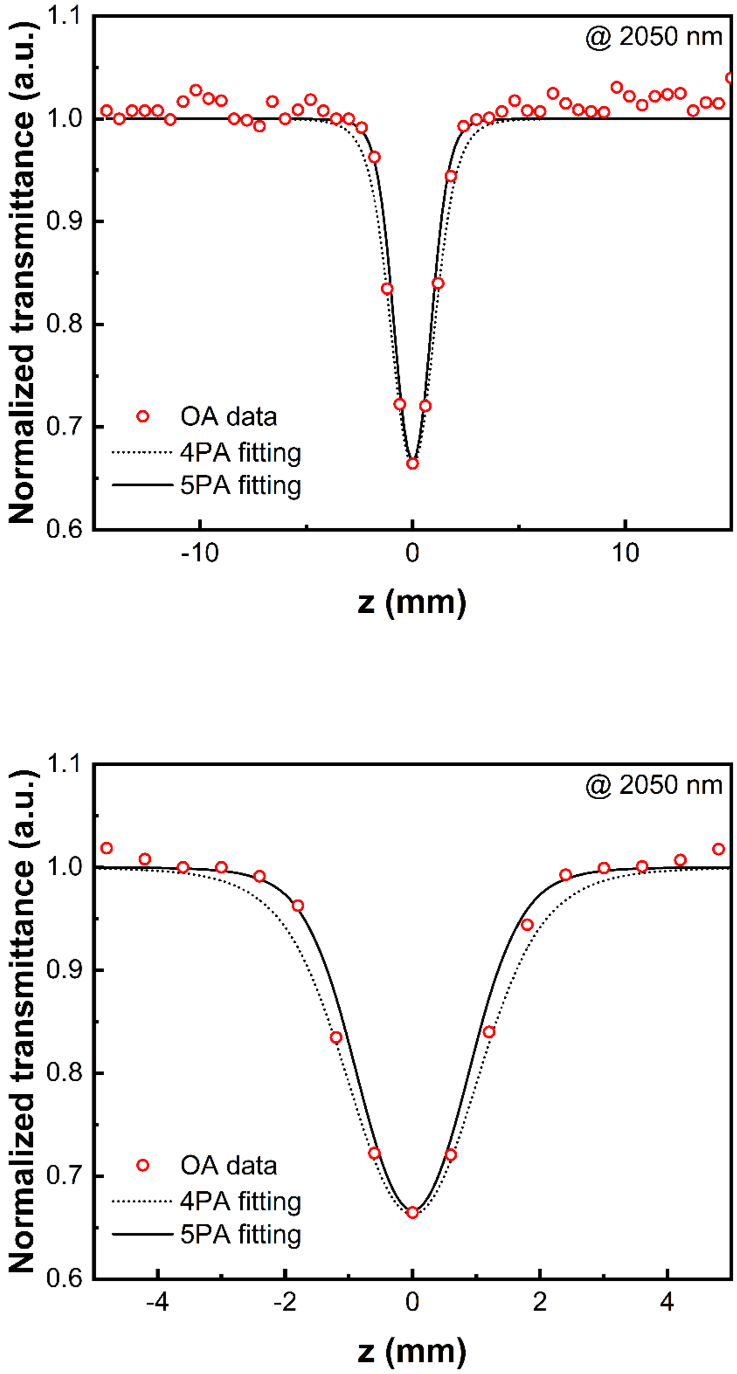


**Figure S88**. The OA Z-scan data obtained at 2050 nm for **3G$_{22,03,02,01}$-s** (red circles, 0.26 wt.% in $CH_2Cl_2$) and theoretical curves calculated for $w_0$ = 32 μm (determined from closed-aperture scans for pure solvent) assuming 4PA (short dot line) or 5PA (solid line).

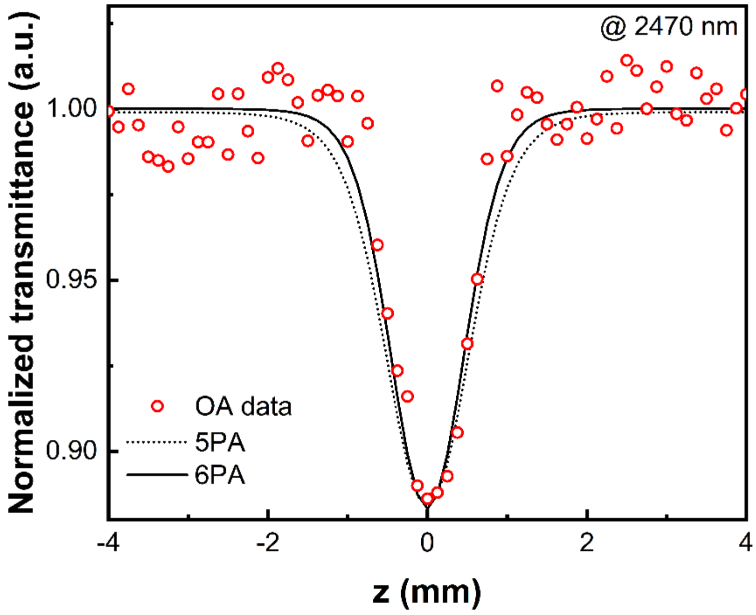


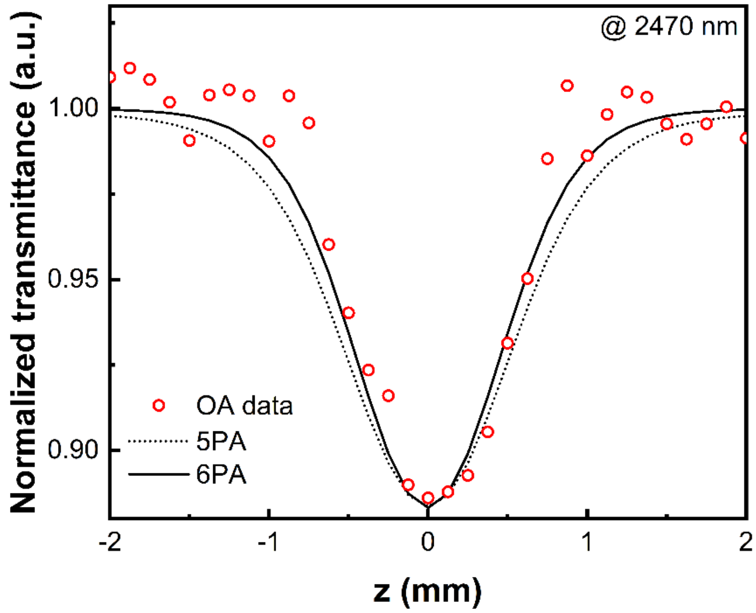


**Figure S89**. The OA Z-scan data obtained at 2470 nm for **3G$_{22,03,02,01}$-s** (red circles, 0.26 wt.% in $CH_2Cl_2$) and theoretical curves calculated for $w_0$ = 29 μm (determined from closed-aperture scans for pure solvent) assuming 5PA (short dot line) or 6PA (solid line).

## Computational studies - 2PA calculations.

2PA cross-sections were calculated with the help of quadratic response theory using the Dalton 2020.1 program.[50] The CAM-B3LYP method[51-54] was employed for all optimized structures with the PCM for solvation ($CH_2Cl_2$: ε = 8.93). The stuttgart_rsc_1997_ecp basis set was used for the transition metal (corresponding to the SDD basis set in Gaussian 16) and the 6-311+G(d) basis set was used for the non-metal atoms. Since the calculation of 2PA spectra is computationally much more demanding than the calculation of the corresponding linear absorption spectra, model complexes **1-M-n** (Fig. S91) were examined, to assess their 2PA performance in comparison with experimental data. Two-photon transition strengths for the ten lowest excited states were calculated with the necessary quadratic response functions (note that **1-M-3** is the largest structure for which calculations could be undertaken).

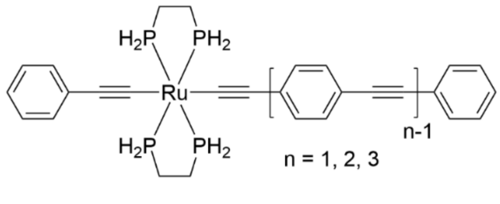

**1-M-n**

**Fig. S90** Model complexes in the 2PA calculations.

The two-photon matrix elements describing an instantaneous, resonant, absorption of two photons with identical energy were computed through a residue of the quadratic response function (Table S12). In the electric dipole approximation, the $(\alpha, \beta)$'$^{\text{th}}$ component of the two-photon transition moment $S^{if}$ between the initial state $i$ and final state $f$ is defined as:

$$S_{\alpha\beta}^{if} = \frac{1}{\hbar}\sum_{k}\left[\frac{\langle i|\mu^{\alpha}|k\rangle\langle k|\mu^{\beta}|f\rangle}{\omega_k - {}^{\omega_f}/_2} + \frac{\langle i|\mu^{\beta}|k\rangle\langle k|\mu^{\beta\alpha}|f\rangle}{\omega_k - {}^{\omega_f}/_2}\right] \quad (5)$$

where $\langle i|\mu^{\alpha}|k\rangle$ is the transition dipole moment between the electronic states $i$ and $k$ along the Cartesian axis α $(\alpha, \beta \in \{x, y, z\})$, and $\omega_k$ and $\omega_f$ denote the excitation energies of the virtual excited state $k$ and the final excited state $f$, respectively. For linearly polarized light with parallel polarization, the 2PA transition strength in atomic units can be described as:

$$\delta_{2PA} = \frac{1}{15}\sum_{\alpha\beta}\left(2S_{\alpha\beta}\bar{S}_{\alpha\beta} + S_{\alpha\alpha}\bar{S}_{\beta\beta}\right) \quad (6)$$

where the sum is over the Cartesian components α and β, and with the bar indicating complex conjugation. The conversion of computationally obtained $\delta_{2PA}$ data (in atomic units) into a form that may be compared with the macroscopic 2PA cross-section $\sigma_2$ in cgs units can be implemented via:[55]

$$\sigma_{2PA} = \frac{N\pi^3 a a_0^5 \omega^2}{c_0} g(2\omega, \omega_0, \Gamma)\delta_{2PA} \quad (7)$$

where $N$ is an integer value that depends on the specific experimental setup, and where $N = 4$ for single-beam experimental data such as that from the Z-scan technique. In the above equation, $\alpha$ is the fine structure constant, $a_0$ is the Bohr radius, $c_0$ is the speed of light, $\omega_0$ and $g(2\omega, \omega_0, \Gamma)$ is the lineshape function describing spectral broadening effects with $\omega_0$ the excitation energy. The broadening factor $\Gamma$ is introduced as an empirical damping parameter to describe the broadening effects, correcting for the infinitely sharp calculated vertical excitations, and allowing for comparison to experimental peaks, in which rovibrational excitations and collisional dynamics also play a role. Here $\Gamma$ is defined as the half width at half maximum (HWHM) and is chosen to be 0.1 eV in the calculations.[56] A Gaussian lineshape function is used for $g(2\omega, \omega_0, \Gamma)$,

$$g(2\omega) = \frac{\sqrt{ln2}}{\Gamma\sqrt{\pi}} exp\left[-ln2\left(\frac{2\omega - \omega_0}{\Gamma}\right)^2\right] \quad (8)$$

where $\omega$ is the photon energy and $\omega_0$ is the excitation energy. The common units for 2PA cross-section are Göppert-Mayer units with 1 GM corresponding to $10^{-50}$ $cm^4$ s $photon^{-1}$.

**Table S12.** Two-photon transition moments of model complexes in the Cartesian coordinate system.

| Complex | Symm. | No. | Energy (eV)[a] | $S_{xx}$[b] | $S_{yy}$[b] | $S_{zz}$[b] | $S_{xy}$[b] | $S_{xz}$[b] | $S_{yz}$[b] |
|---|---|---|---|---|---|---|---|---|---|
| **1-M-1** | $A_1$ | 1 | 4.05 | 0 | 0 | 0 | -19.4 | 0 | 0 |
| | $A_1$ | 2 | 4.21 | -1.5 | 0 | 0.1 | 0 | 0 | -2.8 |
| | $A_1$ | 3 | 4.26 | -0.1 | 0 | 0 | 0 | -15.1 | 0 |
| | $A_1$ | 4 | 4.32 | -343.4 | 7.8 | 13 | 0 | 0 | 0 |
| | $A_1$ | 5 | 4.4 | -68.3 | 3.6 | -2.4 | 0 | 0 | 0 |
| | $A_1$ | 6 | 4.41 | 0 | 0 | 0 | 0 | 8.3 | 0 |
| | $A_1$ | 7 | 4.42 | 0.4 | 0 | 0 | 0 | 0 | 2.7 |
| | $A_1$ | 8 | 4.67 | -0.1 | 0 | 0 | 10.9 | 0 | 0 |
| | $A_1$ | 9 | 4.72 | 0.1 | 0 | 0 | 4.2 | -29.3 | 0 |
| | $A_1$ | 10 | 4.73 | 0 | 0 | 0 | 46.7 | 2.6 | 0 |
| **1-M-2** | $A_1$ | 1 | 3.52 | -528.7 | 6.4 | 5 | 0 | 0 | -2 |
| | $A_1$ | 2 | 4 | 25.1 | 1.1 | -1.4 | 0 | 0 | 1.2 |
| | $A_1$ | 3 | 4.07 | 0 | 0 | 0 | -27.1 | -4.6 | 0 |
| | $A_1$ | 4 | 4.28 | 0.4 | 0 | 0 | -1.9 | -22.4 | 0 |
| | $A_1$ | 5 | 4.33 | -263.5 | 5.6 | 8.9 | 0.1 | -0.1 | 0.8 |
| | $A_1$ | 6 | 4.45 | -0.3 | 0 | 0 | -6.6 | -23.4 | 0 |
| | $A_1$ | 7 | 4.49 | -92.1 | 2.5 | -1.4 | 0 | 0 | -2.2 |
| | $A_1$ | 8 | 4.54 | 0.5 | 0 | 0 | 7.9 | -5 | 0 |
| | $A_1$ | 9 | 4.59 | -540.7 | 2.7 | 7.6 | 0 | 0.1 | -0.3 |
| | $A_1$ | 10 | 4.67 | 0.8 | 0 | 0 | -18.2 | -14.8 | 0 |
| **1-M-3** | $A_1$ | 1 | 3.25 | -741.2 | 6 | 3.9 | 0 | 0 | 1.9 |
| | $A_1$ | 2 | 3.89 | -187.5 | 0.8 | 2.7 | 0 | 0 | 1.4 |
| | $A_1$ | 3 | 3.98 | 713 | -5.6 | -4.4 | 0 | 0 | -1 |
| | $A_1$ | 4 | 4.08 | 0 | 0 | 0 | 26.6 | -5.5 | 0 |
| | $A_1$ | 5 | 4.26 | 0 | 0 | 0 | 1.5 | -26.9 | 0 |
| | $A_1$ | 6 | 4.33 | -381.7 | 4.4 | 9.2 | 0 | 0 | 0.2 |
| | $A_1$ | 7 | 4.44 | 209.1 | -4.6 | 1.2 | 0 | 0 | -0.5 |
| | $A_1$ | 8 | 4.48 | 0.1 | 0 | 0 | -8 | 25.7 | 0 |
| | $A_1$ | 9 | 4.53 | -0.1 | 0 | 0 | 1.3 | 0.7 | 0 |
| | $A_1$ | 10 | 4.63 | 0.1 | 0 | 0 | -23.5 | 17.7 | 0 |

[a] Following convention,[51,57-62] the energies are reported in eV. [b] The two-photon transition moment $S_{if}$ between the initial state $i$ and final state $f$ along the Cartesian axis. $S$ in atomic units. 1 a.u. = $1.896788\times10^{-50}$ $cm^4$ s $photon^{-1}$.

## References


1 P. V. Simpson, L. A. Watson, A. Barlow, G. Wang, M. P. Cifuentes and M. G. Humphrey, *Angew. Chem. Int. Ed.,* 2016, **55**, 2387.

2 L. Zhang, M. Morshedi and M. G. Humphrey, *Angew. Chem. Int. Ed.,* 2022, **61***,* e202116181.

3 L. Zhang, S. Ma, T. Wang, S. Li, L. Wang, D. Li, Y. Tian and Q. Zhang, *Anal. Chem.,* 2023, **95**, 1635.

4 H. Wang, M. Morshedi, M. S. Kodikara, Y. de Coene, K. Clays, C. Zhang and M. G. Humphrey, *Angew. Chem. Int. Ed.,* 2023, **62**, e202301754.

5 L. Zhang, M. Morshedi, M. S. Kodikara and M. G. Humphrey, *Angew. Chem. Int. Ed.,* 2022, **61**, e202208168.

6 M. Shanu, J. N. Acharyya, M. Sankar and G. Vijaya Prakash, *Inorg. Chem.,* 2023, **62**, 12895.

7 Z. Zheng, H. Zhang, H. Cao, J. Gong, M. He, X. Gou, T. Yang, P. Wei, J. Qian, W. Xi and B. Z. Tang, *ACS Nano,* 2022, **16**, 6444.

8 M. Kumar, S. Perumbilavil, D. R. Vinayakumara, A. Goel, R. Philip and S. Kumar, *ACS Omega,* 2023, **8**, 45961.

9 M. A. M. Nogueira, A. S. Reyna, H. D. S. Souza, B. F. Lira, P. F. de Athayde-Filho and C. B. de Araújo, *J. Phys. Chem. B,* 2023, **127**, 7018.

10 B. Li, X. Lu, Y. Tian and D. Li, *Angew. Chem. Int. Ed.,* 2022, **61**, e202206755.

11 N. Liu, Z. Chen, W. Fan, J. Su, T. Lin, S. Xiao, J. Meng, J. He, J. J. Vittal and J. Jiang, *Angew. Chem. Int. Ed.,* 2022, **61**, e202115205.

12 J. Li, B. Li, X. Yao, W. Duan, W. Zhang, Y. Tian and D. Li, *Inorg. Chem.,* 2022, **61**, 19282.

13 B. Li, X. Yu, J. Wang, H. Tang, X. Sun, L. Cheng, H. Zhou, Y. Tian and D. Li, *Adv. Funct. Mater.,* 2023, **33**, 2305391.

14 J. B. Cho, D. Y. Park, K.-H. Lee, S. Kim, Y. S. Kim, M. S. Jeong and J. I. Jang, *Adv. Opt. Mater.,* 2024, **12**, 2301824, doi.org/10.1002/adom.202301824.

15 X. Zhang, S. Xiao, Z. Guo, B. Yuan, X. Wang, S. Zhang, Y. Shi, G. Xing, T. He and R. Chen, *J. Phys. Chem. Lett.,* 2023, **14**, 7581.

16 J. Wang, P. Lu, Y. Shuai, Z. Fan, Y. Li, X. Li, R. Hong, C. Tao, Q. Wang, H. Lin, Z. Han and D. Zhang, *ACS Appl. Nano Mater.,* 2023, **6**, 13421.

17 K. H. Wei, B. J. Chen, L. B. Zhang, H. R. Zhu and S. H. Fan, *Laser Phys. Lett.,* 2022, **19**, 056003.

18 Q. Fan, Z. Yan, H. Zhou, Y. Yao, Z. Wang, Y. Gao, Y. Wang, S. Lu, M. Liu and W. Ji, *J. Mater. Chem. C,* 2023, **11**, 5788.

19 J. Jiang, G. Niu, L. Sui, X. Wang, X. Zeng, Y. Zhang, L. Che, G. Wu, K. Yuan and X. Yang, *Adv. Opt. Mater.,* 2023, **11**, 2202634.

20 V. Pradeep Kumar, C. Pradeep, M. M. Raj Sha, P. Radhakrishnan and A. Mujeeb, *Opt. Laser Technol.,* 2023, **158**, 108809.

21 Z. Wei, Y. Pan, G. Hou, X. Ran, Z. Chi, Y. He, Y. Kuang, X. Wang, R. Liu and L. Guo, *ACS Appl. Mater. Interfaces,* 2022, **14**, 2452.

22 P. Chen, H. Liu, Y. Cui, C. Liu, Y. Li, Y. Gao, J. Cheng and T. He, *J. Phys. Chem. C,* 2023, **127**, 2464.

23 A. Bundulis, A. Berzina, V. V. Kim, B. Polyakov, A. Novikovs and R. A. Ganeev, *Nanomaterials,* 2023, **13**, 2320.

24 I. M. Gonçalves, A. Medda, A. J. A. Carvalho, C. L. A. V. Campos, S. Ghosh, A. S. L. Gomes and A. Patra, *J. Phys. Chem. C,* 2023, **127**, 16679.

25 W. L. F. Armarego and C. L. L. Chai, *Purification of Laboratory Chemicals (Sixth Ed.)*, Butterworth-Heinemann: Oxford, 2009; Ch. 5, 445.

26 S. Takahashi, Y. Kuroyama, K. Sonogashira and N. Hagihara, *Synthesis,* 1980, **8**, 627.

27 G. T. Dalton, M. P. Cifuentes, L. A. Watson, S. Petrie, R. Stranger, M. Samoc and M. G. Humphrey, *Inorg. Chem.,* 2009, **48**, 6534.

28 T. Ljungdahl, K. Pettersson, B. Albinsson, J. Mårtensson, *Eur. J. Org. Chem.,* 2006, 3087.

29 C.-Y. Wong, M. C. W. Chan, N. Zhu and C.-M. Che, *Organometallics,* 2004, **23**, 2263.

30 D. Touchard, P. Haquette, S. Guesmi, L. L. Pichon, A. Daridor, L. Toupet and P. H. Dixneuf, *Organometallics,* 1997, **16**, 3640.

31 S. K. Hurst, M. P. Cifuentes and M. G. Humphrey, *Organometallics,* 2002, **21**, 2353.

32 C. E. Powell, S. K. Hurst, J. P. Morrall, M. P. Cifuentes, R. L. Roberts, M. Samoc and M. G. Humphrey, *Organometallics,* 2007, **26**, 4456.

33 T. Schwich, A. Barlow, M. P. Cifuentes, J. Szeremeta, M. Samoc and M. G. Humphrey, *Chem. Eur. J.,* 2017, **23**, 8395.

34 K. A. Green, P. V. Simpson, T. C. Corkery, M. P. Cifuentes, M. Samoc and M. G. Humphrey, *Macromol. Rapid Commun.,* 2012, **33**, 573.

35 A. M. McDonagh, M. G. Humphrey, M. Samoc, B. Luther-Davies, S. Houbrechts, T. Wada, H. Sasabe and A. Persoons, *J. Am. Chem. Soc.,* 1999, **121**, 1405.

36 R. Evans, Z. Deng, A. K. Rogerson, A. S. McLachlan, J. J. Richards, M. Nilsson and G. A. Morris, *Angew. Chem. Int. Ed.,* 2013, **52**, 3199.

37 M. Holz and H. Weingartner, *J. Magn. Reson.,* 1991, **92**, 115.

38 M. Sheik-Bahae, A. Said, T. Wei, D. J. Hagan and E. W. van Stryland, *IEEE J. Quant. Electron.,* 1990, **26**, 760.

39 D. C. Hutchings, M. Sheik-Bahae, D. J. Hagan and E. W. Van Stryland, *Opt. Quantum Electron.,* 1992, **24**, 1.

40 M. Sheik-Bahae, in *Encyclopedia of Modern Optics,* Vol 4 (Eds: B. D. Guenther, D. G. Steel), Academic Press, London, 2018, 317.

41 D. S. Corrêa, L. De Boni, L. Misoguti, I. Cohanoschi, F. Hernandez and C. R. Mendonça, *Opt. Commun.,* 2007, **277**, 440.

42 S.-I. Tanaka, J. Miyazaki, D. K. Tiwari, T. Jin and Y. Inouye, *Angew. Chem. Int. Ed.,* 2011, **50**, 431.

43 N. Li, M. Echeverría, S. Moya, J. Ruiz and D. Astruc, *Inorg. Chem.,* 2014, **53**, 6954.

44 H.-F. Chow, M.-K. Ng, C.-W. Leung and G.-X. Wang, *J. Am. Chem. Soc.,* 2004, **126**, 12907.

45 J. Lim, M. Kostiainen, J. Maly, V. C. P. da Costa, O. Annunziata, G. M. Pavan and E. E. Simanek, *J. Am. Chem. Soc.,* 2013, **135**, 4660.

46 M. A. van Dongen, A. Desai, B. G. Orr, J. R. Baker and M. M. Banaszak Holl, *Polymer,* 2013, **54**, 4126.

47 M. J. Frisch, G. W. Trucks, H. B. Schlegel, G. E. Scuseria, M. A. Robb, J. R. Cheeseman, G. Scalmani, V. Barone, G. A. Petersson, H. Nakatsuji, X. Li, M. Caricato, A. V. Marenich, J. Bloino, B. G. Janesko, R. Gomperts, B. Mennucci, H. P. Hratchian, J. V. Ortiz, A. F. Izmaylov, J. L. Sonnenberg, D. Williams-Young, F. Ding, F. Lipparini, F. Egidi, J. Goings, B. Peng, A. Petrone, T. Henderson, D. Ranasinghe, V. G. Zakrzewski, J. Gao, N. Rega, G. Zheng, W. Liang, M. Hada, M. Ehara, K. Toyota, R. Fukuda, J. Hasegawa, M. Ishida, T. Nakajima, Y. Honda, O. Kitao, H. Nakai, T. Vreven, K. Throssell, J. A. Montgomery Jr., J. E. Peralta, F. Ogliaro, M. J. Bearpark, J. J. Heyd, E. N. Brothers, K. N. Kudin, N. Staroverov, T. A. Keith, R. Kobayashi, J. Normand, K. Raghavachari, A. P. Rendell, J. C. Burant, S. S. Iyengar, J. Tomasi, M. Cossi, J. M. Millam, M. Klene, C. Adamo, R. Cammi, J. W. Ochterski, R. L. Martin, K. Morokuma, O. Farkas, J. B. Foresman and D. J. Fox., Gaussian 16, Revision C.01, Gaussian, Inc., Wallingford CT, 2016.

48 W. Humphrey, A. Dalke and K. Schulten, *J. Molec. Graphics,* 1996, **14**, 33.

49 T. Lu and F. Chen, *J. Comput. Chem.,* 2012, **33**, 580.

50 K. Aidas, C. Angeli, K. L. Bak, V. Bakken, R. Bast, L. Boman, O. Christiansen, R. Cimiraglia, S. Coriani, P. Dahle, E. K. Dalskov, U. Ekström, T. Enevoldsen, J. J. Eriksen, P. Ettenhuber, B. Fernández, L. Ferrighi, H. Fliegl, L. Frediani, K. Hald, A. Halkier, C. Hättig, H. Heiberg, T.

Helgaker, A. C. Hennum, H. Hettema, E. Hjertenæs, S. Høst, I.-M. Høyvik, M. F. Iozzi, B. Jansik, H. J. Aa. Jensen, D. Jonsson, P. Jørgensen, J. Kauczor, S. Kirpekar, T. Kjærgaard, W. Klopper, S. Knecht, R. Kobayashi, H. Koch, J. Kongsted, A. Krapp, A. Kristensen, A. Ligabue, O. B. Lutnæs, J. I. Melo, K. V. Mikkelsen, R. H. Myhre, C. Neiss, C. B. Nielsen, P. Norman, J. Olsen, J. M. H. Olsen, A. Osted, M. J. Packer, F. Pawlowski, T. B. Pedersen, P. F. Provasi, S. Reine, Z. Rinkevicius, T. A. Ruden, K. Ruud, V. Rybkin, P. Salek, C. C. M. Samson, A. Sánchez de Merás, T. Saue, S. P. A. Sauer, B. Schimmelpfennig, K. Sneskov, A. H. Steindal, K. O. Sylvester-Hvid, P. R. Taylor, A. M. Teale, E. I. Tellgren, D. P. Tew, A. J. Thorvaldsen, L. Thøgersen, O. Vahtras, M. A. Watson, D. J. D. Wilson, M. Ziolkowski and H. Agren, *WIREs Comput. Mol. Sci.,* 2014, **4**, 269. doi: 10.1002/wcms.1172; Dalton, a Molecular Electronic Structure Program, Release Dalton2020.1, 2022: see http://daltonprogram.org/.

51 M. Samoc, G. T. Dalton, J. A. Gladysz, Q. Zheng, Y. Velkov, H. Ågren, P. Norman and M. G. Humphrey, *Inorg. Chem.,* 2008, **47**, 9946.

52 D. H. Friese, M. T. P. Beerepoot, M. Ringholm and K. Ruud, *J. Chem. Theory Comput.,* 2015, **11**, 1129.

53 X. Wang, D. Wang, J. Li, M. Zhang, D. Kang and P. Song, *ACS Omega,* 2022, **7**, 9743.

54 D. L. Silva, N. A. Murugan, J. Kongsted, Z. Rinkevicius, S. Canuto and H. Ågren, *J. Phys. Chem. B,* 2012, **116**, 8169.

55 M. T. P. Beerepoot, D. H. Friese, N. H. List, J. Kongsted and K. Ruud, *Phys. Chem. Chem. Phys.,* 2015, **17**, 193064.

56 K. Matczyszyn, J. Olesiak-Banska, K. Nakatani, P. Yu, N. A. Murugan, R. Zaleśny, A. Roztoczyńska, J. Bednarska, W. Bartkowiak, J. Kongsted, H. Ågren and M. Samoć, *J. Phys. Chem. B,* 2015, **119**, 1515.

57 P. Sałek, O. Vahtras, J. Guo, Y. Luo, T. Helgaker and H. Ågren, *Chem. Phys. Lett.,* 2003, **374**, 446.

58 A. Masunov and S. Tretiak, *J. Phys. Chem. B,* 2004, **108**, 899.

59 K. Zhao, L. Ferrighi, L. Frediani, C.-K. Wang, Y. Luo, *J. Chem. Phys.,* 2007, **126**, 204509.

60 F. Terenziani, C. Katan, E. Badaeva, S. Tretiak and M. Blanchard-Desce, *Adv. Mater.,* 2008, **20**, 4641.

61 K. A. Nguyen, P. N. Day and R. Pachter, *J. Phys. Chem. A,* 2009, **113**, 13943.

62 G. Kang, K. Nasiri Avanaki, M. A. Mosquera, R. K. Burdick, J. P. Villabona-Monsalve, T. Goodson III and G. C. Schatz, *J. Am. Chem. Soc.,* 2020, **142**, 10446.
